# Lifetime Probability of Developing Migraine: A Novel Epidemiological Perspective Based on Global Burden of Disease Data

**DOI:** 10.1155/prm/9739777

**Published:** 2026-08-23

**Authors:** Zhe Wang, Yuhao Li, Shuo Feng, Xinjing Zhao, Xinyue Yang, Lin Tian, Tianyu Gao, Yashuang Wu, Guoyu Zhou, Shengfeng Wang, Jing Han

**Affiliations:** ^1^ College of Acupuncture and Tuina, Shandong University of Traditional Chinese Medicine, Jinan, China, sdutcm.edu.cn; ^2^ Department of Epidemiology and Biostatistics, School of Public Health, Peking University, Beijing, China, pku.edu.cn; ^3^ Key Laboratory of Epidemiology of Major Diseases, Ministry of Education, Beijing, China, meb.gov.tr; ^4^ Department of Neurology, Qilu Hospital of Shandong University, Jinan, China, qiluhospital.com; ^5^ Department of Geriatric Medicine, Qilu Hospital of Shandong University, Jinan, China, qiluhospital.com; ^6^ Institute for Artificial Intelligence, Peking University, Beijing, China, pku.edu.cn; ^7^ Department of Acupuncture, Affiliated Hospital of Shandong University of Traditional Chinese Medicine, Jinan, China, sdutcm.edu.cn

**Keywords:** burden, epidemiology, global, lifetime risk, migraine

## Abstract

Migraine affects over one billion people worldwide and imposes substantial burdens, yet the lifetime risk of developing migraine has been understudied. Using Global Burden of Disease 2021 data, we estimated age‐, sex‐, and location‐specific lifetime probabilities with the adjusted‐for‐multiple‐primaries method, which accounts for competing mortality and life expectancy. Trends were quantified by average annual percentage change, socioeconomic inequality by concentration indices, and future trajectories with autoregressive integrated moving average models. In 2021, the global lifetime probability of migraine was 51.53% (95% CI: 51.51%–51.55%); females faced a higher risk than males (59.84% vs. 41.99%; ratio: 1.42). Regional estimates ranged from 60.58% in Western Europe to 35.63% in Eastern Sub‐Saharan Africa, paralleling the sociodemographic index gradient (high SDI 55.74% vs. low SDI 45.13%). Risk accumulated mainly during early and middle adulthood. The concentration index declined from 0.35 in 1990 to 0.27 in 2021, indicating reduced socioeconomic disparities, while projections suggest global lifetime risk will rise to 57.18% by 2050. Roughly half the world’s population is therefore expected to experience migraine during their lives, with pronounced heterogeneity by sex, region, and socioeconomic level, underscoring the need for targeted public‐health strategies, particularly for younger individuals during high‐riskperiods.

## 1. Introduction

Migraine, a prevalent neurological disorder, affects over one billion people worldwide, generating substantial individual, economic, and socioeconomic burdens [1]. Approximately 14% of the global population experiences migraine annually, making it the second leading contributor to the global burden of neurological diseases [1–3]. This debilitating condition is characterized by recurrent episodes of moderate to severe headaches, frequently accompanied by heightened sensitivity to light and sound, as well as gastrointestinal disturbances [4, 5]. Various factors can trigger migraine attacks, including stress, exhaustion, and specific dietary elements, while the burden tends to increase with urbanization and lifestyle changes [1, 6].

Estimates of lifetime risk (defined as the cumulative probability of a disease developing in a person of a given age and sex during that person’s remaining lifespan, after accounting for competing risks of death) provide a measure of disease risk in large populations [7]. Estimates of lifetime risk of migraine may be useful for the long‐term planning of health systems. While existing research has explored migraine’s global impact [8, 9], there is a notable gap in studies focusing on the lifetime risk.

In this paper, we aim to quantify the lifetime risk of developing migraine using data from the Global Burden of Disease (GBD) Study 2021. We examine how this risk varies by age, sex, geographical region, and Socioeconomic Index (SDI) levels. These findings will contribute to a better understanding of the global distribution of migraine burden and inform targeted public health interventions for high‐risk populations.

## 2. Methods

### 2.1. Data Source

This research employed an ecological study design based on secondary aggregated data from the GBD Study 2021. The GBD 2021 results, accessible through the GBD Collaborative Network website (https://www.healthdata.org/), provide the most up‐to‐date estimates for 369 diseases, injuries, and impairments, as well as 88 risk factors, across 204 countries and territories grouped into 21 regions [8]. Through the GBD query tool, we collected age‐, sex‐, and location‐specific data on migraine incidence and mortality rates, along with total population and all‐cause mortality data from 1990 to 2021, covering all 21 GBD regions and 204 countries/territories. This study used de‐identified, aggregated data and did not require ethical approval. Detailed methodology is documented in GBD publications [10, 11].

The SDI, a composite measure ranging from 0 (*lowest development*) to 1 (*highest development*), is calculated as the geometric mean of three indicators: fertility rate among those under 25 years old, educational attainment for those aged 15 and above, and per capita income. Based on SDI values, countries were categorized into five strata by the GBD and World Bank standards: low (< 0.46), low–middle (0.46–0.64), middle (0.65–0.74), high–middle (0.75–0.85), and high (> 0.85) [12].

### 2.2. Disease Definition

In the GBD 2021 classification system, headache disorders are categorized as Level 3 conditions under neurological disorders (Level 2) and noncommunicable diseases (Level 1). Migraine is specifically identified as a distinct entity at Level 4 within the headache disorders group. According to the International Classification of Diseases (ICD), migraine is coded as 346–346.93 in ICD‐9 and G43‐G43.919 in ICD‐10 [13].

### 2.3. Statistical Analysis

We estimated the lifetime risk of migraine across 204 countries/territories for the total population and by sex using the adjusted‐for‐multiple‐primaries method [14, 15]. This approach accounts for competing mortality risks and adjusts for multiple primary events in incidence data. We set the migraine‐specific mortality rate to zero, an assumption justified by the clinical reality that migraine rarely causes direct mortality [16]. Although research indicates migraine may be associated with increased risk of certain cardiovascular and cerebrovascular events [17, 18], these indirect effects are typically reflected in all‐cause mortality data and incorporated as competing risks in our calculations. Age‐specific incidence, mortality, and all‐cause mortality data were stratified by 5‐year age groups to calculate sex‐specific lifetime risks of developing migraine, representing the probability from the initial age group. The 95% confidence interval (CI) for lifetime risk was estimated using Poisson and binomial distributions (see Supporting Methods).

Temporal trends in lifetime risk were assessed using average annual percentage change (AAPC) and corresponding 95% CIs, calculated using Joinpoint Trend Analysis Software Version 5.3.0.0 (Statistical Research and Applications Branch, National Cancer Institute, Calverton, MD, USA). Negative and positive AAPC values indicate decreasing and increasing trends, respectively. For future projections (2021–2050), we employed an autoregressive integrated moving average (ARIMA) model, with the optimal ARIMA(p,d,q) specification automatically selected using the auto.arima() function in *R* [19, 20].

Sex disparities were evaluated by calculating female‐to‐male lifetime risk ratios and their 95% CIs through 100,000 resampling iterations, assuming uniform distribution within the given 95% CIs of lifetime incidence risk.

To assess inequalities in migraine burden across development levels, we calculated concentration indices ranging from −1 to 1. Negative values indicate burden concentration in low‐SDI regions, while positive values suggest concentration in high‐SDI regions [21, 22]. We also computed Spearman correlation coefficients between lifetime risk and SDI. Frontier analysis was applied to quantitatively evaluate the relationship between migraine burden and sociodemographic development, determining achievable lifetime risk levels based on SDI‐measured development status, using free disposal hull analysis on 1000 bootstrapped samples with LOESS regression to generate smoothed frontier curves [23] (see Supporting Methods).

All statistical analyses were conducted using *R* Version 4.4.2 (R Foundation for Statistical Computing, Vienna, Austria). The maps were generated utilizing the *R* package “maps” Version 3.4.2. All hypothesis tests were two‐tailed and considered significant at a *p* value < 0.05.

## 3. Results

### 3.1. Global Burden

Analysis of global migraine patterns revealed a substantial lifetime risk, with 51.53% (95% CI: 51.51%–51.55%) of individuals experiencing migraine during their lifetime. Sex‐specific analysis unveiled a marked disparity, with females demonstrating significantly higher lifetime risk (59.84%, 95% CI: 59.81%–59.86%) compared to males (41.99%, 95% CI: 41.96%–42.02%), indicating that females are approximately 1.42 (95% CI: 1.42–1.43) times more likely to experience migraine during their lifetime (Table 1). CIs calculated using the binomial distribution were consistent with those derived from the Poisson distribution.

**TABLE 1:** Lifetime risks (%) of developing migraine within selected age intervals by sex in 2021.

Population	Birth to death	10 years to death	20 years to death	30 years to death	40 years to death	50 years to death	60 years to death	70 years to death	80 years to death
*Both sexes*									
Global	51.53 (51.51–51.55)	44.06 (44.04–44.08)	28.15 (28.13–28.16)	18.29 (18.28–18.31)	10.32 (10.31–10.33)	5.21 (5.21–5.22)	2.22 (2.22–2.22)	0.99 (0.99–0.99)	0.30 (0.30–0.31)
High SDI	55.74 (55.69–55.79)	48.26 (48.21–48.30)	28.98 (28.94–29.03)	18.09 (18.05–18.12)	9.94 (9.91–9.97)	4.88 (4.86–4.90)	2.18 (2.17–2.18)	1.13 (1.13–1.14)	0.45 (0.45–0.45)
High–middle SDI	52.06 (52.01–52.11)	45.62 (45.57–45.66)	29.87 (29.83–29.91)	19.72 (19.68–19.76)	11.46 (11.43–11.49)	6.04 (6.02–6.06)	2.68 (2.67–2.69)	1.23 (1.22–1.23)	0.38 (0.38–0.38)
Middle SDI	53.40 (53.36–53.43)	45.51 (45.47–45.54)	29.20 (29.17–29.23)	19.16 (19.13–19.18)	10.94 (10.92–10.96)	5.66 (5.65–5.67)	2.45 (2.45–2.46)	1.08 (1.08–1.08)	0.31 (0.31–0.31)
Low–middle SDI	52.29 (52.26–52.33)	43.90 (43.86–43.93)	27.71 (27.67–27.74)	17.86 (17.84–17.89)	9.85 (9.83–9.88)	4.76 (4.74–4.77)	1.88 (1.87–1.88)	0.73 (0.72–0.73)	0.17 (0.17–0.17)
Low SDI	45.13 (45.07–45.19)	38.77 (38.71–38.82)	24.98 (24.93–25.03)	16.11 (16.06–16.15)	8.92 (8.89–8.95)	4.27 (4.25–4.29)	1.62 (1.62–1.63)	0.61 (0.60–0.61)	0.13 (0.12–0.13)

*Males*									
Global	41.99 (41.96–42.02)	36.30 (36.27–36.33)	23.41 (23.39–23.44)	15.34 (15.32–15.36)	8.79 (8.77–8.81)	4.49 (4.49–4.50)	1.93 (1.93–1.94)	0.82 (0.82–0.82)	0.22 (0.22–0.22)
High SDI	42.44 (42.35–42.53)	36.96 (36.88–37.05)	23.54 (23.47–23.62)	15.22 (15.15–15.28)	8.50 (8.45–8.55)	4.34 (4.31–4.36)	2.01 (2.00–2.02)	1.04 (1.03–1.04)	0.38 (0.37–0.38)
High–middle SDI	41.12 (41.04–41.20)	36.27 (36.19–36.35)	24.16 (24.09–24.23)	16.22 (16.16–16.28)	9.62 (9.58–9.67)	5.12 (5.10–5.14)	2.30 (2.29–2.31)	1.01 (1.00–1.01)	0.28 (0.28–0.28)
Middle SDI	43.50 (43.44–43.56)	37.60 (37.55–37.66)	24.32 (24.26–24.37)	16.06 (16.02–16.11)	9.35 (9.32–9.38)	4.86 (4.84–4.87)	2.10 (2.09–2.11)	0.86 (0.86–0.87)	0.22 (0.22–0.22)
Low–middle SDI	44.11 (44.05–44.17)	37.64 (37.59–37.70)	23.97 (23.91–24.02)	15.54 (15.49–15.58)	8.72 (8.69–8.75)	4.31 (4.30–4.33)	1.72 (1.71–1.73)	0.62 (0.62–0.62)	0.13 (0.13–0.13)
Low SDI	37.77 (37.69–37.86)	32.74 (32.66–32.82)	21.20 (21.12–21.28)	13.79 (13.73–13.85)	7.73 (7.69–7.77)	3.75 (3.73–3.77)	1.45 (1.44–1.46)	0.51 (0.50–0.51)	0.09 (0.09–0.09)

*Females*									
Global	59.84 (59.81–59.86)	50.50 (50.48–50.53)	31.65 (31.62–31.67)	20.30 (20.28–20.32)	11.27 (11.26–11.29)	5.63 (5.62–5.64)	2.39 (2.38–2.39)	1.11 (1.10–1.11)	0.36 (0.36–0.36)
High SDI	66.57 (66.52–66.62)	57.02 (56.98–57.07)	32.30 (32.26–32.34)	19.44 (19.40–19.48)	10.43 (10.39–10.46)	4.95 (4.92–4.97)	2.14 (2.13–2.15)	1.11 (1.11–1.12)	0.47 (0.46–0.47)
High–middle SDI	61.63 (61.58–61.68)	53.46 (53.41–53.51)	34.06 (34.01–34.11)	21.92 (21.88–21.97)	12.44 (12.40–12.47)	6.46 (6.44–6.49)	2.83 (2.81–2.84)	1.32 (1.32–1.33)	0.43 (0.43–0.43)
Middle SDI	62.04 (62.00–62.08)	52.03 (51.99–52.07)	32.75 (32.72–32.79)	21.21 (21.18–21.24)	11.90 (11.87–11.93)	6.12 (6.10–6.14)	2.67 (2.66–2.68)	1.23 (1.23–1.24)	0.38 (0.38–0.38)
Low–middle SDI	59.51 (59.46–59.55)	49.10 (49.05–49.14)	30.49 (30.44–30.53)	19.49 (19.45–19.53)	10.59 (10.56–10.62)	5.04 (5.02–5.06)	1.99 (1.98–2.00)	0.82 (0.81–0.82)	0.21 (0.21–0.21)
Low SDI	51.74 (51.66–51.82)	44.00 (43.93–44.07)	28.04 (27.97–28.11)	17.93 (17.87–17.99)	9.88 (9.83–9.93)	4.71 (4.68–4.74)	1.79 (1.78–1.80)	0.72 (0.71–0.72)	0.16 (0.16–0.17)

### 3.2. Regional and Socioeconomic Variations

**FIGURE 1:**
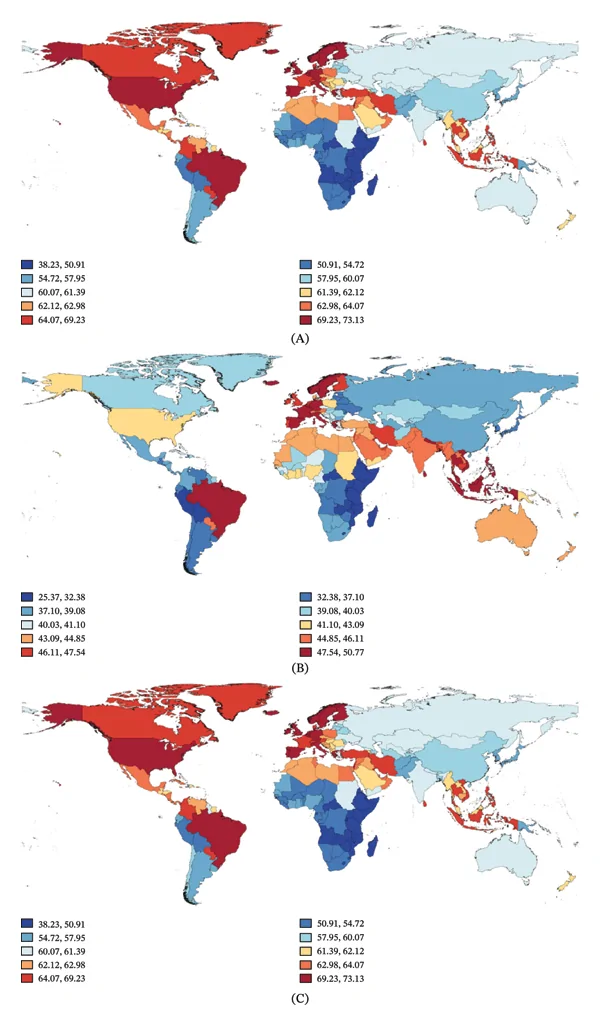
Lifetime risks of developing migraine in 2021. (A) Global. (B) Males. (C) Females.

Geographical analysis revealed notable regional differences in lifetime risk. Western Europe exhibited the highest lifetime risk at 60.58% (95% CI: 60.53%–60.64%), with several European countries showing particularly high rates, including Italy (63.14%, 95% CI: 63.00%–63.28%), Belgium (62.78%, 95% CI: 62.43%–63.12%), Norway (61.47%, 95% CI: 60.96%–61.98%), and Sweden (61.19%, 95% CI: 60.81%–61.57%). This was followed by high‐income North America (57.81%, 95% CI: 57.72%–57.89%) and Southeast Asia (56.74%, 95% CI: 56.68%–56.80%). In contrast, Eastern Sub‐Saharan Africa showed the lowest risk (35.63%, 95% CI: 35.53%–35.73%), with countries such as South Sudan (32.08%, 95% CI: 31.43%–32.73%), Somalia (32.59%, 95% CI: 32.17%–33.01%), and Ethiopia (34.76%, 95% CI: 34.54%–34.98%) reporting the lowest rates globally (Table 1, S1‐S3, Figure 1).

**FIGURE 2:**
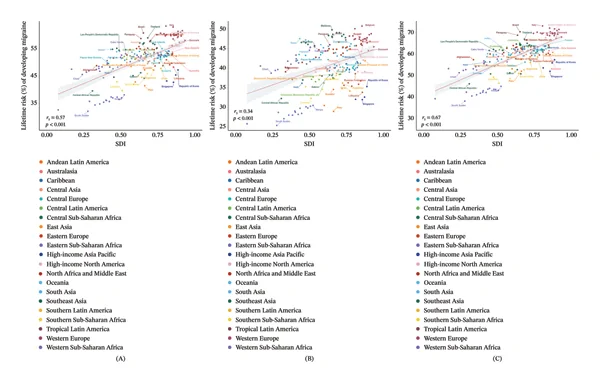
Association between lifetime risks of developing migraine and SDI in 2021. (A) Global. (B) Men. (C) Females.

Correlation analysis revealed significant positive associations between lifetime risk of migraine and SDI across all populations (*r*_*s*_ = 0.57, *p* < 0.001), with consistent patterns observed in both males (*r*_*s*_ = 0.34, *p* < 0.001) and females (*r*_*s*_ = 0.67, *p* < 0.001) (Figure 2). At the SDI level, a general gradient was observed. High‐SDI regions reported the highest lifetime risk (55.74%, 95% CI: 55.69%–55.79%), followed by middle‐SDI (53.40%, 95% CI: 53.36%–53.43%), low–middle‐SDI (52.29%, 95% CI: 52.26%–52.33%), high–middle‐SDI (52.06%, 95% CI: 52.01%–52.11%), and low‐SDI regions showing the lowest risk (45.13%, 95% CI: 45.07%–45.19%). These gradients may partly reflect underdiagnosis in resource‐limited settings rather than true biological differences (Table 1).

### 3.3. Age‐Conditional Patterns

**FIGURE 3:**
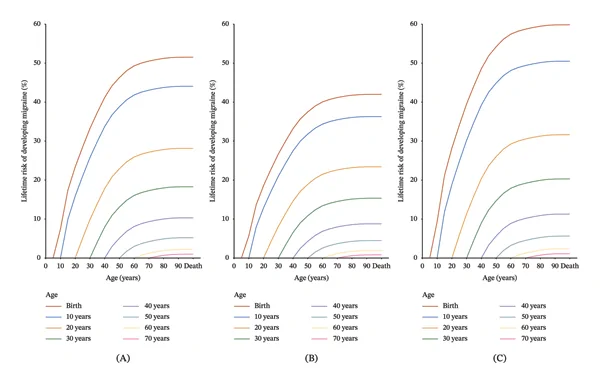
Global lifetime risks of developing migraine in men and women by age at diagnosis in 2021. (A) Global. (B) Males. (C) Females. Curves are represented for baseline ages at birth, 10, 20, 30, 40, 50, 60, and 70 years of migraine‐free life. Risks until death represent lifetime risk from the baseline age.

The age‐conditional remaining risks of developing migraine varied by sex (Table 1) and areas of the world in 2021 (Tables S1‐S3; Figures S1–S3). The lifetime risk of developing migraine exhibited a distinct age‐dependent pattern, with younger individuals showing higher lifetime risks compared to older populations. The risk accumulation predominantly occurred between ages 10 and 50, with the rate of increase gradually plateauing after age 50. While females had consistently higher remaining risks throughout life compared to males, both sexes followed similar age‐related patterns (Figure 3).

### 3.4. Sex Disparities

Analysis of female‐to‐male ratios in migraine lifetime risk revealed distinct temporal and age‐specific patterns across different SDI levels (Figure S4). Temporally, the female‐to‐male ratio showed a declining trend globally and across most SDI levels, except for low and low‐middle‐SDI regions. In 1990, the global female‐to‐male ratio was 1.46 (95% CI: 1.46–1.47), indicating that females were 46% more likely to experience migraine during their lifetime compared to males. This disparity showed a slight decrease over the study period, with the global female‐to‐male ratio declining to 1.42 (95% CI: 1.42–1.43) by 2021 (Table S4). Age‐specific analysis demonstrated a J‐shaped curve in the female‐to‐male ratio across all SDI levels. The ratio exhibited a declining trend before age 65, followed by a substantial increase thereafter (Figure S4; Table S5).

### 3.5. Temporal Trends and Projections

**FIGURE 4:**
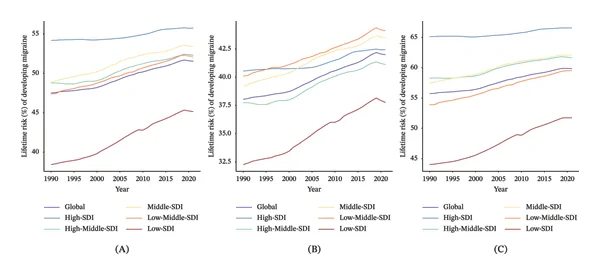
Trends in lifetime risk of migraine by SDI level, 1990–2021. (A) Global. (B) Males. (C) Females.

From 1990 to 2021, lifetime risk of migraine showed an increasing trend across all SDI levels (Figure 4). The high‐SDI regions maintained the highest lifetime risk throughout the study period, rising from 54.18% (95% CI: 54.12%–54.23%) to 55.74% (95% CI: 55.69%–55.79%). The low‐SDI regions, despite having the lowest lifetime risk, demonstrated the steepest increase from 38.42% (95% CI: 38.34%–38.50%) to 45.13% (95% CI: 45.07%–45.19%) (Tables S6–S8).

**FIGURE 5:**
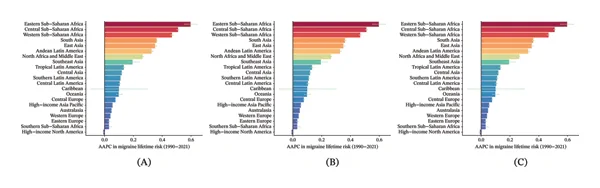
Regional patterns of AAPC in migraine lifetime risk, 1990–2021. (A) Global. (B) Males. (C) Females.

Consistent with these trends, the AAPC in migraine lifetime risk showed a gradient distribution corresponding to SDI levels, with higher values in low‐SDI regions (0.51%, 95% CI: 0.47%–0.56%) and lower values in high‐SDI regions (0.09%,95% CI: 0.09%–0.10%). Globally, the AAPC was 0.26% (95% CI: 0.26%–0.27%). The highest AAPC was observed in Eastern Sub‐Saharan Africa (0.62%,95% CI: 0.51%–0.75%), followed by Central Sub‐Saharan Africa (0.51%,95% CI: 0.50%–0.53%) and Western Sub‐Saharan Africa (0.49%,95% CI: 0.48%–0.50%). In contrast, high‐income North America (0.00%,95% CI: −0.01%‐0.00%), Eastern Europe (0.04%,95% CI: 0.02%–0.05%), and Western Europe (0.06%,95% CI: 0.06%–0.06%) showed the lowest AAPC values during 1990–2021 (Figure 5, Tables S6–S8).

**FIGURE 6:**
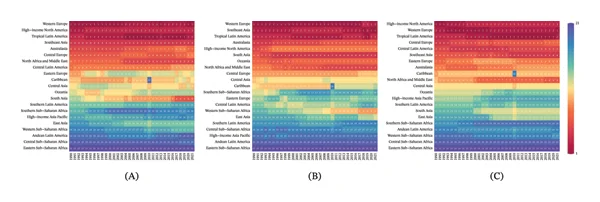
Temporal trends of regional migraine risk rankings, 1990–2021. (A) Global. (B) Males. (C) Females.

These changes in lifetime risk were reflected in the regional rankings (Figure 6). Western Europe, high‐income North America, tropical Latin America, and Southeast Asia maintained consistently high rankings throughout the study period. Eastern Europe and Southern Sub‐Saharan Africa showed slightly declining trends in their rankings. East Asia and Western Sub‐Saharan Africa demonstrated upward trends. Notably, South Asia exhibited the most substantial increase, rising from Rank 13 in 1990 to Rank 6 by 2021.

**FIGURE 7:**
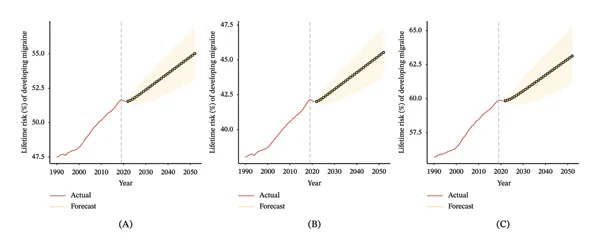
ARIMA(1,1,0)‐based forecast of global migraine lifetime risk from 1990 to 2050. (A) Global. (B) Males. (C) Females. The widening confidence intervals in later projection years reflect cumulative forecast uncertainty inherent to long‐term ARIMA modeling.

Based on the ARIMA (1,1,0) model forecast, the global lifetime risk of migraine is projected to increase from 53.63% (95% CI: 52.18%–55.08%) in 2030 to 57.18% (95% CI: 51.26%–63.10%) in 2050 (Figure 7; Table S9).

### 3.6. Health Inequalities

**FIGURE 8:**
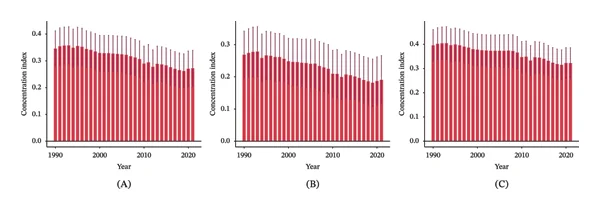
Temporal trends in concentration index of migraine lifetime risk, 1990–2021. (A) Global. (B) Males. (C) Females.

The concentration index of migraine lifetime risk remained positive from 1990 to 2021, indicating that the burden of migraine was consistently concentrated in regions with higher socioeconomic development. However, this index showed a gradual declining trend over the 30‐year period, decreasing from 0.35 (95% CI: 0.28, 0.41) in 1990 to 0.27 (95% CI: 0.21, 0.34) in 2021, though this may partly reflect rising burden in low‐SDI regions rather than genuine equity improvement (Figure 8; Tables S10–S12).

### 3.7. Frontier Analysis

**FIGURE 9:**
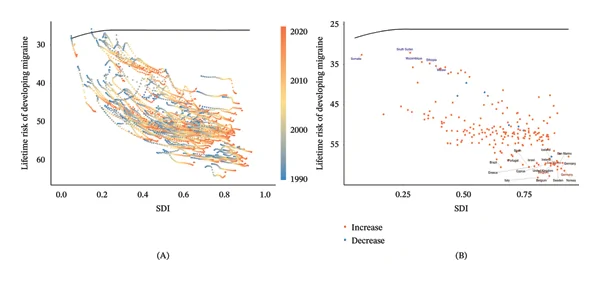
Frontier analysis of migraine lifetime risk in 204 countries and regions in 2021. The frontier curve represents the theoretical minimum lifetime risk achievable at each SDI level. The top 15 countries furthest from the frontier are marked in black; countries with low SDI (< 0.5) and nearest to the frontier are marked in blue; countries with high SDI (> 0.85) and furthest from the frontier are marked in red. Red dots indicate an increase in lifetime risk from 1990 to 2021; blue dots indicate a decrease in lifetime risk from 1990 to 2021.

A comprehensive frontier analysis of migraine lifetime risk across 204 countries and territories from 1990 to 2021 revealed substantial heterogeneity in disease burden relative to socioeconomic development. The visual analysis identified 15 countries with markedly elevated lifetime risks and significant deviations from the theoretical frontier, including several high‐SDI nations (Norway, Germany, Belgium, and Italy) and middle‐SDI countries (such as Brazil). Several low‐SDI countries (< 0.5), particularly in Eastern Africa (Somalia, Ethiopia, and South Sudan), positioned closer to the frontier, though this may partly reflect underdiagnosis rather than truly lower migraine burden (Figure 9).

## 4. Discussion

In this systematic analysis of global migraine patterns, we estimated that the lifetime probability of developing migraine reached 51.53% (95% CI: 51.51%–51.55%) in 2021. To our knowledge, this is the first assessment of the lifetime risk of developing migraine in a global population. Lifetime risk assessment offers a distinct perspective by quantifying the cumulative probability of developing migraine during one’s remaining lifespan while accounting for competing mortality risks [14, 15, 24], making it particularly valuable for understanding the long‐term population burden and facilitating risk communication in clinical settings.

By calculating age‐conditional lifetime risk, we provide a more intuitive measure of disease probability for specific age groups. Our analysis revealed distinct age‐dependent patterns in migraine risk accumulation. The lifetime risk primarily increased during early and middle adulthood, with the rate of increase plateauing in later years. Individuals remaining migraine‐free by age 50 showed markedly low lifetime risk; late‐onset migraine is rare. This age‐specific pattern aligns with previous findings based on traditional epidemiological metrics, where migraine incidence peaks in adolescence (10–14 years), while prevalence and disability‐adjusted life years (DALYs) reach their maximum in middle age (40–44 years) [9]. The concentration of lifetime risk in younger age groups likely reflects the complex interplay of biological and environmental factors during these critical life stages, including hormonal changes during puberty, increased academic and occupational stressors, and lifestyle modifications associated with urbanization [25]. The concentration of migraine onset risk during developmental and working‐age periods highlights the critical need for age‐targeted preventive interventions. These lifetime risk estimates offer clinically relevant diagnostic context for neurologists, particularly informing differential diagnosis in older patients where true migraine onset after age 50 is statistically uncommon. Incorporating age‐conditional lifetime risk into clinical assessment may enhance diagnostic accuracy and resource allocation across the lifespan.

Our analysis demonstrated marked sex disparities in migraine lifetime risk, with females showing a significantly higher probability (59.84%) compared to males (41.99%), yielding a female‐to‐male ratio of 1.42 in 2021. These patterns align with previous epidemiological studies based on incidence and prevalence, where female predominance has been consistently documented [8, 26, 27]. The higher lifetime risk in females likely reflects well‐established biological mechanisms, such as differences in brain structure, genetic polymorphisms, neuronal pathways, and hormonal influences, including estrogen fluctuations [28, 29]. Females typically demonstrate higher pain sensitivity and greater heterogeneity in pain responses than males [30], which may be attributed to the complex interplay of biological, psychological, and sociocultural factors that influence females’ sensitivity to environmental stressors [30]. Consistent with incidence trends, this ratio exhibited a slight decline from 1.46 in 1990, suggesting a gradual narrowing of the sex gap [9]. The observed temporal reduction in sex disparity, albeit modest, may reflect improving healthcare access and increasing awareness of migraine among males. Notably, this declining trend was primarily observed in high‐ and middle‐SDI regions, while remaining relatively stable in low‐SDI countries. In workers, migraines are mostly associated with strenuous physical work in males [29]. This regional heterogeneity may reflect escalating health challenges faced by males in rapidly developing societies, including intense physical labor, increased occupational stress, and poor awareness of personal health management [31, 32].

Age‐stratified analysis demonstrated a J‐shaped curve in the female‐to‐male ratio, characterized by decreasing sex disparity before age 65 followed by a subsequent increase. However, the clinical significance of this post‐65 increase is attenuated by the notably low absolute lifetime risk in this demographic. Our findings indicate an overall trajectory of diminishing sex disparity in lifetime migraine risk with advancing age. Females exhibit more pronounced age‐related fluctuations in migraine prevalence compared to males. These sex‐specific variations correlate with established hormonal influences, particularly estrogen dynamics, which manifest at puberty onset, intensify during perimenopause, and diminish in late menopause [33]. However, the sharp post‐age‐65 increase in the female‐to‐male ratio, particularly pronounced in low‐SDI regions, may partly reflect differential survival [34], as higher male mortality in older age groups suppresses male lifetime risk estimates, thereby reducing the male denominator in sex ratio calculations.

Our trends analysis revealed a distinctive pattern in migraine lifetime risk across SDI levels from 1990 to 2021, contrasting with previously reported incidence‐based trends. Notably, while traditional epidemiological metrics (ASIR and ASPR) showed the highest increase in middle‐SDI regions and relative stability in low‐SDI areas [9], lifetime risk demonstrated an inverse gradient in growth rates. Low‐SDI regions, despite having the lowest absolute lifetime risk, exhibited the steepest increase (AAPC: 0.51 [95% CI: 0.47–0.56]), rising substantially from 38.42% to 45.13%. This was followed by a gradual decrease in growth rates with increasing SDI levels, culminating in minimal changes in high‐SDI regions (AAPC: 0.09% [95% CI: 0.09–0.10]). The discrepancy between lifetime risk and traditional metrics may be attributed to their fundamental differences in measurement. While age‐standardized rates provide a snapshot of disease burden at specific time points, lifetime risk incorporates competing mortality risks and life expectancy [14, 15, 24], thereby capturing the cumulative impact of improved healthcare access and increasing life expectancy in developing regions. Our ARIMA model projects this trend to continue, with global lifetime risk reaching 57.18% (95% CI: 51.26%–63.10%) by 2050, predominantly driven by sustained growth in currently developing regions. These findings highlight the importance of considering multiple epidemiological measures when assessing disease burden, as they may reveal different aspects of health system development and disease patterns across socioeconomic gradients.

Geographic analysis revealed substantial heterogeneity in migraine lifetime risk across regions and SDI levels. Our findings demonstrate a general socioeconomic gradient, with high‐SDI regions consistently exhibiting the highest lifetime risk, followed by a sequential decrease through middle‐SDI, low–middle‐SDI, high–middle‐SDI, to low‐SDI regions. This gradient aligns with the positive correlation observed between lifetime risk and SDI across all populations (*p* < 0.001). However, this relationship reveals interesting complexities when examined at the regional level. Western Europe maintained the highest regional lifetime risk, with particularly elevated rates in countries such as Italy, Belgium, and Norway, a trend likely driven by lifestyle changes, accelerated industrialization, and increased mental stress, as well as superior healthcare infrastructure enabling better diagnosis [9]. Conversely, Eastern Sub‐Saharan Africa demonstrated the lowest lifetime risk, with countries such as South Sudan and Somalia reporting the lowest global rates. Interestingly, Southeast Asia showed unexpectedly high lifetime risk despite its middle‐SDI classification, suggesting that factors beyond socioeconomic development influence regional migraine patterns. This exception suggests potential roles for genetic predisposition, environmental factors, or regional healthcare system efficiencies [35].

This phenomenon was further confirmed by our frontier analysis, which quantified the relationship between lifetime risk and socioeconomic development. The analysis revealed significant deviations from theoretical expectations based on SDI levels, with several high‐SDI nations positioned far from the frontier, indicating suboptimal management of migraine burden despite abundant healthcare resources. Conversely, certain low‐SDI countries in Eastern Africa were positioned closer to the frontier, achieving relatively better outcomes despite limited resources. However, these patterns must be interpreted cautiously, as limited healthcare infrastructure in low‐SDI regions may lead to underdiagnosis [36, 37], potentially creating an artificial impression of better performance. These findings highlight the need for tailored prevention and therapeutic strategies that consider the multifaceted nature of migraine etiology and the specific healthcare contexts of different regions [38].

Several limitations should be acknowledged, although efforts were made to mitigate their impact. First, while the GBD data rely on varying diagnostic criteria across countries, potentially introducing case definition heterogeneity, the GBD methodology employs rigorous statistical approaches to harmonize data from diverse sources and reconcile differences in case definitions [39]. Nevertheless, cultural differences in pain perception and reporting exist [40, 41], which may affect cross‐country comparisons. Second, despite GBD’s comprehensive data integration, underdiagnosis may be more prevalent in regions with limited healthcare access, disproportionately affecting estimates in low‐SDI countries. Third, the GBD framework does not distinguish between first‐onset and recurrent episodes in migraine incidence data, as individuals who never experienced migraine and those with remitted inactive migraine are classified into a single “no active migraine” state [42]. Following approaches adopted in other GBD‐based neurological lifetime risk analyses [24], we assumed that individuals with prior migraine history carry the same incidence risk as those who have never had migraine, a limitation similarly acknowledged in GBD‐based neurological epidemiology studies [42, 43]. Finally, this study is subject to ecological fallacy, where associations observed at the population level may not necessarily hold true at the individual level [44]. However, ecological approaches can serve as critical complementary evidence, especially when individual‐level associations may be confounded by unmeasured variables [45]. Future research should focus on developing more standardized diagnostic approaches across diverse healthcare settings, improving data collection in underserved regions and refining analytical methods to better incorporate the complex interactions between migraine and comorbid conditions in lifetime risk assessment.

## 5. Conclusion

This global analysis demonstrates that approximately half the world’s population (51.53%) will develop migraine during their lifetime, with females having a 1.4 times higher risk than males. Substantial geographical heterogeneity exists, with an inverse gradient in growth rates across SDI levels suggesting an ongoing epidemiological transition. Projections indicate continued increase to 57.18% by 2050, emphasizing migraine’s significance as a global health priority requiring context‐specific prevention and management strategies.

## Author Contributions

Zhe Wang conducted data analysis, visualization, and primary drafting of the manuscript; Yuhao Li contributed to data acquisition, visualization, and data analysis; Shuo Feng, Jing Han, and Xinjing Zhao were responsible for data visualization and manuscript writing; Xinyue Yang, Lin Tian, Tianyu Gao, and Yashuang Wu performed data analysis and manuscript writing; Guoyu Zhou, Shengfeng Wang, and Jing Han designed the study, conceptualized the research, and critically reviewed the manuscript.

## Funding

This work was supported by the National Natural Science Foundation of China (Grant no. 82374572) and the Taishan Scholar Program (Grant no. tsqn202312376).

## Disclosure

All authors reviewed and approved the final manuscript and are accountable for all aspects of the work, including its accuracy and integrity.

## Ethics Statement

This study used de‐identified, aggregated data and did not require ethical approval.

## Consent

The authors have nothing to report.

## Conflicts of Interest

The authors declare no conflicts of interest.

## Supporting Information

Additional supporting information can be found online in the Supporting Information section.

## Supporting information


**Supporting Information 1** Appendix A: Supplementary Methods: A short introduction about the methods of lifetime risk of developing migraine. A short introduction about the methods of frontier analysis.

**Supporting Information 2** Appendix B: Supplementary Tables and Figures: Figure S1: Lifetime and age‐conditional probability of developing migraine in the 21 areas of the world for both sexes, 2021. Figure S2: Lifetime and age‐conditional probability of developing migraine in the 21 areas of the world for males, 2021. Figure S3: Lifetime and age‐conditional probability of developing migraine in the 21 areas of the world for females, 2021. Figure S4: Female‐to‐male ratios of migraine lifetime risk by temporal and age patterns across SDI levels. Supplementary Tables: Table S1: Age‐conditional probability (%) of developing migraine, both sexes, 2021. Table S2: Age‐conditional probability (%) of developing migraine, males, 2021. Table S3: Age‐conditional probability (%) of developing migraine, females, 2021. Table S4: Female‐to‐male ratios of migraine lifetime risk with 95% CIs, 2021. Table S5: Age‐specific female‐to‐male ratios of migraine lifetime risk with 95% CIs, 2021. Table S6: Regional AAPC (%) in migraine lifetime risk with 95% CIs, 1990–2021, both sexes. Table S7: Regional AAPC (%) in migraine lifetime risk with 95% CIs, 1990–2021, males. Table S8: Regional AAPC (%) in migraine lifetime risk with 95% CIs, 1990–2021, females. Table S9: Projected global migraine lifetime risk (%) with 95% CIs, 2020–2050. Table S10: Concentration index of migraine lifetime risk (%) with 95% CIs, 1990–2021, both sexes. Table S11: Concentration index of migraine lifetime risk (%) with 95% CIs, 1990–2021, males. Table S12: Concentration index of migraine lifetime risk (%) with 95% CIs, 1990–2021, females.

## Data Availability

The datasets used and/or analyzed during the current study are available from the corresponding authors on reasonable request.
