# Supplementary material for: Lifetime Probability of Developing Migraine: A Novel Epidemiological Perspective Based on Global Burden of Disease Data
Source: Pain Res Manag. 2026 Aug 23;2026:9739777. doi: 10.1155/prm/9739777 (PMC13500892; doi:10.1155/prm/9739777)
Supplement: Supplementary file 1 [file PRM-2026-9739777-s001.docx]

Appendix A: Supplementary Methods

[**Methods for Estimating Lifetime Risk of Migraine**	1](#_Toc198629404)

[**Methods for Frontier Analysis**	4](#_Toc198629405)

**Methods for Estimating Lifetime Risk of Migraine**

The lifetime risk of migraine is an estimate of a person’s chances of being diagnosed with migraine at some point during their life. Lifetime risk estimates are usually expressed as the odds of developing migraine (‘1 in X’), as a percentage, or as the number of people diagnosed per 100 people.

Here we describe briefly introduction about the methods of lifetime risk of developing migraine as follows:

1. **Cumulative rate**

$$Cumulative rate=\sum_{i=1}^{A} W_{i}R_{i}$$

A denotes the age-band, usually use 74 for cumulate rate calculate.

W_i_ denotes the width of the i^th^ age interval

R_i_ denotes the age-specific incidence rate

2. **Cumulative risk**

The cumulative risk can be calculated by the formular:

$$Cumulate risk=1-e^{-cumulative rate}$$

3. **The “adjusted for multiple primaries (AMP)” method**

Using all-cause mortality and migraine incidence, mortality, we calculated the lifetime risk by five-year age groups:

$$S=\int_{0}^{\infty} \lambda_{c}\left( a \right)S_{0}\left( a \right)\mathrm{da}=\sum_{i=1}^{f} \frac{R_{i}}{R_{i}+M_{i}-D_{i}}\hat{S}_{0}^{*}\left( a_{i} \right)\times\left( 1-exp\left( -\frac{w_{i}}{N_{i}}\left( R_{i}+M_{i}-D_{i} \right) \right) \right)$$

where for age group i:

S denotes the probability of being diagnosed with migraine

Mi denotes the annual number of deaths (all-cause mortality);

Di denotes the annual number of migraine deaths (migraine mortality);

Ri denotes the annual number of migraine cases;

Ni denotes the population;

λc denotes the migraine incidence rate;

S ̂_0^* (a_i )denotes the probability of being alive and migraine free at age ai;

Wi denotes the width of the ith age group;

where the i^th^ interval is from a_i_ to a_i+1_,

w_i=_ (a_i+1_-a_i_)

$$\hat{S}_{0}^{*}\left( a_{i} \right)=exp (-\sum_{j=1}^{i-1} \frac{R_{j}{+ (M}_{j}-D_{j})}{N_{j}})$$

The “adjusted for multiple primaries (AMP)” method addresses the issues of multiple primary tumors by assuming that

(1) one cannot die of migraine if one has never had migraine;

(2) people with migraine have the same risk of developing migraine again as those who have never had migraine before;

and (3) the probability of dying from other causes (not migraine) is the same between migraine patients and those who have never had migraine.

In practice, migraine incidence and mortality data are usually presented in the form of frequency table for calculation, and the final age band is usually 85 years old or above (e.g.0-4,5-4,10-10, … , 80-84, 85+). For the final age band (e.g. 85+), the integral for the final age band evaluates to the following:

$$\frac{R_{f}}{R_{f}+M_{f}-D_{f}}S_{0}\left( a_{f} \right)$$

The derivation of variance to the following:

let： $\lambda_{c}=\frac{R_{i}}{R_{i}+M_{i}-D_{i}},\hat{S}_{0}^{*}=\hat{S}_{0}^{*}\left( a_{i} \right),S_{x}=\left( 1-\exp\left( -\frac{w_{i}}{N_{i}}\left( R_{i}+M_{i}-D_{i} \right) \right) \right)$ then

$Var\left( S \right)=\sum_{i=1}^{f} Var\left( \lambda_{c}\hat{S}_{0}^{*}S_{x} \right)$=$\sum_{i=1}^{f} \left[ E\left( \lambda_{c}^{2}\left( \hat{S}_{0}^{*} \right)^{2}S_{x}^{2} \right)-{E\left( \lambda_{c}\hat{S}_{0}^{*}S_{x} \right)}^{2} \right] (1)$

$$E\left( \lambda_{c}^{2}\left( \hat{S}_{0}^{*} \right)^{2}S_{x}^{2} \right)=E (\lambda_{c}^{2})E\left( \hat{S}_{0}^{*} \right)^{2}E\left( S_{x}^{2} \right)=\left( var(\hat{S}_{0}^{*})+{(E\hat{S}_{0}^{*})}^{2}][Var\left( S_{x} \right)+{E (S_{x})}^{2}]\} \right)$$

$$Var\left( S \right)=\sum_{i=1}^{f} \{[Var (\lambda_{c})+\left( E\lambda_{c} \right)^{2}][Var\left( \hat{S}_{0}^{*} \right)+\left( E\hat{S}_{0}^{*} \right)^{2}][Var\left( S_{x} \right)+\left( ES_{x} \right)^{2}-\left( E\lambda_{c} \right)^{2}\left( E\hat{S}_{0}^{*} \right)^{2}\left( ES_{x} \right)^{2}\}$$

Let:

$p_{ci}=\frac{R_{i}}{R_{i}+M_{i}-D_{i}},$ then

$$E\lambda_{c}=p_{ci}, \left( Binomial\& Poisson \right) (2)$$

$$Var\left( \lambda_{c} \right)=\left\{ \begin{aligned} \frac{p_{ci}\left( 1-p_{ci} \right)}{R_{i}+M_{i}-D_{i}}, \left( Binomial \right) \\ \frac{p_{ci}}{R_{i}+M_{i}-D_{i}}, \left( Poisson \right) \end{aligned} (3) \right.$$

$\log\left( \hat{S}_{0}^{*} \right)=-\sum_{j=1}^{i-1} \frac{R_{j}+M_{j}-D_{j}}{N_{j}}$, let $p_{oj}=\frac{R_{j}+M_{j}-D_{j}}{N_{j}},$ then

$$Var\left( log\hat{S}_{0}^{*} \right)=\sum_{j=1}^{i-1} Var\left( \frac{R_{j}+M_{j}-D_{j}}{N_{j}} \right)=\left\{ \begin{aligned} \sum_{j=1}^{i-1} \frac{p_{oj}\left( 1-p_{oj} \right)}{N_{j}}, (Binomial) \\ \sum_{j=1}^{i-1} \frac{p_{oj}}{N_{j}}, (Poisson) \end{aligned} \right.$$

According to delta method, if $\hat{\theta}\sim N\left( \theta,\sigma^{2} \right)$, then $f\left( \hat{\theta} \right)\sim N (f\left( \theta\right),{[f^{'}\left( \theta\right)]}^{2}\sigma^{2})$. Let $\hat{S}_{0}^{*}=e^{\hat{\theta}}, then$

$$E\left( \hat{S}_{0}^{*} \right)=\exp\left( -\sum_{j=1}^{i-1} p_{oj} \right), \left( Binomial \& Poisson \right) (4)$$

$$Var\left( \hat{S}_{0}^{*} \right)={(e^{\hat{\theta}})}^{2}Var\left( log\hat{S}_{0}^{*} \right)=\left( \hat{S}_{0}^{*} \right)^{2}Var\left( log\hat{S}_{0}^{*} \right)=\left\{ \begin{aligned} \left( \hat{S}_{0}^{*} \right)^{2}\times\sum_{j=1}^{i-1} \frac{p_{oj}\left( 1-p_{oj} \right)}{N_{j}}, \left( Binomial \right) \\ \left( \hat{S}_{0}^{*} \right)^{2}\times\sum_{j=1}^{i-1} \frac{p_{oj}}{N_{j}}, \left( Poisson \right) \end{aligned} \right. (5)$$

$S_{x}=\left( 1-\exp\left( -\frac{w_{i}}{N_{i}}\left( R_{i}+M_{i}-D_{i} \right) \right) \right)$,

Let $S_{x}^{'}=\exp\left( -\frac{w_{i}}{N_{i}}\left( R_{i}+M_{i}-D_{i} \right) \right),$ $\log\left( S_{x}^{'} \right)=-\frac{w_{i}}{N_{i}}\left( R_{i}+M_{i}-D_{i} \right)$, and $p_{xi}=\frac{R_{i}}{R_{i}+M_{i}-D_{i}}$，then

Var ($\log S_{x}^{'})=\left\{ \begin{aligned} {w_{i}}^{2}\frac{p_{xi}\left( 1-p_{xi} \right)}{N_{i}}, (Binomial) \\ {w_{i}}^{2}\frac{p_{xi}}{N_{i}}, (Poisson) \end{aligned} \right.$

Let $S_{x}=1-e^{\hat{\theta}},$ according to delta method，then：

$$E\left( S_{x} \right)=\left( 1-\exp\left( -\frac{w_{i}}{N_{i}}\left( R_{i}+M_{i}-D_{i} \right) \right) \right), \left( Binomial \& Poisson \right) (6)$$

$Var\left( S_{x} \right)={(e^{\hat{\theta}})}^{2}\times Var\left( e^{\hat{\theta}} \right)=\left\{ \begin{aligned} {(S_{x}^{'})}^{2}\times{w_{i}}^{2}\frac{p_{xi}\left( 1-p_{xi} \right)}{N_{i}}, (Binomial) \\ {(S_{x}^{'})}^{2}\times{w_{i}}^{2}\frac{p_{xi}}{N_{i}}, (Poisson) \end{aligned} \right.$ (7)

Reference:

**1.** **Sasieni P, Shelton J, Ormiston-Smith N, et al. What is the lifetime risk of developing cancer?: The effect of adjusting for multiple primaries. Brit J Cancer 2011;105 (3):460-5**

**2.Ahmad AS, Ormiston-Smith N, Sasieni PD. Trends in the lifetime risk of developing cancer in Great Britain: comparison of risk for those born from 1930 to 1960. Br J Cancer 2015;112:943-7.**

**3. Wang S, Zheng R, Li J, Zeng H, Li L, Chen R, et al. Global, regional, and national lifetime risks of developing and dying from gastrointestinal cancers in 185 countries: a population-based systematic analysis of GLOBOCAN. The Lancet Gastroenterology & Hepatology. 2024;9:229–37.**

**4. Zheng R, Wang S, Zhang S, Zeng H, Chen R, Sun K, et al. Global, regional, and national lifetime probabilities of developing cancer in 2020. Science Bulletin. 2023;68:2620–8.**

**Methods for Frontier Analysis**

To evaluate the relationship between disease burden and sociodemographic development, we employed frontier analysis as a quantitative approach to identify the minimum achievable lifetime risks of incidence and mortality based on development status measured by the Socio-demographic Index (SDI). The frontier analysis determined the lowest attainable lifetime risk levels for each country/territory according to its SDI. The distance to the frontier, termed the effective difference, represents potential unrealized improvement opportunities (reduction in disease lifetime risk) that should be achievable given a country's position on the development spectrum.

We utilized Data Envelopment Analysis (DEA) with the Free Disposal Hull (FDH) method to construct a nonlinear frontier, generating migraine frontiers stratified by SDI using data from 1990 to 2021. To account for uncertainty, we performed 1,000 bootstrap samples, randomly sampling (with replacement) from all countries and territories across all years. The average lifetime risk of migraine was calculated for each SDI value. Subsequently, we applied LOESS regression with a local polynomial order of 1 and span of 0.2 to generate smoothed frontier curves. Super-efficient countries were excluded from frontier generation to eliminate the influence of outliers.

**Reference:**

**1. Access GBDH, Quality Collaborators. Electronic address cue, Access GBDH, Quality C. Healthcare Access and Quality Index based on mortality from causes amenable to personal health care in 195 countries and territories, 1990-2015: a novel analysis from the Global Burden of Disease Study 2015. Lancet. May 18 2017;doi:10.1016/S0140-6736(17)30818-8**

**2. Xie Y, Bowe B, Xian H, Balasubramanian S, Al-Aly Z. Rate of Kidney Function Decline and Risk of Hospitalizations in Stage 3A CKD. Clinical journal of the American Society of Nephrology : CJASN. Nov 6 2015;10(11):1946-55. doi:10.2215/CJN.04480415**
