# Supplementary material for: Lifetime Probability of Developing Migraine: A Novel Epidemiological Perspective Based on Global Burden of Disease Data
Source: Pain Res Manag. 2026 Aug 23;2026:9739777. doi: 10.1155/prm/9739777 (PMC13500892; doi:10.1155/prm/9739777)
Supplement: Supplementary file 2 [file PRM-2026-9739777-s002.docx]

Appendix B: Supplementary Tables & Figures

[Table S1 Age-conditional probability (%) of developing migraine, both sexes, 2021.	2](#_Toc192321059)

[Table S2. Age-conditional probability (%) of developing migraine, males, 2021.	9](#_Toc192321060)

[Table S3. Age-conditional probability (%) of developing migraine, females, 2021.	16](#_Toc192321061)

[Table S4 Female-to-male ratios of migraine lifetime risk with 95% confidence intervals, 2021	25](#_Toc192321062)

[Table S5 Age-specific female-to-male ratios of migraine lifetime risk with 95% confidence intervals, 2021	30](#_Toc192321063)

[Table S6. Regional AAPC in migraine lifetime risk with 95% confidence intervals, 1990-2021, both sexes.	41](#_Toc192321064)

[Table S7. Regional AAPC in migraine lifetime risk with 95% confidence intervals, 1990-2021, males.	50](#_Toc192321065)

[Table S8. Regional AAPC in migraine lifetime risk with 95% confidence intervals, 1990-2021, females.	59](#_Toc192321066)

[Table S9. Projected global migraine lifetime risk (%) with 95% confidence intervals, 2020-2050.	68](#_Toc192321067)

[Table S10. Concentration index of migraine lifetime risk (%) with 95% confidence intervals, 1990-2021, both sexes.	69](#_Toc192321068)

[Table S11. Concentration index of migraine lifetime risk (%) with 95% confidence intervals, 1990-2021, males.	70](#_Toc192321069)

[Table S12. Concentration index of migraine lifetime risk (%) with 95% confidence intervals, 1990-2021, females.	71](#_Toc192321070)

[Figure S1. Lifetime and age-conditional probability of developing migraine in the 21 areas of the world for both sexes, 2021	72](#_Toc192321071)

[Figure S2. Lifetime and age-conditional probability of developing migraine in the 21 areas of the world for males, 2021	73](#_Toc192321072)

[Figure S3. Lifetime and age-conditional probability of developing migraine in the 21 areas of the world for females, 2021	74](#_Toc192321073)

[Figure S4 Female-to-male ratios of migraine lifetime risk by temporal and age patterns across SDI levels.	75](#_Toc192321074)

Table S1. Age-conditional probability (%) of developing migraine, both sexes, 2021.

| **Region** | Birth to death | 10 years to death | 20 years to death | 30 years to death | 40 years to death | 50 years to death | 60 years to death | 70 years to death | 80 years to death |
| --- | --- | --- | --- | --- | --- | --- | --- | --- | --- |
| **Global** | 51.53(51.51-51.55) | 44.06(44.04-44.08) | 28.15(28.13-28.16) | 18.29(18.28-18.31) | 10.32(10.31-10.33) | 5.21(5.21-5.22) | 2.22(2.22-2.22) | 0.99(0.99-0.99) | 0.30(0.30-0.31) |
| **Afghanistan** | 48.70(48.38-49.02) | 39.29(38.98-39.60) | 23.76(23.48-24.04) | 14.47(14.24-14.70) | 7.47(7.32-7.62) | 3.38(3.30-3.46) | 1.15(1.11-1.20) | 0.35(0.33-0.37) | 0.05(0.04-0.06) |
| **Albania** | 51.90(50.88-52.91) | 45.35(44.36-46.33) | 30.15(29.21-31.09) | 20.40(19.53-21.26) | 11.87(11.15-12.60) | 5.98(5.53-6.43) | 2.60(2.44-2.76) | 1.19(1.10-1.28) | 0.32(0.27-0.36) |
| **Algeria** | 54.58(54.34-54.83) | 44.79(44.55-45.03) | 28.46(28.23-28.68) | 18.23(18.03-18.42) | 10.23(10.06-10.39) | 5.32(5.21-5.43) | 2.43(2.38-2.48) | 1.10(1.07-1.13) | 0.28(0.26-0.29) |
| **American Samoa** | 53.17(45.15-61.19) | 46.17(38.31-54.03) | 29.58(22.08-37.08) | 19.10(12.92-25.29) | 10.81(6.44-15.19) | 5.44(3.01-7.88) | 2.17(1.08-3.26) | 0.91(0.20-1.62) | 0.24(-0.15-0.63) |
| **Andean Latin America** | 43.29(43.05-43.53) | 35.18(34.94-35.41) | 22.66(22.44-22.88) | 15.69(15.51-15.88) | 8.98(8.84-9.12) | 4.37(4.30-4.45) | 1.85(1.82-1.88) | 0.78(0.76-0.80) | 0.22(0.21-0.23) |
| **Andorra** | 59.81(55.73-63.89) | 50.20(46.19-54.20) | 29.41(25.60-33.22) | 18.19(14.83-21.54) | 9.79(6.89-12.69) | 4.54(2.37-6.71) | 2.02(1.06-2.98) | 1.13(0.59-1.68) | 0.50(0.12-0.88) |
| **Angola** | 44.49(44.15-44.83) | 38.52(38.19-38.86) | 24.59(24.28-24.90) | 15.63(15.38-15.88) | 8.49(8.31-8.66) | 3.91(3.82-4.00) | 1.36(1.31-1.40) | 0.49(0.46-0.52) | 0.09(0.08-0.10) |
| **Antigua and Barbuda** | 53.38(48.21-58.55) | 42.38(37.31-47.45) | 25.89(21.15-30.63) | 17.76(13.63-21.89) | 10.10(6.70-13.49) | 4.95(2.88-7.03) | 2.09(1.30-2.89) | 0.95(0.44-1.45) | 0.28(-0.02-0.57) |
| **Argentina** | 47.06(46.79-47.33) | 41.78(41.52-42.04) | 26.83(26.58-27.08) | 17.25(17.03-17.47) | 9.77(9.60-9.95) | 4.82(4.71-4.93) | 2.04(2.00-2.08) | 0.99(0.97-1.01) | 0.36(0.34-0.37) |
| **Armenia** | 52.16(51.20-53.11) | 45.56(44.63-46.49) | 30.08(29.20-30.96) | 20.10(19.33-20.87) | 11.54(10.88-12.21) | 5.80(5.39-6.21) | 2.46(2.32-2.60) | 1.11(1.02-1.19) | 0.31(0.27-0.35) |
| **Australasia** | 52.70(52.42-52.99) | 46.56(46.28-46.84) | 29.48(29.21-29.75) | 19.10(18.86-19.35) | 10.79(10.57-11.01) | 5.41(5.25-5.56) | 2.46(2.40-2.53) | 1.37(1.34-1.40) | 0.57(0.54-0.59) |
| **Australia** | 52.52(52.20-52.83) | 46.33(46.02-46.64) | 29.37(29.08-29.67) | 19.04(18.77-19.31) | 10.75(10.51-10.99) | 5.39(5.22-5.57) | 2.46(2.39-2.54) | 1.37(1.34-1.41) | 0.57(0.55-0.60) |
| **Austria** | 58.45(58.03-58.87) | 49.63(49.22-50.04) | 29.53(29.14-29.92) | 18.32(17.97-18.68) | 9.90(9.59-10.22) | 4.59(4.36-4.81) | 2.02(1.94-2.10) | 1.11(1.06-1.15) | 0.47(0.44-0.50) |
| **Azerbaijan** | 50.41(49.89-50.93) | 43.95(43.45-44.44) | 28.76(28.30-29.22) | 19.00(18.61-19.40) | 10.75(10.42-11.07) | 5.23(5.05-5.41) | 2.05(1.98-2.12) | 0.82(0.77-0.87) | 0.19(0.17-0.21) |
| **Bahamas** | 52.23(49.52-54.94) | 41.20(38.54-43.86) | 24.64(22.15-27.14) | 16.54(14.57-18.52) | 9.12(7.77-10.47) | 4.30(3.59-5.00) | 1.72(1.41-2.03) | 0.74(0.53-0.95) | 0.20(0.09-0.31) |
| **Bahrain** | 52.90(51.64-54.15) | 42.99(41.76-44.22) | 26.64(25.49-27.80) | 17.22(16.19-18.24) | 10.02(9.14-10.89) | 5.34(4.70-5.98) | 2.41(2.08-2.74) | 0.99(0.75-1.24) | 0.20(0.09-0.31) |
| **Bangladesh** | 53.92(53.79-54.05) | 45.35(45.23-45.48) | 29.13(29.02-29.25) | 19.02(18.92-19.12) | 10.67(10.59-10.76) | 5.29(5.23-5.34) | 2.25(2.23-2.27) | 0.96(0.95-0.97) | 0.25(0.25-0.26) |
| **Barbados** | 53.30(50.20-56.39) | 42.35(39.32-45.39) | 25.91(23.07-28.76) | 17.77(15.35-20.18) | 10.18(8.29-12.06) | 5.09(3.97-6.21) | 2.24(1.85-2.63) | 1.07(0.83-1.32) | 0.34(0.20-0.48) |
| **Belarus** | 48.69(48.09-49.29) | 42.60(42.02-43.19) | 28.17(27.63-28.72) | 18.56(18.13-18.99) | 10.40(10.09-10.71) | 5.17(5.01-5.32) | 2.10(2.05-2.16) | 0.85(0.81-0.88) | 0.22(0.20-0.23) |
| **Belgium** | 62.78(62.43-63.12) | 51.35(51.01-51.69) | 28.35(28.03-28.68) | 17.18(16.88-17.48) | 9.15(8.89-9.41) | 4.16(3.98-4.34) | 1.83(1.77-1.90) | 1.03(0.99-1.06) | 0.45(0.42-0.47) |
| **Belize** | 52.50(49.85-55.15) | 41.53(38.91-44.15) | 25.07(22.57-27.57) | 16.98(14.84-19.13) | 9.55(7.86-11.24) | 4.65(3.61-5.68) | 1.97(1.52-2.42) | 0.92(0.60-1.24) | 0.29(0.10-0.48) |
| **Benin** | 48.26(47.75-48.76) | 40.64(40.15-41.13) | 25.25(24.79-25.71) | 16.23(15.83-16.63) | 8.98(8.68-9.29) | 4.26(4.08-4.44) | 1.58(1.51-1.66) | 0.65(0.60-0.69) | 0.14(0.12-0.17) |
| **Bermuda** | 54.08(47.62-60.53) | 43.00(36.64-49.37) | 26.39(20.38-32.40) | 18.14(13.08-23.19) | 10.47(6.38-14.56) | 5.37(2.81-7.93) | 2.48(1.50-3.47) | 1.26(0.70-1.81) | 0.44(0.11-0.77) |
| **Bhutan** | 53.77(51.86-55.69) | 45.20(43.37-47.03) | 29.00(27.28-30.72) | 19.06(17.50-20.63) | 10.80(9.44-12.17) | 5.49(4.54-6.44) | 2.42(2.02-2.81) | 1.06(0.84-1.28) | 0.30(0.18-0.42) |
| **Bolivia (Plurinational State of)** | 42.79(42.24-43.34) | 34.53(33.99-35.07) | 21.88(21.38-22.37) | 14.94(14.53-15.35) | 8.30(8.02-8.59) | 3.82(3.68-3.95) | 1.43(1.38-1.49) | 0.50(0.47-0.53) | 0.10(0.09-0.11) |
| **Bosnia and Herzegovina** | 52.23(51.36-53.11) | 45.59(44.74-46.45) | 30.18(29.37-30.99) | 20.25(19.55-20.96) | 11.61(11.04-12.19) | 5.80(5.45-6.15) | 2.45(2.34-2.56) | 1.08(1.01-1.14) | 0.29(0.26-0.32) |
| **Botswana** | 44.45(43.34-45.56) | 38.41(37.36-39.45) | 24.27(23.35-25.20) | 15.20(14.53-15.87) | 8.06(7.65-8.47) | 3.61(3.39-3.83) | 1.19(1.09-1.30) | 0.41(0.35-0.47) | 0.07(0.05-0.09) |
| **Brazil** | 60.61(60.51-60.71) | 41.98(41.88-42.07) | 23.03(22.94-23.12) | 15.80(15.73-15.87) | 8.87(8.81-8.92) | 4.32(4.29-4.35) | 1.87(1.86-1.89) | 0.89(0.88-0.90) | 0.29(0.28-0.29) |
| **Brunei Darussalam** | 43.12(40.59-45.64) | 38.16(35.69-40.63) | 24.55(22.17-26.92) | 15.52(13.36-17.68) | 8.70(6.90-10.49) | 4.30(3.16-5.43) | 1.90(1.36-2.45) | 0.92(0.51-1.34) | 0.31(0.02-0.61) |
| **Bulgaria** | 50.98(50.27-51.69) | 44.35(43.67-45.04) | 28.98(28.35-29.62) | 19.15(18.66-19.64) | 10.64(10.29-10.99) | 5.01(4.85-5.18) | 1.89(1.84-1.95) | 0.73(0.70-0.75) | 0.16(0.14-0.17) |
| **Burkina Faso** | 46.83(46.45-47.21) | 39.37(39.00-39.73) | 24.32(23.98-24.66) | 15.48(15.19-15.77) | 8.45(8.23-8.66) | 3.93(3.82-4.05) | 1.39(1.34-1.43) | 0.53(0.50-0.56) | 0.11(0.09-0.12) |
| **Burundi** | 35.69(35.09-36.30) | 31.65(31.08-32.23) | 21.56(21.03-22.09) | 13.89(13.45-14.32) | 7.87(7.56-8.18) | 3.89(3.73-4.05) | 1.58(1.49-1.66) | 0.62(0.56-0.67) | 0.13(0.11-0.16) |
| **Cabo Verde** | 53.05(50.80-55.31) | 44.86(42.67-47.05) | 28.28(26.23-30.34) | 18.60(16.77-20.44) | 10.73(9.17-12.28) | 5.48(4.51-6.45) | 2.33(1.92-2.74) | 1.11(0.82-1.40) | 0.30(0.17-0.43) |
| **Cambodia** | 55.02(54.62-55.41) | 47.30(46.92-47.68) | 29.53(29.18-29.88) | 18.64(18.33-18.94) | 10.20(9.96-10.45) | 5.03(4.89-5.17) | 1.93(1.87-1.98) | 0.73(0.69-0.76) | 0.17(0.15-0.19) |
| **Cameroon** | 48.14(47.82-48.45) | 40.41(40.11-40.71) | 24.82(24.54-25.10) | 15.73(15.50-15.96) | 8.48(8.31-8.64) | 3.88(3.79-3.97) | 1.34(1.30-1.38) | 0.50(0.48-0.53) | 0.10(0.09-0.11) |
| **Canada** | 56.50(56.26-56.75) | 49.30(49.06-49.54) | 29.07(28.84-29.30) | 17.92(17.72-18.13) | 9.66(9.49-9.83) | 4.69(4.57-4.80) | 2.09(2.04-2.13) | 1.14(1.12-1.16) | 0.49(0.47-0.50) |
| **Caribbean** | 50.57(50.32-50.83) | 39.94(39.70-40.19) | 24.04(23.81-24.26) | 16.25(16.06-16.43) | 9.09(8.95-9.23) | 4.38(4.31-4.45) | 1.81(1.78-1.84) | 0.81(0.80-0.83) | 0.24(0.23-0.25) |
| **Central African Republic** | 39.51(38.78-40.25) | 33.96(33.28-34.64) | 21.06(20.47-21.64) | 12.89(12.46-13.31) | 6.54(6.31-6.78) | 2.71(2.60-2.83) | 0.78(0.73-0.84) | 0.23(0.20-0.26) | 0.03(0.02-0.04) |
| **Central Asia** | 50.79(50.61-50.96) | 44.22(44.05-44.40) | 28.81(28.65-28.98) | 19.01(18.87-19.15) | 10.73(10.62-10.84) | 5.23(5.17-5.30) | 2.09(2.06-2.12) | 0.86(0.85-0.88) | 0.21(0.20-0.22) |
| **Central Europe** | 52.49(52.34-52.64) | 45.94(45.79-46.09) | 30.31(30.17-30.45) | 20.28(20.16-20.40) | 11.57(11.47-11.66) | 5.73(5.68-5.79) | 2.41(2.39-2.43) | 1.06(1.05-1.07) | 0.30(0.29-0.30) |
| **Central Latin America** | 51.92(51.81-52.03) | 41.70(41.60-41.81) | 25.99(25.89-26.09) | 17.25(17.17-17.33) | 9.64(9.57-9.70) | 4.68(4.65-4.72) | 2.01(2.00-2.02) | 0.90(0.89-0.91) | 0.27(0.27-0.28) |
| **Central Sub-Saharan Africa** | 44.16(43.99-44.33) | 38.24(38.08-38.39) | 24.41(24.27-24.56) | 15.56(15.44-15.68) | 8.49(8.41-8.57) | 3.93(3.89-3.98) | 1.37(1.35-1.39) | 0.50(0.49-0.51) | 0.09(0.09-0.10) |
| **Chad** | 45.45(45.00-45.90) | 38.16(37.73-38.59) | 23.48(23.08-23.88) | 14.93(14.59-15.27) | 8.07(7.82-8.32) | 3.69(3.56-3.83) | 1.29(1.23-1.34) | 0.49(0.45-0.52) | 0.10(0.08-0.11) |
| **Chile** | 47.70(47.29-48.11) | 42.27(41.87-42.67) | 27.14(26.76-27.53) | 17.63(17.29-17.97) | 10.11(9.82-10.39) | 5.09(4.91-5.27) | 2.26(2.19-2.33) | 1.17(1.13-1.22) | 0.47(0.44-0.49) |
| **China** | 49.37(49.32-49.42) | 44.43(44.38-44.48) | 30.67(30.63-30.72) | 20.89(20.85-20.93) | 12.61(12.58-12.64) | 7.09(7.07-7.11) | 3.29(3.28-3.31) | 1.53(1.52-1.53) | 0.47(0.46-0.47) |
| **Colombia** | 52.85(52.59-53.10) | 41.84(41.59-42.09) | 26.17(25.94-26.40) | 17.61(17.41-17.81) | 10.10(9.94-10.27) | 5.08(4.98-5.18) | 2.32(2.28-2.36) | 1.11(1.09-1.14) | 0.37(0.36-0.39) |
| **Comoros** | 37.21(34.71-39.70) | 33.11(30.74-35.49) | 22.87(20.70-25.04) | 15.03(13.21-16.85) | 8.77(7.46-10.07) | 4.53(3.84-5.22) | 1.97(1.65-2.30) | 0.84(0.63-1.05) | 0.20(0.10-0.31) |
| **Congo** | 45.06(44.26-45.85) | 38.99(38.23-39.76) | 24.82(24.13-25.51) | 15.73(15.19-16.27) | 8.48(8.14-8.83) | 3.89(3.71-4.07) | 1.36(1.27-1.45) | 0.49(0.44-0.55) | 0.09(0.07-0.11) |
| **Cook Islands** | 54.91(42.07-67.75) | 47.88(35.31-60.45) | 31.04(18.93-43.16) | 20.32(9.68-30.95) | 11.73(3.45-20.01) | 6.11(1.21-11.00) | 2.62(0.61-4.64) | 1.22(0.04-2.40) | 0.38(-0.29-1.06) |
| **Costa Rica** | 52.93(52.15-53.72) | 42.19(41.42-42.97) | 26.58(25.85-27.31) | 17.89(17.27-18.52) | 10.26(9.74-10.77) | 5.19(4.87-5.51) | 2.43(2.29-2.57) | 1.21(1.12-1.30) | 0.44(0.38-0.50) |
| **Croatia** | 52.53(51.76-53.29) | 45.90(45.15-46.65) | 30.63(29.92-31.34) | 20.58(19.96-21.21) | 11.89(11.36-12.42) | 6.04(5.70-6.39) | 2.66(2.54-2.77) | 1.23(1.17-1.29) | 0.36(0.33-0.39) |
| **Cuba** | 53.08(52.59-53.58) | 42.08(41.60-42.57) | 25.55(25.09-26.00) | 17.42(17.03-17.80) | 9.88(9.58-10.17) | 4.81(4.67-4.96) | 2.00(1.94-2.05) | 0.89(0.85-0.92) | 0.25(0.24-0.27) |
| **Cyprus** | 59.93(58.92-60.95) | 50.43(49.42-51.44) | 29.66(28.68-30.64) | 18.41(17.52-19.30) | 9.96(9.13-10.79) | 4.54(3.90-5.19) | 1.95(1.70-2.20) | 1.05(0.93-1.18) | 0.42(0.34-0.51) |
| **Czechia** | 52.56(52.06-53.06) | 45.90(45.41-46.39) | 30.44(29.97-30.90) | 20.50(20.10-20.90) | 11.86(11.53-12.18) | 6.05(5.82-6.27) | 2.68(2.60-2.75) | 1.26(1.22-1.30) | 0.39(0.37-0.41) |
| **Côte d’Ivoire** | 48.20(47.86-48.54) | 40.53(40.20-40.85) | 25.07(24.77-25.37) | 16.02(15.77-16.28) | 8.76(8.57-8.94) | 4.14(4.03-4.25) | 1.53(1.48-1.58) | 0.62(0.58-0.65) | 0.14(0.12-0.15) |
| **Democratic People's Republic of Korea** | 48.42(48.05-48.78) | 42.75(42.40-43.10) | 28.85(28.53-29.17) | 19.25(18.97-19.52) | 11.37(11.16-11.58) | 6.21(6.09-6.32) | 2.74(2.68-2.79) | 1.21(1.17-1.24) | 0.37(0.35-0.39) |
| **Democratic Republic of the Congo** | 44.29(44.08-44.49) | 38.36(38.17-38.56) | 24.55(24.36-24.73) | 15.71(15.56-15.86) | 8.63(8.53-8.74) | 4.04(3.98-4.09) | 1.42(1.40-1.45) | 0.53(0.51-0.54) | 0.10(0.09-0.11) |
| **Denmark** | 57.19(56.66-57.72) | 48.95(48.43-49.47) | 29.51(29.01-30.01) | 18.36(17.89-18.83) | 9.97(9.56-10.38) | 4.63(4.33-4.93) | 2.03(1.92-2.13) | 1.10(1.04-1.15) | 0.46(0.42-0.50) |
| **Djibouti** | 36.44(34.59-38.29) | 32.35(30.60-34.10) | 22.18(20.63-23.72) | 14.41(13.21-15.62) | 8.14(7.34-8.95) | 4.02(3.58-4.46) | 1.66(1.41-1.91) | 0.66(0.49-0.84) | 0.14(0.05-0.23) |
| **Dominica** | 51.43(44.88-57.98) | 40.62(34.28-46.95) | 24.45(18.55-30.35) | 16.56(11.56-21.55) | 9.24(5.43-13.05) | 4.41(2.56-6.27) | 1.77(1.08-2.46) | 0.76(0.31-1.20) | 0.20(-0.03-0.42) |
| **Dominican Republic** | 51.87(51.35-52.39) | 41.07(40.56-41.58) | 24.86(24.38-25.33) | 16.90(16.49-17.31) | 9.63(9.30-9.96) | 4.79(4.59-4.99) | 2.09(2.01-2.18) | 1.01(0.95-1.06) | 0.34(0.30-0.37) |
| **East Asia** | 49.39(49.34-49.44) | 44.42(44.37-44.47) | 30.65(30.60-30.69) | 20.86(20.83-20.90) | 12.59(12.56-12.62) | 7.08(7.06-7.10) | 3.29(3.28-3.30) | 1.52(1.52-1.53) | 0.47(0.46-0.47) |
| **Eastern Europe** | 50.47(50.35-50.60) | 44.37(44.25-44.50) | 28.95(28.83-29.07) | 18.91(18.83-19.00) | 10.53(10.47-10.59) | 5.21(5.18-5.25) | 2.12(2.11-2.13) | 0.86(0.85-0.87) | 0.22(0.22-0.22) |
| **Eastern Sub-Saharan Africa** | 35.63(35.53-35.73) | 31.74(31.64-31.84) | 21.66(21.57-21.75) | 13.96(13.89-14.03) | 7.87(7.83-7.92) | 3.86(3.83-3.88) | 1.53(1.51-1.54) | 0.58(0.57-0.59) | 0.12(0.11-0.12) |
| **Ecuador** | 47.18(46.73-47.64) | 37.45(37.00-37.90) | 23.59(23.17-24.01) | 16.40(16.04-16.77) | 9.46(9.18-9.75) | 4.70(4.53-4.87) | 2.08(2.01-2.15) | 0.96(0.92-1.00) | 0.31(0.29-0.34) |
| **Egypt** | 54.93(54.77-55.08) | 42.99(42.84-43.14) | 26.13(25.98-26.27) | 16.38(16.26-16.51) | 8.95(8.85-9.05) | 4.35(4.29-4.40) | 1.72(1.70-1.74) | 0.59(0.58-0.60) | 0.09(0.08-0.09) |
| **El Salvador** | 52.28(51.55-53.00) | 41.59(40.87-42.31) | 26.07(25.41-26.73) | 17.39(16.83-17.95) | 9.75(9.33-10.16) | 4.71(4.47-4.95) | 2.06(1.96-2.16) | 0.95(0.89-1.01) | 0.31(0.27-0.34) |
| **Equatorial Guinea** | 44.09(42.55-45.63) | 38.20(36.74-39.67) | 24.53(23.19-25.86) | 15.75(14.65-16.85) | 8.65(7.86-9.44) | 4.05(3.58-4.51) | 1.45(1.22-1.68) | 0.55(0.41-0.70) | 0.11(0.05-0.17) |
| **Eritrea** | 35.98(35.17-36.79) | 31.89(31.12-32.66) | 21.69(21.00-22.37) | 13.96(13.42-14.50) | 7.86(7.51-8.21) | 3.83(3.64-4.01) | 1.49(1.38-1.59) | 0.55(0.48-0.62) | 0.11(0.08-0.14) |
| **Estonia** | 49.63(48.04-51.23) | 43.53(41.96-45.11) | 29.06(27.60-30.53) | 19.44(18.20-20.69) | 11.30(10.29-12.31) | 5.98(5.38-6.59) | 2.73(2.52-2.94) | 1.28(1.17-1.40) | 0.43(0.37-0.49) |
| **Eswatini** | 41.91(40.37-43.44) | 35.91(34.49-37.32) | 21.93(20.77-23.09) | 13.08(12.32-13.85) | 6.38(5.96-6.80) | 2.52(2.30-2.73) | 0.65(0.56-0.74) | 0.16(0.12-0.20) | 0.02(0.01-0.03) |
| **Ethiopia** | 34.76(34.54-34.98) | 31.43(31.22-31.64) | 21.83(21.64-22.03) | 14.42(14.26-14.59) | 8.43(8.31-8.55) | 4.31(4.25-4.38) | 1.82(1.79-1.85) | 0.74(0.72-0.76) | 0.16(0.15-0.17) |
| **Fiji** | 51.93(50.13-53.74) | 45.00(43.27-46.73) | 28.53(26.94-30.11) | 18.22(16.91-19.52) | 10.04(9.12-10.97) | 4.79(4.33-5.24) | 1.67(1.48-1.85) | 0.58(0.47-0.70) | 0.11(0.06-0.17) |
| **Finland** | 59.72(59.15-60.30) | 50.18(49.61-50.75) | 29.33(28.79-29.87) | 18.11(17.62-18.59) | 9.78(9.36-10.20) | 4.55(4.26-4.85) | 2.02(1.92-2.13) | 1.13(1.08-1.18) | 0.50(0.46-0.53) |
| **France** | 59.64(59.48-59.80) | 50.29(50.13-50.44) | 29.86(29.71-30.02) | 18.48(18.34-18.61) | 9.95(9.83-10.07) | 4.60(4.52-4.68) | 2.06(2.03-2.09) | 1.17(1.15-1.18) | 0.52(0.51-0.54) |
| **Gabon** | 46.12(44.73-47.52) | 39.99(38.65-41.33) | 25.59(24.36-26.82) | 16.32(15.31-17.33) | 8.93(8.25-9.62) | 4.18(3.84-4.52) | 1.49(1.34-1.65) | 0.57(0.47-0.67) | 0.11(0.07-0.15) |
| **Gambia** | 49.69(48.52-50.85) | 41.76(40.63-42.89) | 25.73(24.68-26.79) | 16.36(15.46-17.26) | 8.86(8.20-9.51) | 4.08(3.72-4.45) | 1.44(1.29-1.60) | 0.56(0.46-0.65) | 0.11(0.07-0.14) |
| **Georgia** | 51.36(50.42-52.30) | 44.73(43.80-45.65) | 29.21(28.36-30.06) | 19.34(18.64-20.04) | 10.91(10.37-11.44) | 5.29(5.01-5.57) | 2.09(2.00-2.18) | 0.85(0.80-0.91) | 0.20(0.18-0.22) |
| **Germany** | 60.91(60.78-61.04) | 50.64(50.51-50.76) | 28.96(28.84-29.08) | 17.79(17.68-17.90) | 9.48(9.38-9.58) | 4.31(4.24-4.37) | 1.87(1.85-1.89) | 1.02(1.01-1.03) | 0.43(0.42-0.44) |
| **Ghana** | 50.18(49.88-50.49) | 42.26(41.97-42.55) | 26.26(25.99-26.53) | 16.84(16.61-17.07) | 9.25(9.09-9.42) | 4.37(4.27-4.47) | 1.59(1.55-1.63) | 0.64(0.61-0.66) | 0.13(0.12-0.15) |
| **Greece** | 60.35(59.96-60.75) | 50.41(50.02-50.80) | 29.25(28.88-29.62) | 17.97(17.65-18.30) | 9.63(9.36-9.90) | 4.36(4.18-4.55) | 1.85(1.79-1.91) | 0.99(0.95-1.02) | 0.40(0.39-0.42) |
| **Greenland** | 55.01(48.25-61.78) | 47.79(41.22-54.36) | 27.56(21.42-33.70) | 16.51(11.33-21.70) | 8.51(4.49-12.53) | 3.88(2.03-5.73) | 1.53(0.71-2.35) | 0.75(0.10-1.40) | 0.28(-0.20-0.76) |
| **Grenada** | 51.89(46.72-57.06) | 40.96(35.90-46.02) | 24.62(19.87-29.38) | 16.62(12.49-20.76) | 9.22(6.27-12.17) | 4.32(2.93-5.71) | 1.67(1.11-2.22) | 0.67(0.33-1.02) | 0.16(-0.02-0.35) |
| **Guam** | 53.95(49.59-58.30) | 46.97(42.66-51.28) | 30.34(26.27-34.41) | 19.95(16.40-23.50) | 11.62(8.93-14.30) | 6.14(4.57-7.71) | 2.74(2.02-3.46) | 1.38(0.88-1.88) | 0.53(0.19-0.88) |
| **Guatemala** | 51.04(50.59-51.48) | 40.45(40.01-40.89) | 25.10(24.69-25.51) | 16.58(16.22-16.93) | 9.15(8.89-9.42) | 4.33(4.18-4.48) | 1.80(1.74-1.86) | 0.77(0.73-0.81) | 0.21(0.19-0.23) |
| **Guinea** | 47.34(46.84-47.83) | 39.80(39.33-40.28) | 24.59(24.15-25.03) | 15.64(15.26-16.01) | 8.45(8.18-8.73) | 3.86(3.71-4.00) | 1.33(1.27-1.39) | 0.50(0.46-0.53) | 0.09(0.08-0.11) |
| **Guinea-Bissau** | 47.68(46.47-48.88) | 39.91(38.76-41.07) | 24.27(23.22-25.31) | 15.18(14.35-16.01) | 7.96(7.40-8.52) | 3.45(3.16-3.75) | 1.08(0.94-1.21) | 0.36(0.28-0.44) | 0.06(0.03-0.09) |
| **Guyana** | 50.54(48.60-52.48) | 39.69(37.79-41.58) | 23.44(21.71-25.18) | 15.51(14.11-16.91) | 8.28(7.40-9.17) | 3.67(3.27-4.08) | 1.30(1.13-1.48) | 0.48(0.37-0.59) | 0.10(0.05-0.16) |
| **Haiti** | 47.55(47.08-48.01) | 37.27(36.82-37.71) | 21.95(21.55-22.35) | 14.50(14.19-14.81) | 7.70(7.49-7.91) | 3.35(3.24-3.45) | 1.13(1.08-1.18) | 0.38(0.35-0.41) | 0.07(0.06-0.08) |
| **High-income Asia Pacific** | 45.76(45.63-45.89) | 40.75(40.63-40.88) | 26.39(26.27-26.51) | 16.89(16.78-17.00) | 9.72(9.63-9.82) | 5.05(4.99-5.12) | 2.49(2.47-2.52) | 1.39(1.38-1.40) | 0.61(0.60-0.61) |
| **High-income North America** | 57.81(57.72-57.89) | 50.72(50.64-50.80) | 29.54(29.47-29.62) | 17.91(17.85-17.98) | 9.46(9.41-9.51) | 4.44(4.41-4.47) | 1.86(1.85-1.87) | 0.95(0.94-0.95) | 0.37(0.36-0.37) |
| **Honduras** | 51.52(50.98-52.06) | 40.86(40.33-41.39) | 25.39(24.89-25.89) | 16.73(16.29-17.16) | 9.11(8.78-9.44) | 4.17(3.99-4.34) | 1.61(1.54-1.68) | 0.60(0.56-0.64) | 0.13(0.11-0.15) |
| **Hungary** | 52.08(51.56-52.61) | 45.44(44.92-45.95) | 30.01(29.52-30.49) | 20.09(19.68-20.50) | 11.43(11.12-11.74) | 5.64(5.46-5.82) | 2.34(2.29-2.40) | 1.04(1.00-1.07) | 0.30(0.28-0.32) |
| **Iceland** | 59.82(57.60-62.03) | 50.26(48.05-52.47) | 29.41(27.28-31.54) | 18.18(16.23-20.12) | 9.88(8.15-11.62) | 4.65(3.35-5.94) | 2.10(1.52-2.67) | 1.19(0.90-1.48) | 0.53(0.33-0.73) |
| **India** | 53.65(53.61-53.69) | 45.68(45.64-45.72) | 29.07(29.03-29.11) | 18.77(18.74-18.80) | 10.36(10.34-10.39) | 5.00(4.98-5.01) | 1.98(1.98-1.99) | 0.76(0.76-0.77) | 0.18(0.18-0.18) |
| **Indonesia** | 57.00(56.91-57.09) | 49.54(49.46-49.63) | 30.82(30.74-30.90) | 19.45(19.38-19.52) | 10.70(10.65-10.76) | 5.28(5.25-5.31) | 2.02(2.00-2.03) | 0.77(0.76-0.77) | 0.18(0.17-0.18) |
| **Iran (Islamic Republic of)** | 57.51(57.34-57.69) | 47.80(47.63-47.96) | 30.26(30.11-30.42) | 19.44(19.31-19.57) | 10.93(10.82-11.04) | 5.66(5.59-5.74) | 2.53(2.50-2.57) | 1.14(1.12-1.16) | 0.29(0.28-0.30) |
| **Iraq** | 53.60(53.34-53.85) | 43.83(43.58-44.08) | 27.55(27.32-27.79) | 17.47(17.26-17.68) | 9.61(9.44-9.78) | 4.83(4.73-4.93) | 2.05(2.00-2.09) | 0.83(0.80-0.86) | 0.19(0.17-0.20) |
| **Ireland** | 60.03(59.48-60.58) | 50.49(49.95-51.04) | 29.64(29.11-30.17) | 18.34(17.85-18.83) | 9.87(9.44-10.31) | 4.58(4.24-4.91) | 2.03(1.89-2.17) | 1.13(1.06-1.20) | 0.49(0.44-0.54) |
| **Israel** | 59.98(59.55-60.41) | 50.45(50.02-50.87) | 29.61(29.20-30.02) | 18.33(17.95-18.72) | 9.94(9.59-10.29) | 4.65(4.37-4.93) | 2.08(1.97-2.20) | 1.17(1.11-1.23) | 0.51(0.47-0.55) |
| **Italy** | 63.14(63.00-63.28) | 53.10(52.96-53.24) | 30.28(30.15-30.42) | 18.76(18.63-18.88) | 10.04(9.93-10.15) | 4.63(4.54-4.71) | 2.02(1.99-2.05) | 1.12(1.10-1.13) | 0.47(0.46-0.48) |
| **Jamaica** | 52.76(51.78-53.73) | 41.81(40.86-42.77) | 25.41(24.50-26.32) | 17.33(16.53-18.14) | 9.78(9.14-10.42) | 4.78(4.41-5.15) | 2.05(1.90-2.19) | 0.95(0.85-1.04) | 0.29(0.24-0.35) |
| **Japan** | 45.30(45.14-45.46) | 40.61(40.46-40.77) | 26.53(26.38-26.68) | 17.05(16.91-17.18) | 9.82(9.71-9.94) | 5.13(5.05-5.22) | 2.54(2.51-2.58) | 1.42(1.40-1.43) | 0.62(0.61-0.63) |
| **Jordan** | 54.53(54.07-54.99) | 44.69(44.24-45.14) | 28.31(27.87-28.74) | 18.27(17.87-18.68) | 10.45(10.09-10.80) | 5.55(5.31-5.80) | 2.61(2.49-2.74) | 1.23(1.14-1.31) | 0.35(0.30-0.40) |
| **Kazakhstan** | 51.10(50.70-51.50) | 44.45(44.06-44.84) | 28.81(28.45-29.18) | 18.86(18.56-19.16) | 10.49(10.26-10.71) | 4.98(4.86-5.10) | 1.91(1.86-1.96) | 0.75(0.72-0.78) | 0.16(0.15-0.18) |
| **Kenya** | 38.08(37.79-38.38) | 34.12(33.84-34.40) | 23.27(23.01-23.52) | 14.97(14.76-15.18) | 8.42(8.28-8.56) | 4.18(4.10-4.26) | 1.68(1.64-1.72) | 0.66(0.63-0.68) | 0.14(0.12-0.15) |
| **Kiribati** | 50.69(45.56-55.83) | 43.90(38.98-48.81) | 27.72(23.24-32.20) | 17.62(13.97-21.26) | 9.67(7.08-12.26) | 4.61(3.21-6.00) | 1.66(1.03-2.29) | 0.62(0.19-1.04) | 0.13(-0.08-0.34) |
| **Kuwait** | 56.05(55.34-56.77) | 46.05(45.35-46.75) | 29.50(28.84-30.16) | 19.13(18.52-19.75) | 10.98(10.40-11.56) | 6.06(5.57-6.56) | 3.07(2.76-3.38) | 1.57(1.37-1.77) | 0.53(0.42-0.65) |
| **Kyrgyzstan** | 51.26(50.58-51.94) | 44.65(43.98-45.31) | 29.10(28.46-29.74) | 19.23(18.66-19.80) | 10.90(10.45-11.36) | 5.38(5.11-5.66) | 2.23(2.11-2.35) | 0.98(0.89-1.06) | 0.27(0.23-0.31) |
| **Lao People's Democratic Republic** | 54.31(53.70-54.91) | 46.67(46.09-47.24) | 29.06(28.53-29.60) | 18.30(17.84-18.77) | 10.01(9.63-10.39) | 4.94(4.72-5.17) | 1.89(1.79-1.98) | 0.72(0.66-0.78) | 0.18(0.15-0.21) |
| **Latvia** | 49.04(47.69-50.40) | 42.97(41.63-44.30) | 28.51(27.23-29.78) | 18.88(17.86-19.91) | 10.73(9.98-11.47) | 5.46(5.09-5.83) | 2.35(2.22-2.48) | 1.03(0.95-1.11) | 0.31(0.27-0.35) |
| **Lebanon** | 55.18(54.51-55.85) | 45.32(44.66-45.98) | 28.91(28.28-29.53) | 18.72(18.16-19.28) | 10.60(10.11-11.08) | 5.52(5.19-5.84) | 2.51(2.39-2.64) | 1.15(1.09-1.22) | 0.31(0.28-0.34) |
| **Lesotho** | 39.59(38.44-40.74) | 33.78(32.75-34.82) | 20.32(19.50-21.14) | 11.88(11.37-12.40) | 5.63(5.37-5.90) | 2.11(1.97-2.24) | 0.50(0.45-0.55) | 0.11(0.09-0.13) | 0.01(0.01-0.01) |
| **Liberia** | 48.43(47.67-49.20) | 40.71(39.98-41.44) | 25.15(24.47-25.83) | 16.07(15.50-16.64) | 8.79(8.37-9.21) | 4.15(3.89-4.41) | 1.52(1.40-1.63) | 0.61(0.53-0.68) | 0.13(0.09-0.16) |
| **Libya** | 53.54(52.93-54.15) | 43.79(43.21-44.38) | 27.56(27.02-28.09) | 17.43(16.98-17.88) | 9.65(9.30-10.00) | 4.92(4.69-5.14) | 2.15(2.04-2.26) | 0.93(0.86-1.00) | 0.23(0.20-0.27) |
| **Lithuania** | 48.16(47.01-49.31) | 42.42(41.30-43.54) | 28.42(27.37-29.47) | 18.93(18.04-19.83) | 10.83(10.18-11.48) | 5.60(5.27-5.92) | 2.46(2.35-2.58) | 1.10(1.04-1.17) | 0.34(0.31-0.38) |
| **Luxembourg** | 59.02(57.56-60.48) | 50.01(48.58-51.45) | 29.61(28.24-30.98) | 18.37(17.09-19.64) | 9.93(8.75-11.10) | 4.62(3.73-5.51) | 2.06(1.70-2.43) | 1.15(0.95-1.36) | 0.50(0.37-0.64) |
| **Madagascar** | 36.10(35.70-36.51) | 32.04(31.66-32.43) | 21.87(21.52-22.23) | 14.12(13.83-14.41) | 7.98(7.79-8.18) | 3.90(3.80-4.00) | 1.54(1.48-1.59) | 0.58(0.54-0.61) | 0.11(0.10-0.13) |
| **Malawi** | 35.38(34.92-35.84) | 31.28(30.85-31.72) | 21.06(20.67-21.45) | 13.28(12.98-13.58) | 7.18(7.00-7.37) | 3.32(3.22-3.41) | 1.20(1.15-1.24) | 0.40(0.37-0.43) | 0.07(0.06-0.08) |
| **Malaysia** | 54.99(54.71-55.28) | 47.90(47.62-48.18) | 30.63(30.37-30.88) | 19.56(19.32-19.79) | 10.92(10.73-11.12) | 5.55(5.43-5.66) | 2.26(2.21-2.31) | 0.93(0.90-0.96) | 0.26(0.24-0.28) |
| **Maldives** | 56.58(54.34-58.82) | 48.72(46.56-50.88) | 30.61(28.55-32.68) | 19.91(17.97-21.86) | 11.72(9.89-13.56) | 6.42(4.90-7.95) | 2.96(2.10-3.82) | 1.42(0.95-1.90) | 0.49(0.22-0.76) |
| **Mali** | 46.33(45.96-46.71) | 38.92(38.55-39.28) | 23.98(23.64-24.32) | 15.25(14.96-15.54) | 8.23(8.02-8.43) | 3.77(3.66-3.87) | 1.30(1.25-1.34) | 0.48(0.45-0.51) | 0.09(0.08-0.10) |
| **Malta** | 59.58(57.66-61.51) | 50.11(48.25-51.97) | 29.38(27.65-31.12) | 18.17(16.53-19.80) | 9.83(8.33-11.33) | 4.59(3.48-5.70) | 2.05(1.66-2.43) | 1.14(0.95-1.34) | 0.50(0.36-0.64) |
| **Marshall Islands** | 51.30(43.98-58.61) | 44.37(37.30-51.43) | 27.89(21.39-34.39) | 17.66(12.50-22.82) | 9.68(6.13-13.22) | 4.59(2.64-6.53) | 1.61(0.69-2.54) | 0.58(-0.06-1.23) | 0.13(-0.25-0.50) |
| **Mauritania** | 51.73(50.82-52.64) | 43.70(42.81-44.59) | 27.45(26.60-28.30) | 17.87(17.11-18.64) | 10.10(9.48-10.72) | 4.96(4.58-5.33) | 1.95(1.80-2.09) | 0.85(0.76-0.95) | 0.20(0.15-0.25) |
| **Mauritius** | 56.77(55.37-58.16) | 48.87(47.53-50.22) | 30.65(29.39-31.91) | 19.47(18.40-20.55) | 10.84(10.03-11.65) | 5.55(5.07-6.02) | 2.32(2.13-2.50) | 1.02(0.90-1.14) | 0.32(0.24-0.39) |
| **Mexico** | 51.84(51.69-51.99) | 42.16(42.01-42.30) | 26.25(26.12-26.39) | 17.36(17.25-17.48) | 9.63(9.55-9.71) | 4.64(4.60-4.69) | 1.95(1.93-1.97) | 0.85(0.84-0.86) | 0.25(0.24-0.26) |
| **Micronesia (Federated States of)** | 52.00(46.49-57.51) | 45.04(39.70-50.39) | 28.51(23.51-33.51) | 18.20(14.06-22.34) | 10.08(7.22-12.94) | 4.89(3.39-6.40) | 1.81(1.11-2.51) | 0.71(0.20-1.21) | 0.16(-0.09-0.42) |
| **Monaco** | 59.52(52.04-67.00) | 49.93(42.63-57.22) | 29.13(22.18-36.07) | 17.89(11.90-23.89) | 9.52(4.81-14.22) | 4.23(1.48-6.98) | 1.78(0.85-2.70) | 0.93(0.44-1.42) | 0.37(0.05-0.68) |
| **Mongolia** | 50.80(49.85-51.76) | 44.19(43.25-45.12) | 28.66(27.79-29.52) | 18.80(18.07-19.52) | 10.53(9.97-11.08) | 5.08(4.76-5.40) | 2.02(1.86-2.17) | 0.84(0.73-0.95) | 0.21(0.15-0.26) |
| **Montenegro** | 51.86(49.78-53.93) | 45.23(43.18-47.28) | 29.86(27.94-31.77) | 19.96(18.28-21.65) | 11.30(9.96-12.65) | 5.48(4.70-6.26) | 2.18(1.94-2.41) | 0.86(0.73-1.00) | 0.17(0.12-0.22) |
| **Morocco** | 54.55(54.30-54.80) | 44.74(44.49-44.98) | 28.36(28.13-28.60) | 18.07(17.87-18.28) | 10.03(9.86-10.20) | 5.10(5.00-5.20) | 2.22(2.18-2.26) | 0.95(0.92-0.97) | 0.23(0.22-0.24) |
| **Mozambique** | 34.37(34.02-34.72) | 30.33(29.99-30.66) | 20.24(19.95-20.53) | 12.59(12.37-12.81) | 6.76(6.63-6.89) | 3.09(3.02-3.17) | 1.10(1.06-1.13) | 0.36(0.34-0.38) | 0.06(0.05-0.07) |
| **Myanmar** | 54.53(54.31-54.75) | 46.87(46.67-47.08) | 29.22(29.03-29.41) | 18.40(18.24-18.57) | 10.06(9.93-10.18) | 4.97(4.90-5.04) | 1.90(1.87-1.93) | 0.73(0.71-0.74) | 0.18(0.17-0.19) |
| **Namibia** | 44.92(43.77-46.06) | 38.81(37.72-39.90) | 24.55(23.58-25.52) | 15.43(14.68-16.17) | 8.19(7.73-8.65) | 3.64(3.41-3.87) | 1.18(1.07-1.28) | 0.39(0.33-0.45) | 0.06(0.04-0.08) |
| **Nauru** | 50.44(33.60-67.29) | 43.58(27.30-59.85) | 27.26(12.38-42.13) | 17.11(5.28-28.93) | 9.17(1.17-17.17) | 4.22(-0.08-8.53) | 1.42(-0.52-3.36) | 0.49(-0.66-1.64) | 0.09(-0.47-0.65) |
| **Nepal** | 54.63(54.34-54.92) | 45.48(45.20-45.76) | 28.81(28.54-29.07) | 18.49(18.25-18.72) | 10.07(9.88-10.25) | 4.73(4.62-4.83) | 1.83(1.79-1.86) | 0.67(0.65-0.69) | 0.14(0.13-0.16) |
| **Netherlands** | 59.36(59.06-59.65) | 50.11(49.82-50.40) | 29.46(29.18-29.75) | 18.27(18.01-18.53) | 9.85(9.62-10.08) | 4.53(4.37-4.70) | 1.98(1.92-2.05) | 1.08(1.05-1.11) | 0.45(0.43-0.47) |
| **New Zealand** | 53.62(52.93-54.30) | 47.69(47.01-48.37) | 30.00(29.35-30.64) | 19.44(18.85-20.03) | 10.98(10.45-11.50) | 5.48(5.10-5.85) | 2.48(2.31-2.64) | 1.35(1.27-1.44) | 0.54(0.48-0.59) |
| **Nicaragua** | 52.04(51.38-52.69) | 41.38(40.73-42.03) | 25.91(25.29-26.53) | 17.34(16.79-17.89) | 9.80(9.36-10.25) | 4.79(4.52-5.06) | 2.08(1.96-2.19) | 0.94(0.86-1.01) | 0.29(0.25-0.34) |
| **Niger** | 47.41(47.01-47.81) | 39.90(39.51-40.29) | 24.74(24.37-25.11) | 15.91(15.59-16.23) | 8.83(8.58-9.08) | 4.17(4.03-4.31) | 1.52(1.46-1.57) | 0.61(0.57-0.65) | 0.13(0.11-0.15) |
| **Nigeria** | 48.96(48.84-49.08) | 41.82(41.70-41.93) | 25.88(25.77-25.98) | 16.65(16.56-16.75) | 9.28(9.20-9.35) | 4.51(4.47-4.56) | 1.74(1.72-1.76) | 0.73(0.72-0.75) | 0.17(0.16-0.17) |
| **Niue** | 50.44(-0.91-101.79) | 43.72(-3.34-90.79) | 28.09(-14.08-70.26) | 18.20(-16.77-53.17) | 10.31(-15.85-36.47) | 5.19(-9.65-20.02) | 2.05(-3.71-7.82) | 0.85(-2.22-3.93) | 0.21(-1.12-1.55) |
| **North Africa and Middle East** | 54.11(54.04-54.18) | 43.98(43.92-44.05) | 27.60(27.54-27.66) | 17.57(17.52-17.63) | 9.79(9.75-9.84) | 5.01(4.98-5.03) | 2.19(2.18-2.20) | 0.93(0.92-0.94) | 0.22(0.22-0.23) |
| **North Macedonia** | 51.43(50.35-52.50) | 44.80(43.75-45.85) | 29.42(28.45-30.39) | 19.56(18.71-20.41) | 10.99(10.33-11.65) | 5.27(4.91-5.64) | 2.05(1.93-2.16) | 0.79(0.72-0.85) | 0.15(0.12-0.18) |
| **Northern Mariana Islands** | 53.48(45.67-61.28) | 46.48(38.83-54.13) | 29.78(22.54-37.01) | 19.30(13.32-25.27) | 10.94(6.61-15.27) | 5.51(3.18-7.83) | 2.21(1.10-3.32) | 0.93(0.14-1.72) | 0.25(-0.20-0.69) |
| **Norway** | 61.47(60.96-61.98) | 51.95(51.45-52.46) | 29.66(29.17-30.14) | 18.46(18.02-18.91) | 10.01(9.60-10.41) | 4.71(4.40-5.02) | 2.10(1.97-2.24) | 1.17(1.10-1.23) | 0.50(0.45-0.54) |
| **Oceania** | 49.97(49.49-50.45) | 43.29(42.83-43.75) | 27.45(27.03-27.87) | 17.53(17.18-17.88) | 9.65(9.39-9.90) | 4.61(4.47-4.75) | 1.63(1.57-1.70) | 0.59(0.55-0.63) | 0.12(0.10-0.14) |
| **Oman** | 52.76(51.99-53.52) | 42.88(42.12-43.63) | 26.39(25.69-27.09) | 16.99(16.36-17.61) | 9.86(9.31-10.42) | 5.19(4.78-5.59) | 2.34(2.14-2.54) | 0.99(0.86-1.12) | 0.24(0.17-0.31) |
| **Pakistan** | 51.30(51.19-51.42) | 43.68(43.57-43.79) | 27.69(27.59-27.79) | 17.76(17.68-17.85) | 9.67(9.61-9.74) | 4.55(4.52-4.59) | 1.74(1.73-1.76) | 0.63(0.62-0.64) | 0.13(0.13-0.14) |
| **Palau** | 51.52(37.07-65.96) | 44.57(30.62-58.53) | 28.04(15.48-40.60) | 18.10(8.75-27.45) | 10.34(4.29-16.38) | 5.24(1.86-8.61) | 2.06(0.54-3.58) | 0.83(-0.20-1.85) | 0.20(-0.41-0.81) |
| **Palestine** | 54.86(54.10-55.62) | 45.01(44.26-45.76) | 28.58(27.86-29.30) | 18.33(17.66-19.00) | 10.28(9.69-10.86) | 5.31(4.91-5.70) | 2.37(2.20-2.55) | 1.04(0.92-1.15) | 0.26(0.19-0.32) |
| **Panama** | 52.34(51.47-53.20) | 41.68(40.82-42.54) | 26.21(25.40-27.03) | 17.70(16.99-18.41) | 10.25(9.66-10.83) | 5.26(4.87-5.65) | 2.50(2.33-2.68) | 1.26(1.16-1.37) | 0.46(0.40-0.53) |
| **Papua New Guinea** | 49.50(48.94-50.05) | 42.87(42.34-43.39) | 27.16(26.68-27.64) | 17.32(16.92-17.73) | 9.50(9.20-9.80) | 4.51(4.34-4.67) | 1.57(1.49-1.64) | 0.54(0.49-0.59) | 0.10(0.08-0.13) |
| **Paraguay** | 58.62(58.07-59.17) | 40.34(39.80-40.89) | 22.33(21.82-22.84) | 15.28(14.84-15.72) | 8.53(8.18-8.89) | 4.09(3.88-4.30) | 1.69(1.61-1.77) | 0.75(0.70-0.81) | 0.21(0.18-0.24) |
| **Peru** | 41.37(41.04-41.71) | 34.20(33.88-34.53) | 22.51(22.21-22.81) | 15.64(15.39-15.90) | 9.00(8.81-9.19) | 4.42(4.32-4.52) | 1.89(1.85-1.94) | 0.80(0.77-0.83) | 0.23(0.21-0.24) |
| **Philippines** | 56.69(56.54-56.84) | 49.24(49.09-49.38) | 30.46(30.33-30.60) | 19.06(18.95-19.18) | 10.38(10.30-10.47) | 5.07(5.02-5.12) | 1.92(1.90-1.94) | 0.73(0.72-0.74) | 0.18(0.17-0.18) |
| **Poland** | 53.67(53.40-53.93) | 47.26(47.00-47.52) | 31.10(30.85-31.34) | 20.83(20.62-21.03) | 11.93(11.77-12.10) | 5.98(5.87-6.08) | 2.57(2.53-2.60) | 1.17(1.15-1.19) | 0.35(0.34-0.36) |
| **Portugal** | 59.96(59.58-60.35) | 50.42(50.05-50.80) | 29.59(29.23-29.94) | 18.29(17.97-18.62) | 9.85(9.58-10.13) | 4.53(4.34-4.73) | 1.98(1.91-2.05) | 1.09(1.06-1.13) | 0.47(0.44-0.49) |
| **Puerto Rico** | 53.96(52.98-54.94) | 42.94(41.97-43.92) | 26.38(25.45-27.31) | 18.18(17.40-18.96) | 10.54(9.94-11.15) | 5.43(5.05-5.81) | 2.56(2.41-2.71) | 1.34(1.26-1.43) | 0.51(0.46-0.57) |
| **Qatar** | 52.56(51.47-53.66) | 42.63(41.55-43.72) | 26.07(25.08-27.06) | 17.45(16.52-18.37) | 10.48(9.59-11.37) | 5.84(5.07-6.61) | 2.88(2.37-3.39) | 1.34(0.97-1.71) | 0.36(0.14-0.59) |
| **Republic of Korea** | 47.13(46.90-47.36) | 41.29(41.07-41.52) | 26.13(25.92-26.34) | 16.59(16.39-16.78) | 9.50(9.33-9.66) | 4.84(4.73-4.96) | 2.34(2.29-2.40) | 1.28(1.26-1.31) | 0.53(0.51-0.55) |
| **Republic of Moldova** | 48.60(47.66-49.55) | 42.56(41.65-43.46) | 28.23(27.40-29.05) | 18.66(17.99-19.34) | 10.56(10.05-11.08) | 5.35(5.07-5.62) | 2.23(2.14-2.33) | 0.94(0.88-1.00) | 0.28(0.24-0.31) |
| **Romania** | 51.58(51.19-51.97) | 44.95(44.57-45.33) | 29.57(29.22-29.93) | 19.70(19.41-20.00) | 11.13(10.91-11.35) | 5.42(5.30-5.53) | 2.21(2.17-2.25) | 0.94(0.92-0.97) | 0.25(0.24-0.26) |
| **Russian Federation** | 50.88(50.73-51.04) | 44.70(44.55-44.85) | 29.04(28.90-29.17) | 18.91(18.80-19.01) | 10.48(10.41-10.55) | 5.15(5.12-5.19) | 2.08(2.06-2.09) | 0.83(0.82-0.84) | 0.21(0.20-0.21) |
| **Rwanda** | 37.28(36.69-37.87) | 33.15(32.58-33.71) | 22.79(22.27-23.31) | 14.87(14.44-15.31) | 8.56(8.25-8.87) | 4.30(4.13-4.46) | 1.78(1.70-1.86) | 0.72(0.66-0.77) | 0.16(0.13-0.19) |
| **Saint Kitts and Nevis** | 52.34(45.62-59.05) | 41.38(34.91-47.86) | 24.97(19.05-30.88) | 16.89(11.94-21.84) | 9.48(5.68-13.28) | 4.58(2.53-6.63) | 1.88(1.04-2.72) | 0.81(0.20-1.42) | 0.21(-0.12-0.54) |
| **Saint Lucia** | 52.32(48.28-56.37) | 41.41(37.48-45.34) | 25.05(21.42-28.69) | 17.03(14.04-20.03) | 9.62(7.41-11.83) | 4.70(3.47-5.93) | 1.99(1.50-2.48) | 0.91(0.60-1.22) | 0.27(0.10-0.44) |
| **Saint Vincent and the Grenadines** | 52.21(46.99-57.42) | 41.23(36.15-46.32) | 24.84(20.13-29.55) | 16.83(12.96-20.70) | 9.41(6.63-12.18) | 4.54(3.05-6.03) | 1.92(1.31-2.53) | 0.87(0.48-1.27) | 0.25(0.04-0.47) |
| **Samoa** | 52.96(48.94-56.98) | 46.00(42.05-49.95) | 29.42(25.65-33.20) | 19.03(15.76-22.31) | 10.75(8.24-13.27) | 5.42(3.98-6.85) | 2.15(1.54-2.76) | 0.90(0.51-1.29) | 0.23(0.02-0.44) |
| **San Marino** | 60.39(53.74-67.04) | 50.89(44.37-57.42) | 30.10(23.81-36.40) | 18.75(13.01-24.50) | 10.23(5.18-15.28) | 4.81(0.88-8.73) | 2.20(0.28-4.13) | 1.29(0.40-2.17) | 0.61(0.03-1.19) |
| **Sao Tome and Principe** | 52.53(48.74-56.32) | 44.36(40.65-48.07) | 27.82(24.28-31.36) | 18.13(14.99-21.28) | 10.25(7.70-12.81) | 5.05(3.48-6.63) | 1.99(1.32-2.66) | 0.87(0.41-1.33) | 0.21(-0.01-0.43) |
| **Saudi Arabia** | 53.12(52.87-53.38) | 43.46(43.21-43.71) | 27.33(27.09-27.56) | 17.50(17.30-17.70) | 9.92(9.76-10.09) | 5.21(5.08-5.33) | 2.37(2.30-2.44) | 1.06(1.00-1.11) | 0.29(0.25-0.32) |
| **Senegal** | 50.53(50.07-50.99) | 42.60(42.15-43.05) | 26.61(26.19-27.04) | 17.20(16.83-17.58) | 9.54(9.25-9.82) | 4.54(4.38-4.70) | 1.66(1.60-1.73) | 0.67(0.63-0.71) | 0.14(0.12-0.16) |
| **Serbia** | 51.88(51.37-52.39) | 45.29(44.79-45.78) | 29.99(29.52-30.47) | 20.11(19.69-20.54) | 11.52(11.16-11.88) | 5.68(5.46-5.89) | 2.32(2.25-2.38) | 0.97(0.93-1.00) | 0.24(0.23-0.26) |
| **Seychelles** | 56.44(51.85-61.04) | 48.56(44.12-53.00) | 30.37(25.99-34.75) | 19.33(15.61-23.04) | 10.86(7.91-13.82) | 5.61(3.78-7.44) | 2.34(1.51-3.17) | 1.01(0.52-1.51) | 0.30(0.02-0.57) |
| **Sierra Leone** | 46.66(46.07-47.26) | 39.20(38.64-39.77) | 24.19(23.67-24.71) | 15.45(15.01-15.90) | 8.47(8.13-8.80) | 3.99(3.80-4.17) | 1.46(1.38-1.54) | 0.59(0.54-0.64) | 0.13(0.11-0.15) |
| **Singapore** | 42.66(41.95-43.38) | 38.13(37.43-38.84) | 25.35(24.70-26.00) | 16.22(15.64-16.80) | 9.38(8.84-9.92) | 4.95(4.52-5.38) | 2.51(2.32-2.70) | 1.42(1.31-1.54) | 0.64(0.55-0.72) |
| **Slovakia** | 52.01(51.31-52.71) | 45.37(44.70-46.05) | 29.97(29.34-30.61) | 20.07(19.53-20.61) | 11.47(11.03-11.90) | 5.71(5.44-5.98) | 2.40(2.32-2.49) | 1.07(1.01-1.12) | 0.29(0.27-0.32) |
| **Slovenia** | 52.93(51.85-54.02) | 46.28(45.21-47.35) | 30.82(29.81-31.83) | 20.89(19.99-21.79) | 12.26(11.48-13.04) | 6.43(5.88-6.99) | 3.00(2.78-3.21) | 1.51(1.40-1.62) | 0.52(0.46-0.59) |
| **Solomon Islands** | 51.54(49.36-53.73) | 44.64(42.52-46.77) | 28.24(26.26-30.23) | 17.96(16.33-19.59) | 9.83(8.69-10.98) | 4.70(4.05-5.35) | 1.69(1.39-2.00) | 0.63(0.44-0.83) | 0.14(0.04-0.24) |
| **Somalia** | 32.59(32.17-33.01) | 28.74(28.33-29.14) | 19.16(18.81-19.51) | 11.99(11.72-12.25) | 6.40(6.25-6.55) | 2.83(2.74-2.91) | 0.90(0.86-0.94) | 0.25(0.23-0.27) | 0.03(0.02-0.04) |
| **South Africa** | 45.79(45.55-46.02) | 39.97(39.75-40.19) | 25.17(24.98-25.36) | 15.82(15.68-15.95) | 8.53(8.44-8.62) | 3.98(3.93-4.03) | 1.45(1.43-1.47) | 0.57(0.56-0.58) | 0.12(0.11-0.12) |
| **South Asia** | 53.30(53.26-53.34) | 45.32(45.29-45.36) | 28.85(28.82-28.88) | 18.64(18.61-18.67) | 10.29(10.26-10.31) | 4.96(4.95-4.97) | 1.97(1.97-1.98) | 0.76(0.76-0.77) | 0.18(0.18-0.18) |
| **South Sudan** | 32.08(31.43-32.73) | 28.42(27.80-29.03) | 19.31(18.76-19.86) | 12.29(11.87-12.71) | 6.72(6.47-6.97) | 3.15(3.02-3.28) | 1.18(1.11-1.25) | 0.42(0.37-0.46) | 0.08(0.06-0.09) |
| **Southeast Asia** | 56.74(56.68-56.80) | 49.12(49.06-49.18) | 30.64(30.59-30.70) | 19.39(19.34-19.43) | 10.73(10.69-10.77) | 5.38(5.36-5.40) | 2.15(2.14-2.16) | 0.88(0.87-0.88) | 0.24(0.24-0.24) |
| **Southern Latin America** | 47.23(47.01-47.45) | 41.91(41.70-42.13) | 26.92(26.71-27.12) | 17.36(17.18-17.54) | 9.87(9.72-10.01) | 4.89(4.80-4.99) | 2.10(2.07-2.13) | 1.04(1.02-1.06) | 0.38(0.37-0.40) |
| **Southern Sub-Saharan Africa** | 44.90(44.70-45.09) | 39.02(38.83-39.20) | 24.53(24.37-24.69) | 15.34(15.23-15.45) | 8.16(8.09-8.24) | 3.72(3.68-3.76) | 1.29(1.27-1.30) | 0.48(0.47-0.49) | 0.09(0.09-0.09) |
| **Spain** | 60.52(60.35-60.69) | 50.61(50.44-50.78) | 29.48(29.31-29.64) | 18.25(18.10-18.39) | 9.86(9.73-9.99) | 4.58(4.49-4.68) | 2.04(2.00-2.08) | 1.15(1.13-1.17) | 0.51(0.50-0.52) |
| **Sri Lanka** | 57.86(57.52-58.19) | 49.91(49.58-50.24) | 31.56(31.25-31.87) | 20.26(19.99-20.53) | 11.44(11.22-11.67) | 6.00(5.85-6.15) | 2.61(2.55-2.67) | 1.19(1.15-1.22) | 0.38(0.36-0.40) |
| **Sudan** | 52.58(52.32-52.85) | 43.01(42.75-43.26) | 27.08(26.84-27.33) | 17.13(16.91-17.34) | 9.26(9.08-9.43) | 4.55(4.45-4.66) | 1.91(1.86-1.96) | 0.77(0.74-0.80) | 0.17(0.16-0.19) |
| **Suriname** | 51.53(49.22-53.83) | 40.74(38.49-43.00) | 24.60(22.51-26.69) | 16.61(14.91-18.31) | 9.25(8.03-10.47) | 4.45(3.83-5.07) | 1.84(1.57-2.11) | 0.84(0.65-1.02) | 0.25(0.15-0.36) |
| **Sweden** | 61.19(60.81-61.57) | 52.05(51.67-52.43) | 30.24(29.88-30.61) | 18.80(18.47-19.13) | 10.17(9.87-10.47) | 4.78(4.54-5.01) | 2.14(2.04-2.24) | 1.19(1.15-1.24) | 0.51(0.48-0.54) |
| **Switzerland** | 57.93(57.51-58.34) | 49.50(49.09-49.90) | 29.78(29.40-30.17) | 18.63(18.27-18.98) | 10.18(9.86-10.51) | 4.85(4.60-5.11) | 2.23(2.12-2.34) | 1.28(1.23-1.34) | 0.58(0.55-0.62) |
| **Syrian Arab Republic** | 55.65(55.20-56.11) | 45.79(45.35-46.23) | 29.31(28.90-29.73) | 18.24(17.90-18.58) | 9.79(9.54-10.04) | 4.97(4.82-5.12) | 2.17(2.11-2.24) | 0.93(0.89-0.98) | 0.22(0.19-0.24) |
| **Taiwan (Province of China)** | 52.09(51.74-52.43) | 45.73(45.39-46.07) | 30.86(30.55-31.17) | 20.90(20.62-21.18) | 12.58(12.34-12.81) | 7.10(6.94-7.27) | 3.41(3.33-3.50) | 1.72(1.68-1.77) | 0.65(0.62-0.68) |
| **Tajikistan** | 49.84(49.27-50.41) | 43.37(42.82-43.92) | 28.20(27.68-28.72) | 18.56(18.09-19.03) | 10.43(10.05-10.81) | 5.06(4.83-5.28) | 1.99(1.89-2.08) | 0.80(0.73-0.87) | 0.19(0.16-0.23) |
| **Thailand** | 58.87(58.66-59.08) | 50.07(49.87-50.28) | 30.82(30.64-31.00) | 19.52(19.37-19.67) | 10.93(10.82-11.03) | 5.71(5.64-5.78) | 2.51(2.48-2.54) | 1.20(1.18-1.22) | 0.42(0.41-0.44) |
| **Timor-Leste** | 54.67(53.18-56.16) | 47.02(45.58-48.46) | 29.38(28.01-30.76) | 18.50(17.26-19.74) | 10.07(9.09-11.06) | 4.98(4.39-5.57) | 1.93(1.72-2.15) | 0.74(0.62-0.87) | 0.18(0.11-0.25) |
| **Togo** | 49.26(48.64-49.87) | 41.45(40.85-42.04) | 25.73(25.18-26.28) | 16.52(16.07-16.98) | 9.01(8.68-9.33) | 4.21(4.03-4.39) | 1.52(1.44-1.60) | 0.60(0.55-0.66) | 0.13(0.10-0.15) |
| **Tokelau** | 50.10(-7.02-107.23) | 43.49(-10.51-97.49) | 27.92(-21.51-77.36) | 18.17(-24.28-60.62) | 10.39(-23.70-44.48) | 5.30(-17.55-28.16) | 2.18(-7.27-11.63) | 0.95(-3.33-5.22) | 0.26(-1.75-2.27) |
| **Tonga** | 53.89(48.32-59.46) | 46.94(41.45-52.42) | 30.35(25.07-35.62) | 19.81(15.11-24.52) | 11.29(7.55-15.03) | 5.76(3.51-8.01) | 2.37(1.42-3.33) | 1.04(0.47-1.62) | 0.30(-0.01-0.61) |
| **Trinidad and Tobago** | 51.95(50.42-53.49) | 41.00(39.49-42.51) | 24.58(23.20-25.97) | 16.56(15.51-17.61) | 9.24(8.47-10.00) | 4.44(4.04-4.85) | 1.82(1.68-1.97) | 0.81(0.71-0.90) | 0.23(0.18-0.29) |
| **Tropical Latin America** | 60.54(60.45-60.64) | 41.93(41.83-42.02) | 23.02(22.93-23.11) | 15.79(15.72-15.86) | 8.86(8.81-8.92) | 4.32(4.28-4.35) | 1.87(1.86-1.88) | 0.89(0.88-0.90) | 0.29(0.28-0.29) |
| **Tunisia** | 55.06(54.61-55.52) | 45.22(44.77-45.66) | 28.80(28.38-29.22) | 18.37(18.01-18.73) | 10.22(9.93-10.52) | 5.26(5.08-5.45) | 2.35(2.28-2.43) | 1.05(1.00-1.09) | 0.27(0.24-0.29) |
| **Turkey** | 54.67(54.50-54.84) | 45.01(44.84-45.17) | 28.95(28.79-29.10) | 18.64(18.51-18.78) | 10.52(10.41-10.64) | 5.50(5.42-5.57) | 2.52(2.49-2.55) | 1.16(1.14-1.18) | 0.31(0.30-0.32) |
| **Turkmenistan** | 49.42(48.63-50.21) | 42.88(42.12-43.65) | 27.61(26.90-28.32) | 18.06(17.46-18.65) | 10.11(9.67-10.55) | 4.83(4.60-5.07) | 1.84(1.74-1.94) | 0.72(0.65-0.79) | 0.17(0.14-0.20) |
| **Tuvalu** | 51.93(35.48-68.37) | 45.02(29.06-60.99) | 28.62(13.67-43.56) | 18.37(5.79-30.94) | 10.30(1.09-19.52) | 5.11(0.14-10.08) | 1.95(-0.04-3.95) | 0.78(-0.47-2.03) | 0.19(-0.49-0.86) |
| **Uganda** | 35.65(35.32-35.98) | 31.64(31.32-31.96) | 21.62(21.33-21.91) | 13.91(13.67-14.15) | 7.82(7.65-7.98) | 3.82(3.73-3.90) | 1.53(1.48-1.57) | 0.59(0.56-0.62) | 0.12(0.11-0.14) |
| **Ukraine** | 49.73(49.44-50.03) | 43.97(43.68-44.25) | 28.97(28.71-29.23) | 19.02(18.82-19.22) | 10.69(10.55-10.83) | 5.37(5.30-5.45) | 2.23(2.20-2.25) | 0.92(0.91-0.94) | 0.24(0.23-0.25) |
| **United Arab Emirates** | 53.01(52.40-53.62) | 43.09(42.49-43.69) | 26.55(25.98-27.11) | 16.45(16.02-16.88) | 9.89(9.49-10.30) | 5.42(5.07-5.78) | 2.58(2.32-2.83) | 1.06(0.87-1.26) | 0.24(0.11-0.37) |
| **United Kingdom** | 60.24(60.08-60.40) | 51.50(51.34-51.65) | 30.14(29.99-30.29) | 18.55(18.42-18.69) | 9.88(9.77-10.00) | 4.50(4.43-4.58) | 1.93(1.90-1.96) | 1.03(1.02-1.05) | 0.41(0.41-0.42) |
| **United Republic of Tanzania** | 36.95(36.66-37.23) | 32.74(32.47-33.01) | 22.40(22.15-22.65) | 14.41(14.20-14.61) | 8.18(8.04-8.32) | 4.06(3.99-4.14) | 1.67(1.63-1.70) | 0.67(0.65-0.70) | 0.15(0.14-0.16) |
| **United States Virgin Islands** | 53.04(45.97-60.10) | 42.14(35.10-49.19) | 25.52(18.92-32.13) | 17.38(12.44-22.31) | 9.96(6.45-13.48) | 5.07(3.01-7.14) | 2.33(1.59-3.08) | 1.19(0.74-1.64) | 0.43(0.12-0.74) |
| **United States of America** | 57.94(57.86-58.03) | 50.87(50.78-50.95) | 29.60(29.52-29.68) | 17.92(17.85-17.99) | 9.44(9.39-9.50) | 4.41(4.38-4.45) | 1.84(1.83-1.85) | 0.93(0.92-0.93) | 0.35(0.35-0.36) |
| **Uruguay** | 47.16(46.12-48.20) | 41.87(40.85-42.89) | 26.83(25.87-27.79) | 17.30(16.48-18.11) | 9.72(9.09-10.35) | 4.77(4.41-5.14) | 2.01(1.89-2.12) | 0.98(0.91-1.05) | 0.35(0.31-0.39) |
| **Uzbekistan** | 51.04(50.74-51.35) | 44.49(44.20-44.78) | 29.08(28.81-29.36) | 19.29(19.04-19.53) | 11.00(10.80-11.20) | 5.46(5.33-5.58) | 2.26(2.20-2.31) | 0.98(0.94-1.01) | 0.26(0.24-0.28) |
| **Vanuatu** | 51.62(48.40-54.83) | 44.71(41.59-47.84) | 28.31(25.40-31.22) | 17.99(15.56-20.41) | 9.85(8.14-11.55) | 4.71(3.77-5.65) | 1.68(1.26-2.10) | 0.61(0.34-0.88) | 0.13(0.00-0.26) |
| **Venezuela (Bolivarian Republic of)** | 51.07(50.70-51.44) | 40.44(40.08-40.80) | 24.98(24.66-25.31) | 16.31(16.06-16.55) | 8.99(8.81-9.16) | 4.32(4.23-4.41) | 1.83(1.79-1.86) | 0.78(0.76-0.81) | 0.22(0.21-0.23) |
| **Viet Nam** | 57.01(56.86-57.16) | 49.16(49.01-49.31) | 31.01(30.87-31.14) | 19.83(19.71-19.95) | 11.13(11.02-11.23) | 5.73(5.67-5.80) | 2.40(2.37-2.43) | 1.03(1.01-1.05) | 0.30(0.29-0.31) |
| **Western Europe** | 60.58(60.53-60.64) | 51.01(50.95-51.07) | 29.69(29.64-29.75) | 18.34(18.29-18.39) | 9.85(9.80-9.89) | 4.53(4.50-4.56) | 1.99(1.97-2.00) | 1.10(1.09-1.10) | 0.47(0.46-0.47) |
| **Western Sub-Saharan Africa** | 48.40(48.32-48.48) | 41.01(40.93-41.09) | 25.36(25.28-25.43) | 16.26(16.19-16.32) | 8.97(8.92-9.01) | 4.27(4.24-4.30) | 1.58(1.57-1.59) | 0.64(0.63-0.65) | 0.14(0.13-0.14) |
| **Yemen** | 51.48(51.16-51.79) | 41.93(41.62-42.23) | 26.08(25.80-26.36) | 16.29(16.06-16.53) | 8.76(8.58-8.95) | 4.27(4.16-4.38) | 1.71(1.67-1.76) | 0.66(0.63-0.69) | 0.13(0.12-0.15) |
| **Zambia** | 36.96(36.52-37.40) | 32.34(31.92-32.76) | 21.37(20.99-21.74) | 13.25(12.97-13.53) | 7.08(6.91-7.25) | 3.22(3.12-3.31) | 1.12(1.07-1.17) | 0.35(0.33-0.38) | 0.05(0.04-0.06) |
| **Zimbabwe** | 42.82(42.38-43.26) | 36.85(36.43-37.27) | 22.94(22.57-23.30) | 14.05(13.79-14.32) | 7.13(6.98-7.28) | 2.94(2.87-3.02) | 0.82(0.79-0.85) | 0.22(0.21-0.24) | 0.02(0.02-0.03) |

Table S2. Age-conditional probability (%) of developing migraine, males, 2021.

| Region | Birth to death | 10 years to death | 20 years to death | 30 years to death | 40 years to death | 50 years to death | 60 years to death | 70 years to death | 80 years to death |
| --- | --- | --- | --- | --- | --- | --- | --- | --- | --- |
| Global | 41.99(41.96-42.02) | 36.30(36.27-36.33) | 23.41(23.39-23.44) | 15.34(15.32-15.36) | 8.79(8.77-8.81) | 4.49(4.49-4.50) | 1.93(1.93-1.94) | 0.82(0.82-0.82) | 0.22(0.22-0.22) |
| Afghanistan | 39.08(38.63-39.53) | 30.75(30.33-31.18) | 17.93(17.54-18.32) | 11.66(11.33-11.98) | 6.62(6.42-6.82) | 3.19(3.08-3.31) | 1.18(1.11-1.26) | 0.34(0.31-0.37) | 0.04(0.03-0.05) |
| Albania | 40.25(38.44-42.05) | 35.48(33.73-37.23) | 23.93(22.26-25.59) | 16.83(15.34-18.31) | 10.32(9.15-11.48) | 5.53(4.94-6.13) | 2.57(2.37-2.78) | 1.17(1.04-1.29) | 0.29(0.22-0.35) |
| Algeria | 44.83(44.41-45.25) | 36.15(35.74-36.56) | 22.64(22.26-23.02) | 15.57(15.25-15.90) | 9.68(9.43-9.93) | 5.45(5.30-5.61) | 2.68(2.61-2.75) | 1.17(1.13-1.22) | 0.28(0.26-0.30) |
| American Samoa | 45.22(32.37-58.07) | 39.80(27.24-52.36) | 25.76(13.84-37.68) | 16.71(7.38-26.05) | 9.54(3.50-15.57) | 4.81(1.73-7.89) | 1.92(0.47-3.37) | 0.80(-0.18-1.77) | 0.21(-0.33-0.75) |
| Andean Latin America | 30.79(30.41-31.16) | 25.50(25.14-25.86) | 16.10(15.77-16.43) | 11.04(10.78-11.30) | 6.64(6.47-6.80) | 3.49(3.41-3.57) | 1.49(1.45-1.53) | 0.60(0.57-0.62) | 0.15(0.14-0.16) |
| Andorra | 47.51(39.64-55.38) | 40.40(32.64-48.16) | 25.72(18.43-33.00) | 16.64(10.56-22.71) | 8.97(4.08-13.85) | 4.33(1.22-7.43) | 2.02(0.79-3.25) | 1.11(0.29-1.92) | 0.44(-0.13-1.01) |
| Angola | 36.98(36.47-37.50) | 32.31(31.81-32.80) | 20.72(20.27-21.16) | 13.23(12.88-13.58) | 7.16(6.95-7.37) | 3.26(3.15-3.36) | 1.10(1.04-1.15) | 0.35(0.31-0.38) | 0.05(0.04-0.06) |
| Antigua and Barbuda | 41.12(31.67-50.57) | 33.62(24.34-42.89) | 20.47(11.84-29.11) | 14.05(6.75-21.36) | 8.45(2.93-13.97) | 4.46(1.60-7.33) | 1.92(0.83-3.01) | 0.84(0.11-1.56) | 0.22(-0.19-0.64) |
| Argentina | 36.19(35.73-36.64) | 32.13(31.68-32.57) | 21.21(20.80-21.63) | 13.87(13.52-14.21) | 7.90(7.64-8.15) | 3.96(3.83-4.09) | 1.71(1.66-1.76) | 0.80(0.76-0.83) | 0.25(0.23-0.26) |
| Armenia | 40.86(39.19-42.54) | 36.08(34.45-37.71) | 24.16(22.63-25.68) | 16.54(15.29-17.79) | 9.90(8.95-10.86) | 5.17(4.68-5.65) | 2.25(2.07-2.43) | 0.96(0.83-1.09) | 0.24(0.19-0.29) |
| Australasia | 43.33(42.83-43.83) | 38.17(37.68-38.66) | 24.92(24.45-25.39) | 16.56(16.15-16.98) | 9.43(9.09-9.78) | 4.87(4.65-5.10) | 2.32(2.24-2.40) | 1.30(1.25-1.35) | 0.50(0.47-0.53) |
| Australia | 43.13(42.58-43.68) | 37.93(37.38-38.47) | 24.79(24.27-25.31) | 16.48(16.03-16.93) | 9.38(9.00-9.76) | 4.85(4.61-5.10) | 2.31(2.22-2.40) | 1.30(1.25-1.35) | 0.50(0.47-0.54) |
| Austria | 45.87(45.06-46.68) | 39.29(38.49-40.09) | 25.28(24.53-26.03) | 16.40(15.75-17.06) | 8.81(8.27-9.36) | 4.23(3.91-4.55) | 1.95(1.84-2.05) | 1.04(0.97-1.11) | 0.39(0.35-0.44) |
| Azerbaijan | 39.52(38.64-40.39) | 34.83(34.00-35.66) | 23.15(22.39-23.91) | 15.71(15.08-16.33) | 9.25(8.79-9.70) | 4.67(4.45-4.88) | 1.88(1.78-1.98) | 0.71(0.63-0.78) | 0.14(0.11-0.17) |
| Bahamas | 39.55(35.22-43.88) | 32.06(27.85-36.27) | 18.95(15.12-22.78) | 12.62(9.82-15.42) | 7.25(5.57-8.93) | 3.60(2.79-4.41) | 1.41(1.01-1.82) | 0.56(0.29-0.83) | 0.13(0.00-0.26) |
| Bahrain | 45.42(43.58-47.27) | 36.63(34.83-38.44) | 22.94(21.29-24.59) | 15.77(14.36-17.19) | 9.77(8.61-10.94) | 5.45(4.61-6.28) | 2.58(2.11-3.06) | 1.01(0.65-1.37) | 0.17(0.02-0.32) |
| Bangladesh | 46.96(46.75-47.17) | 40.35(40.16-40.55) | 26.36(26.18-26.55) | 17.24(17.07-17.40) | 9.78(9.66-9.91) | 5.01(4.94-5.09) | 2.20(2.17-2.23) | 0.91(0.89-0.93) | 0.23(0.22-0.24) |
| Barbados | 41.11(35.57-46.65) | 33.63(28.17-39.08) | 20.51(15.40-25.63) | 14.11(9.90-18.32) | 8.55(5.53-11.57) | 4.60(3.02-6.18) | 2.09(1.53-2.65) | 0.99(0.62-1.36) | 0.30(0.08-0.52) |
| Belarus | 35.40(34.38-36.42) | 31.20(30.20-32.19) | 20.87(19.97-21.77) | 14.00(13.38-14.62) | 8.22(7.85-8.58) | 4.19(4.03-4.34) | 1.69(1.62-1.77) | 0.60(0.55-0.64) | 0.12(0.10-0.13) |
| Belgium | 50.77(50.09-51.44) | 42.26(41.60-42.92) | 25.86(25.23-26.49) | 16.47(15.91-17.03) | 8.70(8.24-9.16) | 4.09(3.81-4.37) | 1.85(1.77-1.94) | 1.00(0.94-1.06) | 0.38(0.34-0.42) |
| Belize | 40.12(35.78-44.46) | 32.67(28.41-36.93) | 19.63(15.62-23.64) | 13.32(10.03-16.60) | 7.91(5.57-10.26) | 4.16(2.80-5.52) | 1.82(1.21-2.44) | 0.84(0.40-1.28) | 0.25(-0.01-0.51) |
| Benin | 41.03(40.27-41.79) | 34.96(34.22-35.69) | 21.86(21.18-22.54) | 14.16(13.59-14.73) | 7.80(7.41-8.19) | 3.67(3.47-3.88) | 1.35(1.26-1.45) | 0.50(0.44-0.56) | 0.09(0.06-0.11) |
| Bermuda | 41.64(29.59-53.68) | 34.09(22.22-45.96) | 20.86(9.72-32.01) | 14.39(5.52-23.26) | 8.74(2.41-15.07) | 4.72(1.48-7.95) | 2.15(0.98-3.32) | 1.02(0.28-1.77) | 0.31(-0.10-0.73) |
| Bhutan | 47.23(44.24-50.22) | 40.64(37.80-43.48) | 26.66(24.01-29.30) | 17.54(15.17-19.91) | 10.09(8.09-12.08) | 5.30(3.99-6.60) | 2.42(1.87-2.97) | 1.05(0.72-1.37) | 0.29(0.11-0.47) |
| Bolivia (Plurinational State of) | 30.47(29.63-31.31) | 25.08(24.27-25.89) | 15.61(14.88-16.33) | 10.53(9.98-11.08) | 6.12(5.80-6.45) | 3.03(2.88-3.18) | 1.14(1.07-1.21) | 0.37(0.33-0.41) | 0.06(0.05-0.08) |
| Bosnia and Herzegovina | 40.31(38.72-41.90) | 35.50(33.94-37.07) | 23.85(22.37-25.32) | 16.68(15.47-17.90) | 10.11(9.23-11.00) | 5.29(4.85-5.72) | 2.33(2.18-2.48) | 0.99(0.89-1.09) | 0.24(0.20-0.29) |
| Botswana | 37.15(35.49-38.81) | 32.38(30.84-33.93) | 20.54(19.22-21.86) | 12.89(12.00-13.78) | 6.73(6.25-7.22) | 2.90(2.64-3.15) | 0.89(0.77-1.02) | 0.25(0.19-0.32) | 0.03(0.01-0.04) |
| Brazil | 47.89(47.72-48.06) | 34.60(34.43-34.77) | 18.37(18.22-18.52) | 12.52(12.40-12.64) | 7.46(7.38-7.54) | 3.87(3.83-3.91) | 1.68(1.66-1.70) | 0.77(0.76-0.79) | 0.23(0.22-0.24) |
| Brunei Darussalam | 32.02(28.04-35.99) | 28.19(24.30-32.08) | 18.45(14.77-22.12) | 11.79(8.65-14.92) | 6.77(4.42-9.13) | 3.62(2.30-4.94) | 1.81(1.02-2.60) | 0.92(0.27-1.58) | 0.29(-0.19-0.76) |
| Bulgaria | 38.98(37.74-40.23) | 34.18(32.98-35.39) | 22.56(21.46-23.66) | 15.45(14.66-16.23) | 8.99(8.51-9.46) | 4.33(4.15-4.52) | 1.63(1.56-1.70) | 0.56(0.53-0.60) | 0.10(0.08-0.11) |
| Burkina Faso | 39.65(39.07-40.24) | 33.72(33.16-34.28) | 20.95(20.43-21.46) | 13.48(13.07-13.89) | 7.33(7.06-7.60) | 3.37(3.24-3.50) | 1.18(1.12-1.24) | 0.41(0.37-0.44) | 0.07(0.05-0.08) |
| Burundi | 28.69(27.84-29.54) | 25.48(24.67-26.29) | 17.33(16.61-18.05) | 11.29(10.75-11.84) | 6.48(6.13-6.83) | 3.23(3.06-3.41) | 1.35(1.25-1.45) | 0.49(0.43-0.56) | 0.08(0.06-0.11) |
| Cabo Verde | 45.49(41.99-48.99) | 38.93(35.56-42.30) | 24.73(21.66-27.81) | 16.34(13.75-18.92) | 9.30(7.29-11.31) | 4.62(3.55-5.69) | 1.87(1.37-2.38) | 0.77(0.42-1.13) | 0.16(0.02-0.30) |
| Cambodia | 46.48(45.84-47.12) | 40.51(39.90-41.13) | 25.84(25.28-26.40) | 16.44(15.98-16.91) | 9.06(8.71-9.40) | 4.38(4.21-4.56) | 1.60(1.52-1.67) | 0.54(0.50-0.59) | 0.11(0.09-0.13) |
| Cameroon | 41.09(40.63-41.56) | 34.92(34.47-35.37) | 21.61(21.20-22.01) | 13.81(13.49-14.13) | 7.42(7.21-7.62) | 3.35(3.25-3.46) | 1.14(1.09-1.20) | 0.38(0.35-0.41) | 0.06(0.05-0.07) |
| Canada | 39.14(38.65-39.64) | 34.76(34.27-35.25) | 22.47(22.01-22.94) | 14.37(13.98-14.77) | 7.85(7.54-8.16) | 4.05(3.88-4.23) | 1.90(1.85-1.96) | 1.05(1.01-1.09) | 0.41(0.38-0.44) |
| Caribbean | 38.70(38.28-39.12) | 31.47(31.07-31.87) | 18.85(18.49-19.21) | 12.74(12.45-13.02) | 7.48(7.29-7.66) | 3.83(3.74-3.91) | 1.59(1.55-1.62) | 0.68(0.65-0.70) | 0.18(0.17-0.20) |
| Central African Republic | 32.24(31.20-33.28) | 27.89(26.94-28.84) | 17.21(16.42-18.00) | 10.51(9.97-11.04) | 5.25(4.99-5.51) | 2.08(1.95-2.21) | 0.55(0.48-0.62) | 0.13(0.10-0.16) | 0.01(0.00-0.02) |
| Central Asia | 39.64(39.35-39.94) | 34.92(34.64-35.21) | 23.17(22.90-23.43) | 15.68(15.47-15.90) | 9.21(9.06-9.36) | 4.65(4.58-4.73) | 1.90(1.86-1.94) | 0.74(0.71-0.76) | 0.16(0.14-0.17) |
| Central Europe | 40.39(40.11-40.66) | 35.65(35.38-35.92) | 23.85(23.60-24.10) | 16.59(16.39-16.79) | 9.97(9.83-10.11) | 5.14(5.07-5.20) | 2.22(2.19-2.24) | 0.93(0.91-0.94) | 0.22(0.22-0.23) |
| Central Latin America | 37.43(37.25-37.61) | 30.44(30.26-30.62) | 18.44(18.28-18.60) | 12.47(12.35-12.60) | 7.57(7.49-7.65) | 3.99(3.95-4.03) | 1.74(1.72-1.76) | 0.74(0.73-0.76) | 0.21(0.20-0.21) |
| Central Sub-Saharan Africa | 36.88(36.64-37.13) | 32.22(31.99-32.46) | 20.67(20.47-20.88) | 13.23(13.07-13.40) | 7.19(7.09-7.29) | 3.29(3.24-3.33) | 1.12(1.09-1.14) | 0.36(0.34-0.37) | 0.05(0.04-0.06) |
| Chad | 38.72(38.05-39.40) | 32.92(32.28-33.57) | 20.46(19.87-21.06) | 13.18(12.69-13.67) | 7.19(6.87-7.51) | 3.33(3.17-3.48) | 1.19(1.11-1.26) | 0.42(0.38-0.47) | 0.07(0.05-0.09) |
| Chile | 34.17(33.45-34.89) | 30.63(29.93-31.34) | 20.70(20.04-21.36) | 13.75(13.19-14.30) | 7.96(7.55-8.38) | 4.16(3.96-4.37) | 1.95(1.86-2.03) | 0.99(0.93-1.05) | 0.35(0.31-0.39) |
| China | 39.04(38.95-39.12) | 35.42(35.34-35.50) | 24.60(24.53-24.68) | 16.76(16.70-16.82) | 10.15(10.11-10.20) | 5.61(5.59-5.64) | 2.59(2.58-2.60) | 1.14(1.13-1.14) | 0.31(0.30-0.31) |
| Colombia | 38.45(38.01-38.89) | 30.99(30.56-31.42) | 18.92(18.53-19.32) | 12.98(12.65-13.30) | 8.08(7.84-8.33) | 4.43(4.30-4.57) | 2.07(2.01-2.12) | 0.95(0.91-0.98) | 0.29(0.27-0.31) |
| Comoros | 30.14(26.49-33.79) | 26.86(23.41-30.30) | 18.50(15.39-21.60) | 12.27(9.79-14.75) | 7.25(5.65-8.86) | 3.81(3.01-4.62) | 1.73(1.28-2.18) | 0.71(0.41-1.00) | 0.14(0.01-0.27) |
| Congo | 38.00(36.79-39.22) | 33.23(32.08-34.38) | 21.39(20.36-22.41) | 13.74(12.97-14.51) | 7.52(7.06-7.97) | 3.50(3.26-3.73) | 1.24(1.11-1.37) | 0.42(0.34-0.50) | 0.06(0.03-0.09) |
| Cook Islands | 46.23(23.64-68.82) | 40.77(18.56-62.98) | 26.62(5.30-47.95) | 17.47(-0.32-35.26) | 10.15(-2.20-22.50) | 5.27(-0.85-11.38) | 2.21(-0.18-4.60) | 0.98(-0.51-2.47) | 0.29(-0.57-1.14) |
| Costa Rica | 38.41(37.03-39.80) | 31.09(29.72-32.46) | 19.14(17.87-20.40) | 13.17(12.15-14.19) | 8.23(7.49-8.97) | 4.56(4.16-4.95) | 2.19(2.01-2.38) | 1.07(0.94-1.20) | 0.37(0.28-0.45) |
| Croatia | 40.49(39.08-41.89) | 35.72(34.34-37.09) | 24.27(22.98-25.57) | 17.03(15.94-18.11) | 10.42(9.59-11.26) | 5.55(5.11-5.98) | 2.54(2.39-2.69) | 1.14(1.04-1.23) | 0.30(0.25-0.34) |
| Cuba | 40.91(40.04-41.77) | 33.37(32.53-34.22) | 20.17(19.39-20.95) | 13.72(13.08-14.36) | 8.11(7.68-8.54) | 4.16(3.99-4.33) | 1.71(1.64-1.78) | 0.71(0.67-0.76) | 0.18(0.16-0.20) |
| Cyprus | 47.42(45.45-49.39) | 40.32(38.38-42.27) | 25.66(23.77-27.55) | 16.59(14.96-18.21) | 8.92(7.44-10.39) | 4.27(3.27-5.26) | 1.95(1.63-2.27) | 1.03(0.83-1.23) | 0.37(0.24-0.51) |
| Czechia | 40.70(39.79-41.61) | 35.88(34.99-36.78) | 24.20(23.36-25.04) | 17.01(16.33-17.69) | 10.42(9.92-10.93) | 5.57(5.28-5.85) | 2.55(2.46-2.65) | 1.15(1.09-1.21) | 0.31(0.28-0.35) |
| Côte d’Ivoire | 41.36(40.86-41.86) | 35.22(34.75-35.70) | 21.99(21.56-22.43) | 14.22(13.87-14.57) | 7.81(7.58-8.04) | 3.66(3.54-3.79) | 1.34(1.28-1.41) | 0.50(0.46-0.54) | 0.09(0.07-0.11) |
| Democratic People's Republic of Korea | 38.15(37.55-38.74) | 34.08(33.52-34.65) | 23.22(22.71-23.72) | 15.44(15.04-15.84) | 9.10(8.83-9.37) | 4.82(4.69-4.95) | 2.02(1.96-2.09) | 0.82(0.77-0.86) | 0.22(0.19-0.25) |
| Democratic Republic of the Congo | 37.08(36.78-37.38) | 32.41(32.12-32.69) | 20.84(20.59-21.10) | 13.39(13.18-13.59) | 7.31(7.19-7.44) | 3.37(3.31-3.44) | 1.16(1.13-1.20) | 0.38(0.36-0.40) | 0.05(0.05-0.06) |
| Denmark | 44.55(43.55-45.55) | 38.50(37.52-39.48) | 25.06(24.12-26.00) | 16.35(15.50-17.20) | 8.87(8.15-9.59) | 4.29(3.85-4.74) | 1.98(1.84-2.11) | 1.05(0.97-1.14) | 0.39(0.34-0.45) |
| Djibouti | 29.82(27.23-32.42) | 26.50(24.07-28.93) | 18.05(15.95-20.15) | 11.77(10.22-13.32) | 6.75(5.84-7.67) | 3.38(2.90-3.87) | 1.42(1.12-1.72) | 0.52(0.32-0.72) | 0.09(0.00-0.18) |
| Dominica | 39.53(28.46-50.60) | 32.18(21.58-42.78) | 19.32(9.59-29.05) | 13.07(5.22-20.92) | 7.65(2.36-12.95) | 3.87(1.67-6.07) | 1.54(0.62-2.46) | 0.61(-0.02-1.23) | 0.13(-0.19-0.46) |
| Dominican Republic | 39.78(38.91-40.65) | 32.42(31.58-33.27) | 19.57(18.80-20.34) | 13.34(12.71-13.96) | 7.96(7.51-8.41) | 4.19(3.96-4.43) | 1.85(1.74-1.96) | 0.86(0.78-0.94) | 0.27(0.22-0.31) |
| East Asia | 39.06(38.98-39.14) | 35.42(35.34-35.50) | 24.59(24.52-24.67) | 16.75(16.69-16.81) | 10.14(10.09-10.19) | 5.60(5.58-5.63) | 2.58(2.57-2.59) | 1.14(1.13-1.14) | 0.31(0.30-0.31) |
| Eastern Europe | 37.85(37.64-38.07) | 33.41(33.20-33.62) | 21.95(21.76-22.14) | 14.64(14.51-14.76) | 8.53(8.46-8.61) | 4.37(4.33-4.40) | 1.81(1.79-1.82) | 0.68(0.66-0.69) | 0.14(0.14-0.15) |
| Eastern Sub-Saharan Africa | 28.33(28.19-28.48) | 25.24(25.10-25.38) | 17.07(16.95-17.20) | 11.07(10.98-11.16) | 6.27(6.22-6.32) | 3.06(3.03-3.09) | 1.23(1.21-1.24) | 0.42(0.41-0.43) | 0.07(0.06-0.07) |
| Ecuador | 34.56(33.83-35.28) | 28.02(27.31-28.73) | 17.28(16.62-17.94) | 11.93(11.40-12.46) | 7.27(6.89-7.64) | 3.92(3.73-4.12) | 1.76(1.68-1.85) | 0.79(0.73-0.85) | 0.24(0.20-0.27) |
| Egypt | 44.39(44.13-44.64) | 34.21(33.96-34.46) | 20.09(19.85-20.32) | 13.64(13.44-13.84) | 8.36(8.22-8.50) | 4.47(4.40-4.53) | 1.94(1.91-1.97) | 0.65(0.63-0.67) | 0.11(0.10-0.12) |
| El Salvador | 37.09(35.97-38.21) | 29.78(28.68-30.88) | 17.86(16.88-18.83) | 11.98(11.22-12.74) | 7.22(6.73-7.71) | 3.81(3.54-4.07) | 1.70(1.57-1.83) | 0.76(0.67-0.84) | 0.23(0.18-0.28) |
| Equatorial Guinea | 37.49(35.37-39.62) | 32.75(30.75-34.75) | 21.00(19.19-22.81) | 13.41(11.95-14.87) | 7.26(6.28-8.25) | 3.35(2.79-3.91) | 1.18(0.88-1.48) | 0.39(0.22-0.57) | 0.06(0.00-0.12) |
| Eritrea | 28.74(27.62-29.85) | 25.45(24.41-26.50) | 17.12(16.23-18.01) | 10.98(10.33-11.63) | 6.13(5.75-6.51) | 2.92(2.71-3.13) | 1.13(1.00-1.27) | 0.37(0.29-0.46) | 0.05(0.02-0.08) |
| Estonia | 36.77(33.98-39.57) | 32.56(29.82-35.30) | 22.21(19.70-24.72) | 15.31(13.34-17.29) | 9.43(8.03-10.84) | 5.22(4.55-5.90) | 2.44(2.18-2.71) | 1.07(0.90-1.24) | 0.30(0.21-0.39) |
| Eswatini | 34.57(32.35-36.80) | 29.82(27.81-31.84) | 18.10(16.51-19.69) | 10.70(9.76-11.64) | 5.03(4.56-5.50) | 1.81(1.56-2.06) | 0.39(0.29-0.49) | 0.07(0.04-0.10) | 0.00(0.00-0.01) |
| Ethiopia | 28.00(27.70-28.31) | 25.27(24.98-25.57) | 17.37(17.11-17.64) | 11.59(11.37-11.80) | 6.84(6.71-6.98) | 3.54(3.46-3.61) | 1.54(1.50-1.58) | 0.59(0.56-0.61) | 0.11(0.10-0.12) |
| Fiji | 43.94(41.12-46.76) | 38.57(35.88-41.26) | 24.70(22.26-27.13) | 15.77(13.86-17.68) | 8.67(7.46-9.89) | 4.04(3.51-4.57) | 1.33(1.10-1.57) | 0.42(0.28-0.56) | 0.06(0.01-0.12) |
| Finland | 47.28(46.18-48.39) | 40.19(39.10-41.28) | 25.53(24.51-26.55) | 16.49(15.61-17.36) | 8.85(8.14-9.57) | 4.24(3.82-4.67) | 1.96(1.83-2.09) | 1.06(0.98-1.14) | 0.42(0.36-0.47) |
| France | 47.96(47.66-48.26) | 40.63(40.33-40.92) | 25.88(25.60-26.17) | 16.70(16.45-16.95) | 8.93(8.73-9.14) | 4.24(4.12-4.36) | 1.97(1.93-2.00) | 1.08(1.06-1.11) | 0.43(0.42-0.45) |
| Gabon | 38.25(36.10-40.41) | 33.46(31.41-35.51) | 21.55(19.72-23.38) | 13.85(12.44-15.27) | 7.58(6.73-8.42) | 3.51(3.12-3.89) | 1.22(1.03-1.42) | 0.40(0.28-0.53) | 0.06(0.02-0.10) |
| Gambia | 42.40(40.65-44.15) | 36.07(34.39-37.76) | 22.41(20.86-23.97) | 14.39(13.12-15.65) | 7.79(6.96-8.61) | 3.57(3.15-4.00) | 1.26(1.06-1.46) | 0.44(0.32-0.55) | 0.07(0.03-0.11) |
| Georgia | 40.07(38.46-41.68) | 35.28(33.70-36.86) | 23.34(21.91-24.77) | 15.75(14.66-16.84) | 9.17(8.46-9.88) | 4.53(4.23-4.84) | 1.78(1.66-1.90) | 0.66(0.58-0.73) | 0.12(0.10-0.15) |
| Germany | 48.57(48.32-48.81) | 41.14(40.90-41.38) | 25.91(25.68-26.14) | 16.59(16.38-16.79) | 8.75(8.58-8.92) | 4.09(4.00-4.18) | 1.84(1.81-1.86) | 0.97(0.95-0.99) | 0.36(0.35-0.37) |
| Ghana | 42.71(42.25-43.17) | 36.37(35.93-36.82) | 22.69(22.28-23.09) | 14.64(14.31-14.97) | 7.99(7.77-8.21) | 3.71(3.59-3.82) | 1.32(1.27-1.38) | 0.47(0.43-0.50) | 0.08(0.06-0.09) |
| Greece | 48.28(47.50-49.05) | 40.69(39.93-41.45) | 25.53(24.81-26.24) | 16.35(15.75-16.94) | 8.65(8.20-9.11) | 4.02(3.77-4.27) | 1.76(1.68-1.84) | 0.91(0.86-0.96) | 0.33(0.30-0.36) |
| Greenland | 39.08(26.54-51.62) | 34.44(22.24-46.64) | 21.58(10.55-32.60) | 13.41(4.74-22.09) | 7.00(1.01-12.98) | 3.34(1.07-5.60) | 1.36(0.21-2.51) | 0.66(-0.30-1.61) | 0.21(-0.50-0.92) |
| Grenada | 40.02(31.55-48.49) | 32.55(24.34-40.76) | 19.49(11.85-27.13) | 13.11(6.71-19.50) | 7.59(3.53-11.66) | 3.76(2.09-5.42) | 1.42(0.66-2.19) | 0.51(0.02-1.01) | 0.08(-0.21-0.38) |
| Guam | 45.71(38.65-52.77) | 40.29(33.33-47.25) | 26.25(19.84-32.66) | 17.19(11.92-22.47) | 9.98(6.38-13.59) | 5.22(3.29-7.15) | 2.28(1.39-3.16) | 1.09(0.45-1.73) | 0.38(-0.05-0.82) |
| Guatemala | 36.57(35.86-37.27) | 29.38(28.69-30.07) | 17.66(17.03-18.29) | 11.86(11.36-12.36) | 7.13(6.80-7.46) | 3.70(3.52-3.88) | 1.59(1.50-1.67) | 0.65(0.60-0.71) | 0.17(0.14-0.20) |
| Guinea | 40.23(39.47-40.99) | 34.23(33.50-34.96) | 21.30(20.62-21.98) | 13.71(13.16-14.26) | 7.46(7.09-7.82) | 3.44(3.26-3.61) | 1.21(1.14-1.29) | 0.42(0.38-0.47) | 0.07(0.05-0.08) |
| Guinea-Bissau | 40.14(38.36-41.91) | 33.95(32.27-35.64) | 20.65(19.16-22.14) | 12.91(11.80-14.02) | 6.66(6.00-7.33) | 2.83(2.49-3.16) | 0.86(0.70-1.03) | 0.25(0.16-0.34) | 0.03(0.00-0.06) |
| Guyana | 38.11(35.17-41.05) | 30.74(27.91-33.57) | 17.86(15.39-20.34) | 11.67(9.83-13.51) | 6.47(5.46-7.48) | 3.01(2.55-3.46) | 1.03(0.81-1.26) | 0.34(0.21-0.48) | 0.06(0.01-0.12) |
| Haiti | 36.15(35.42-36.88) | 29.20(28.51-29.89) | 17.10(16.49-17.70) | 11.27(10.81-11.72) | 6.31(6.03-6.59) | 2.96(2.82-3.10) | 1.02(0.95-1.09) | 0.33(0.29-0.37) | 0.06(0.04-0.07) |
| High-income Asia Pacific | 33.42(33.19-33.64) | 29.66(29.44-29.89) | 19.70(19.49-19.91) | 12.87(12.69-13.05) | 7.65(7.51-7.79) | 4.33(4.25-4.41) | 2.34(2.31-2.37) | 1.31(1.30-1.33) | 0.52(0.51-0.53) |
| High-income North America | 41.80(41.65-41.96) | 37.08(36.93-37.23) | 23.13(22.98-23.27) | 14.43(14.31-14.54) | 7.68(7.59-7.76) | 3.78(3.74-3.82) | 1.63(1.62-1.64) | 0.84(0.83-0.85) | 0.29(0.29-0.30) |
| Honduras | 37.15(36.23-38.07) | 29.89(28.99-30.79) | 18.05(17.20-18.89) | 12.14(11.44-12.83) | 7.25(6.79-7.71) | 3.66(3.44-3.88) | 1.44(1.34-1.54) | 0.51(0.45-0.57) | 0.10(0.08-0.12) |
| Hungary | 40.02(39.07-40.97) | 35.21(34.28-36.13) | 23.54(22.67-24.41) | 16.37(15.67-17.07) | 9.81(9.34-10.27) | 5.00(4.79-5.22) | 2.11(2.03-2.19) | 0.88(0.83-0.93) | 0.22(0.20-0.25) |
| Iceland | 47.55(43.29-51.82) | 40.46(36.19-44.72) | 25.79(21.74-29.84) | 16.73(13.17-20.29) | 9.10(6.04-12.17) | 4.49(2.39-6.59) | 2.17(1.34-3.01) | 1.24(0.76-1.72) | 0.52(0.19-0.85) |
| India | 46.04(45.98-46.11) | 40.14(40.08-40.21) | 25.99(25.93-26.05) | 16.74(16.69-16.79) | 9.30(9.26-9.33) | 4.58(4.56-4.60) | 1.84(1.83-1.85) | 0.66(0.66-0.67) | 0.14(0.14-0.14) |
| Indonesia | 48.64(48.49-48.79) | 42.91(42.76-43.05) | 27.41(27.28-27.54) | 17.55(17.45-17.66) | 9.81(9.73-9.88) | 4.81(4.77-4.85) | 1.80(1.78-1.82) | 0.64(0.62-0.65) | 0.13(0.13-0.14) |
| Iran (Islamic Republic of) | 46.68(46.38-46.98) | 38.43(38.14-38.73) | 24.45(24.19-24.72) | 16.80(16.59-17.01) | 10.29(10.13-10.46) | 5.68(5.58-5.78) | 2.71(2.66-2.75) | 1.15(1.12-1.18) | 0.27(0.26-0.29) |
| Iraq | 43.85(43.44-44.27) | 35.19(34.79-35.60) | 21.72(21.34-22.10) | 14.70(14.39-15.02) | 8.87(8.64-9.09) | 4.72(4.59-4.84) | 2.08(2.01-2.14) | 0.76(0.72-0.80) | 0.14(0.12-0.16) |
| Ireland | 47.50(46.43-48.56) | 40.40(39.35-41.46) | 25.75(24.73-26.78) | 16.68(15.78-17.59) | 9.02(8.27-9.77) | 4.38(3.87-4.89) | 2.06(1.87-2.25) | 1.14(1.02-1.25) | 0.44(0.37-0.52) |
| Israel | 47.52(46.71-48.32) | 40.42(39.62-41.22) | 25.76(25.00-26.53) | 16.71(16.02-17.39) | 9.05(8.44-9.65) | 4.40(3.98-4.83) | 2.08(1.93-2.23) | 1.16(1.06-1.25) | 0.47(0.40-0.53) |
| Italy | 50.10(49.82-50.39) | 42.89(42.61-43.17) | 26.86(26.60-27.13) | 17.38(17.14-17.61) | 9.32(9.13-9.52) | 4.48(4.36-4.61) | 2.06(2.02-2.10) | 1.11(1.09-1.13) | 0.42(0.40-0.43) |
| Jamaica | 40.64(38.96-42.31) | 33.18(31.55-34.82) | 20.13(18.59-21.67) | 13.76(12.44-15.08) | 8.23(7.26-9.20) | 4.33(3.84-4.83) | 1.88(1.68-2.08) | 0.84(0.71-0.97) | 0.24(0.17-0.31) |
| Japan | 32.60(32.32-32.89) | 29.15(28.87-29.43) | 19.51(19.24-19.77) | 12.84(12.61-13.06) | 7.68(7.50-7.86) | 4.38(4.28-4.49) | 2.38(2.35-2.42) | 1.34(1.32-1.36) | 0.54(0.53-0.55) |
| Jordan | 45.38(44.64-46.12) | 36.65(35.93-37.38) | 23.07(22.37-23.76) | 15.94(15.31-16.57) | 9.98(9.47-10.49) | 5.69(5.36-6.02) | 2.86(2.68-3.03) | 1.30(1.18-1.42) | 0.36(0.29-0.43) |
| Kazakhstan | 39.51(38.85-40.17) | 34.73(34.09-35.37) | 22.82(22.23-23.41) | 15.25(14.80-15.70) | 8.74(8.45-9.03) | 4.23(4.09-4.36) | 1.58(1.52-1.65) | 0.54(0.50-0.58) | 0.09(0.08-0.10) |
| Kenya | 30.48(30.06-30.91) | 27.32(26.92-27.72) | 18.48(18.13-18.84) | 11.97(11.70-12.24) | 6.78(6.62-6.93) | 3.32(3.24-3.41) | 1.33(1.28-1.37) | 0.46(0.43-0.49) | 0.07(0.06-0.08) |
| Kiribati | 42.37(34.52-50.22) | 37.09(29.64-44.54) | 23.48(16.84-30.12) | 14.80(9.74-19.87) | 8.04(4.82-11.26) | 3.74(2.12-5.35) | 1.28(0.44-2.11) | 0.44(-0.15-1.02) | 0.09(-0.19-0.36) |
| Kuwait | 47.09(45.90-48.28) | 37.90(36.73-39.07) | 23.95(22.87-25.02) | 16.71(15.78-17.65) | 10.67(9.84-11.49) | 6.29(5.63-6.94) | 3.34(2.95-3.74) | 1.63(1.36-1.89) | 0.50(0.36-0.64) |
| Kyrgyzstan | 39.92(38.81-41.02) | 35.17(34.09-36.25) | 23.34(22.32-24.36) | 15.80(14.95-16.65) | 9.31(8.70-9.92) | 4.76(4.44-5.08) | 2.01(1.84-2.18) | 0.83(0.71-0.96) | 0.20(0.14-0.26) |
| Lao People's Democratic Republic | 46.09(45.12-47.05) | 40.19(39.28-41.10) | 25.70(24.86-26.53) | 16.41(15.70-17.11) | 9.10(8.57-9.62) | 4.46(4.18-4.74) | 1.67(1.55-1.79) | 0.60(0.52-0.67) | 0.13(0.09-0.17) |
| Latvia | 35.89(33.59-38.19) | 31.68(29.41-33.95) | 21.33(19.20-23.47) | 14.45(12.93-15.97) | 8.63(7.73-9.53) | 4.55(4.16-4.93) | 1.98(1.80-2.16) | 0.79(0.68-0.90) | 0.20(0.15-0.24) |
| Lebanon | 45.21(44.07-46.35) | 36.44(35.32-37.57) | 22.79(21.74-23.83) | 15.64(14.74-16.54) | 9.66(8.94-10.39) | 5.38(4.96-5.80) | 2.59(2.41-2.77) | 1.09(0.99-1.19) | 0.25(0.21-0.29) |
| Lesotho | 32.29(30.62-33.95) | 27.75(26.29-29.21) | 16.59(15.49-17.68) | 9.59(8.98-10.21) | 4.32(4.02-4.62) | 1.45(1.30-1.60) | 0.29(0.23-0.34) | 0.04(0.03-0.06) | 0.00(0.00-0.00) |
| Liberia | 41.79(40.65-42.93) | 35.62(34.53-36.71) | 22.32(21.31-23.32) | 14.49(13.67-15.30) | 8.02(7.48-8.57) | 3.83(3.50-4.15) | 1.45(1.29-1.61) | 0.56(0.45-0.66) | 0.11(0.06-0.15) |
| Libya | 43.82(42.81-44.83) | 35.19(34.22-36.16) | 21.77(20.90-22.65) | 14.80(14.11-15.49) | 9.03(8.53-9.52) | 4.93(4.63-5.23) | 2.30(2.14-2.46) | 0.94(0.84-1.04) | 0.21(0.16-0.26) |
| Lithuania | 33.83(31.84-35.83) | 30.18(28.23-32.12) | 20.72(18.97-22.48) | 14.15(12.80-15.51) | 8.54(7.74-9.34) | 4.60(4.27-4.94) | 2.07(1.91-2.22) | 0.85(0.75-0.94) | 0.22(0.17-0.26) |
| Luxembourg | 47.20(44.54-49.86) | 40.24(37.66-42.82) | 25.70(23.28-28.12) | 16.69(14.47-18.90) | 8.99(7.02-10.96) | 4.35(3.06-5.63) | 2.02(1.55-2.49) | 1.10(0.79-1.41) | 0.42(0.21-0.63) |
| Madagascar | 29.02(28.44-29.60) | 25.78(25.23-26.33) | 17.53(17.04-18.02) | 11.42(11.03-11.80) | 6.54(6.30-6.77) | 3.25(3.13-3.37) | 1.34(1.27-1.41) | 0.48(0.43-0.52) | 0.08(0.06-0.10) |
| Malawi | 27.99(27.35-28.64) | 24.73(24.13-25.34) | 16.46(15.94-16.99) | 10.37(10.00-10.73) | 5.59(5.39-5.78) | 2.50(2.40-2.61) | 0.87(0.81-0.93) | 0.24(0.21-0.27) | 0.03(0.02-0.03) |
| Malaysia | 47.82(47.37-48.27) | 41.94(41.50-42.37) | 27.18(26.79-27.58) | 17.56(17.22-17.90) | 9.85(9.59-10.12) | 4.95(4.81-5.09) | 1.96(1.90-2.01) | 0.77(0.73-0.81) | 0.20(0.18-0.23) |
| Maldives | 49.82(46.51-53.12) | 43.71(40.58-46.84) | 28.66(25.74-31.59) | 18.92(16.22-21.62) | 11.14(8.61-13.68) | 6.06(4.01-8.11) | 2.79(1.71-3.87) | 1.32(0.68-1.97) | 0.44(0.08-0.81) |
| Mali | 39.43(38.86-40.00) | 33.55(33.00-34.10) | 20.88(20.37-21.39) | 13.45(13.03-13.87) | 7.33(7.05-7.60) | 3.38(3.25-3.52) | 1.19(1.13-1.25) | 0.41(0.38-0.45) | 0.06(0.05-0.08) |
| Malta | 47.37(43.71-51.03) | 40.30(36.75-43.85) | 25.71(22.50-28.91) | 16.68(13.77-19.58) | 9.03(6.46-11.59) | 4.39(2.70-6.09) | 2.08(1.54-2.63) | 1.17(0.83-1.50) | 0.47(0.23-0.72) |
| Marshall Islands | 43.44(32.32-54.56) | 38.08(27.44-48.71) | 24.22(14.62-33.82) | 15.36(8.11-22.61) | 8.42(3.81-13.04) | 3.98(1.55-6.42) | 1.41(0.17-2.65) | 0.51(-0.35-1.37) | 0.11(-0.40-0.62) |
| Mauritania | 44.57(43.12-46.02) | 38.16(36.74-39.58) | 24.30(22.94-25.65) | 16.09(14.89-17.28) | 9.22(8.29-10.14) | 4.65(4.13-5.17) | 1.93(1.72-2.14) | 0.83(0.69-0.97) | 0.19(0.12-0.25) |
| Mauritius | 48.19(45.85-50.53) | 42.09(39.84-44.33) | 27.05(24.98-29.11) | 17.38(15.71-19.05) | 9.76(8.63-10.88) | 4.92(4.34-5.50) | 1.99(1.75-2.22) | 0.81(0.65-0.97) | 0.23(0.13-0.32) |
| Mexico | 37.35(37.10-37.59) | 30.67(30.43-30.91) | 18.57(18.35-18.78) | 12.52(12.35-12.68) | 7.53(7.42-7.63) | 3.93(3.87-3.98) | 1.68(1.65-1.70) | 0.70(0.69-0.72) | 0.19(0.18-0.20) |
| Micronesia (Federated States of) | 43.91(35.56-52.25) | 38.51(30.48-46.55) | 24.56(17.18-31.95) | 15.63(9.83-21.43) | 8.62(5.00-12.24) | 4.12(2.33-5.91) | 1.49(0.56-2.41) | 0.55(-0.14-1.25) | 0.12(-0.24-0.49) |
| Monaco | 46.86(32.15-61.57) | 39.78(25.34-54.22) | 25.14(11.46-38.82) | 16.11(4.75-27.46) | 8.51(0.24-16.78) | 3.96(0.12-7.80) | 1.73(0.48-2.98) | 0.88(0.13-1.63) | 0.31(-0.15-0.77) |
| Mongolia | 39.30(37.74-40.87) | 34.56(33.03-36.08) | 22.74(21.37-24.11) | 15.24(14.19-16.28) | 8.78(8.10-9.45) | 4.31(3.96-4.66) | 1.71(1.50-1.91) | 0.66(0.52-0.81) | 0.14(0.08-0.21) |
| Montenegro | 39.85(36.19-43.51) | 35.04(31.44-38.64) | 23.37(20.01-26.74) | 16.21(13.40-19.01) | 9.66(7.69-11.62) | 4.88(3.94-5.81) | 1.99(1.68-2.31) | 0.74(0.55-0.93) | 0.12(0.06-0.18) |
| Morocco | 44.71(44.27-45.15) | 36.02(35.59-36.46) | 22.49(22.09-22.90) | 15.42(15.07-15.76) | 9.50(9.23-9.76) | 5.24(5.10-5.39) | 2.46(2.40-2.53) | 1.00(0.96-1.04) | 0.22(0.20-0.23) |
| Mozambique | 27.08(26.58-27.57) | 23.86(23.39-24.33) | 15.72(15.33-16.11) | 9.77(9.50-10.03) | 5.16(5.02-5.30) | 2.26(2.19-2.34) | 0.76(0.72-0.81) | 0.21(0.19-0.23) | 0.02(0.02-0.03) |
| Myanmar | 45.78(45.41-46.14) | 39.87(39.53-40.21) | 25.38(25.07-25.68) | 16.12(15.87-16.37) | 8.86(8.69-9.03) | 4.28(4.19-4.36) | 1.55(1.52-1.59) | 0.53(0.51-0.55) | 0.11(0.10-0.12) |
| Namibia | 37.11(35.41-38.82) | 32.32(30.72-33.91) | 20.41(19.04-21.78) | 12.76(11.79-13.73) | 6.63(6.10-7.15) | 2.81(2.55-3.07) | 0.83(0.70-0.96) | 0.22(0.16-0.28) | 0.02(0.01-0.03) |
| Nauru | 42.19(16.88-67.49) | 36.85(12.61-61.09) | 23.08(1.45-44.71) | 14.34(-1.90-30.57) | 7.57(-2.34-17.49) | 3.35(-1.77-8.48) | 1.05(-1.39-3.49) | 0.33(-1.06-1.72) | 0.06(-0.56-0.68) |
| Nepal | 47.56(47.08-48.04) | 40.41(39.94-40.87) | 25.73(25.30-26.17) | 16.43(16.06-16.81) | 9.03(8.77-9.30) | 4.35(4.22-4.48) | 1.69(1.64-1.74) | 0.58(0.55-0.61) | 0.11(0.10-0.12) |
| Netherlands | 46.51(45.94-47.08) | 39.76(39.20-40.33) | 25.44(24.90-25.98) | 16.53(16.05-17.02) | 8.94(8.53-9.35) | 4.32(4.06-4.57) | 2.01(1.92-2.09) | 1.08(1.03-1.12) | 0.39(0.36-0.42) |
| New Zealand | 44.28(43.10-45.47) | 39.33(38.16-40.50) | 25.59(24.48-26.71) | 17.00(16.00-18.00) | 9.70(8.86-10.55) | 4.99(4.45-5.53) | 2.36(2.16-2.57) | 1.31(1.18-1.43) | 0.49(0.40-0.57) |
| Nicaragua | 37.74(36.64-38.83) | 30.46(29.38-31.54) | 18.58(17.56-19.59) | 12.63(11.79-13.47) | 7.71(7.11-8.31) | 4.08(3.76-4.40) | 1.79(1.64-1.94) | 0.76(0.66-0.86) | 0.21(0.16-0.27) |
| Niger | 40.57(39.96-41.17) | 34.58(33.99-35.16) | 21.67(21.12-22.22) | 14.09(13.62-14.56) | 7.82(7.48-8.15) | 3.73(3.56-3.90) | 1.40(1.33-1.48) | 0.53(0.48-0.58) | 0.10(0.08-0.12) |
| Nigeria | 41.70(41.52-41.89) | 36.02(35.84-36.19) | 22.62(22.45-22.78) | 14.75(14.61-14.89) | 8.25(8.15-8.35) | 4.01(3.95-4.06) | 1.58(1.56-1.61) | 0.63(0.62-0.65) | 0.13(0.12-0.14) |
| Niue | 43.00(-43.25-129.26) | 37.80(-41.83-117.44) | 24.43(-48.80-97.66) | 15.84(-45.33-77.00) | 8.99(-34.42-52.39) | 4.46(-16.88-25.80) | 1.69(-6.00-9.38) | 0.64(-3.63-4.92) | 0.14(-1.56-1.85) |
| North Africa and Middle East | 44.17(44.06-44.28) | 35.36(35.25-35.46) | 21.86(21.76-21.96) | 14.96(14.88-15.04) | 9.17(9.11-9.23) | 5.02(4.99-5.05) | 2.34(2.32-2.35) | 0.93(0.92-0.95) | 0.20(0.20-0.21) |
| North Macedonia | 39.69(37.80-41.58) | 34.88(33.05-36.72) | 23.24(21.57-24.91) | 16.09(14.71-17.46) | 9.55(8.60-10.50) | 4.79(4.36-5.22) | 1.94(1.78-2.10) | 0.72(0.62-0.81) | 0.12(0.08-0.16) |
| Northern Mariana Islands | 45.60(33.15-58.05) | 40.15(27.98-52.32) | 26.02(14.66-37.39) | 16.90(8.02-25.79) | 9.63(3.87-15.39) | 4.82(2.10-7.54) | 1.87(0.50-3.25) | 0.74(-0.24-1.73) | 0.18(-0.36-0.72) |
| Norway | 48.82(47.84-49.80) | 41.74(40.77-42.71) | 26.04(25.12-26.97) | 17.01(16.18-17.83) | 9.31(8.59-10.03) | 4.58(4.08-5.08) | 2.17(1.99-2.36) | 1.20(1.10-1.31) | 0.47(0.40-0.54) |
| Oceania | 42.37(41.65-43.10) | 37.19(36.50-37.87) | 23.80(23.17-24.43) | 15.21(14.70-15.72) | 8.42(8.09-8.76) | 4.01(3.84-4.18) | 1.40(1.32-1.48) | 0.47(0.42-0.52) | 0.09(0.07-0.11) |
| Oman | 44.90(43.78-46.02) | 36.15(35.04-37.25) | 22.51(21.53-23.49) | 15.38(14.57-16.19) | 9.42(8.73-10.12) | 5.15(4.67-5.64) | 2.37(2.10-2.65) | 0.92(0.73-1.10) | 0.17(0.10-0.25) |
| Pakistan | 45.28(45.11-45.45) | 39.21(39.05-39.37) | 25.14(24.99-25.29) | 16.12(15.99-16.25) | 8.92(8.83-9.01) | 4.33(4.29-4.38) | 1.69(1.67-1.72) | 0.60(0.58-0.61) | 0.12(0.11-0.12) |
| Palau | 44.52(24.83-64.22) | 39.12(20.28-57.97) | 25.15(8.08-42.21) | 16.15(3.80-28.50) | 9.11(1.70-16.52) | 4.56(0.68-8.43) | 1.82(-0.27-3.90) | 0.79(-0.80-2.37) | 0.23(-0.84-1.30) |
| Palestine | 44.98(43.69-46.28) | 36.25(34.98-37.53) | 22.66(21.45-23.87) | 15.56(14.47-16.65) | 9.62(8.74-10.51) | 5.34(4.82-5.87) | 2.54(2.28-2.79) | 1.04(0.87-1.22) | 0.22(0.13-0.31) |
| Panama | 38.45(36.92-39.97) | 31.17(29.67-32.67) | 19.29(17.88-20.69) | 13.36(12.17-14.54) | 8.43(7.55-9.32) | 4.75(4.23-5.26) | 2.33(2.10-2.56) | 1.15(1.00-1.30) | 0.40(0.30-0.50) |
| Papua New Guinea | 42.03(41.20-42.86) | 36.88(36.09-37.67) | 23.60(22.88-24.32) | 15.08(14.49-15.66) | 8.34(7.95-8.74) | 3.96(3.76-4.16) | 1.37(1.27-1.46) | 0.45(0.39-0.51) | 0.08(0.06-0.11) |
| Paraguay | 46.01(45.07-46.94) | 33.41(32.49-34.34) | 18.04(17.18-18.89) | 12.28(11.57-12.99) | 7.22(6.71-7.73) | 3.67(3.42-3.92) | 1.50(1.39-1.61) | 0.62(0.55-0.70) | 0.15(0.12-0.19) |
| Peru | 28.94(28.42-29.45) | 24.35(23.86-24.85) | 15.71(15.26-16.16) | 10.80(10.44-11.15) | 6.53(6.31-6.75) | 3.45(3.34-3.56) | 1.49(1.44-1.54) | 0.60(0.57-0.63) | 0.15(0.14-0.17) |
| Philippines | 48.12(47.88-48.36) | 42.36(42.13-42.59) | 26.81(26.60-27.02) | 16.94(16.77-17.11) | 9.25(9.14-9.37) | 4.39(4.33-4.45) | 1.55(1.53-1.58) | 0.51(0.50-0.53) | 0.10(0.09-0.11) |
| Poland | 41.23(40.75-41.71) | 36.61(36.14-37.08) | 24.48(24.04-24.92) | 17.02(16.67-17.36) | 10.28(10.03-10.52) | 5.36(5.23-5.49) | 2.36(2.32-2.41) | 1.03(1.00-1.06) | 0.26(0.25-0.28) |
| Portugal | 47.24(46.50-47.99) | 40.15(39.43-40.88) | 25.51(24.82-26.19) | 16.44(15.85-17.04) | 8.79(8.32-9.25) | 4.16(3.90-4.42) | 1.88(1.80-1.97) | 1.00(0.95-1.06) | 0.38(0.35-0.42) |
| Puerto Rico | 41.31(39.56-43.05) | 33.78(32.05-35.51) | 20.59(18.95-22.22) | 14.17(12.86-15.47) | 8.63(7.74-9.53) | 4.74(4.27-5.21) | 2.27(2.09-2.44) | 1.17(1.05-1.28) | 0.43(0.35-0.50) |
| Qatar | 46.15(44.56-47.75) | 37.35(35.78-38.92) | 23.64(22.33-24.95) | 16.46(15.26-17.66) | 10.43(9.28-11.57) | 6.04(5.06-7.02) | 3.08(2.39-3.77) | 1.39(0.87-1.91) | 0.34(0.04-0.65) |
| Republic of Korea | 35.31(34.92-35.70) | 30.82(30.44-31.20) | 20.03(19.67-20.39) | 12.90(12.59-13.22) | 7.57(7.32-7.81) | 4.18(4.05-4.32) | 2.22(2.16-2.27) | 1.22(1.18-1.27) | 0.45(0.42-0.48) |
| Republic of Moldova | 35.63(33.99-37.26) | 31.45(29.89-33.01) | 21.20(19.81-22.59) | 14.39(13.37-15.40) | 8.61(7.97-9.25) | 4.53(4.24-4.82) | 1.95(1.82-2.08) | 0.77(0.68-0.85) | 0.20(0.16-0.24) |
| Romania | 39.55(38.84-40.26) | 34.75(34.06-35.44) | 23.13(22.49-23.76) | 16.00(15.51-16.49) | 9.49(9.18-9.79) | 4.76(4.63-4.89) | 1.96(1.91-2.01) | 0.79(0.76-0.82) | 0.18(0.17-0.19) |
| Russian Federation | 38.58(38.33-38.83) | 33.98(33.74-34.23) | 22.19(21.96-22.41) | 14.73(14.59-14.88) | 8.54(8.46-8.63) | 4.34(4.30-4.38) | 1.77(1.76-1.79) | 0.65(0.64-0.67) | 0.13(0.13-0.14) |
| Rwanda | 29.75(28.90-30.60) | 26.45(25.64-27.26) | 18.04(17.31-18.77) | 11.79(11.22-12.36) | 6.78(6.42-7.15) | 3.40(3.21-3.58) | 1.42(1.31-1.52) | 0.51(0.44-0.58) | 0.08(0.06-0.11) |
| Saint Kitts and Nevis | 40.11(28.28-51.94) | 32.66(21.33-43.99) | 19.63(9.49-29.78) | 13.30(5.37-21.22) | 7.80(2.55-13.04) | 3.94(1.58-6.30) | 1.57(0.47-2.66) | 0.61(-0.19-1.42) | 0.13(-0.27-0.54) |
| Saint Lucia | 40.15(33.29-47.01) | 32.70(26.06-39.34) | 19.67(13.65-25.69) | 13.34(8.65-18.03) | 7.89(4.84-10.94) | 4.09(2.61-5.56) | 1.72(1.09-2.36) | 0.74(0.32-1.16) | 0.19(-0.02-0.40) |
| Saint Vincent and the Grenadines | 40.23(31.70-48.76) | 32.75(24.37-41.14) | 19.68(12.00-27.35) | 13.32(7.30-19.35) | 7.86(4.09-11.63) | 4.06(2.27-5.86) | 1.73(0.93-2.53) | 0.76(0.22-1.29) | 0.20(-0.08-0.47) |
| Samoa | 45.22(38.94-51.50) | 39.81(33.65-45.97) | 25.80(19.97-31.64) | 16.78(11.87-21.69) | 9.60(6.09-13.11) | 4.86(3.00-6.72) | 1.94(1.09-2.79) | 0.80(0.23-1.38) | 0.21(-0.11-0.53) |
| San Marino | 47.80(34.31-61.30) | 40.70(27.37-54.03) | 26.02(13.21-38.83) | 16.94(5.58-28.30) | 9.25(-0.25-18.75) | 4.59(-1.79-10.97) | 2.25(-0.34-4.84) | 1.31(-0.07-2.68) | 0.58(-0.34-1.51) |
| Sao Tome and Principe | 45.36(39.41-51.30) | 38.82(33.01-44.62) | 24.66(19.17-30.14) | 16.28(11.57-20.99) | 9.26(5.67-12.85) | 4.59(2.55-6.62) | 1.83(0.90-2.76) | 0.75(0.11-1.38) | 0.15(-0.12-0.43) |
| Saudi Arabia | 45.76(45.38-46.14) | 36.60(36.22-36.98) | 22.65(22.31-22.99) | 15.52(15.24-15.79) | 9.56(9.35-9.78) | 5.32(5.16-5.47) | 2.57(2.47-2.67) | 1.12(1.04-1.19) | 0.29(0.24-0.34) |
| Senegal | 43.34(42.63-44.05) | 36.98(36.30-37.67) | 23.24(22.60-23.88) | 15.13(14.58-15.68) | 8.40(8.02-8.79) | 4.00(3.80-4.19) | 1.49(1.40-1.57) | 0.55(0.50-0.60) | 0.10(0.08-0.12) |
| Serbia | 40.25(39.36-41.14) | 35.44(34.57-36.31) | 23.78(22.95-24.60) | 16.60(15.88-17.31) | 10.00(9.46-10.54) | 5.16(4.89-5.42) | 2.21(2.12-2.29) | 0.90(0.84-0.95) | 0.21(0.18-0.23) |
| Seychelles | 48.61(42.01-55.20) | 42.51(35.96-49.05) | 27.43(20.93-33.93) | 17.67(12.29-23.06) | 9.96(5.98-13.93) | 5.04(2.75-7.33) | 2.03(1.01-3.06) | 0.81(0.15-1.46) | 0.20(-0.15-0.55) |
| Sierra Leone | 39.96(39.06-40.85) | 34.04(33.20-34.89) | 21.31(20.53-22.08) | 13.83(13.19-14.47) | 7.65(7.21-8.10) | 3.65(3.42-3.89) | 1.39(1.27-1.50) | 0.54(0.47-0.61) | 0.11(0.07-0.14) |
| Singapore | 31.95(30.71-33.19) | 28.38(27.16-29.60) | 19.15(18.03-20.28) | 12.61(11.67-13.54) | 7.61(6.80-8.41) | 4.37(3.80-4.94) | 2.43(2.19-2.67) | 1.41(1.24-1.59) | 0.59(0.46-0.72) |
| Slovakia | 40.01(38.75-41.27) | 35.21(33.99-36.43) | 23.57(22.44-24.70) | 16.42(15.52-17.32) | 9.88(9.24-10.52) | 5.10(4.78-5.42) | 2.20(2.08-2.31) | 0.92(0.84-0.99) | 0.22(0.18-0.26) |
| Slovenia | 41.25(39.25-43.25) | 36.43(34.44-38.42) | 24.73(22.88-26.59) | 17.53(15.98-19.09) | 10.92(9.68-12.16) | 6.02(5.27-6.77) | 2.95(2.68-3.21) | 1.45(1.28-1.62) | 0.45(0.35-0.55) |
| Solomon Islands | 43.50(40.19-46.80) | 38.13(34.93-41.33) | 24.26(21.32-27.21) | 15.40(13.10-17.70) | 8.46(7.02-9.90) | 4.01(3.25-4.78) | 1.43(1.04-1.82) | 0.52(0.27-0.77) | 0.11(-0.01-0.24) |
| Somalia | 25.78(25.22-26.33) | 22.69(22.17-23.21) | 14.89(14.46-15.33) | 9.22(8.92-9.53) | 4.82(4.66-4.98) | 2.03(1.94-2.13) | 0.62(0.57-0.67) | 0.14(0.12-0.16) | 0.01(0.01-0.02) |
| South Africa | 38.14(37.79-38.48) | 33.55(33.23-33.87) | 21.10(20.84-21.36) | 13.17(13.00-13.34) | 6.96(6.86-7.06) | 3.14(3.09-3.20) | 1.10(1.07-1.12) | 0.37(0.36-0.39) | 0.06(0.05-0.06) |
| South Asia | 45.99(45.93-46.05) | 39.97(39.92-40.03) | 25.85(25.80-25.90) | 16.66(16.62-16.70) | 9.26(9.23-9.30) | 4.57(4.56-4.59) | 1.85(1.84-1.85) | 0.67(0.67-0.68) | 0.15(0.14-0.15) |
| South Sudan | 25.37(24.46-26.27) | 22.44(21.59-23.29) | 15.03(14.27-15.79) | 9.60(9.06-10.13) | 5.32(5.05-5.60) | 2.52(2.38-2.66) | 0.96(0.88-1.05) | 0.31(0.26-0.36) | 0.04(0.03-0.06) |
| Southeast Asia | 48.21(48.11-48.31) | 42.34(42.24-42.43) | 27.06(26.98-27.15) | 17.33(17.26-17.40) | 9.68(9.63-9.73) | 4.79(4.76-4.82) | 1.84(1.83-1.85) | 0.69(0.68-0.70) | 0.17(0.16-0.17) |
| Southern Latin America | 35.59(35.21-35.96) | 31.67(31.30-32.04) | 21.02(20.68-21.37) | 13.80(13.52-14.09) | 7.89(7.68-8.10) | 4.00(3.89-4.10) | 1.77(1.73-1.81) | 0.84(0.82-0.87) | 0.27(0.25-0.29) |
| Southern Sub-Saharan Africa | 37.29(37.00-37.58) | 32.66(32.40-32.93) | 20.51(20.29-20.73) | 12.75(12.61-12.89) | 6.65(6.57-6.73) | 2.91(2.87-2.96) | 0.96(0.94-0.98) | 0.30(0.29-0.31) | 0.04(0.04-0.05) |
| Spain | 48.22(47.89-48.56) | 40.76(40.43-41.09) | 25.78(25.47-26.09) | 16.67(16.40-16.94) | 8.95(8.73-9.17) | 4.27(4.13-4.41) | 1.96(1.92-2.01) | 1.07(1.04-1.10) | 0.42(0.40-0.44) |
| Sri Lanka | 49.09(48.51-49.67) | 42.94(42.38-43.51) | 27.79(27.26-28.33) | 18.04(17.60-18.49) | 10.33(10.00-10.67) | 5.37(5.18-5.57) | 2.28(2.20-2.36) | 0.98(0.93-1.03) | 0.28(0.25-0.32) |
| Sudan | 42.77(42.34-43.20) | 34.30(33.88-34.72) | 21.15(20.75-21.54) | 14.30(13.96-14.64) | 8.64(8.39-8.88) | 4.64(4.50-4.77) | 2.10(2.03-2.17) | 0.81(0.77-0.85) | 0.16(0.15-0.18) |
| Suriname | 39.35(35.62-43.09) | 31.99(28.35-35.63) | 19.11(15.76-22.47) | 12.87(10.28-15.45) | 7.51(5.88-9.14) | 3.80(3.08-4.53) | 1.55(1.20-1.91) | 0.66(0.41-0.91) | 0.18(0.05-0.31) |
| Sweden | 47.74(46.98-48.49) | 41.34(40.59-42.09) | 26.49(25.77-27.21) | 17.30(16.67-17.92) | 9.46(8.91-10.01) | 4.67(4.28-5.06) | 2.23(2.09-2.36) | 1.24(1.17-1.31) | 0.49(0.44-0.54) |
| Switzerland | 45.48(44.70-46.27) | 39.20(38.42-39.97) | 25.49(24.76-26.22) | 16.68(16.03-17.33) | 9.15(8.58-9.72) | 4.54(4.15-4.94) | 2.21(2.06-2.35) | 1.26(1.17-1.34) | 0.52(0.46-0.58) |
| Syrian Arab Republic | 44.50(43.58-45.43) | 35.77(34.86-36.67) | 22.19(21.32-23.06) | 15.14(14.49-15.79) | 9.29(8.90-9.67) | 5.09(4.90-5.29) | 2.39(2.29-2.48) | 0.98(0.92-1.04) | 0.22(0.19-0.25) |
| Taiwan (Province of China) | 41.90(41.33-42.48) | 37.26(36.70-37.83) | 25.37(24.86-25.89) | 17.19(16.75-17.63) | 10.41(10.07-10.76) | 5.82(5.62-6.02) | 2.77(2.68-2.85) | 1.35(1.30-1.41) | 0.48(0.45-0.52) |
| Tajikistan | 39.01(38.09-39.93) | 34.37(33.48-35.26) | 22.83(22.00-23.66) | 15.48(14.77-16.19) | 9.13(8.60-9.66) | 4.66(4.38-4.94) | 1.93(1.80-2.07) | 0.77(0.67-0.87) | 0.17(0.13-0.22) |
| Thailand | 49.08(48.71-49.45) | 42.73(42.37-43.08) | 27.23(26.92-27.54) | 17.51(17.28-17.75) | 9.92(9.77-10.08) | 5.15(5.07-5.24) | 2.23(2.19-2.27) | 1.02(0.99-1.04) | 0.34(0.32-0.36) |
| Timor-Leste | 46.60(44.20-48.99) | 40.66(38.35-42.98) | 26.08(23.89-28.27) | 16.72(14.78-18.65) | 9.33(7.92-10.75) | 4.63(3.87-5.39) | 1.77(1.48-2.06) | 0.65(0.48-0.83) | 0.15(0.06-0.24) |
| Togo | 41.84(40.90-42.78) | 35.61(34.71-36.51) | 22.15(21.33-22.98) | 14.26(13.59-14.92) | 7.75(7.33-8.17) | 3.57(3.35-3.78) | 1.26(1.14-1.37) | 0.44(0.37-0.51) | 0.07(0.04-0.10) |
| Tokelau | 43.11(-55.88-142.09) | 37.95(-56.64-132.53) | 24.67(-63.81-113.14) | 16.15(-61.58-93.87) | 9.35(-53.20-71.90) | 4.82(-35.46-45.11) | 1.99(-13.80-17.78) | 0.85(-6.02-7.72) | 0.23(-2.83-3.30) |
| Tonga | 45.60(36.44-54.75) | 40.18(31.18-49.17) | 26.15(17.53-34.77) | 17.08(9.64-24.52) | 9.82(4.46-15.19) | 5.00(2.16-7.84) | 2.01(0.79-3.23) | 0.83(0.07-1.60) | 0.22(-0.17-0.61) |
| Trinidad and Tobago | 39.71(37.22-42.20) | 32.24(29.81-34.67) | 19.18(17.02-21.34) | 12.88(11.35-14.42) | 7.52(6.52-8.52) | 3.81(3.34-4.28) | 1.55(1.36-1.74) | 0.64(0.51-0.77) | 0.16(0.09-0.23) |
| Tropical Latin America | 47.82(47.65-47.99) | 34.56(34.40-34.73) | 18.37(18.22-18.52) | 12.52(12.40-12.63) | 7.46(7.38-7.53) | 3.87(3.83-3.91) | 1.68(1.66-1.70) | 0.77(0.76-0.78) | 0.23(0.22-0.24) |
| Tunisia | 44.84(44.05-45.64) | 36.10(35.32-36.88) | 22.48(21.75-23.21) | 15.36(14.77-15.96) | 9.43(9.00-9.86) | 5.19(4.96-5.43) | 2.45(2.35-2.55) | 1.00(0.94-1.06) | 0.21(0.18-0.24) |
| Turkey | 43.38(43.08-43.68) | 35.43(35.14-35.73) | 22.63(22.36-22.90) | 15.65(15.42-15.89) | 9.75(9.57-9.92) | 5.45(5.35-5.55) | 2.62(2.57-2.66) | 1.10(1.07-1.13) | 0.25(0.24-0.27) |
| Turkmenistan | 38.65(37.43-39.87) | 33.97(32.79-35.14) | 22.31(21.24-23.38) | 14.93(14.09-15.76) | 8.59(8.04-9.14) | 4.20(3.93-4.47) | 1.62(1.49-1.76) | 0.59(0.50-0.69) | 0.12(0.08-0.15) |
| Tuvalu | 44.06(19.12-69.00) | 38.69(14.67-62.71) | 24.81(2.65-46.96) | 15.90(-1.98-33.79) | 8.89(-3.37-21.15) | 4.34(-1.95-10.63) | 1.62(-1.09-4.33) | 0.63(-1.12-2.37) | 0.15(-0.79-1.09) |
| Uganda | 28.19(27.74-28.65) | 24.98(24.55-25.42) | 16.84(16.45-17.22) | 10.81(10.51-11.11) | 6.04(5.87-6.22) | 2.90(2.80-2.99) | 1.13(1.08-1.19) | 0.38(0.34-0.41) | 0.05(0.04-0.06) |
| Ukraine | 36.28(35.79-36.76) | 32.33(31.86-32.80) | 21.59(21.17-22.01) | 14.50(14.22-14.77) | 8.55(8.38-8.71) | 4.45(4.37-4.53) | 1.89(1.85-1.93) | 0.73(0.71-0.75) | 0.16(0.15-0.17) |
| United Arab Emirates | 46.28(45.27-47.29) | 37.48(36.49-38.48) | 23.77(22.86-24.68) | 16.61(16.00-17.23) | 10.61(10.03-11.18) | 6.24(5.71-6.76) | 3.28(2.85-3.70) | 1.57(1.20-1.94) | 0.46(0.18-0.74) |
| United Kingdom | 46.82(46.52-47.13) | 40.51(40.21-40.82) | 25.80(25.52-26.09) | 16.68(16.43-16.93) | 8.97(8.77-9.18) | 4.27(4.16-4.38) | 1.94(1.90-1.98) | 1.02(1.00-1.04) | 0.37(0.36-0.39) |
| United Republic of Tanzania | 28.43(28.01-28.84) | 25.32(24.93-25.71) | 17.33(16.98-17.68) | 11.34(11.07-11.61) | 6.53(6.37-6.69) | 3.29(3.20-3.37) | 1.40(1.35-1.45) | 0.52(0.49-0.55) | 0.09(0.08-0.10) |
| United States Virgin Islands | 40.18(29.02-51.33) | 32.65(21.52-43.78) | 19.47(9.53-29.41) | 13.20(5.68-20.72) | 7.95(2.90-12.99) | 4.28(1.63-6.93) | 1.96(1.08-2.85) | 0.95(0.37-1.53) | 0.30(-0.09-0.69) |
| United States of America | 42.07(41.91-42.24) | 37.31(37.15-37.47) | 23.20(23.05-23.35) | 14.44(14.32-14.56) | 7.66(7.58-7.74) | 3.75(3.71-3.79) | 1.60(1.59-1.62) | 0.81(0.80-0.82) | 0.28(0.28-0.29) |
| Uruguay | 34.80(33.00-36.60) | 31.04(29.28-32.80) | 20.60(18.98-22.22) | 13.49(12.19-14.78) | 7.63(6.74-8.51) | 3.81(3.40-4.22) | 1.63(1.49-1.78) | 0.75(0.65-0.84) | 0.22(0.17-0.28) |
| Uzbekistan | 40.09(39.58-40.60) | 35.38(34.89-35.87) | 23.64(23.18-24.09) | 16.16(15.77-16.54) | 9.66(9.37-9.96) | 5.05(4.88-5.21) | 2.19(2.11-2.26) | 0.93(0.87-0.98) | 0.22(0.20-0.25) |
| Vanuatu | 43.26(38.39-48.14) | 37.90(33.20-42.61) | 24.06(19.76-28.35) | 15.20(11.86-18.55) | 8.28(6.24-10.33) | 3.86(2.83-4.90) | 1.32(0.82-1.82) | 0.45(0.14-0.76) | 0.08(-0.06-0.23) |
| Venezuela (Bolivarian Republic of) | 36.21(35.64-36.78) | 29.03(28.48-29.59) | 17.25(16.76-17.73) | 11.56(11.22-11.91) | 6.95(6.73-7.17) | 3.60(3.49-3.70) | 1.51(1.46-1.56) | 0.60(0.57-0.63) | 0.14(0.13-0.16) |
| Viet Nam | 48.35(48.10-48.61) | 42.25(42.00-42.50) | 27.21(26.98-27.43) | 17.53(17.35-17.72) | 9.87(9.72-10.01) | 4.95(4.87-5.02) | 1.94(1.90-1.97) | 0.74(0.71-0.76) | 0.17(0.16-0.19) |
| Western Europe | 48.03(47.92-48.15) | 40.95(40.84-41.06) | 25.95(25.85-26.06) | 16.76(16.66-16.85) | 8.98(8.90-9.05) | 4.28(4.23-4.32) | 1.96(1.94-1.97) | 1.05(1.04-1.06) | 0.40(0.39-0.41) |
| Western Sub-Saharan Africa | 41.22(41.10-41.35) | 35.33(35.21-35.45) | 22.09(21.98-22.20) | 14.32(14.23-14.41) | 7.91(7.85-7.97) | 3.75(3.72-3.78) | 1.41(1.39-1.42) | 0.53(0.52-0.54) | 0.10(0.09-0.10) |
| Yemen | 41.49(41.01-41.97) | 33.03(32.57-33.50) | 19.95(19.52-20.37) | 13.33(12.98-13.67) | 7.91(7.66-8.15) | 4.11(3.97-4.25) | 1.76(1.69-1.82) | 0.62(0.58-0.66) | 0.11(0.09-0.13) |
| Zambia | 30.27(29.65-30.90) | 26.43(25.85-27.02) | 17.24(16.74-17.74) | 10.75(10.40-11.10) | 5.70(5.51-5.89) | 2.51(2.41-2.61) | 0.84(0.79-0.90) | 0.23(0.20-0.25) | 0.02(0.02-0.03) |
| Zimbabwe | 35.27(34.62-35.93) | 30.58(29.97-31.19) | 18.99(18.47-19.51) | 11.60(11.25-11.95) | 5.78(5.60-5.95) | 2.27(2.18-2.36) | 0.58(0.54-0.62) | 0.12(0.11-0.14) | 0.01(0.01-0.01) |

Table S3. Age-conditional probability (%) of developing migraine, females, 2021.

| Region | Birth to death | 10 years to death | 20 years to death | 30 years to death | 40 years to death | 50 years to death | 60 years to death | 70 years to death | 80 years to death |
| --- | --- | --- | --- | --- | --- | --- | --- | --- | --- |
| Global | 59.84(59.81-59.86) | 50.50(50.48-50.53) | 31.65(31.62-31.67) | 20.30(20.28-20.32) | 11.27(11.26-11.29) | 5.63(5.62-5.64) | 2.39(2.38-2.39) | 1.11(1.10-1.11) | 0.36(0.36-0.36) |
| Afghanistan | 57.50(57.08-57.91) | 46.94(46.54-47.34) | 28.67(28.31-29.03) | 16.58(16.27-16.88) | 7.94(7.73-8.15) | 3.36(3.26-3.47) | 1.08(1.03-1.14) | 0.35(0.32-0.38) | 0.05(0.05-0.06) |
| Albania | 61.85(60.78-62.92) | 53.37(52.33-54.41) | 34.49(33.50-35.48) | 22.35(21.42-23.28) | 12.31(11.48-13.13) | 6.01(5.41-6.61) | 2.54(2.27-2.80) | 1.17(1.05-1.29) | 0.34(0.27-0.40) |
| Algeria | 62.86(62.58-63.14) | 51.91(51.63-52.18) | 32.81(32.55-33.07) | 19.86(19.62-20.10) | 10.36(10.15-10.57) | 5.09(4.93-5.24) | 2.19(2.12-2.26) | 1.03(0.99-1.07) | 0.27(0.25-0.29) |
| American Samoa | 60.42(50.79-70.06) | 51.80(42.36-61.24) | 32.64(23.60-41.69) | 20.99(13.13-28.85) | 11.80(5.69-17.90) | 5.93(2.15-9.70) | 2.35(0.65-4.06) | 1.00(-0.02-2.01) | 0.26(-0.29-0.81) |
| Andean Latin America | 53.88(53.59-54.16) | 42.86(42.58-43.14) | 27.35(27.08-27.61) | 18.81(18.58-19.04) | 10.45(10.26-10.65) | 4.93(4.80-5.05) | 2.11(2.06-2.15) | 0.93(0.90-0.96) | 0.29(0.27-0.30) |
| Andorra | 69.81(65.62-74.01) | 57.77(53.68-61.86) | 31.35(27.42-35.28) | 18.57(14.90-22.25) | 9.96(6.58-13.33) | 4.50(1.64-7.37) | 1.95(0.30-3.60) | 1.11(0.32-1.89) | 0.52(0.02-1.01) |
| Angola | 50.83(50.37-51.29) | 43.59(43.14-44.03) | 27.49(27.07-27.90) | 17.35(17.00-17.70) | 9.46(9.20-9.72) | 4.43(4.28-4.58) | 1.58(1.52-1.65) | 0.63(0.59-0.68) | 0.14(0.11-0.16) |
| Antigua and Barbuda | 62.97(57.41-68.53) | 48.50(43.06-53.93) | 29.02(23.91-34.13) | 19.59(14.97-24.21) | 10.74(6.72-14.76) | 5.08(2.29-7.88) | 2.15(0.96-3.34) | 1.01(0.31-1.70) | 0.31(-0.10-0.72) |
| Argentina | 56.12(55.81-56.42) | 49.58(49.28-49.88) | 30.80(30.51-31.09) | 19.40(19.13-19.67) | 10.88(10.65-11.11) | 5.29(5.12-5.46) | 2.21(2.15-2.27) | 1.10(1.07-1.13) | 0.42(0.40-0.44) |
| Armenia | 61.53(60.54-62.51) | 52.93(51.97-53.88) | 33.75(32.84-34.66) | 21.86(21.02-22.71) | 12.14(11.36-12.92) | 5.94(5.36-6.52) | 2.49(2.27-2.71) | 1.16(1.04-1.27) | 0.35(0.29-0.40) |
| Australasia | 60.70(60.38-61.02) | 53.52(53.21-53.84) | 32.67(32.37-32.97) | 20.58(20.29-20.86) | 11.50(11.24-11.76) | 5.63(5.42-5.84) | 2.49(2.38-2.60) | 1.38(1.33-1.43) | 0.60(0.57-0.63) |
| Australia | 60.50(60.15-60.85) | 53.27(52.93-53.62) | 32.58(32.25-32.91) | 20.54(20.23-20.86) | 11.49(11.20-11.78) | 5.63(5.39-5.86) | 2.50(2.38-2.62) | 1.39(1.34-1.45) | 0.61(0.57-0.64) |
| Austria | 68.59(68.17-69.01) | 57.44(57.03-57.85) | 31.70(31.31-32.10) | 18.76(18.39-19.14) | 10.05(9.71-10.40) | 4.52(4.24-4.80) | 1.92(1.79-2.05) | 1.06(1.01-1.12) | 0.48(0.44-0.51) |
| Azerbaijan | 59.93(59.35-60.51) | 51.47(50.92-52.01) | 32.60(32.10-33.11) | 20.94(20.48-21.40) | 11.42(11.02-11.83) | 5.40(5.13-5.66) | 2.09(1.98-2.19) | 0.86(0.79-0.93) | 0.22(0.19-0.24) |
| Bahamas | 62.21(59.16-65.25) | 47.73(44.73-50.72) | 28.24(25.40-31.08) | 18.85(16.43-21.26) | 10.13(8.29-11.98) | 4.67(3.54-5.79) | 1.90(1.42-2.38) | 0.86(0.55-1.16) | 0.25(0.08-0.42) |
| Bahrain | 63.52(61.92-65.13) | 52.45(50.88-54.03) | 33.15(31.65-34.65) | 20.04(18.65-21.44) | 10.41(9.14-11.67) | 5.03(4.08-5.99) | 2.07(1.62-2.51) | 0.89(0.59-1.19) | 0.20(0.06-0.35) |
| Bangladesh | 59.79(59.63-59.95) | 49.22(49.07-49.38) | 31.00(30.86-31.14) | 20.22(20.09-20.35) | 11.25(11.13-11.36) | 5.50(5.42-5.58) | 2.32(2.29-2.36) | 1.02(1.00-1.04) | 0.28(0.27-0.30) |
| Barbados | 62.85(59.52-66.18) | 48.40(45.15-51.65) | 28.96(25.92-31.99) | 19.55(16.91-22.19) | 10.74(8.55-12.93) | 5.13(3.68-6.58) | 2.21(1.67-2.76) | 1.07(0.76-1.37) | 0.35(0.16-0.53) |
| Belarus | 59.67(59.03-60.31) | 51.63(51.01-52.26) | 33.26(32.67-33.85) | 21.32(20.81-21.84) | 11.48(11.05-11.91) | 5.59(5.32-5.86) | 2.28(2.19-2.37) | 0.97(0.93-1.02) | 0.28(0.26-0.30) |
| Belgium | 72.12(71.77-72.47) | 57.74(57.40-58.08) | 29.02(28.68-29.35) | 16.81(16.50-17.13) | 8.93(8.65-9.22) | 3.95(3.73-4.18) | 1.70(1.60-1.79) | 0.96(0.92-1.01) | 0.45(0.42-0.48) |
| Belize | 62.46(59.55-65.37) | 48.03(45.14-50.92) | 28.62(25.84-31.40) | 19.23(16.69-21.77) | 10.48(8.30-12.65) | 4.93(3.43-6.43) | 2.08(1.40-2.77) | 1.00(0.53-1.47) | 0.33(0.04-0.61) |
| Benin | 54.54(53.87-55.20) | 45.33(44.69-45.97) | 27.80(27.19-28.41) | 17.77(17.22-18.32) | 9.87(9.42-10.33) | 4.75(4.45-5.05) | 1.79(1.67-1.90) | 0.79(0.71-0.86) | 0.20(0.16-0.23) |
| Bermuda | 63.97(57.24-70.70) | 49.41(42.79-56.03) | 29.81(23.51-36.10) | 20.30(14.72-25.88) | 11.37(6.45-16.28) | 5.63(1.93-9.33) | 2.61(0.80-4.42) | 1.38(0.52-2.23) | 0.52(0.04-0.99) |
| Bhutan | 59.94(57.54-62.34) | 49.39(47.08-51.69) | 31.15(28.96-33.33) | 20.35(18.34-22.36) | 11.35(9.54-13.17) | 5.59(4.24-6.93) | 2.38(1.80-2.95) | 1.05(0.75-1.36) | 0.30(0.14-0.47) |
| Bolivia (Plurinational State of) | 53.21(52.54-53.88) | 42.04(41.38-42.70) | 26.47(25.86-27.08) | 18.01(17.48-18.54) | 9.72(9.31-10.13) | 4.32(4.09-4.55) | 1.64(1.56-1.72) | 0.61(0.56-0.66) | 0.14(0.11-0.16) |
| Bosnia and Herzegovina | 62.17(61.24-63.09) | 53.63(52.73-54.53) | 34.61(33.75-35.46) | 22.38(21.59-23.16) | 12.26(11.57-12.95) | 5.91(5.40-6.41) | 2.43(2.24-2.61) | 1.09(1.00-1.17) | 0.31(0.27-0.35) |
| Botswana | 51.06(49.58-52.54) | 43.72(42.30-45.13) | 27.37(26.09-28.65) | 17.09(16.10-18.07) | 9.15(8.47-9.83) | 4.19(3.80-4.59) | 1.47(1.31-1.64) | 0.58(0.47-0.69) | 0.12(0.07-0.16) |
| Brazil | 70.39(70.29-70.49) | 46.52(46.43-46.62) | 25.39(25.30-25.48) | 17.26(17.18-17.34) | 9.29(9.22-9.36) | 4.36(4.31-4.40) | 1.89(1.87-1.91) | 0.92(0.91-0.93) | 0.31(0.30-0.32) |
| Brunei Darussalam | 53.66(50.64-56.68) | 47.49(44.56-50.43) | 29.82(26.98-32.67) | 18.48(15.75-21.21) | 9.99(7.56-12.41) | 4.60(2.86-6.34) | 1.88(1.10-2.65) | 0.87(0.36-1.38) | 0.31(-0.05-0.66) |
| Bulgaria | 61.31(60.55-62.08) | 52.78(52.05-53.52) | 33.81(33.13-34.48) | 21.63(21.06-22.20) | 11.59(11.13-12.05) | 5.36(5.08-5.64) | 2.03(1.95-2.11) | 0.83(0.79-0.87) | 0.20(0.18-0.22) |
| Burkina Faso | 52.87(52.37-53.37) | 43.87(43.39-44.35) | 26.77(26.32-27.22) | 17.02(16.62-17.41) | 9.37(9.05-9.69) | 4.43(4.23-4.62) | 1.60(1.53-1.68) | 0.67(0.63-0.72) | 0.16(0.13-0.18) |
| Burundi | 42.36(41.51-43.22) | 37.50(36.68-38.33) | 25.60(24.83-26.37) | 16.50(15.83-17.16) | 9.36(8.83-9.89) | 4.64(4.34-4.95) | 1.87(1.74-2.01) | 0.78(0.69-0.88) | 0.20(0.15-0.24) |
| Cabo Verde | 60.08(57.31-62.85) | 50.24(47.54-52.94) | 31.42(28.82-34.02) | 20.54(18.09-22.99) | 11.85(9.62-14.09) | 6.10(4.45-7.75) | 2.64(1.91-3.37) | 1.35(0.92-1.79) | 0.42(0.21-0.63) |
| Cambodia | 62.29(61.82-62.77) | 52.76(52.31-53.22) | 31.98(31.56-32.41) | 19.86(19.48-20.24) | 10.70(10.37-11.02) | 5.29(5.09-5.50) | 2.06(1.98-2.14) | 0.82(0.77-0.86) | 0.21(0.18-0.23) |
| Cameroon | 54.46(54.04-54.88) | 45.13(44.72-45.54) | 27.36(26.98-27.74) | 17.24(16.92-17.57) | 9.36(9.11-9.62) | 4.35(4.20-4.50) | 1.54(1.48-1.60) | 0.64(0.59-0.68) | 0.14(0.12-0.16) |
| Canada | 69.23(69.00-69.46) | 59.15(58.92-59.37) | 31.91(31.69-32.12) | 18.79(18.60-18.99) | 9.90(9.72-10.07) | 4.63(4.49-4.76) | 2.00(1.93-2.06) | 1.09(1.06-1.12) | 0.48(0.46-0.50) |
| Caribbean | 60.24(59.94-60.53) | 46.20(45.91-46.49) | 27.36(27.09-27.62) | 18.30(18.08-18.53) | 9.91(9.73-10.08) | 4.62(4.51-4.73) | 1.92(1.88-1.97) | 0.90(0.87-0.93) | 0.29(0.27-0.30) |
| Central African Republic | 46.22(45.22-47.22) | 39.41(38.47-40.35) | 24.36(23.53-25.20) | 15.00(14.35-15.65) | 7.83(7.40-8.25) | 3.39(3.18-3.61) | 1.04(0.95-1.14) | 0.35(0.29-0.41) | 0.06(0.03-0.08) |
| Central Asia | 60.20(60.00-60.40) | 51.69(51.49-51.88) | 32.72(32.53-32.90) | 21.00(20.83-21.16) | 11.46(11.32-11.61) | 5.44(5.34-5.53) | 2.14(2.10-2.18) | 0.92(0.89-0.94) | 0.25(0.23-0.26) |
| Central Europe | 62.68(62.52-62.84) | 54.24(54.08-54.39) | 34.90(34.76-35.05) | 22.54(22.41-22.68) | 12.32(12.20-12.44) | 5.92(5.84-6.01) | 2.44(2.41-2.47) | 1.11(1.10-1.12) | 0.33(0.33-0.34) |
| Central Latin America | 63.10(62.98-63.21) | 49.65(49.53-49.76) | 30.61(30.51-30.72) | 19.84(19.75-19.93) | 10.58(10.50-10.66) | 4.94(4.89-5.00) | 2.12(2.10-2.15) | 0.98(0.97-1.00) | 0.32(0.31-0.32) |
| Central Sub-Saharan Africa | 50.79(50.57-51.02) | 43.58(43.36-43.80) | 27.56(27.36-27.76) | 17.47(17.30-17.64) | 9.56(9.43-9.69) | 4.48(4.40-4.55) | 1.59(1.56-1.62) | 0.63(0.61-0.65) | 0.13(0.12-0.14) |
| Chad | 51.39(50.80-51.98) | 42.57(42.00-43.14) | 25.82(25.28-26.35) | 16.30(15.83-16.77) | 8.88(8.51-9.25) | 4.13(3.91-4.36) | 1.45(1.36-1.54) | 0.59(0.53-0.65) | 0.13(0.10-0.16) |
| Chile | 58.74(58.30-59.17) | 51.38(50.95-51.80) | 31.35(30.94-31.76) | 19.78(19.39-20.16) | 11.10(10.76-11.44) | 5.44(5.19-5.69) | 2.34(2.23-2.45) | 1.23(1.17-1.29) | 0.51(0.47-0.55) |
| China | 58.83(58.77-58.88) | 52.40(52.35-52.45) | 35.48(35.43-35.53) | 23.85(23.81-23.90) | 14.24(14.20-14.28) | 8.05(8.01-8.08) | 3.76(3.74-3.78) | 1.80(1.79-1.81) | 0.59(0.58-0.59) |
| Colombia | 64.09(63.84-64.35) | 49.51(49.26-49.76) | 30.57(30.34-30.80) | 19.97(19.76-20.19) | 10.80(10.62-10.99) | 5.17(5.04-5.31) | 2.35(2.29-2.42) | 1.17(1.13-1.20) | 0.42(0.39-0.44) |
| Comoros | 43.65(40.31-46.98) | 38.71(35.53-41.90) | 26.61(23.68-29.54) | 17.31(14.77-19.84) | 9.97(8.01-11.94) | 5.07(3.94-6.20) | 2.14(1.67-2.61) | 0.94(0.64-1.24) | 0.25(0.09-0.42) |
| Congo | 51.31(50.26-52.35) | 43.94(42.94-44.95) | 27.58(26.66-28.50) | 17.29(16.56-18.03) | 9.30(8.79-9.80) | 4.24(3.95-4.53) | 1.45(1.32-1.58) | 0.55(0.47-0.64) | 0.11(0.07-0.15) |
| Cook Islands | 61.97(47.52-76.43) | 53.30(39.25-67.35) | 34.02(20.43-47.62) | 22.25(9.64-34.86) | 12.89(1.96-23.82) | 6.82(-1.03-14.68) | 3.00(-1.01-7.02) | 1.45(-0.56-3.46) | 0.48(-0.58-1.53) |
| Costa Rica | 63.84(63.04-64.64) | 49.67(48.88-50.47) | 30.86(30.10-31.61) | 20.24(19.55-20.93) | 11.05(10.43-11.66) | 5.34(4.89-5.80) | 2.48(2.26-2.71) | 1.27(1.14-1.39) | 0.47(0.39-0.55) |
| Croatia | 62.54(61.73-63.36) | 53.98(53.19-54.77) | 35.04(34.29-35.79) | 22.67(21.98-23.37) | 12.49(11.86-13.12) | 6.11(5.64-6.59) | 2.61(2.42-2.80) | 1.24(1.16-1.32) | 0.39(0.35-0.43) |
| Cuba | 63.30(62.78-63.83) | 48.74(48.22-49.26) | 29.14(28.66-29.63) | 19.65(19.22-20.08) | 10.75(10.39-11.11) | 5.08(4.86-5.29) | 2.14(2.05-2.22) | 1.00(0.96-1.05) | 0.31(0.29-0.34) |
| Cyprus | 69.69(68.66-70.71) | 57.65(56.64-58.66) | 31.27(30.28-32.25) | 18.49(17.55-19.44) | 9.88(8.97-10.78) | 4.42(3.64-5.19) | 1.86(1.46-2.25) | 1.01(0.84-1.18) | 0.43(0.32-0.54) |
| Czechia | 62.57(62.04-63.10) | 54.01(53.50-54.53) | 34.95(34.45-35.44) | 22.68(22.24-23.13) | 12.53(12.14-12.93) | 6.16(5.85-6.47) | 2.65(2.52-2.78) | 1.27(1.22-1.33) | 0.42(0.39-0.45) |
| Côte d’Ivoire | 54.91(54.45-55.37) | 45.60(45.15-46.05) | 27.87(27.45-28.29) | 17.74(17.37-18.11) | 9.80(9.50-10.10) | 4.68(4.49-4.87) | 1.75(1.67-1.84) | 0.77(0.72-0.83) | 0.20(0.16-0.23) |
| Democratic People's Republic of Korea | 57.57(57.14-57.99) | 50.24(49.83-50.65) | 33.40(33.02-33.78) | 22.12(21.78-22.47) | 12.77(12.49-13.06) | 6.92(6.74-7.10) | 3.03(2.95-3.12) | 1.34(1.30-1.39) | 0.41(0.39-0.44) |
| Democratic Republic of the Congo | 51.04(50.76-51.31) | 43.82(43.55-44.09) | 27.80(27.55-28.05) | 17.70(17.48-17.92) | 9.74(9.58-9.91) | 4.59(4.50-4.69) | 1.64(1.60-1.68) | 0.65(0.62-0.68) | 0.14(0.13-0.15) |
| Denmark | 67.33(66.79-67.88) | 56.84(56.30-57.38) | 31.91(31.39-32.43) | 19.00(18.51-19.50) | 10.19(9.74-10.65) | 4.59(4.22-4.95) | 1.94(1.78-2.10) | 1.05(0.99-1.12) | 0.47(0.42-0.51) |
| Djibouti | 43.61(41.00-46.22) | 38.62(36.13-41.11) | 26.37(24.12-28.61) | 16.97(15.13-18.81) | 9.62(8.23-11.01) | 4.80(3.94-5.67) | 1.99(1.55-2.42) | 0.86(0.55-1.16) | 0.22(0.05-0.39) |
| Dominica | 61.59(54.24-68.95) | 47.33(40.20-54.46) | 28.16(21.44-34.87) | 18.89(12.97-24.82) | 10.24(5.28-15.20) | 4.75(1.75-7.74) | 1.92(0.77-3.07) | 0.85(0.23-1.47) | 0.23(-0.07-0.52) |
| Dominican Republic | 61.99(61.41-62.57) | 47.72(47.16-48.29) | 28.53(27.99-29.08) | 19.25(18.76-19.75) | 10.59(10.16-11.01) | 5.07(4.77-5.38) | 2.22(2.09-2.35) | 1.10(1.02-1.18) | 0.38(0.33-0.43) |
| East Asia | 58.83(58.77-58.88) | 52.36(52.31-52.41) | 35.44(35.39-35.49) | 23.82(23.77-23.86) | 14.21(14.17-14.25) | 8.02(7.99-8.05) | 3.75(3.73-3.77) | 1.79(1.78-1.80) | 0.59(0.58-0.59) |
| Eastern Europe | 60.88(60.74-61.02) | 53.06(52.93-53.20) | 33.83(33.70-33.95) | 21.50(21.40-21.61) | 11.50(11.41-11.59) | 5.55(5.49-5.60) | 2.23(2.21-2.24) | 0.93(0.93-0.94) | 0.26(0.25-0.26) |
| Eastern Sub-Saharan Africa | 42.26(42.11-42.40) | 37.54(37.40-37.68) | 25.58(25.45-25.71) | 16.42(16.31-16.53) | 9.30(9.22-9.38) | 4.61(4.56-4.66) | 1.84(1.82-1.86) | 0.76(0.75-0.78) | 0.18(0.18-0.19) |
| Ecuador | 57.61(57.10-58.12) | 44.66(44.16-45.17) | 27.97(27.50-28.45) | 19.30(18.87-19.73) | 10.75(10.38-11.12) | 5.12(4.86-5.37) | 2.27(2.16-2.38) | 1.08(1.02-1.14) | 0.37(0.33-0.41) |
| Egypt | 64.05(63.88-64.23) | 50.27(50.10-50.44) | 30.67(30.51-30.83) | 18.21(18.06-18.37) | 9.21(9.08-9.34) | 4.17(4.08-4.25) | 1.52(1.49-1.55) | 0.54(0.52-0.55) | 0.07(0.07-0.08) |
| El Salvador | 63.22(62.49-63.95) | 49.07(48.35-49.79) | 30.29(29.62-30.96) | 19.73(19.12-20.34) | 10.60(10.08-11.12) | 4.98(4.62-5.33) | 2.19(2.04-2.34) | 1.04(0.96-1.13) | 0.35(0.30-0.40) |
| Equatorial Guinea | 50.86(48.72-53.00) | 43.56(41.51-45.61) | 27.34(25.46-29.23) | 17.17(15.61-18.73) | 9.32(8.16-10.48) | 4.38(3.67-5.08) | 1.60(1.28-1.93) | 0.67(0.45-0.88) | 0.15(0.05-0.26) |
| Eritrea | 43.01(41.88-44.13) | 38.05(36.97-39.13) | 25.89(24.90-26.87) | 16.57(15.74-17.40) | 9.28(8.68-9.88) | 4.49(4.17-4.81) | 1.73(1.58-1.89) | 0.68(0.58-0.78) | 0.15(0.10-0.20) |
| Estonia | 60.42(58.72-62.13) | 52.38(50.69-54.07) | 33.99(32.41-35.56) | 22.04(20.63-23.44) | 12.14(10.87-13.42) | 6.17(5.24-7.10) | 2.75(2.36-3.13) | 1.33(1.18-1.48) | 0.47(0.39-0.56) |
| Eswatini | 48.66(46.56-50.76) | 41.32(39.34-43.30) | 25.07(23.39-26.74) | 15.06(13.86-16.27) | 7.61(6.84-8.39) | 3.19(2.79-3.59) | 0.92(0.77-1.08) | 0.28(0.20-0.36) | 0.04(0.01-0.06) |
| Ethiopia | 41.20(40.89-41.50) | 37.23(36.94-37.53) | 25.89(25.61-26.17) | 16.98(16.73-17.23) | 9.95(9.75-10.15) | 5.12(5.00-5.25) | 2.15(2.10-2.20) | 0.94(0.90-0.97) | 0.24(0.23-0.26) |
| Fiji | 59.24(56.98-61.51) | 50.68(48.51-52.86) | 31.70(29.68-33.71) | 20.18(18.45-21.91) | 11.11(9.76-12.46) | 5.36(4.61-6.11) | 1.93(1.65-2.21) | 0.72(0.55-0.90) | 0.16(0.07-0.25) |
| Finland | 69.69(69.12-70.27) | 57.66(57.09-58.22) | 31.26(30.72-31.81) | 18.51(18.00-19.01) | 9.91(9.45-10.37) | 4.47(4.10-4.85) | 1.93(1.76-2.10) | 1.09(1.02-1.16) | 0.50(0.46-0.55) |
| France | 68.84(68.68-69.00) | 57.43(57.27-57.59) | 31.81(31.66-31.97) | 18.89(18.74-19.03) | 10.15(10.01-10.28) | 4.59(4.48-4.69) | 2.00(1.95-2.06) | 1.15(1.13-1.17) | 0.55(0.53-0.56) |
| Gabon | 52.82(51.01-54.63) | 45.39(43.64-47.15) | 28.85(27.21-30.50) | 18.38(16.96-19.81) | 10.14(9.08-11.21) | 4.84(4.22-5.45) | 1.80(1.54-2.06) | 0.76(0.58-0.93) | 0.17(0.09-0.25) |
| Gambia | 56.03(54.47-57.58) | 46.49(44.97-48.00) | 28.29(26.86-29.73) | 17.90(16.64-19.16) | 9.78(8.77-10.79) | 4.58(3.95-5.21) | 1.64(1.40-1.88) | 0.68(0.53-0.82) | 0.15(0.09-0.21) |
| Georgia | 61.11(60.14-62.09) | 52.51(51.55-53.47) | 33.32(32.42-34.21) | 21.44(20.64-22.23) | 11.75(11.06-12.43) | 5.60(5.14-6.05) | 2.22(2.07-2.37) | 0.95(0.88-1.03) | 0.25(0.22-0.28) |
| Germany | 70.97(70.84-71.10) | 57.77(57.64-57.90) | 30.25(30.13-30.38) | 17.69(17.58-17.81) | 9.35(9.25-9.46) | 4.14(4.06-4.22) | 1.75(1.71-1.78) | 0.97(0.96-0.99) | 0.44(0.43-0.44) |
| Ghana | 56.50(56.11-56.89) | 46.94(46.56-47.32) | 28.70(28.35-29.06) | 18.26(17.95-18.57) | 10.07(9.83-10.32) | 4.80(4.65-4.96) | 1.79(1.73-1.85) | 0.78(0.74-0.82) | 0.19(0.17-0.21) |
| Greece | 69.80(69.41-70.19) | 57.46(57.08-57.84) | 30.93(30.56-31.29) | 18.27(17.92-18.61) | 9.77(9.46-10.08) | 4.33(4.09-4.58) | 1.81(1.70-1.91) | 0.99(0.94-1.03) | 0.43(0.40-0.46) |
| Greenland | 67.68(60.67-74.68) | 57.77(51.02-64.52) | 31.15(24.73-37.56) | 18.09(12.33-23.85) | 9.25(4.39-14.12) | 4.11(1.34-6.89) | 1.62(0.33-2.90) | 0.79(-0.09-1.67) | 0.30(-0.32-0.92) |
| Grenada | 62.17(56.20-68.13) | 47.72(41.87-53.58) | 28.29(22.78-33.80) | 18.91(13.99-23.82) | 10.16(6.31-14.00) | 4.63(2.42-6.84) | 1.82(0.97-2.67) | 0.78(0.29-1.26) | 0.21(-0.05-0.46) |
| Guam | 61.53(56.72-66.34) | 52.92(48.15-57.69) | 33.78(29.16-38.40) | 22.08(17.71-26.45) | 12.79(9.17-16.40) | 6.80(4.33-9.27) | 3.07(1.87-4.27) | 1.59(0.80-2.37) | 0.64(0.12-1.17) |
| Guatemala | 61.89(61.40-62.38) | 47.93(47.45-48.41) | 29.41(28.95-29.86) | 19.01(18.59-19.42) | 10.05(9.71-10.40) | 4.57(4.35-4.80) | 1.90(1.81-1.99) | 0.85(0.79-0.90) | 0.25(0.22-0.28) |
| Guinea | 53.23(52.59-53.88) | 44.11(43.49-44.73) | 26.77(26.19-27.34) | 16.89(16.39-17.39) | 9.19(8.80-9.59) | 4.26(4.03-4.50) | 1.48(1.39-1.58) | 0.60(0.54-0.65) | 0.13(0.10-0.16) |
| Guinea-Bissau | 54.18(52.57-55.78) | 44.78(43.23-46.33) | 26.91(25.49-28.33) | 16.76(15.58-17.95) | 8.92(8.03-9.81) | 3.98(3.48-4.49) | 1.29(1.08-1.50) | 0.47(0.34-0.60) | 0.09(0.03-0.14) |
| Guyana | 60.74(58.43-63.05) | 46.42(44.16-48.68) | 27.18(25.08-29.29) | 17.95(16.16-19.73) | 9.43(8.13-10.73) | 4.13(3.43-4.82) | 1.52(1.25-1.79) | 0.60(0.43-0.78) | 0.15(0.06-0.23) |
| Haiti | 56.48(55.91-57.06) | 42.91(42.36-43.46) | 24.76(24.27-25.25) | 16.15(15.77-16.54) | 8.31(8.02-8.60) | 3.49(3.34-3.65) | 1.18(1.11-1.24) | 0.41(0.37-0.45) | 0.08(0.06-0.10) |
| High-income Asia Pacific | 56.31(56.17-56.45) | 50.00(49.86-50.14) | 31.37(31.24-31.50) | 19.58(19.45-19.70) | 10.92(10.81-11.03) | 5.36(5.27-5.45) | 2.49(2.44-2.54) | 1.37(1.35-1.39) | 0.63(0.62-0.64) |
| High-income North America | 69.74(69.66-69.82) | 60.25(60.17-60.33) | 32.65(32.58-32.73) | 19.07(19.00-19.13) | 9.87(9.82-9.93) | 4.49(4.45-4.53) | 1.84(1.83-1.86) | 0.94(0.93-0.94) | 0.38(0.37-0.38) |
| Honduras | 62.10(61.50-62.70) | 48.02(47.43-48.62) | 29.34(28.78-29.90) | 18.84(18.34-19.35) | 9.82(9.40-10.23) | 4.31(4.06-4.56) | 1.67(1.57-1.76) | 0.66(0.60-0.72) | 0.16(0.13-0.19) |
| Hungary | 62.09(61.54-62.65) | 53.54(53.00-54.09) | 34.50(33.98-35.02) | 22.26(21.81-22.72) | 12.15(11.77-12.54) | 5.82(5.54-6.09) | 2.38(2.29-2.47) | 1.09(1.04-1.13) | 0.34(0.31-0.36) |
| Iceland | 69.68(67.49-71.87) | 57.65(55.46-59.83) | 31.27(29.15-33.39) | 18.50(16.48-20.53) | 9.90(8.03-11.77) | 4.46(2.97-5.96) | 1.91(1.16-2.66) | 1.07(0.72-1.42) | 0.49(0.26-0.72) |
| India | 60.65(60.60-60.71) | 50.47(50.42-50.52) | 31.40(31.35-31.45) | 20.23(20.18-20.27) | 11.07(11.04-11.11) | 5.24(5.22-5.27) | 2.07(2.06-2.08) | 0.84(0.84-0.85) | 0.22(0.21-0.22) |
| Indonesia | 64.43(64.32-64.54) | 55.19(55.09-55.29) | 33.31(33.22-33.41) | 20.65(20.57-20.74) | 11.19(11.12-11.26) | 5.54(5.49-5.59) | 2.16(2.14-2.18) | 0.86(0.85-0.88) | 0.22(0.21-0.22) |
| Iran (Islamic Republic of) | 66.65(66.47-66.84) | 55.40(55.22-55.58) | 34.45(34.29-34.62) | 20.97(20.82-21.12) | 11.08(10.94-11.22) | 5.48(5.38-5.59) | 2.35(2.30-2.40) | 1.11(1.08-1.14) | 0.31(0.29-0.32) |
| Iraq | 62.49(62.19-62.79) | 51.55(51.26-51.85) | 32.48(32.20-32.76) | 19.56(19.30-19.82) | 10.08(9.85-10.31) | 4.83(4.68-4.99) | 1.99(1.92-2.06) | 0.89(0.84-0.93) | 0.23(0.21-0.25) |
| Ireland | 69.66(69.10-70.22) | 57.63(57.08-58.19) | 31.26(30.72-31.80) | 18.50(17.98-19.02) | 9.89(9.41-10.37) | 4.45(4.05-4.85) | 1.90(1.70-2.10) | 1.07(0.97-1.16) | 0.48(0.42-0.55) |
| Israel | 69.75(69.31-70.20) | 57.72(57.28-58.15) | 31.33(30.90-31.76) | 18.56(18.15-18.98) | 9.95(9.56-10.34) | 4.50(4.16-4.84) | 1.94(1.77-2.12) | 1.10(1.02-1.17) | 0.50(0.45-0.55) |
| Italy | 73.13(72.99-73.27) | 60.19(60.06-60.33) | 31.36(31.23-31.49) | 18.50(18.38-18.62) | 9.75(9.64-9.86) | 4.33(4.24-4.42) | 1.83(1.78-1.88) | 1.02(1.00-1.04) | 0.46(0.45-0.47) |
| Jamaica | 62.46(61.35-63.56) | 48.04(46.96-49.13) | 28.64(27.60-29.68) | 19.26(18.31-20.20) | 10.50(9.71-11.30) | 4.95(4.43-5.48) | 2.11(1.90-2.32) | 1.00(0.88-1.13) | 0.33(0.26-0.40) |
| Japan | 56.00(55.83-56.18) | 50.03(49.86-50.20) | 31.63(31.47-31.80) | 19.78(19.63-19.93) | 11.04(10.90-11.18) | 5.45(5.33-5.57) | 2.54(2.48-2.60) | 1.40(1.38-1.42) | 0.65(0.64-0.66) |
| Jordan | 63.52(62.98-64.06) | 52.51(51.98-53.04) | 33.31(32.79-33.83) | 20.26(19.77-20.76) | 10.66(10.21-11.12) | 5.30(4.95-5.66) | 2.33(2.16-2.51) | 1.13(1.02-1.23) | 0.34(0.27-0.40) |
| Kazakhstan | 60.65(60.21-61.10) | 52.06(51.62-52.50) | 32.89(32.48-33.31) | 21.04(20.67-21.41) | 11.41(11.10-11.72) | 5.35(5.15-5.55) | 2.07(1.99-2.14) | 0.86(0.82-0.90) | 0.21(0.19-0.23) |
| Kenya | 45.02(44.62-45.43) | 40.24(39.85-40.63) | 27.38(27.02-27.75) | 17.62(17.30-17.94) | 9.96(9.72-10.20) | 5.00(4.85-5.14) | 2.05(1.98-2.11) | 0.88(0.83-0.92) | 0.21(0.19-0.24) |
| Kiribati | 57.73(51.32-64.14) | 49.34(43.19-55.49) | 30.76(25.08-36.45) | 19.54(14.65-24.42) | 10.75(6.88-14.61) | 5.20(2.88-7.52) | 1.90(0.97-2.83) | 0.72(0.13-1.31) | 0.16(-0.15-0.46) |
| Kuwait | 64.23(63.30-65.16) | 53.37(52.46-54.27) | 34.22(33.34-35.10) | 21.04(20.18-21.90) | 11.39(10.55-12.23) | 5.98(5.19-6.77) | 2.92(2.33-3.51) | 1.60(1.25-1.96) | 0.62(0.42-0.82) |
| Kyrgyzstan | 60.65(59.87-61.42) | 52.10(51.34-52.86) | 33.05(32.32-33.78) | 21.27(20.60-21.95) | 11.68(11.09-12.28) | 5.62(5.20-6.04) | 2.29(2.11-2.47) | 1.03(0.92-1.15) | 0.31(0.25-0.37) |
| Lao People's Democratic Republic | 61.55(60.80-62.29) | 52.11(51.40-52.82) | 31.57(30.90-32.23) | 19.60(19.01-20.19) | 10.57(10.06-11.08) | 5.24(4.90-5.58) | 2.05(1.90-2.19) | 0.82(0.74-0.91) | 0.22(0.17-0.26) |
| Latvia | 59.97(58.54-61.39) | 51.93(50.53-53.33) | 33.55(32.22-34.89) | 21.61(20.43-22.79) | 11.74(10.75-12.74) | 5.82(5.18-6.45) | 2.47(2.24-2.69) | 1.13(1.03-1.23) | 0.36(0.31-0.42) |
| Lebanon | 63.76(63.02-64.50) | 52.71(51.98-53.44) | 33.44(32.74-34.13) | 20.34(19.70-20.99) | 10.72(10.13-11.31) | 5.35(4.91-5.79) | 2.38(2.19-2.58) | 1.18(1.08-1.28) | 0.37(0.31-0.42) |
| Lesotho | 46.48(44.90-48.05) | 39.37(37.92-40.82) | 23.64(22.42-24.85) | 13.96(13.12-14.80) | 6.83(6.35-7.31) | 2.72(2.49-2.95) | 0.73(0.64-0.81) | 0.20(0.16-0.24) | 0.02(0.01-0.03) |
| Liberia | 54.53(53.51-55.56) | 45.22(44.24-46.20) | 27.50(26.59-28.42) | 17.41(16.62-18.19) | 9.52(8.90-10.14) | 4.46(4.05-4.87) | 1.59(1.42-1.75) | 0.66(0.55-0.77) | 0.15(0.10-0.20) |
| Libya | 62.18(61.47-62.89) | 51.27(50.60-51.94) | 32.27(31.64-32.89) | 19.41(18.85-19.96) | 10.00(9.53-10.47) | 4.81(4.48-5.14) | 2.00(1.85-2.14) | 0.91(0.81-1.01) | 0.26(0.20-0.31) |
| Lithuania | 59.80(58.62-60.98) | 51.91(50.76-53.06) | 33.64(32.54-34.74) | 21.71(20.71-22.71) | 11.83(10.99-12.68) | 5.93(5.39-6.46) | 2.57(2.36-2.77) | 1.19(1.10-1.29) | 0.40(0.35-0.44) |
| Luxembourg | 68.65(67.07-70.23) | 57.50(55.93-59.08) | 31.63(30.11-33.14) | 18.82(17.40-20.25) | 10.12(8.77-11.46) | 4.58(3.45-5.70) | 1.97(1.37-2.57) | 1.11(0.84-1.37) | 0.51(0.34-0.67) |
| Madagascar | 42.35(41.80-42.90) | 37.46(36.93-37.99) | 25.47(24.99-25.96) | 16.31(15.90-16.73) | 9.16(8.85-9.46) | 4.45(4.28-4.62) | 1.72(1.64-1.80) | 0.68(0.62-0.73) | 0.15(0.12-0.18) |
| Malawi | 41.95(41.31-42.60) | 37.03(36.42-37.65) | 24.97(24.40-25.54) | 15.78(15.31-16.25) | 8.67(8.34-9.00) | 4.10(3.92-4.29) | 1.54(1.46-1.62) | 0.58(0.53-0.63) | 0.12(0.10-0.14) |
| Malaysia | 61.86(61.52-62.21) | 53.50(53.16-53.83) | 33.73(33.41-34.05) | 21.29(20.98-21.59) | 11.77(11.50-12.04) | 6.00(5.82-6.19) | 2.52(2.44-2.59) | 1.09(1.04-1.13) | 0.31(0.29-0.34) |
| Maldives | 65.00(62.15-67.86) | 55.30(52.53-58.07) | 34.12(31.43-36.80) | 21.68(19.09-24.26) | 12.19(9.72-14.67) | 6.49(4.36-8.62) | 2.93(1.61-4.26) | 1.43(0.76-2.10) | 0.50(0.13-0.88) |
| Mali | 52.46(51.96-52.96) | 43.46(42.97-43.94) | 26.34(25.89-26.79) | 16.60(16.20-16.99) | 9.00(8.70-9.31) | 4.15(3.97-4.32) | 1.42(1.35-1.49) | 0.56(0.52-0.60) | 0.12(0.10-0.14) |
| Malta | 69.49(67.57-71.42) | 57.49(55.63-59.35) | 31.18(29.38-32.97) | 18.44(16.69-20.19) | 9.85(8.22-11.48) | 4.41(3.11-5.72) | 1.87(1.34-2.40) | 1.04(0.80-1.27) | 0.46(0.30-0.62) |
| Marshall Islands | 58.56(49.20-67.91) | 50.00(40.91-59.09) | 31.02(22.54-39.49) | 19.56(12.52-26.59) | 10.62(5.41-15.83) | 5.04(1.99-8.09) | 1.78(0.39-3.18) | 0.66(-0.31-1.62) | 0.14(-0.41-0.69) |
| Mauritania | 57.73(56.59-58.86) | 48.06(46.95-49.18) | 29.62(28.55-30.68) | 19.02(18.05-19.98) | 10.63(9.82-11.44) | 5.15(4.63-5.67) | 1.96(1.76-2.16) | 0.87(0.74-1.01) | 0.22(0.15-0.29) |
| Mauritius | 64.23(62.66-65.80) | 54.53(53.00-56.06) | 33.34(31.90-34.79) | 20.93(19.63-22.24) | 11.52(10.43-12.61) | 5.93(5.20-6.65) | 2.52(2.22-2.83) | 1.15(0.98-1.32) | 0.38(0.27-0.48) |
| Mexico | 63.04(62.89-63.20) | 50.36(50.21-50.52) | 31.06(30.91-31.20) | 20.06(19.93-20.19) | 10.64(10.53-10.74) | 4.94(4.88-5.01) | 2.08(2.05-2.11) | 0.94(0.92-0.95) | 0.29(0.28-0.30) |
| Micronesia (Federated States of) | 59.50(52.58-66.41) | 50.90(44.17-57.64) | 31.83(25.46-38.21) | 20.27(14.72-25.82) | 11.20(6.95-15.45) | 5.47(3.01-7.92) | 2.05(0.99-3.11) | 0.81(0.10-1.51) | 0.19(-0.16-0.54) |
| Monaco | 69.23(61.73-76.74) | 57.22(49.98-64.45) | 30.88(24.01-37.76) | 18.16(11.96-24.36) | 9.60(4.38-14.82) | 4.22(0.56-7.88) | 1.72(0.24-3.20) | 0.92(0.28-1.55) | 0.39(-0.02-0.80) |
| Mongolia | 60.42(59.33-61.51) | 51.88(50.81-52.95) | 32.83(31.82-33.84) | 21.06(20.15-21.97) | 11.49(10.71-12.27) | 5.46(4.92-6.00) | 2.17(1.93-2.41) | 0.94(0.79-1.09) | 0.25(0.17-0.33) |
| Montenegro | 61.97(59.78-64.17) | 53.42(51.25-55.59) | 34.39(32.38-36.39) | 22.15(20.30-24.00) | 12.04(10.44-13.64) | 5.70(4.55-6.85) | 2.24(1.83-2.64) | 0.93(0.74-1.11) | 0.20(0.13-0.28) |
| Morocco | 62.81(62.53-63.10) | 51.83(51.54-52.11) | 32.67(32.40-32.93) | 19.67(19.43-19.92) | 10.14(9.93-10.35) | 4.86(4.72-4.99) | 2.00(1.94-2.05) | 0.89(0.86-0.93) | 0.24(0.22-0.25) |
| Mozambique | 40.63(40.13-41.13) | 35.76(35.28-36.24) | 23.86(23.43-24.29) | 14.91(14.56-15.25) | 8.15(7.91-8.39) | 3.89(3.75-4.04) | 1.48(1.41-1.54) | 0.56(0.52-0.60) | 0.12(0.10-0.13) |
| Myanmar | 61.85(61.59-62.11) | 52.39(52.14-52.63) | 31.78(31.55-32.01) | 19.77(19.56-19.97) | 10.70(10.53-10.86) | 5.34(5.24-5.45) | 2.12(2.08-2.17) | 0.87(0.84-0.89) | 0.23(0.22-0.25) |
| Namibia | 51.80(50.27-53.32) | 44.39(42.93-45.86) | 27.92(26.58-29.27) | 17.54(16.43-18.65) | 9.45(8.67-10.23) | 4.34(3.92-4.77) | 1.50(1.33-1.67) | 0.57(0.47-0.68) | 0.11(0.07-0.15) |
| Nauru | 57.98(35.85-80.10) | 49.47(27.95-70.99) | 30.61(10.49-50.74) | 19.24(2.14-36.34) | 10.39(-2.75-23.54) | 4.89(-3.00-12.78) | 1.70(-1.52-4.92) | 0.61(-1.18-2.40) | 0.12(-0.79-1.04) |
| Nepal | 60.26(59.90-60.61) | 49.03(48.69-49.38) | 30.50(30.18-30.82) | 19.56(19.27-19.85) | 10.68(10.43-10.92) | 5.01(4.85-5.17) | 1.95(1.89-2.01) | 0.77(0.73-0.80) | 0.18(0.17-0.20) |
| Netherlands | 69.38(69.07-69.68) | 57.58(57.29-57.88) | 31.29(31.00-31.58) | 18.55(18.27-18.82) | 9.88(9.63-10.12) | 4.39(4.20-4.59) | 1.84(1.76-1.93) | 1.01(0.97-1.05) | 0.44(0.42-0.47) |
| New Zealand | 61.67(60.89-62.45) | 54.73(53.97-55.49) | 33.14(32.42-33.86) | 20.79(20.11-21.47) | 11.55(10.93-12.17) | 5.62(5.14-6.11) | 2.46(2.22-2.71) | 1.34(1.22-1.45) | 0.55(0.48-0.62) |
| Nicaragua | 63.15(62.44-63.86) | 49.05(48.35-49.75) | 30.31(29.64-30.98) | 19.75(19.13-20.37) | 10.61(10.07-11.16) | 4.97(4.59-5.36) | 2.17(2.00-2.34) | 1.02(0.92-1.13) | 0.34(0.28-0.41) |
| Niger | 53.32(52.80-53.84) | 44.25(43.75-44.76) | 27.01(26.53-27.49) | 17.17(16.74-17.60) | 9.46(9.12-9.80) | 4.47(4.26-4.68) | 1.62(1.53-1.70) | 0.68(0.63-0.74) | 0.16(0.13-0.19) |
| Nigeria | 54.84(54.69-54.99) | 46.24(46.10-46.39) | 28.08(27.95-28.22) | 17.89(17.76-18.01) | 9.92(9.82-10.02) | 4.81(4.74-4.87) | 1.85(1.82-1.88) | 0.83(0.81-0.85) | 0.21(0.20-0.22) |
| Niue | 57.10(-10.71-124.90) | 48.85(-13.30-111.01) | 30.70(-24.42-85.83) | 19.78(-26.89-66.44) | 11.20(-27.03-49.42) | 5.69(-21.11-32.49) | 2.29(-10.21-14.79) | 0.97(-3.87-5.82) | 0.25(-1.68-2.19) |
| North Africa and Middle East | 63.08(63.00-63.15) | 51.57(51.49-51.64) | 32.32(32.25-32.39) | 19.51(19.45-19.58) | 10.11(10.06-10.17) | 4.88(4.84-4.92) | 2.03(2.01-2.05) | 0.92(0.91-0.93) | 0.24(0.23-0.24) |
| North Macedonia | 61.63(60.45-62.81) | 53.09(51.93-54.25) | 34.07(32.99-35.16) | 21.86(20.88-22.84) | 11.77(10.93-12.61) | 5.48(4.92-6.03) | 2.08(1.89-2.26) | 0.82(0.73-0.91) | 0.17(0.13-0.21) |
| Northern Mariana Islands | 61.20(51.73-70.66) | 52.52(43.24-61.81) | 33.25(24.37-42.13) | 21.50(13.66-29.34) | 12.20(5.80-18.61) | 6.23(2.06-10.40) | 2.55(0.56-4.54) | 1.12(-0.16-2.40) | 0.31(-0.40-1.03) |
| Norway | 71.48(70.96-72.00) | 59.46(58.95-59.98) | 31.27(30.78-31.76) | 18.61(18.14-19.07) | 9.91(9.48-10.35) | 4.49(4.12-4.85) | 1.91(1.71-2.10) | 1.05(0.98-1.13) | 0.47(0.42-0.52) |
| Oceania | 57.08(56.46-57.70) | 48.78(48.19-49.38) | 30.41(29.86-30.96) | 19.32(18.85-19.79) | 10.64(10.27-11.02) | 5.16(4.93-5.39) | 1.88(1.78-1.98) | 0.71(0.65-0.78) | 0.16(0.13-0.19) |
| Oman | 63.47(62.45-64.50) | 52.44(51.42-53.45) | 33.18(32.21-34.15) | 20.11(19.19-21.03) | 10.50(9.63-11.37) | 5.14(4.43-5.85) | 2.17(1.88-2.47) | 1.00(0.81-1.19) | 0.29(0.17-0.40) |
| Pakistan | 56.85(56.71-57.00) | 47.58(47.44-47.73) | 29.64(29.51-29.77) | 18.96(18.85-19.08) | 10.24(10.15-10.33) | 4.76(4.70-4.82) | 1.80(1.78-1.83) | 0.68(0.66-0.69) | 0.15(0.14-0.16) |
| Palau | 59.34(39.65-79.03) | 50.77(31.72-69.82) | 31.76(14.82-48.70) | 20.40(7.56-33.24) | 11.48(2.09-20.87) | 5.77(-0.29-11.82) | 2.23(-0.44-4.89) | 0.85(-0.51-2.20) | 0.17(-0.53-0.87) |
| Palestine | 63.39(62.52-64.26) | 52.38(51.52-53.23) | 33.16(32.33-34.00) | 20.12(19.32-20.92) | 10.52(9.78-11.26) | 5.16(4.59-5.73) | 2.19(1.94-2.44) | 1.01(0.86-1.15) | 0.27(0.19-0.36) |
| Panama | 63.53(62.62-64.44) | 49.43(48.54-50.33) | 30.71(29.86-31.57) | 20.16(19.37-20.95) | 11.02(10.33-11.72) | 5.36(4.83-5.89) | 2.51(2.24-2.78) | 1.29(1.15-1.43) | 0.49(0.40-0.58) |
| Papua New Guinea | 56.54(55.82-57.27) | 48.30(47.61-48.99) | 30.06(29.42-30.69) | 19.06(18.51-19.61) | 10.47(10.03-10.90) | 5.04(4.77-5.31) | 1.81(1.68-1.93) | 0.66(0.58-0.74) | 0.14(0.10-0.18) |
| Paraguay | 68.78(68.18-69.37) | 44.91(44.32-45.50) | 24.77(24.21-25.32) | 16.85(16.34-17.36) | 9.06(8.61-9.50) | 4.20(3.89-4.51) | 1.77(1.65-1.90) | 0.83(0.75-0.90) | 0.26(0.21-0.30) |
| Peru | 52.08(51.68-52.47) | 42.19(41.80-42.58) | 27.42(27.06-27.78) | 18.92(18.59-19.24) | 10.60(10.33-10.87) | 5.06(4.89-5.24) | 2.20(2.13-2.27) | 0.98(0.94-1.02) | 0.31(0.28-0.33) |
| Philippines | 64.41(64.22-64.59) | 55.15(54.98-55.33) | 33.26(33.10-33.43) | 20.60(20.45-20.75) | 11.15(11.03-11.28) | 5.54(5.46-5.62) | 2.19(2.15-2.22) | 0.90(0.88-0.92) | 0.24(0.23-0.25) |
| Poland | 63.92(63.65-64.19) | 55.67(55.41-55.94) | 35.67(35.42-35.91) | 23.07(22.85-23.30) | 12.68(12.48-12.89) | 6.16(6.00-6.31) | 2.59(2.53-2.65) | 1.22(1.19-1.24) | 0.39(0.37-0.40) |
| Portugal | 69.66(69.26-70.05) | 57.62(57.25-58.00) | 31.25(30.88-31.61) | 18.49(18.15-18.83) | 9.89(9.58-10.19) | 4.45(4.21-4.70) | 1.91(1.79-2.03) | 1.07(1.02-1.12) | 0.48(0.45-0.51) |
| Puerto Rico | 63.90(62.98-64.83) | 49.36(48.44-50.28) | 29.78(28.89-30.67) | 20.28(19.48-21.09) | 11.36(10.66-12.07) | 5.64(5.12-6.17) | 2.64(2.39-2.89) | 1.41(1.29-1.52) | 0.55(0.48-0.62) |
| Qatar | 64.24(62.77-65.72) | 53.16(51.70-54.61) | 33.80(32.38-35.22) | 20.64(19.25-22.02) | 10.93(9.57-12.28) | 5.47(4.25-6.70) | 2.41(1.68-3.15) | 1.16(0.68-1.64) | 0.35(0.04-0.65) |
| Republic of Korea | 57.47(57.21-57.72) | 50.25(50.00-50.50) | 30.84(30.60-31.08) | 19.13(18.90-19.35) | 10.59(10.39-10.79) | 5.09(4.93-5.26) | 2.31(2.22-2.41) | 1.25(1.21-1.29) | 0.55(0.52-0.57) |
| Republic of Moldova | 59.41(58.40-60.42) | 51.42(50.45-52.39) | 33.16(32.26-34.05) | 21.29(20.50-22.09) | 11.50(10.81-12.18) | 5.62(5.17-6.06) | 2.31(2.16-2.45) | 1.00(0.92-1.08) | 0.32(0.27-0.36) |
| Romania | 61.82(61.40-62.23) | 53.29(52.89-53.69) | 34.30(33.92-34.68) | 22.10(21.76-22.44) | 12.01(11.73-12.30) | 5.71(5.52-5.91) | 2.30(2.24-2.36) | 1.02(0.99-1.05) | 0.29(0.28-0.31) |
| Russian Federation | 61.04(60.87-61.20) | 53.20(53.04-53.37) | 33.82(33.67-33.98) | 21.45(21.32-21.58) | 11.43(11.33-11.54) | 5.49(5.42-5.55) | 2.18(2.16-2.20) | 0.90(0.89-0.91) | 0.24(0.24-0.25) |
| Rwanda | 43.75(42.96-44.53) | 38.77(38.01-39.53) | 26.56(25.85-27.27) | 17.17(16.56-17.79) | 9.80(9.32-10.27) | 4.91(4.64-5.19) | 2.03(1.91-2.15) | 0.87(0.79-0.95) | 0.22(0.18-0.26) |
| Saint Kitts and Nevis | 62.45(55.17-69.73) | 48.06(41.04-55.08) | 28.70(22.15-35.24) | 19.33(13.48-25.17) | 10.55(5.54-15.56) | 4.95(1.58-8.33) | 2.06(0.68-3.44) | 0.93(0.06-1.81) | 0.26(-0.21-0.74) |
| Saint Lucia | 62.51(58.19-66.84) | 48.11(43.93-52.29) | 28.72(24.81-32.64) | 19.35(15.89-22.80) | 10.58(7.73-13.44) | 5.01(3.14-6.89) | 2.15(1.35-2.94) | 1.03(0.57-1.49) | 0.33(0.07-0.59) |
| Saint Vincent and the Grenadines | 62.27(56.26-68.28) | 47.83(42.05-53.62) | 28.44(23.06-33.82) | 19.09(14.45-23.72) | 10.37(6.63-14.10) | 4.87(2.51-7.23) | 2.06(1.05-3.08) | 0.98(0.39-1.57) | 0.30(-0.02-0.63) |
| Samoa | 60.07(55.06-65.09) | 51.47(46.54-56.40) | 32.37(27.64-37.10) | 20.77(16.53-25.01) | 11.62(8.15-15.10) | 5.80(3.66-7.93) | 2.26(1.38-3.15) | 0.94(0.42-1.46) | 0.24(-0.03-0.51) |
| San Marino | 69.92(63.19-76.65) | 57.88(51.35-64.41) | 31.48(25.16-37.80) | 18.70(12.71-24.69) | 10.08(4.57-15.60) | 4.63(-0.10-9.36) | 2.06(-0.94-5.06) | 1.21(-0.09-2.50) | 0.59(-0.14-1.32) |
| Sao Tome and Principe | 58.93(54.13-63.72) | 49.12(44.41-53.82) | 30.38(25.85-34.90) | 19.57(15.44-23.71) | 11.00(7.46-14.53) | 5.37(3.01-7.74) | 2.09(1.11-3.07) | 0.96(0.32-1.60) | 0.25(-0.07-0.57) |
| Saudi Arabia | 61.97(61.64-62.31) | 51.76(51.43-52.09) | 33.10(32.79-33.41) | 20.04(19.76-20.32) | 10.41(10.16-10.66) | 5.03(4.84-5.22) | 2.13(2.02-2.23) | 0.99(0.91-1.07) | 0.28(0.23-0.34) |
| Senegal | 56.92(56.32-57.51) | 47.33(46.76-47.91) | 29.05(28.50-29.60) | 18.56(18.07-19.05) | 10.29(9.89-10.69) | 4.92(4.67-5.16) | 1.82(1.73-1.91) | 0.78(0.72-0.84) | 0.19(0.16-0.21) |
| Serbia | 62.08(61.53-62.63) | 53.53(52.99-54.07) | 34.49(33.97-35.01) | 22.25(21.76-22.73) | 12.10(11.67-12.53) | 5.75(5.44-6.06) | 2.28(2.18-2.38) | 0.97(0.93-1.02) | 0.26(0.23-0.28) |
| Seychelles | 64.01(57.98-70.04) | 54.33(48.64-60.02) | 33.21(27.63-38.80) | 20.85(16.04-25.66) | 11.49(7.48-15.50) | 5.94(3.11-8.76) | 2.54(1.14-3.94) | 1.15(0.43-1.87) | 0.36(-0.03-0.76) |
| Sierra Leone | 52.64(51.84-53.43) | 43.63(42.87-44.38) | 26.51(25.81-27.21) | 16.77(16.16-17.39) | 9.18(8.69-9.67) | 4.30(4.00-4.60) | 1.54(1.42-1.65) | 0.64(0.57-0.71) | 0.15(0.12-0.19) |
| Singapore | 52.03(51.18-52.87) | 46.53(45.70-47.36) | 30.22(29.46-30.97) | 19.10(18.38-19.82) | 10.76(10.07-11.45) | 5.34(4.74-5.93) | 2.50(2.18-2.82) | 1.37(1.22-1.53) | 0.64(0.53-0.74) |
| Slovakia | 62.13(61.39-62.86) | 53.59(52.87-54.30) | 34.58(33.91-35.25) | 22.36(21.75-22.97) | 12.25(11.71-12.79) | 5.91(5.51-6.31) | 2.44(2.30-2.59) | 1.11(1.05-1.18) | 0.33(0.29-0.37) |
| Slovenia | 62.95(61.82-64.08) | 54.39(53.29-55.48) | 35.32(34.28-36.35) | 23.04(22.08-24.01) | 12.87(11.97-13.77) | 6.46(5.72-7.19) | 2.89(2.52-3.26) | 1.47(1.32-1.63) | 0.54(0.46-0.62) |
| Solomon Islands | 58.88(56.11-61.65) | 50.33(47.63-53.04) | 31.38(28.83-33.93) | 19.91(17.71-22.11) | 10.93(9.21-12.65) | 5.28(4.21-6.36) | 1.94(1.45-2.42) | 0.74(0.44-1.04) | 0.17(0.01-0.32) |
| Somalia | 39.33(38.71-39.95) | 34.64(34.05-35.23) | 23.17(22.64-23.70) | 14.48(14.05-14.91) | 7.78(7.50-8.05) | 3.49(3.34-3.64) | 1.15(1.09-1.22) | 0.36(0.32-0.40) | 0.05(0.04-0.07) |
| South Africa | 52.78(52.47-53.10) | 45.72(45.42-46.02) | 28.64(28.37-28.92) | 17.99(17.79-18.20) | 9.78(9.63-9.93) | 4.62(4.54-4.71) | 1.73(1.70-1.77) | 0.74(0.72-0.77) | 0.17(0.16-0.18) |
| South Asia | 59.97(59.92-60.02) | 49.89(49.85-49.94) | 31.10(31.06-31.14) | 20.05(20.01-20.08) | 10.98(10.95-11.01) | 5.21(5.19-5.23) | 2.06(2.06-2.07) | 0.84(0.84-0.85) | 0.22(0.21-0.22) |
| South Sudan | 38.23(37.33-39.13) | 33.75(32.90-34.60) | 22.82(22.04-23.60) | 14.51(13.89-15.13) | 8.08(7.64-8.51) | 3.89(3.65-4.14) | 1.49(1.37-1.62) | 0.58(0.50-0.66) | 0.13(0.09-0.17) |
| Southeast Asia | 64.24(64.17-64.31) | 54.80(54.73-54.87) | 33.23(33.17-33.29) | 20.71(20.65-20.76) | 11.32(11.28-11.37) | 5.72(5.69-5.75) | 2.34(2.33-2.35) | 1.01(1.00-1.02) | 0.30(0.29-0.30) |
| Southern Latin America | 56.87(56.62-57.12) | 50.12(49.87-50.36) | 30.99(30.76-31.22) | 19.53(19.32-19.75) | 10.95(10.77-11.14) | 5.34(5.21-5.48) | 2.25(2.20-2.30) | 1.14(1.11-1.16) | 0.45(0.43-0.46) |
| Southern Sub-Saharan Africa | 51.77(51.51-52.04) | 44.64(44.38-44.89) | 27.90(27.68-28.13) | 17.45(17.28-17.63) | 9.38(9.26-9.50) | 4.35(4.28-4.42) | 1.56(1.54-1.59) | 0.64(0.62-0.66) | 0.14(0.13-0.15) |
| Spain | 70.18(70.00-70.36) | 57.74(57.57-57.92) | 31.09(30.92-31.26) | 18.45(18.29-18.60) | 9.92(9.78-10.07) | 4.49(4.37-4.61) | 1.95(1.89-2.02) | 1.12(1.09-1.15) | 0.53(0.51-0.54) |
| Sri Lanka | 65.03(64.66-65.40) | 55.27(54.91-55.63) | 33.96(33.62-34.31) | 21.49(21.17-21.80) | 11.99(11.71-12.27) | 6.30(6.09-6.52) | 2.77(2.67-2.88) | 1.30(1.25-1.35) | 0.43(0.40-0.47) |
| Sudan | 60.81(60.49-61.13) | 50.06(49.74-50.37) | 31.34(31.05-31.64) | 18.72(18.45-18.99) | 9.52(9.29-9.75) | 4.48(4.33-4.64) | 1.78(1.71-1.85) | 0.76(0.72-0.81) | 0.19(0.17-0.21) |
| Suriname | 61.46(58.79-64.13) | 47.17(44.57-49.77) | 27.98(25.57-30.40) | 18.75(16.71-20.79) | 10.16(8.55-11.78) | 4.76(3.77-5.74) | 1.99(1.58-2.41) | 0.94(0.68-1.19) | 0.30(0.15-0.45) |
| Sweden | 71.73(71.35-72.10) | 59.77(59.40-60.13) | 31.74(31.39-32.10) | 18.79(18.45-19.13) | 9.96(9.64-10.27) | 4.49(4.23-4.75) | 1.91(1.77-2.05) | 1.07(1.01-1.12) | 0.48(0.44-0.51) |
| Switzerland | 67.97(67.53-68.41) | 57.33(56.91-57.75) | 32.07(31.67-32.47) | 19.17(18.78-19.55) | 10.34(9.98-10.70) | 4.76(4.45-5.06) | 2.09(1.92-2.26) | 1.20(1.13-1.27) | 0.57(0.52-0.61) |
| Syrian Arab Republic | 63.09(62.60-63.58) | 52.06(51.59-52.53) | 32.85(32.41-33.30) | 19.84(19.45-20.24) | 10.30(9.96-10.63) | 4.99(4.76-5.22) | 2.08(1.98-2.17) | 0.93(0.87-0.99) | 0.22(0.19-0.26) |
| Taiwan (Province of China) | 60.87(60.48-61.27) | 52.69(52.31-53.08) | 34.84(34.48-35.20) | 23.29(22.96-23.62) | 13.80(13.50-14.10) | 7.82(7.58-8.06) | 3.78(3.63-3.92) | 1.94(1.86-2.01) | 0.75(0.71-0.79) |
| Tajikistan | 59.19(58.53-59.85) | 50.79(50.15-51.43) | 32.07(31.46-32.69) | 20.51(19.95-21.08) | 11.12(10.63-11.62) | 5.21(4.88-5.54) | 1.99(1.85-2.13) | 0.82(0.72-0.91) | 0.21(0.16-0.26) |
| Thailand | 66.97(66.75-67.18) | 55.64(55.44-55.85) | 32.94(32.76-33.13) | 20.55(20.39-20.72) | 11.39(11.26-11.53) | 5.96(5.86-6.05) | 2.65(2.60-2.70) | 1.30(1.27-1.33) | 0.47(0.46-0.49) |
| Timor-Leste | 61.75(59.90-63.60) | 52.28(50.49-54.07) | 31.67(29.96-33.38) | 19.66(18.08-21.24) | 10.59(9.25-11.93) | 5.24(4.35-6.14) | 2.04(1.72-2.36) | 0.81(0.63-1.00) | 0.21(0.10-0.33) |
| Togo | 55.63(54.84-56.43) | 46.19(45.42-46.96) | 28.20(27.48-28.92) | 17.92(17.31-18.53) | 9.87(9.38-10.35) | 4.67(4.37-4.97) | 1.70(1.58-1.83) | 0.72(0.64-0.80) | 0.17(0.13-0.21) |
| Tokelau | 56.33(-20.84-133.49) | 48.17(-24.81-121.15) | 30.20(-36.70-97.09) | 19.40(-39.00-77.79) | 10.94(-38.65-60.53) | 5.54(-31.81-42.89) | 2.23(-14.78-19.24) | 0.97(-5.63-7.56) | 0.27(-2.44-2.97) |
| Tonga | 60.91(54.21-67.61) | 52.29(45.70-58.89) | 33.15(26.77-39.53) | 21.48(15.59-27.38) | 12.24(7.22-17.27) | 6.30(2.85-9.75) | 2.63(1.06-4.19) | 1.20(0.35-2.05) | 0.36(-0.09-0.82) |
| Trinidad and Tobago | 62.32(60.67-63.97) | 47.88(46.25-49.50) | 28.45(26.93-29.97) | 19.07(17.80-20.34) | 10.33(9.30-11.36) | 4.81(4.17-5.46) | 2.00(1.77-2.24) | 0.93(0.79-1.08) | 0.29(0.21-0.38) |
| Tropical Latin America | 70.34(70.24-70.44) | 46.47(46.38-46.57) | 25.38(25.29-25.47) | 17.25(17.17-17.33) | 9.29(9.22-9.36) | 4.35(4.31-4.40) | 1.88(1.86-1.90) | 0.92(0.90-0.93) | 0.31(0.30-0.32) |
| Tunisia | 63.47(62.97-63.98) | 52.44(51.95-52.94) | 33.21(32.74-33.67) | 20.15(19.72-20.57) | 10.55(10.18-10.93) | 5.22(4.95-5.49) | 2.27(2.16-2.38) | 1.09(1.02-1.15) | 0.32(0.28-0.36) |
| Turkey | 64.09(63.91-64.27) | 52.66(52.48-52.83) | 33.45(33.28-33.62) | 20.37(20.21-20.52) | 10.73(10.58-10.87) | 5.35(5.24-5.46) | 2.37(2.32-2.41) | 1.16(1.14-1.19) | 0.35(0.33-0.36) |
| Turkmenistan | 59.29(58.35-60.23) | 50.83(49.91-51.74) | 31.98(31.11-32.84) | 20.37(19.62-21.12) | 10.98(10.37-11.59) | 5.09(4.72-5.47) | 1.92(1.78-2.07) | 0.79(0.69-0.88) | 0.20(0.16-0.25) |
| Tuvalu | 59.70(38.67-80.72) | 51.11(30.56-71.66) | 32.04(12.55-51.52) | 20.47(3.25-37.69) | 11.38(-2.41-25.17) | 5.61(-2.55-13.77) | 2.14(-0.98-5.26) | 0.86(-0.87-2.59) | 0.21(-0.71-1.13) |
| Uganda | 42.26(41.79-42.72) | 37.40(36.95-37.85) | 25.49(25.06-25.91) | 16.37(16.01-16.74) | 9.28(9.00-9.56) | 4.63(4.46-4.79) | 1.90(1.83-1.98) | 0.81(0.76-0.86) | 0.21(0.18-0.23) |
| Ukraine | 60.78(60.48-61.09) | 53.12(52.83-53.41) | 34.03(33.76-34.30) | 21.70(21.47-21.93) | 11.69(11.50-11.88) | 5.70(5.58-5.82) | 2.32(2.28-2.36) | 0.99(0.97-1.01) | 0.27(0.26-0.28) |
| United Arab Emirates | 63.32(62.52-64.11) | 52.23(51.45-53.02) | 32.90(32.14-33.66) | 19.77(19.09-20.44) | 10.11(9.47-10.75) | 4.71(4.14-5.27) | 1.68(1.38-1.98) | 0.45(0.30-0.59) | 0.03(-0.01-0.06) |
| United Kingdom | 70.37(70.21-70.52) | 59.15(58.99-59.30) | 31.88(31.74-32.03) | 18.78(18.64-18.92) | 9.88(9.75-10.01) | 4.38(4.28-4.47) | 1.81(1.77-1.86) | 0.98(0.96-1.00) | 0.42(0.40-0.43) |
| United Republic of Tanzania | 44.09(43.71-44.47) | 38.78(38.41-39.14) | 26.26(25.92-26.60) | 16.75(16.45-17.04) | 9.48(9.26-9.71) | 4.74(4.61-4.87) | 1.93(1.87-1.99) | 0.84(0.80-0.87) | 0.22(0.20-0.24) |
| United States Virgin Islands | 63.70(57.35-70.05) | 49.16(42.84-55.47) | 29.57(23.29-35.84) | 20.08(14.74-25.41) | 11.19(6.81-15.57) | 5.52(2.58-8.46) | 2.54(1.19-3.90) | 1.34(0.65-2.04) | 0.52(0.06-0.97) |
| United States of America | 69.80(69.72-69.88) | 60.37(60.29-60.45) | 32.74(32.66-32.81) | 19.10(19.03-19.17) | 9.87(9.81-9.93) | 4.47(4.43-4.52) | 1.83(1.81-1.84) | 0.92(0.91-0.93) | 0.37(0.36-0.37) |
| Uruguay | 57.26(56.15-58.37) | 50.39(49.30-51.47) | 30.99(29.95-32.03) | 19.54(18.59-20.48) | 10.85(10.04-11.66) | 5.25(4.70-5.81) | 2.19(2.00-2.39) | 1.10(1.00-1.20) | 0.43(0.37-0.49) |
| Uzbekistan | 60.23(59.89-60.57) | 51.72(51.39-52.05) | 32.78(32.47-33.09) | 21.07(20.79-21.35) | 11.55(11.31-11.80) | 5.53(5.35-5.70) | 2.21(2.14-2.28) | 0.97(0.92-1.02) | 0.28(0.25-0.30) |
| Vanuatu | 59.20(55.15-63.25) | 50.66(46.70-54.61) | 31.71(27.98-35.44) | 20.22(16.90-23.54) | 11.19(8.53-13.86) | 5.48(3.81-7.14) | 2.05(1.33-2.76) | 0.80(0.35-1.24) | 0.18(-0.06-0.42) |
| Venezuela (Bolivarian Republic of) | 62.53(62.16-62.90) | 48.43(48.07-48.79) | 29.73(29.40-30.07) | 19.18(18.90-19.46) | 10.21(9.98-10.44) | 4.73(4.58-4.88) | 2.02(1.96-2.08) | 0.92(0.88-0.96) | 0.28(0.26-0.30) |
| Viet Nam | 64.70(64.52-64.87) | 54.97(54.81-55.14) | 33.75(33.59-33.91) | 21.30(21.16-21.45) | 11.83(11.70-11.96) | 6.16(6.06-6.26) | 2.67(2.62-2.72) | 1.22(1.20-1.25) | 0.39(0.37-0.40) |
| Western Europe | 70.43(70.37-70.49) | 58.30(58.24-58.36) | 31.30(31.25-31.36) | 18.48(18.43-18.53) | 9.83(9.79-9.88) | 4.40(4.36-4.43) | 1.87(1.85-1.89) | 1.05(1.04-1.05) | 0.47(0.47-0.48) |
| Western Sub-Saharan Africa | 54.48(54.37-54.58) | 45.56(45.46-45.66) | 27.71(27.61-27.80) | 17.61(17.52-17.69) | 9.72(9.65-9.79) | 4.65(4.61-4.69) | 1.73(1.71-1.75) | 0.75(0.74-0.76) | 0.18(0.18-0.19) |
| Yemen | 60.11(59.73-60.49) | 49.42(49.05-49.79) | 30.84(30.49-31.18) | 18.36(18.05-18.66) | 9.29(9.03-9.54) | 4.32(4.15-4.49) | 1.67(1.60-1.74) | 0.69(0.65-0.74) | 0.16(0.14-0.18) |
| Zambia | 43.19(42.56-43.81) | 37.80(37.20-38.40) | 25.13(24.58-25.68) | 15.64(15.20-16.09) | 8.53(8.22-8.84) | 4.00(3.83-4.18) | 1.45(1.36-1.53) | 0.53(0.48-0.58) | 0.10(0.08-0.12) |
| Zimbabwe | 49.30(48.71-49.89) | 42.05(41.48-42.62) | 25.96(25.45-26.47) | 15.91(15.52-16.30) | 8.22(7.97-8.46) | 3.50(3.37-3.62) | 1.04(0.99-1.09) | 0.33(0.30-0.35) | 0.04(0.04-0.05) |

Table. S4 Female-to-male ratios of migraine lifetime risk with 95% confidence intervals, 2021

| **Location name** | **Female-to-male ratio 1990** | **Female-to-male ratio 2021** |
| --- | --- | --- |
| **Global** | 1.46(1.46-1.47) | 1.42(1.42-1.43) |
| **High SDI** | 1.61(1.60-1.61) | 1.57(1.56-1.57) |
| **High-middle SDI** | 1.54(1.54-1.55) | 1.50(1.50-1.50) |
| **Middle SDI** | 1.46(1.46-1.47) | 1.43(1.42-1.43) |
| **Low-middle SDI** | 1.34(1.34-1.35) | 1.35(1.35-1.35) |
| **Low SDI** | 1.36(1.36-1.37) | 1.37(1.37-1.37) |
| **Andean Latin America** | 1.77(1.73-1.81) | 1.75(1.73-1.77) |
| **Bolivia (Plurinational State of)** | 1.73(1.65-1.82) | 1.75(1.69-1.80) |
| **Ecuador** | 1.68(1.62-1.74) | 1.67(1.63-1.71) |
| **Peru** | 1.83(1.77-1.90) | 1.80(1.76-1.84) |
| **Australasia** | 1.42(1.40-1.45) | 1.40(1.38-1.42) |
| **Australia** | 1.42(1.40-1.45) | 1.40(1.38-1.42) |
| **New Zealand** | 1.42(1.36-1.48) | 1.39(1.35-1.44) |
| **Caribbean** | 1.55(1.53-1.58) | 1.56(1.54-1.58) |
| **Antigua and Barbuda** | 1.60(1.12-2.27) | 1.56(1.21-2.02) |
| **Bahamas** | 1.57(1.34-1.85) | 1.58(1.39-1.79) |
| **Barbados** | 1.56(1.32-1.84) | 1.54(1.32-1.79) |
| **Belize** | 1.57(1.24-1.98) | 1.56(1.38-1.77) |
| **Cuba** | 1.54(1.50-1.58) | 1.55(1.51-1.58) |
| **Dominica** | 1.60(1.15-2.22) | 1.60(1.16-2.21) |
| **Dominican Republic** | 1.55(1.50-1.61) | 1.56(1.52-1.60) |
| **Grenada** | 1.59(1.16-2.18) | 1.58(1.23-2.02) |
| **Guyana** | 1.60(1.45-1.76) | 1.60(1.46-1.75) |
| **Haiti** | 1.56(1.51-1.62) | 1.56(1.53-1.60) |
| **Jamaica** | 1.53(1.44-1.62) | 1.54(1.47-1.61) |
| **Saint Lucia** | 1.58(1.24-2.02) | 1.57(1.30-1.91) |
| **Saint Vincent and the Grenadines** | 1.58(1.19-2.09) | 1.57(1.23-2.01) |
| **Suriname** | 1.58(1.37-1.81) | 1.57(1.40-1.75) |
| **Trinidad and Tobago** | 1.56(1.45-1.69) | 1.57(1.46-1.69) |
| **Bermuda** | 1.60(1.17-2.20) | 1.58(1.15-2.18) |
| **Puerto Rico** | 1.57(1.51-1.64) | 1.55(1.48-1.62) |
| **Saint Kitts and Nevis** | 1.62(1.03-2.54) | 1.60(1.15-2.25) |
| **United States Virgin Islands** | 1.60(1.23-2.09) | 1.63(1.20-2.22) |
| **Central Asia** | 1.54(1.52-1.56) | 1.52(1.51-1.53) |
| **Armenia** | 1.52(1.45-1.59) | 1.51(1.44-1.58) |
| **Azerbaijan** | 1.54(1.49-1.59) | 1.52(1.48-1.56) |
| **Georgia** | 1.54(1.48-1.60) | 1.53(1.46-1.60) |
| **Kazakhstan** | 1.56(1.52-1.59) | 1.54(1.51-1.56) |
| **Kyrgyzstan** | 1.55(1.48-1.62) | 1.52(1.47-1.57) |
| **Mongolia** | 1.54(1.44-1.64) | 1.54(1.47-1.61) |
| **Tajikistan** | 1.52(1.45-1.59) | 1.52(1.48-1.56) |
| **Turkmenistan** | 1.54(1.46-1.62) | 1.53(1.48-1.59) |
| **Uzbekistan** | 1.53(1.49-1.56) | 1.50(1.48-1.52) |
| **Central Europe** | 1.58(1.57-1.59) | 1.55(1.54-1.56) |
| **Albania** | 1.55(1.47-1.63) | 1.54(1.46-1.62) |
| **Bosnia and Herzegovina** | 1.56(1.50-1.62) | 1.54(1.48-1.61) |
| **Bulgaria** | 1.58(1.53-1.63) | 1.57(1.52-1.63) |
| **Croatia** | 1.57(1.51-1.63) | 1.55(1.49-1.61) |
| **Czechia** | 1.58(1.53-1.62) | 1.54(1.50-1.58) |
| **Hungary** | 1.59(1.55-1.64) | 1.55(1.51-1.59) |
| **North Macedonia** | 1.55(1.46-1.64) | 1.55(1.47-1.64) |
| **Montenegro** | 1.56(1.40-1.73) | 1.56(1.41-1.73) |
| **Poland** | 1.58(1.56-1.61) | 1.55(1.53-1.57) |
| **Romania** | 1.58(1.55-1.61) | 1.56(1.53-1.59) |
| **Serbia** | 1.58(1.53-1.62) | 1.54(1.51-1.58) |
| **Slovakia** | 1.58(1.53-1.64) | 1.55(1.50-1.61) |
| **Slovenia** | 1.57(1.48-1.67) | 1.53(1.45-1.61) |
| **Central Latin America** | 1.71(1.69-1.72) | 1.69(1.68-1.69) |
| **Colombia** | 1.72(1.70-1.75) | 1.67(1.65-1.69) |
| **Costa Rica** | 1.65(1.56-1.75) | 1.66(1.60-1.73) |
| **El Salvador** | 1.73(1.66-1.80) | 1.71(1.65-1.76) |
| **Guatemala** | 1.71(1.65-1.77) | 1.69(1.66-1.73) |
| **Honduras** | 1.67(1.59-1.76) | 1.67(1.63-1.72) |
| **Mexico** | 1.71(1.69-1.72) | 1.69(1.68-1.70) |
| **Nicaragua** | 1.68(1.58-1.77) | 1.67(1.62-1.73) |
| **Panama** | 1.66(1.55-1.76) | 1.65(1.58-1.73) |
| **Venezuela (Bolivarian Republic of)** | 1.70(1.66-1.74) | 1.73(1.70-1.76) |
| **Central Sub-Saharan Africa** | 1.39(1.37-1.41) | 1.38(1.37-1.39) |
| **Angola** | 1.42(1.37-1.47) | 1.37(1.35-1.40) |
| **Central African Republic** | 1.43(1.34-1.52) | 1.43(1.38-1.50) |
| **Congo** | 1.39(1.31-1.49) | 1.35(1.30-1.41) |
| **Democratic Republic of the Congo** | 1.38(1.35-1.40) | 1.38(1.36-1.39) |
| **Equatorial Guinea** | 1.42(1.20-1.68) | 1.36(1.26-1.47) |
| **Gabon** | 1.41(1.27-1.57) | 1.38(1.29-1.48) |
| **East Asia** | 1.56(1.55-1.56) | 1.51(1.50-1.51) |
| **China** | 1.56(1.55-1.56) | 1.51(1.50-1.51) |
| **Democratic People's Republic of Korea** | 1.52(1.48-1.55) | 1.51(1.48-1.54) |
| **Taiwan (Province of China)** | 1.55(1.52-1.58) | 1.45(1.43-1.48) |
| **Eastern Europe** | 1.63(1.62-1.64) | 1.61(1.60-1.62) |
| **Belarus** | 1.69(1.64-1.75) | 1.69(1.63-1.74) |
| **Estonia** | 1.71(1.57-1.86) | 1.65(1.51-1.79) |
| **Latvia** | 1.71(1.60-1.83) | 1.67(1.56-1.80) |
| **Lithuania** | 1.80(1.70-1.91) | 1.77(1.66-1.89) |
| **Republic of Moldova** | 1.69(1.61-1.77) | 1.67(1.59-1.76) |
| **Russian Federation** | 1.61(1.59-1.62) | 1.58(1.57-1.59) |
| **Ukraine** | 1.68(1.66-1.70) | 1.68(1.65-1.70) |
| **Eastern Sub-Saharan Africa** | 1.52(1.50-1.53) | 1.49(1.48-1.50) |
| **Burundi** | 1.50(1.42-1.59) | 1.48(1.42-1.54) |
| **Comoros** | 1.48(1.18-1.85) | 1.46(1.24-1.70) |
| **Djibouti** | 1.50(1.19-1.87) | 1.47(1.31-1.64) |
| **Eritrea** | 1.71(1.60-1.82) | 1.50(1.42-1.58) |
| **Ethiopia** | 1.55(1.52-1.58) | 1.47(1.45-1.49) |
| **Kenya** | 1.44(1.40-1.49) | 1.48(1.45-1.50) |
| **Madagascar** | 1.47(1.41-1.53) | 1.46(1.42-1.50) |
| **Malawi** | 1.48(1.42-1.55) | 1.50(1.45-1.54) |
| **Mozambique** | 1.51(1.45-1.57) | 1.50(1.47-1.54) |
| **Rwanda** | 1.51(1.43-1.58) | 1.47(1.42-1.53) |
| **Somalia** | 1.53(1.45-1.60) | 1.53(1.48-1.57) |
| **United Republic of Tanzania** | 1.57(1.53-1.62) | 1.55(1.52-1.58) |
| **Uganda** | 1.51(1.46-1.55) | 1.50(1.47-1.53) |
| **Zambia** | 1.40(1.34-1.47) | 1.43(1.39-1.47) |
| **South Sudan** | 1.52(1.43-1.61) | 1.51(1.44-1.58) |
| **High-income Asia Pacific** | 1.78(1.77-1.80) | 1.69(1.67-1.70) |
| **Brunei Darussalam** | 1.72(1.36-2.19) | 1.68(1.46-1.94) |
| **Japan** | 1.85(1.83-1.87) | 1.72(1.70-1.73) |
| **Republic of Korea** | 1.67(1.64-1.69) | 1.63(1.61-1.65) |
| **Singapore** | 1.73(1.60-1.86) | 1.63(1.56-1.70) |
| **High-income North America** | 1.67(1.67-1.68) | 1.67(1.66-1.67) |
| **Canada** | 1.80(1.77-1.83) | 1.77(1.75-1.79) |
| **United States of America** | 1.66(1.65-1.67) | 1.66(1.65-1.67) |
| **Greenland** | 1.83(1.29-2.60) | 1.79(1.27-2.56) |
| **North Africa and Middle East** | 1.43(1.42-1.44) | 1.43(1.42-1.43) |
| **Algeria** | 1.41(1.39-1.44) | 1.40(1.39-1.42) |
| **Bahrain** | 1.41(1.27-1.57) | 1.40(1.33-1.47) |
| **Egypt** | 1.40(1.39-1.42) | 1.44(1.43-1.45) |
| **Iran (Islamic Republic of)** | 1.41(1.39-1.42) | 1.43(1.42-1.44) |
| **Iraq** | 1.44(1.41-1.47) | 1.42(1.41-1.44) |
| **Jordan** | 1.39(1.33-1.46) | 1.40(1.37-1.43) |
| **Kuwait** | 1.39(1.30-1.48) | 1.36(1.32-1.41) |
| **Lebanon** | 1.44(1.38-1.51) | 1.41(1.37-1.45) |
| **Libya** | 1.41(1.35-1.47) | 1.42(1.38-1.46) |
| **Morocco** | 1.41(1.39-1.44) | 1.41(1.39-1.42) |
| **Palestine** | 1.42(1.33-1.51) | 1.41(1.36-1.46) |
| **Oman** | 1.43(1.35-1.52) | 1.41(1.37-1.46) |
| **Qatar** | 1.42(1.26-1.60) | 1.39(1.33-1.46) |
| **Saudi Arabia** | 1.37(1.34-1.40) | 1.35(1.34-1.37) |
| **Syrian Arab Republic** | 1.41(1.38-1.45) | 1.42(1.39-1.45) |
| **Tunisia** | 1.41(1.37-1.45) | 1.42(1.39-1.44) |
| **Turkey** | 1.51(1.50-1.53) | 1.48(1.47-1.49) |
| **United Arab Emirates** | 1.41(1.33-1.49) | 1.37(1.33-1.41) |
| **Yemen** | 1.43(1.39-1.47) | 1.45(1.43-1.47) |
| **Afghanistan** | 1.41(1.36-1.45) | 1.47(1.45-1.49) |
| **Sudan** | 1.43(1.40-1.46) | 1.42(1.40-1.44) |
| **Oceania** | 1.35(1.30-1.39) | 1.35(1.32-1.38) |
| **Fiji** | 1.35(1.23-1.48) | 1.35(1.25-1.46) |
| **Kiribati** | 1.38(1.00-1.90) | 1.38(1.09-1.74) |
| **Marshall Islands** | 1.43(0.92-2.15) | 1.38(0.98-1.91) |
| **Micronesia (Federated States of)** | 1.38(1.05-1.80) | 1.37(1.07-1.74) |
| **Papua New Guinea** | 1.34(1.28-1.40) | 1.35(1.31-1.38) |
| **Samoa** | 1.36(1.10-1.68) | 1.34(1.12-1.59) |
| **Solomon Islands** | 1.37(1.18-1.59) | 1.36(1.23-1.49) |
| **Tonga** | 1.36(1.03-1.79) | 1.35(1.06-1.73) |
| **Vanuatu** | 1.38(1.11-1.72) | 1.37(1.19-1.58) |
| **American Samoa** | 1.39(0.95-2.02) | 1.37(0.96-1.95) |
| **Cook Islands** | 1.47(0.74-2.79) | 1.46(0.79-2.67) |
| **Guam** | 1.35(1.09-1.66) | 1.36(1.13-1.63) |
| **Nauru** | 1.64(0.61-3.98) | 1.59(0.66-3.56) |
| **Niue** | -7.09(-16.94-17.44) | -3.85(-12.85-13.62) |
| **Northern Mariana Islands** | 1.37(0.94-1.95) | 1.38(0.97-1.93) |
| **Palau** | 1.49(0.72-2.91) | 1.43(0.73-2.62) |
| **Tokelau** | 1.41(-10.86-11.37) | 0.75(-12.00-12.01) |
| **Tuvalu** | 1.81(0.58-5.34) | 1.54(0.68-3.26) |
| **South Asia** | 1.29(1.29-1.30) | 1.30(1.30-1.31) |
| **Bangladesh** | 1.28(1.27-1.29) | 1.27(1.27-1.28) |
| **Bhutan** | 1.27(1.13-1.41) | 1.27(1.17-1.38) |
| **India** | 1.30(1.30-1.31) | 1.32(1.31-1.32) |
| **Nepal** | 1.25(1.23-1.27) | 1.27(1.25-1.28) |
| **Pakistan** | 1.25(1.24-1.26) | 1.26(1.25-1.26) |
| **Southeast Asia** | 1.35(1.34-1.35) | 1.33(1.33-1.34) |
| **Cambodia** | 1.36(1.33-1.40) | 1.34(1.32-1.36) |
| **Indonesia** | 1.32(1.32-1.33) | 1.32(1.32-1.33) |
| **Lao People's Democratic Republic** | 1.37(1.31-1.43) | 1.34(1.30-1.37) |
| **Malaysia** | 1.30(1.27-1.32) | 1.29(1.28-1.31) |
| **Maldives** | 1.31(1.10-1.57) | 1.31(1.20-1.42) |
| **Myanmar** | 1.39(1.37-1.40) | 1.35(1.34-1.36) |
| **Philippines** | 1.35(1.34-1.36) | 1.34(1.33-1.35) |
| **Sri Lanka** | 1.36(1.34-1.39) | 1.32(1.31-1.34) |
| **Thailand** | 1.41(1.40-1.42) | 1.36(1.35-1.38) |
| **Timor-Leste** | 1.33(1.21-1.46) | 1.33(1.24-1.41) |
| **Viet Nam** | 1.36(1.34-1.37) | 1.34(1.33-1.35) |
| **Mauritius** | 1.34(1.26-1.43) | 1.33(1.26-1.41) |
| **Seychelles** | 1.37(1.04-1.78) | 1.32(1.10-1.58) |
| **Southern Latin America** | 1.63(1.60-1.65) | 1.60(1.58-1.62) |
| **Argentina** | 1.57(1.54-1.60) | 1.55(1.53-1.57) |
| **Chile** | 1.76(1.71-1.81) | 1.72(1.68-1.76) |
| **Uruguay** | 1.65(1.56-1.76) | 1.65(1.56-1.74) |
| **Southern Sub-Saharan Africa** | 1.38(1.36-1.40) | 1.39(1.37-1.40) |
| **Botswana** | 1.39(1.27-1.52) | 1.38(1.30-1.46) |
| **Lesotho** | 1.42(1.30-1.55) | 1.44(1.35-1.54) |
| **Namibia** | 1.39(1.27-1.52) | 1.40(1.32-1.48) |
| **South Africa** | 1.38(1.36-1.40) | 1.38(1.37-1.40) |
| **Eswatini** | 1.42(1.26-1.60) | 1.41(1.30-1.53) |
| **Zimbabwe** | 1.37(1.32-1.41) | 1.40(1.36-1.43) |
| **Tropical Latin America** | 1.52(1.51-1.53) | 1.47(1.47-1.48) |
| **Brazil** | 1.52(1.51-1.53) | 1.47(1.46-1.48) |
| **Paraguay** | 1.48(1.43-1.53) | 1.50(1.46-1.53) |
| **Western Europe** | 1.49(1.49-1.50) | 1.47(1.46-1.47) |
| **Andorra** | 1.50(1.17-1.92) | 1.48(1.24-1.78) |
| **Austria** | 1.52(1.48-1.55) | 1.50(1.47-1.52) |
| **Belgium** | 1.44(1.42-1.47) | 1.42(1.40-1.44) |
| **Cyprus** | 1.49(1.39-1.59) | 1.47(1.41-1.54) |
| **Denmark** | 1.54(1.49-1.59) | 1.51(1.48-1.55) |
| **Finland** | 1.50(1.46-1.54) | 1.47(1.44-1.51) |
| **France** | 1.46(1.45-1.47) | 1.44(1.43-1.45) |
| **Germany** | 1.49(1.48-1.50) | 1.46(1.45-1.47) |
| **Greece** | 1.45(1.42-1.47) | 1.45(1.42-1.47) |
| **Iceland** | 1.49(1.31-1.69) | 1.47(1.33-1.62) |
| **Ireland** | 1.49(1.44-1.54) | 1.47(1.43-1.50) |
| **Israel** | 1.48(1.44-1.52) | 1.47(1.44-1.50) |
| **Italy** | 1.49(1.47-1.50) | 1.46(1.45-1.47) |
| **Luxembourg** | 1.49(1.34-1.65) | 1.46(1.37-1.55) |
| **Malta** | 1.48(1.35-1.63) | 1.47(1.35-1.60) |
| **Netherlands** | 1.55(1.52-1.57) | 1.49(1.47-1.51) |
| **Norway** | 1.52(1.48-1.57) | 1.46(1.43-1.50) |
| **Portugal** | 1.50(1.47-1.54) | 1.47(1.45-1.50) |
| **Spain** | 1.48(1.46-1.49) | 1.46(1.44-1.47) |
| **Sweden** | 1.53(1.50-1.56) | 1.50(1.48-1.53) |
| **Switzerland** | 1.53(1.49-1.57) | 1.49(1.47-1.52) |
| **United Kingdom** | 1.52(1.51-1.54) | 1.50(1.49-1.51) |
| **Monaco** | 1.54(1.06-2.26) | 1.53(1.08-2.17) |
| **San Marino** | 1.54(1.04-2.31) | 1.50(1.10-2.05) |
| **Western Sub-Saharan Africa** | 1.33(1.32-1.33) | 1.32(1.32-1.33) |
| **Benin** | 1.33(1.27-1.39) | 1.33(1.30-1.36) |
| **Burkina Faso** | 1.33(1.29-1.37) | 1.33(1.31-1.36) |
| **Cameroon** | 1.32(1.28-1.36) | 1.33(1.31-1.35) |
| **Cabo Verde** | 1.32(1.12-1.56) | 1.32(1.20-1.46) |
| **Chad** | 1.33(1.28-1.38) | 1.33(1.30-1.36) |
| **Côte d’Ivoire** | 1.34(1.31-1.38) | 1.33(1.31-1.35) |
| **Gambia** | 1.35(1.23-1.49) | 1.32(1.25-1.39) |
| **Ghana** | 1.31(1.28-1.34) | 1.32(1.30-1.34) |
| **Guinea** | 1.30(1.25-1.35) | 1.32(1.29-1.36) |
| **Guinea-Bissau** | 1.40(1.28-1.54) | 1.35(1.27-1.43) |
| **Liberia** | 1.42(1.33-1.50) | 1.31(1.26-1.35) |
| **Mali** | 1.32(1.28-1.36) | 1.33(1.31-1.36) |
| **Mauritania** | 1.30(1.22-1.39) | 1.30(1.24-1.35) |
| **Niger** | 1.30(1.26-1.34) | 1.31(1.29-1.34) |
| **Nigeria** | 1.33(1.32-1.35) | 1.31(1.31-1.32) |
| **Sao Tome and Principe** | 1.33(1.00-1.76) | 1.31(1.10-1.54) |
| **Senegal** | 1.33(1.28-1.38) | 1.31(1.29-1.34) |
| **Sierra Leone** | 1.34(1.28-1.40) | 1.32(1.28-1.36) |
| **Togo** | 1.32(1.26-1.39) | 1.33(1.29-1.37) |

Table S5 Age-specific female-to-male ratios of migraine lifetime risk with 95% confidence intervals, 2021

| Age | Location name | Female-to-male ratios |
| --- | --- | --- |
| 5 | Low-middle SDI | 1.61(1.60-1.61) |
| 5 | Middle SDI | 1.70(1.69-1.70) |
| 5 | High-middle SDI | 1.68(1.68-1.69) |
| 5 | High SDI | 1.74(1.74-1.75) |
| 5 | Global | 1.64(1.64-1.64) |
| 5 | Low SDI | 1.54(1.53-1.55) |
| 5 | High-income North America | 2.01(1.99-2.02) |
| 5 | North Africa and Middle East | 1.31(1.30-1.31) |
| 5 | Oceania | 1.60(1.52-1.69) |
| 5 | South Asia | 1.68(1.67-1.68) |
| 5 | Southeast Asia | 1.61(1.60-1.62) |
| 5 | Southern Latin America | 1.73(1.69-1.76) |
| 5 | Southern Sub-Saharan Africa | 1.54(1.50-1.59) |
| 5 | Tropical Latin America | 1.80(1.80-1.80) |
| 5 | Western Europe | 1.71(1.71-1.72) |
| 5 | Andean Latin America | 2.08(2.05-2.12) |
| 5 | Western Sub-Saharan Africa | 1.51(1.50-1.52) |
| 5 | Central Asia | 1.80(1.77-1.84) |
| 5 | Central Europe | 1.78(1.76-1.80) |
| 5 | Central Latin America | 1.92(1.91-1.94) |
| 5 | Australasia | 1.39(1.37-1.42) |
| 5 | Central Sub-Saharan Africa | 1.55(1.52-1.58) |
| 5 | East Asia | 1.78(1.76-1.79) |
| 5 | Eastern Europe | 1.76(1.74-1.78) |
| 5 | Eastern Sub-Saharan Africa | 1.52(1.50-1.55) |
| 5 | Caribbean | 1.94(1.91-1.98) |
| 5 | High-income Asia Pacific | 1.68(1.66-1.70) |
| 10 | Low-middle SDI | 1.41(1.41-1.42) |
| 10 | Middle SDI | 1.50(1.50-1.50) |
| 10 | High-middle SDI | 1.60(1.60-1.61) |
| 10 | High SDI | 1.80(1.79-1.80) |
| 10 | Global | 1.49(1.49-1.49) |
| 10 | Low SDI | 1.42(1.41-1.42) |
| 10 | High-income North America | 2.01(2.01-2.02) |
| 10 | North Africa and Middle East | 1.33(1.33-1.33) |
| 10 | Oceania | 1.43(1.39-1.46) |
| 10 | South Asia | 1.42(1.42-1.42) |
| 10 | Southeast Asia | 1.46(1.45-1.46) |
| 10 | Southern Latin America | 1.77(1.75-1.79) |
| 10 | Southern Sub-Saharan Africa | 1.41(1.39-1.43) |
| 10 | Tropical Latin America | 1.34(1.34-1.35) |
| 10 | Western Europe | 1.72(1.71-1.72) |
| 10 | Andean Latin America | 1.74(1.71-1.76) |
| 10 | Western Sub-Saharan Africa | 1.38(1.38-1.39) |
| 10 | Central Asia | 1.65(1.63-1.66) |
| 10 | Central Europe | 1.65(1.63-1.66) |
| 10 | Central Latin America | 1.62(1.61-1.63) |
| 10 | Australasia | 1.50(1.48-1.51) |
| 10 | Central Sub-Saharan Africa | 1.42(1.41-1.43) |
| 10 | East Asia | 1.62(1.62-1.63) |
| 10 | Eastern Europe | 1.69(1.68-1.70) |
| 10 | Eastern Sub-Saharan Africa | 1.47(1.46-1.48) |
| 10 | Caribbean | 1.57(1.55-1.59) |
| 10 | High-income Asia Pacific | 1.80(1.79-1.82) |
| 15 | Low-middle SDI | 1.27(1.27-1.28) |
| 15 | Middle SDI | 1.37(1.37-1.38) |
| 15 | High-middle SDI | 1.60(1.59-1.61) |
| 15 | High SDI | 1.91(1.90-1.92) |
| 15 | Global | 1.42(1.41-1.42) |
| 15 | Low SDI | 1.33(1.32-1.34) |
| 15 | High-income North America | 1.93(1.92-1.95) |
| 15 | North Africa and Middle East | 1.67(1.65-1.68) |
| 15 | Oceania | 1.30(1.23-1.37) |
| 15 | South Asia | 1.19(1.19-1.20) |
| 15 | Southeast Asia | 1.35(1.34-1.35) |
| 15 | Southern Latin America | 1.84(1.79-1.89) |
| 15 | Southern Sub-Saharan Africa | 1.33(1.29-1.37) |
| 15 | Tropical Latin America | 1.17(1.15-1.20) |
| 15 | Western Europe | 1.94(1.93-1.95) |
| 15 | Andean Latin America | 1.46(1.39-1.54) |
| 15 | Western Sub-Saharan Africa | 1.29(1.28-1.30) |
| 15 | Central Asia | 1.56(1.53-1.59) |
| 15 | Central Europe | 1.63(1.60-1.66) |
| 15 | Central Latin America | 1.51(1.48-1.54) |
| 15 | Australasia | 1.68(1.64-1.73) |
| 15 | Central Sub-Saharan Africa | 1.34(1.31-1.37) |
| 15 | East Asia | 1.48(1.48-1.49) |
| 15 | Eastern Europe | 1.66(1.63-1.69) |
| 15 | Eastern Sub-Saharan Africa | 1.46(1.43-1.49) |
| 15 | Caribbean | 1.33(1.27-1.39) |
| 15 | High-income Asia Pacific | 1.96(1.93-1.99) |
| 20 | Low-middle SDI | 1.28(1.28-1.29) |
| 20 | Middle SDI | 1.38(1.37-1.39) |
| 20 | High-middle SDI | 1.55(1.54-1.57) |
| 20 | High SDI | 1.67(1.66-1.69) |
| 20 | Global | 1.41(1.40-1.41) |
| 20 | Low SDI | 1.35(1.34-1.37) |
| 20 | High-income North America | 1.68(1.66-1.71) |
| 20 | North Africa and Middle East | 1.83(1.80-1.85) |
| 20 | Oceania | 1.28(1.19-1.38) |
| 20 | South Asia | 1.19(1.18-1.19) |
| 20 | Southeast Asia | 1.29(1.28-1.30) |
| 20 | Southern Latin America | 1.67(1.61-1.73) |
| 20 | Southern Sub-Saharan Africa | 1.35(1.29-1.40) |
| 20 | Tropical Latin America | 1.30(1.27-1.33) |
| 20 | Western Europe | 1.60(1.59-1.61) |
| 20 | Andean Latin America | 1.61(1.51-1.71) |
| 20 | Western Sub-Saharan Africa | 1.30(1.29-1.32) |
| 20 | Central Asia | 1.57(1.52-1.62) |
| 20 | Central Europe | 1.71(1.66-1.76) |
| 20 | Central Latin America | 1.71(1.66-1.75) |
| 20 | Australasia | 1.56(1.50-1.62) |
| 20 | Central Sub-Saharan Africa | 1.36(1.32-1.40) |
| 20 | East Asia | 1.47(1.46-1.49) |
| 20 | Eastern Europe | 1.67(1.63-1.72) |
| 20 | Eastern Sub-Saharan Africa | 1.51(1.48-1.55) |
| 20 | Caribbean | 1.41(1.33-1.50) |
| 20 | High-income Asia Pacific | 1.81(1.77-1.85) |
| 25 | Low-middle SDI | 1.33(1.32-1.33) |
| 25 | Middle SDI | 1.42(1.41-1.43) |
| 25 | High-middle SDI | 1.50(1.49-1.51) |
| 25 | High SDI | 1.40(1.39-1.41) |
| 25 | Global | 1.40(1.40-1.41) |
| 25 | Low SDI | 1.38(1.36-1.39) |
| 25 | High-income North America | 1.42(1.40-1.44) |
| 25 | North Africa and Middle East | 1.89(1.86-1.91) |
| 25 | Oceania | 1.30(1.20-1.41) |
| 25 | South Asia | 1.22(1.21-1.23) |
| 25 | Southeast Asia | 1.28(1.27-1.29) |
| 25 | Southern Latin America | 1.50(1.44-1.56) |
| 25 | Southern Sub-Saharan Africa | 1.35(1.30-1.40) |
| 25 | Tropical Latin America | 1.49(1.45-1.52) |
| 25 | Western Europe | 1.16(1.15-1.17) |
| 25 | Andean Latin America | 1.77(1.67-1.88) |
| 25 | Western Sub-Saharan Africa | 1.29(1.28-1.31) |
| 25 | Central Asia | 1.57(1.51-1.62) |
| 25 | Central Europe | 1.69(1.64-1.75) |
| 25 | Central Latin America | 1.91(1.86-1.96) |
| 25 | Australasia | 1.32(1.26-1.38) |
| 25 | Central Sub-Saharan Africa | 1.36(1.31-1.40) |
| 25 | East Asia | 1.49(1.48-1.50) |
| 25 | Eastern Europe | 1.70(1.65-1.75) |
| 25 | Eastern Sub-Saharan Africa | 1.54(1.50-1.58) |
| 25 | Caribbean | 1.55(1.46-1.65) |
| 25 | High-income Asia Pacific | 1.63(1.59-1.67) |
| 30 | Low-middle SDI | 1.32(1.31-1.33) |
| 30 | Middle SDI | 1.41(1.40-1.42) |
| 30 | High-middle SDI | 1.46(1.45-1.47) |
| 30 | High SDI | 1.31(1.29-1.32) |
| 30 | Global | 1.39(1.38-1.39) |
| 30 | Low SDI | 1.34(1.33-1.36) |
| 30 | High-income North America | 1.31(1.29-1.34) |
| 30 | North Africa and Middle East | 1.77(1.75-1.79) |
| 30 | Oceania | 1.30(1.18-1.44) |
| 30 | South Asia | 1.22(1.21-1.23) |
| 30 | Southeast Asia | 1.26(1.25-1.27) |
| 30 | Southern Latin America | 1.44(1.37-1.51) |
| 30 | Southern Sub-Saharan Africa | 1.33(1.28-1.38) |
| 30 | Tropical Latin America | 1.58(1.54-1.62) |
| 30 | Western Europe | 1.04(1.03-1.05) |
| 30 | Andean Latin America | 1.87(1.75-2.00) |
| 30 | Western Sub-Saharan Africa | 1.24(1.22-1.27) |
| 30 | Central Asia | 1.50(1.45-1.55) |
| 30 | Central Europe | 1.59(1.54-1.65) |
| 30 | Central Latin America | 1.93(1.88-1.99) |
| 30 | Australasia | 1.24(1.18-1.30) |
| 30 | Central Sub-Saharan Africa | 1.32(1.27-1.38) |
| 30 | East Asia | 1.48(1.47-1.49) |
| 30 | Eastern Europe | 1.68(1.63-1.72) |
| 30 | Eastern Sub-Saharan Africa | 1.50(1.46-1.54) |
| 30 | Caribbean | 1.61(1.51-1.71) |
| 30 | High-income Asia Pacific | 1.60(1.55-1.65) |
| 35 | Low-middle SDI | 1.29(1.28-1.30) |
| 35 | Middle SDI | 1.36(1.35-1.37) |
| 35 | High-middle SDI | 1.41(1.40-1.43) |
| 35 | High SDI | 1.38(1.36-1.39) |
| 35 | Global | 1.37(1.36-1.37) |
| 35 | Low SDI | 1.31(1.29-1.33) |
| 35 | High-income North America | 1.42(1.39-1.44) |
| 35 | North Africa and Middle East | 1.47(1.44-1.49) |
| 35 | Oceania | 1.26(1.14-1.39) |
| 35 | South Asia | 1.23(1.22-1.24) |
| 35 | Southeast Asia | 1.19(1.18-1.21) |
| 35 | Southern Latin America | 1.46(1.39-1.54) |
| 35 | Southern Sub-Saharan Africa | 1.32(1.27-1.36) |
| 35 | Tropical Latin America | 1.57(1.53-1.60) |
| 35 | Western Europe | 1.18(1.17-1.19) |
| 35 | Andean Latin America | 1.93(1.80-2.08) |
| 35 | Western Sub-Saharan Africa | 1.22(1.19-1.24) |
| 35 | Central Asia | 1.45(1.40-1.50) |
| 35 | Central Europe | 1.50(1.45-1.55) |
| 35 | Central Latin America | 1.84(1.79-1.90) |
| 35 | Australasia | 1.31(1.25-1.38) |
| 35 | Central Sub-Saharan Africa | 1.29(1.24-1.34) |
| 35 | East Asia | 1.43(1.42-1.44) |
| 35 | Eastern Europe | 1.60(1.56-1.64) |
| 35 | Eastern Sub-Saharan Africa | 1.46(1.42-1.51) |
| 35 | Caribbean | 1.59(1.49-1.70) |
| 35 | High-income Asia Pacific | 1.73(1.67-1.79) |
| 40 | Low-middle SDI | 1.27(1.26-1.29) |
| 40 | Middle SDI | 1.30(1.29-1.31) |
| 40 | High-middle SDI | 1.35(1.33-1.37) |
| 40 | High SDI | 1.36(1.34-1.38) |
| 40 | Global | 1.33(1.32-1.34) |
| 40 | Low SDI | 1.30(1.28-1.32) |
| 40 | High-income North America | 1.43(1.40-1.47) |
| 40 | North Africa and Middle East | 1.28(1.26-1.31) |
| 40 | Oceania | 1.24(1.10-1.41) |
| 40 | South Asia | 1.24(1.23-1.25) |
| 40 | Southeast Asia | 1.14(1.12-1.16) |
| 40 | Southern Latin America | 1.46(1.36-1.57) |
| 40 | Southern Sub-Saharan Africa | 1.33(1.28-1.39) |
| 40 | Tropical Latin America | 1.46(1.42-1.50) |
| 40 | Western Europe | 1.20(1.18-1.22) |
| 40 | Andean Latin America | 1.85(1.72-2.00) |
| 40 | Western Sub-Saharan Africa | 1.20(1.17-1.23) |
| 40 | Central Asia | 1.36(1.30-1.43) |
| 40 | Central Europe | 1.38(1.33-1.43) |
| 40 | Central Latin America | 1.68(1.62-1.73) |
| 40 | Australasia | 1.32(1.22-1.42) |
| 40 | Central Sub-Saharan Africa | 1.29(1.23-1.35) |
| 40 | East Asia | 1.36(1.34-1.38) |
| 40 | Eastern Europe | 1.48(1.44-1.52) |
| 40 | Eastern Sub-Saharan Africa | 1.46(1.42-1.51) |
| 40 | Caribbean | 1.51(1.40-1.63) |
| 40 | High-income Asia Pacific | 1.73(1.66-1.81) |
| 45 | Low-middle SDI | 1.24(1.22-1.26) |
| 45 | Middle SDI | 1.27(1.26-1.29) |
| 45 | High-middle SDI | 1.30(1.27-1.32) |
| 45 | High SDI | 1.25(1.23-1.28) |
| 45 | Global | 1.29(1.28-1.30) |
| 45 | Low SDI | 1.30(1.28-1.33) |
| 45 | High-income North America | 1.31(1.26-1.35) |
| 45 | North Africa and Middle East | 1.23(1.20-1.26) |
| 45 | Oceania | 1.25(1.08-1.44) |
| 45 | South Asia | 1.21(1.20-1.23) |
| 45 | Southeast Asia | 1.15(1.13-1.17) |
| 45 | Southern Latin America | 1.42(1.29-1.55) |
| 45 | Southern Sub-Saharan Africa | 1.37(1.31-1.43) |
| 45 | Tropical Latin America | 1.26(1.22-1.31) |
| 45 | Western Europe | 1.09(1.05-1.12) |
| 45 | Andean Latin America | 1.64(1.50-1.78) |
| 45 | Western Sub-Saharan Africa | 1.24(1.21-1.28) |
| 45 | Central Asia | 1.26(1.19-1.34) |
| 45 | Central Europe | 1.25(1.19-1.31) |
| 45 | Central Latin America | 1.45(1.40-1.50) |
| 45 | Australasia | 1.25(1.10-1.41) |
| 45 | Central Sub-Saharan Africa | 1.32(1.26-1.39) |
| 45 | East Asia | 1.37(1.35-1.39) |
| 45 | Eastern Europe | 1.36(1.32-1.40) |
| 45 | Eastern Sub-Saharan Africa | 1.46(1.41-1.51) |
| 45 | Caribbean | 1.37(1.25-1.49) |
| 45 | High-income Asia Pacific | 1.60(1.50-1.70) |
| 50 | Low-middle SDI | 1.21(1.19-1.22) |
| 50 | Middle SDI | 1.27(1.25-1.28) |
| 50 | High-middle SDI | 1.30(1.28-1.32) |
| 50 | High SDI | 1.21(1.19-1.24) |
| 50 | Global | 1.28(1.27-1.29) |
| 50 | Low SDI | 1.29(1.26-1.31) |
| 50 | High-income North America | 1.22(1.18-1.26) |
| 50 | North Africa and Middle East | 1.16(1.13-1.19) |
| 50 | Oceania | 1.25(1.10-1.43) |
| 50 | South Asia | 1.18(1.16-1.19) |
| 50 | Southeast Asia | 1.16(1.14-1.18) |
| 50 | Southern Latin America | 1.38(1.26-1.52) |
| 50 | Southern Sub-Saharan Africa | 1.42(1.36-1.48) |
| 50 | Tropical Latin America | 1.16(1.12-1.20) |
| 50 | Western Europe | 1.08(1.04-1.12) |
| 50 | Andean Latin America | 1.45(1.33-1.58) |
| 50 | Western Sub-Saharan Africa | 1.26(1.23-1.30) |
| 50 | Central Asia | 1.22(1.15-1.29) |
| 50 | Central Europe | 1.20(1.15-1.26) |
| 50 | Central Latin America | 1.30(1.25-1.34) |
| 50 | Australasia | 1.22(1.06-1.41) |
| 50 | Central Sub-Saharan Africa | 1.33(1.27-1.39) |
| 50 | East Asia | 1.42(1.40-1.44) |
| 50 | Eastern Europe | 1.31(1.27-1.36) |
| 50 | Eastern Sub-Saharan Africa | 1.50(1.46-1.55) |
| 50 | Caribbean | 1.23(1.14-1.33) |
| 50 | High-income Asia Pacific | 1.49(1.39-1.59) |
| 55 | Low-middle SDI | 1.13(1.12-1.15) |
| 55 | Middle SDI | 1.23(1.21-1.24) |
| 55 | High-middle SDI | 1.28(1.26-1.30) |
| 55 | High SDI | 1.21(1.18-1.23) |
| 55 | Global | 1.25(1.24-1.25) |
| 55 | Low SDI | 1.24(1.22-1.26) |
| 55 | High-income North America | 1.25(1.21-1.29) |
| 55 | North Africa and Middle East | 0.95(0.92-0.97) |
| 55 | Oceania | 1.26(1.11-1.43) |
| 55 | South Asia | 1.12(1.10-1.13) |
| 55 | Southeast Asia | 1.13(1.11-1.15) |
| 55 | Southern Latin America | 1.39(1.28-1.52) |
| 55 | Southern Sub-Saharan Africa | 1.44(1.37-1.50) |
| 55 | Tropical Latin America | 1.09(1.05-1.13) |
| 55 | Western Europe | 1.11(1.07-1.15) |
| 55 | Andean Latin America | 1.36(1.26-1.46) |
| 55 | Western Sub-Saharan Africa | 1.21(1.18-1.25) |
| 55 | Central Asia | 1.16(1.10-1.23) |
| 55 | Central Europe | 1.18(1.12-1.23) |
| 55 | Central Latin America | 1.19(1.15-1.23) |
| 55 | Australasia | 1.25(1.05-1.47) |
| 55 | Central Sub-Saharan Africa | 1.34(1.28-1.40) |
| 55 | East Asia | 1.41(1.39-1.43) |
| 55 | Eastern Europe | 1.28(1.24-1.31) |
| 55 | Eastern Sub-Saharan Africa | 1.52(1.48-1.57) |
| 55 | Caribbean | 1.17(1.08-1.25) |
| 55 | High-income Asia Pacific | 1.39(1.29-1.50) |
| 60 | Low-middle SDI | 1.06(1.05-1.08) |
| 60 | Middle SDI | 1.15(1.14-1.16) |
| 60 | High-middle SDI | 1.18(1.16-1.20) |
| 60 | High SDI | 1.13(1.11-1.15) |
| 60 | Global | 1.17(1.16-1.17) |
| 60 | Low SDI | 1.16(1.14-1.18) |
| 60 | High-income North America | 1.23(1.19-1.26) |
| 60 | North Africa and Middle East | 0.79(0.78-0.81) |
| 60 | Oceania | 1.26(1.10-1.44) |
| 60 | South Asia | 1.04(1.03-1.05) |
| 60 | Southeast Asia | 1.14(1.12-1.16) |
| 60 | Southern Latin America | 1.29(1.20-1.39) |
| 60 | Southern Sub-Saharan Africa | 1.39(1.32-1.46) |
| 60 | Tropical Latin America | 1.07(1.03-1.10) |
| 60 | Western Europe | 0.99(0.95-1.03) |
| 60 | Andean Latin America | 1.33(1.24-1.42) |
| 60 | Western Sub-Saharan Africa | 1.15(1.11-1.18) |
| 60 | Central Asia | 1.09(1.03-1.14) |
| 60 | Central Europe | 1.08(1.04-1.12) |
| 60 | Central Latin America | 1.14(1.11-1.18) |
| 60 | Australasia | 1.18(0.99-1.40) |
| 60 | Central Sub-Saharan Africa | 1.28(1.21-1.34) |
| 60 | East Asia | 1.37(1.34-1.39) |
| 60 | Eastern Europe | 1.17(1.15-1.20) |
| 60 | Eastern Sub-Saharan Africa | 1.40(1.35-1.44) |
| 60 | Caribbean | 1.13(1.06-1.22) |
| 60 | High-income Asia Pacific | 1.20(1.11-1.30) |
| 65 | Low-middle SDI | 1.07(1.06-1.08) |
| 65 | Middle SDI | 1.18(1.17-1.19) |
| 65 | High-middle SDI | 1.13(1.12-1.14) |
| 65 | High SDI | 0.95(0.93-0.97) |
| 65 | Global | 1.13(1.12-1.14) |
| 65 | Low SDI | 1.12(1.09-1.14) |
| 65 | High-income North America | 1.02(0.99-1.04) |
| 65 | North Africa and Middle East | 0.80(0.78-0.81) |
| 65 | Oceania | 1.29(1.09-1.52) |
| 65 | South Asia | 1.04(1.03-1.05) |
| 65 | Southeast Asia | 1.18(1.16-1.20) |
| 65 | Southern Latin America | 1.08(1.00-1.15) |
| 65 | Southern Sub-Saharan Africa | 1.42(1.34-1.51) |
| 65 | Tropical Latin America | 1.07(1.03-1.10) |
| 65 | Western Europe | 0.80(0.77-0.82) |
| 65 | Andean Latin America | 1.31(1.22-1.41) |
| 65 | Western Sub-Saharan Africa | 1.09(1.05-1.12) |
| 65 | Central Asia | 1.01(0.96-1.07) |
| 65 | Central Europe | 0.97(0.94-1.00) |
| 65 | Central Latin America | 1.14(1.10-1.18) |
| 65 | Australasia | 0.98(0.84-1.12) |
| 65 | Central Sub-Saharan Africa | 1.24(1.16-1.32) |
| 65 | East Asia | 1.33(1.32-1.35) |
| 65 | Eastern Europe | 1.09(1.07-1.12) |
| 65 | Eastern Sub-Saharan Africa | 1.27(1.23-1.32) |
| 65 | Caribbean | 1.12(1.04-1.21) |
| 65 | High-income Asia Pacific | 0.95(0.89-1.01) |
| 70 | Low-middle SDI | 1.17(1.16-1.19) |
| 70 | Middle SDI | 1.26(1.24-1.27) |
| 70 | High-middle SDI | 1.17(1.15-1.18) |
| 70 | High SDI | 0.93(0.92-0.95) |
| 70 | Global | 1.18(1.18-1.19) |
| 70 | Low SDI | 1.23(1.20-1.26) |
| 70 | High-income North America | 0.99(0.96-1.01) |
| 70 | North Africa and Middle East | 0.88(0.86-0.90) |
| 70 | Oceania | 1.38(1.12-1.70) |
| 70 | South Asia | 1.14(1.12-1.15) |
| 70 | Southeast Asia | 1.28(1.26-1.31) |
| 70 | Southern Latin America | 1.11(1.03-1.19) |
| 70 | Southern Sub-Saharan Africa | 1.70(1.59-1.83) |
| 70 | Tropical Latin America | 1.08(1.04-1.12) |
| 70 | Western Europe | 0.82(0.80-0.84) |
| 70 | Andean Latin America | 1.37(1.26-1.49) |
| 70 | Western Sub-Saharan Africa | 1.21(1.16-1.26) |
| 70 | Central Asia | 1.09(1.02-1.16) |
| 70 | Central Europe | 1.04(1.00-1.07) |
| 70 | Central Latin America | 1.20(1.15-1.24) |
| 70 | Australasia | 0.94(0.85-1.05) |
| 70 | Central Sub-Saharan Africa | 1.45(1.33-1.58) |
| 70 | East Asia | 1.39(1.37-1.40) |
| 70 | Eastern Europe | 1.18(1.15-1.21) |
| 70 | Eastern Sub-Saharan Africa | 1.43(1.37-1.50) |
| 70 | Caribbean | 1.18(1.09-1.28) |
| 70 | High-income Asia Pacific | 0.90(0.87-0.94) |
| 75 | Low-middle SDI | 1.35(1.32-1.37) |
| 75 | Middle SDI | 1.42(1.41-1.44) |
| 75 | High-middle SDI | 1.32(1.30-1.34) |
| 75 | High SDI | 1.03(1.02-1.05) |
| 75 | Global | 1.34(1.33-1.35) |
| 75 | Low SDI | 1.47(1.42-1.51) |
| 75 | High-income North America | 1.09(1.06-1.13) |
| 75 | North Africa and Middle East | 0.99(0.95-1.02) |
| 75 | Oceania | 1.55(1.17-2.04) |
| 75 | South Asia | 1.27(1.25-1.30) |
| 75 | Southeast Asia | 1.48(1.44-1.52) |
| 75 | Southern Latin America | 1.33(1.22-1.44) |
| 75 | Southern Sub-Saharan Africa | 2.35(2.14-2.57) |
| 75 | Tropical Latin America | 1.18(1.13-1.23) |
| 75 | Western Europe | 0.94(0.92-0.97) |
| 75 | Andean Latin America | 1.54(1.39-1.70) |
| 75 | Western Sub-Saharan Africa | 1.49(1.42-1.57) |
| 75 | Central Asia | 1.26(1.14-1.38) |
| 75 | Central Europe | 1.20(1.15-1.25) |
| 75 | Central Latin America | 1.32(1.26-1.38) |
| 75 | Australasia | 1.03(0.93-1.14) |
| 75 | Central Sub-Saharan Africa | 1.93(1.73-2.15) |
| 75 | East Asia | 1.56(1.54-1.58) |
| 75 | Eastern Europe | 1.42(1.36-1.48) |
| 75 | Eastern Sub-Saharan Africa | 1.96(1.85-2.08) |
| 75 | Caribbean | 1.32(1.20-1.44) |
| 75 | High-income Asia Pacific | 0.97(0.93-1.00) |
| 80 | Low-middle SDI | 1.56(1.53-1.60) |
| 80 | Middle SDI | 1.63(1.60-1.65) |
| 80 | High-middle SDI | 1.45(1.42-1.48) |
| 80 | High SDI | 1.18(1.16-1.20) |
| 80 | Global | 1.53(1.52-1.55) |
| 80 | Low SDI | 1.76(1.68-1.84) |
| 80 | High-income North America | 1.26(1.21-1.30) |
| 80 | North Africa and Middle East | 1.15(1.10-1.20) |
| 80 | Oceania | 1.80(1.16-2.70) |
| 80 | South Asia | 1.43(1.40-1.47) |
| 80 | Southeast Asia | 1.74(1.68-1.80) |
| 80 | Southern Latin America | 1.57(1.43-1.74) |
| 80 | Southern Sub-Saharan Africa | 3.21(2.81-3.65) |
| 80 | Tropical Latin America | 1.31(1.24-1.38) |
| 80 | Western Europe | 1.12(1.10-1.15) |
| 80 | Andean Latin America | 1.81(1.59-2.05) |
| 80 | Western Sub-Saharan Africa | 1.81(1.68-1.93) |
| 80 | Central Asia | 1.48(1.33-1.64) |
| 80 | Central Europe | 1.40(1.33-1.48) |
| 80 | Central Latin America | 1.49(1.41-1.58) |
| 80 | Australasia | 1.14(1.01-1.28) |
| 80 | Central Sub-Saharan Africa | 2.54(2.15-2.99) |
| 80 | East Asia | 1.75(1.72-1.78) |
| 80 | Eastern Europe | 1.73(1.66-1.81) |
| 80 | Eastern Sub-Saharan Africa | 2.68(2.46-2.91) |
| 80 | Caribbean | 1.51(1.34-1.69) |
| 80 | High-income Asia Pacific | 1.09(1.05-1.13) |
| 85 | Low-middle SDI | 1.72(1.64-1.80) |
| 85 | Middle SDI | 1.88(1.82-1.93) |
| 85 | High-middle SDI | 1.65(1.60-1.70) |
| 85 | High SDI | 1.28(1.25-1.32) |
| 85 | Global | 1.73(1.71-1.76) |
| 85 | Low SDI | 2.01(1.85-2.19) |
| 85 | High-income North America | 1.31(1.24-1.37) |
| 85 | North Africa and Middle East | 1.28(1.18-1.37) |
| 85 | Oceania | 2.15(0.86-4.70) |
| 85 | South Asia | 1.57(1.51-1.64) |
| 85 | Southeast Asia | 1.86(1.75-1.98) |
| 85 | Southern Latin America | 1.75(1.51-2.02) |
| 85 | Southern Sub-Saharan Africa | 4.09(3.19-5.22) |
| 85 | Tropical Latin America | 1.39(1.27-1.52) |
| 85 | Western Europe | 1.24(1.19-1.28) |
| 85 | Andean Latin America | 2.09(1.71-2.53) |
| 85 | Western Sub-Saharan Africa | 2.02(1.76-2.29) |
| 85 | Central Asia | 1.78(1.46-2.15) |
| 85 | Central Europe | 1.63(1.50-1.77) |
| 85 | Central Latin America | 1.59(1.45-1.74) |
| 85 | Australasia | 1.24(1.04-1.47) |
| 85 | Central Sub-Saharan Africa | 3.12(2.25-4.28) |
| 85 | East Asia | 2.13(2.06-2.20) |
| 85 | Eastern Europe | 2.01(1.86-2.16) |
| 85 | Eastern Sub-Saharan Africa | 3.21(2.75-3.74) |
| 85 | Caribbean | 1.66(1.37-1.99) |
| 85 | High-income Asia Pacific | 1.28(1.21-1.35) |
| 90 | Low-middle SDI | 1.93(1.74-2.14) |
| 90 | Middle SDI | 2.46(2.29-2.63) |
| 90 | High-middle SDI | 1.98(1.85-2.12) |
| 90 | High SDI | 1.40(1.33-1.48) |
| 90 | Global | 2.08(2.01-2.15) |
| 90 | Low SDI | 2.40(1.90-2.98) |
| 90 | High-income North America | 1.32(1.20-1.45) |
| 90 | North Africa and Middle East | 1.28(1.08-1.52) |
| 90 | Oceania | 17.28(-32.89-32.37) |
| 90 | South Asia | 1.91(1.71-2.12) |
| 90 | Southeast Asia | 1.85(1.60-2.14) |
| 90 | Southern Latin America | 1.98(1.45-2.68) |
| 90 | Southern Sub-Saharan Africa | 5.42(2.70-10.57) |
| 90 | Tropical Latin America | 1.52(1.25-1.84) |
| 90 | Western Europe | 1.31(1.22-1.41) |
| 90 | Andean Latin America | 2.56(1.68-3.79) |
| 90 | Western Sub-Saharan Africa | 2.25(1.59-3.09) |
| 90 | Central Asia | 2.34(1.50-3.59) |
| 90 | Central Europe | 1.95(1.62-2.34) |
| 90 | Central Latin America | 1.63(1.34-1.96) |
| 90 | Australasia | 1.38(0.99-1.90) |
| 90 | Central Sub-Saharan Africa | 4.52(1.70-11.18) |
| 90 | East Asia | 3.47(3.20-3.76) |
| 90 | Eastern Europe | 2.03(1.74-2.37) |
| 90 | Eastern Sub-Saharan Africa | 3.83(2.54-5.63) |
| 90 | Caribbean | 1.86(1.25-2.69) |
| 90 | High-income Asia Pacific | 1.54(1.39-1.71) |
| 95 | Low-middle SDI | 3.04(2.13-4.27) |
| 95 | Middle SDI | 4.04(3.21-5.05) |
| 95 | High-middle SDI | 2.60(2.06-3.27) |
| 95 | High SDI | 1.68(1.46-1.92) |
| 95 | Global | 2.89(2.61-3.19) |
| 95 | Low SDI | 4.26(1.39-11.37) |
| 95 | High-income North America | 1.67(1.25-2.22) |
| 95 | North Africa and Middle East | 1.44(0.78-2.46) |
| 95 | Oceania | 1.45(-24.36-24.89) |
| 95 | South Asia | 3.58(2.31-5.44) |
| 95 | Southeast Asia | 2.11(1.34-3.23) |
| 95 | Southern Latin America | 1.84(0.74-4.12) |
| 95 | Southern Sub-Saharan Africa | -2.43(-53.24-50.97) |
| 95 | Tropical Latin America | 1.78(1.12-2.76) |
| 95 | Western Europe | 1.69(1.35-2.12) |
| 95 | Andean Latin America | 6.09(0.95-29.21) |
| 95 | Western Sub-Saharan Africa | 5.33(0.94-24.66) |
| 95 | Central Asia | 2.15(-42.96-43.82) |
| 95 | Central Europe | 2.55(1.32-4.77) |
| 95 | Central Latin America | 2.22(1.37-3.46) |
| 95 | Australasia | 2.06(0.68-5.87) |
| 95 | Central Sub-Saharan Africa | 2.03(-42.69-42.23) |
| 95 | East Asia | 8.16(5.94-11.16) |
| 95 | Eastern Europe | 1.55(0.89-2.65) |
| 95 | Eastern Sub-Saharan Africa | -3.14(-86.48-86.61) |
| 95 | Caribbean | 3.03(1.09-7.73) |
| 95 | High-income Asia Pacific | 1.59(1.30-1.95) |

Table S6. Regional AAPC in migraine lifetime risk with 95% confidence intervals, 1990-2021, both sexes.

| **Region** | **Lifetime Risk 1990** | **Lifetime Risk 2021** | **AAPC(95%CI)** | **P value** |
| --- | --- | --- | --- | --- |
| **Global** | 47.50(47.47-47.52) | 51.53(51.51-51.55) | 0.26 (0.26, 0.27) | <0.001 |
| **High SDI** | 54.18(54.12-54.23) | 55.74(55.69-55.79) | 0.09 (0.09, 0.1) | <0.001 |
| **High-middle SDI** | 48.77(48.71-48.83) | 52.06(52.01-52.11) | 0.21 (0.2, 0.22) | <0.001 |
| **Middle SDI** | 48.84(48.80-48.89) | 53.40(53.36-53.43) | 0.28 (0.28, 0.29) | <0.001 |
| **Low-middle SDI** | 47.39(47.34-47.44) | 52.29(52.26-52.33) | 0.32 (0.31, 0.32) | <0.001 |
| **Low SDI** | 38.42(38.34-38.50) | 45.13(45.07-45.19) | 0.51 (0.47, 0.56) | <0.001 |
| **Andean Latin America** | 39.22(38.87-39.57) | 43.29(43.05-43.53) | 0.32 (0.31, 0.34) | <0.001 |
| **Bolivia (Plurinational State of)** | 38.83(38.04-39.61) | 42.79(42.24-43.34) | 0.3 (0.29, 0.32) | <0.001 |
| **Ecuador** | 45.16(44.48-45.84) | 47.18(46.73-47.64) | 0.15 (0.14, 0.17) | <0.001 |
| **Peru** | 36.63(36.14-37.12) | 41.37(41.04-41.71) | 0.41 (0.39, 0.44) | <0.001 |
| **Australasia** | 51.55(51.17-51.93) | 52.70(52.42-52.99) | 0.07 (0.07, 0.07) | <0.001 |
| **Australia** | 51.37(50.96-51.78) | 52.52(52.20-52.83) | 0.07 (0.07, 0.07) | <0.001 |
| **New Zealand** | 52.42(51.49-53.35) | 53.62(52.93-54.30) | 0.07 (0.07, 0.08) | <0.001 |
| **Caribbean** | 49.02(48.73-49.32) | 50.57(50.32-50.83) | 0.09 (-0.09, 0.28) | 0.268 |
| **Antigua and Barbuda** | 53.06(45.87-60.25) | 53.38(48.21-58.55) | 0.02 (0.01, 0.03) | <0.001 |
| **Bahamas** | 51.74(48.31-55.16) | 52.23(49.52-54.94) | 0.03 (-0.01, 0.07) | 0.102 |
| **Barbados** | 52.60(49.06-56.14) | 53.30(50.20-56.39) | 0.05 (0.03, 0.06) | <0.001 |
| **Belize** | 50.80(46.09-55.51) | 52.50(49.85-55.15) | 0.11 (0.1, 0.11) | <0.001 |
| **Cuba** | 52.67(52.13-53.20) | 53.08(52.59-53.58) | 0.03 (0.03, 0.04) | <0.001 |
| **Dominica** | 52.07(45.23-58.91) | 51.43(44.88-57.98) | -0.02 (-0.05, 0) | 0.079 |
| **Dominican Republic** | 49.79(49.09-50.50) | 51.87(51.35-52.39) | 0.13 (0.13, 0.14) | <0.001 |
| **Grenada** | 51.58(44.96-58.19) | 51.89(46.72-57.06) | 0.02 (0.01, 0.04) | <0.001 |
| **Guyana** | 48.68(46.70-50.66) | 50.54(48.60-52.48) | 0.13 (0.11, 0.14) | <0.001 |
| **Haiti** | 42.45(41.82-43.07) | 47.55(47.08-48.01) | 0.32 (-0.4, 1.11) | 0.314 |
| **Jamaica** | 52.43(51.25-53.61) | 52.76(51.78-53.73) | 0.03 (0.01, 0.04) | 0.002 |
| **Saint Lucia** | 51.88(46.81-56.94) | 52.32(48.28-56.37) | 0.03 (0.03, 0.04) | <0.001 |
| **Saint Vincent and the Grenadines** | 51.18(45.34-57.03) | 52.21(46.99-57.42) | 0.08 (0.07, 0.08) | <0.001 |
| **Suriname** | 49.84(46.91-52.77) | 51.53(49.22-53.83) | 0.11 (0.1, 0.12) | <0.001 |
| **Trinidad and Tobago** | 51.41(49.80-53.02) | 51.95(50.42-53.49) | 0.04 (0.03, 0.04) | <0.001 |
| **Bermuda** | 52.99(46.47-59.51) | 54.08(47.62-60.53) | 0.07 (0.06, 0.08) | <0.001 |
| **Puerto Rico** | 52.68(51.72-53.63) | 53.96(52.98-54.94) | 0.08 (0.06, 0.1) | <0.001 |
| **Saint Kitts and Nevis** | 50.95(41.76-60.14) | 52.34(45.62-59.05) | 0.09 (0.08, 0.1) | <0.001 |
| **United States Virgin Islands** | 52.35(46.79-57.90) | 53.04(45.97-60.10) | 0.04 (0.03, 0.04) | <0.001 |
| **Central Asia** | 48.72(48.49-48.95) | 50.79(50.61-50.96) | 0.13 (0.12, 0.14) | <0.001 |
| **Armenia** | 50.10(49.12-51.08) | 52.16(51.20-53.11) | 0.09 (-0.03, 0.23) | 0.098 |
| **Azerbaijan** | 47.82(47.14-48.51) | 50.41(49.89-50.93) | 0.16 (0.13, 0.19) | <0.001 |
| **Georgia** | 50.00(49.22-50.77) | 51.36(50.42-52.30) | 0.1 (0.08, 0.14) | <0.001 |
| **Kazakhstan** | 49.47(49.01-49.94) | 51.10(50.70-51.50) | 0.1 (0.09, 0.11) | <0.001 |
| **Kyrgyzstan** | 48.32(47.41-49.24) | 51.26(50.58-51.94) | 0.18 (0.17, 0.19) | <0.001 |
| **Mongolia** | 46.40(45.12-47.68) | 50.80(49.85-51.76) | 0.3 (0.29, 0.31) | <0.001 |
| **Tajikistan** | 47.26(46.40-48.12) | 49.84(49.27-50.41) | 0.18 (0.12, 0.27) | <0.001 |
| **Turkmenistan** | 47.75(46.75-48.76) | 49.42(48.63-50.21) | 0.11 (0.1, 0.12) | <0.001 |
| **Uzbekistan** | 49.09(48.65-49.53) | 51.04(50.74-51.35) | 0.13 (0.12, 0.13) | <0.001 |
| **Central Europe** | 51.03(50.87-51.19) | 52.49(52.34-52.64) | 0.09 (0.09, 0.1) | <0.001 |
| **Albania** | 50.00(48.96-51.04) | 51.90(50.88-52.91) | 0.13 (0.1, 0.15) | <0.001 |
| **Bosnia and Herzegovina** | 51.24(50.43-52.04) | 52.23(51.36-53.11) | 0.06 (0, 0.17) | 0.051 |
| **Bulgaria** | 50.56(49.93-51.19) | 50.98(50.27-51.69) | 0.03 (0.02, 0.03) | <0.001 |
| **Croatia** | 51.32(50.54-52.10) | 52.53(51.76-53.29) | 0.11 (0.08, 0.13) | <0.001 |
| **Czechia** | 51.24(50.71-51.77) | 52.56(52.06-53.06) | 0.08 (0.08, 0.09) | <0.001 |
| **Hungary** | 50.48(49.93-51.03) | 52.08(51.56-52.61) | 0.1 (0.1, 0.11) | <0.001 |
| **North Macedonia** | 49.98(48.80-51.16) | 51.43(50.35-52.50) | 0.09 (0.08, 0.11) | <0.001 |
| **Montenegro** | 51.56(49.40-53.71) | 51.86(49.78-53.93) | 0.02 (0.01, 0.02) | <0.001 |
| **Poland** | 52.21(51.93-52.50) | 53.67(53.40-53.93) | 0.09 (0.09, 0.1) | <0.001 |
| **Romania** | 50.03(49.65-50.41) | 51.58(51.19-51.97) | 0.09 (0.08, 0.1) | <0.001 |
| **Serbia** | 50.32(49.76-50.88) | 51.88(51.37-52.39) | 0.12 (0.07, 0.15) | <0.001 |
| **Slovakia** | 51.02(50.28-51.77) | 52.01(51.31-52.71) | 0.07 (0.06, 0.08) | <0.001 |
| **Slovenia** | 51.62(50.38-52.86) | 52.93(51.85-54.02) | 0.09 (0.09, 0.09) | <0.001 |
| **Central Latin America** | 49.92(49.77-50.08) | 51.92(51.81-52.03) | 0.12 (0.11, 0.13) | <0.001 |
| **Colombia** | 50.61(50.27-50.95) | 52.85(52.59-53.10) | 0.16 (0.15, 0.17) | <0.001 |
| **Costa Rica** | 52.29(51.20-53.39) | 52.93(52.15-53.72) | 0.04 (0.03, 0.05) | <0.001 |
| **El Salvador** | 49.04(48.18-49.91) | 52.28(51.55-53.00) | 0.2 (0.19, 0.22) | <0.001 |
| **Guatemala** | 46.70(46.03-47.37) | 51.04(50.59-51.48) | 0.3 (0.29, 0.32) | <0.001 |
| **Honduras** | 48.83(47.87-49.78) | 51.52(50.98-52.06) | 0.19 (0.14, 0.25) | <0.001 |
| **Mexico** | 50.01(49.80-50.22) | 51.84(51.69-51.99) | 0.11 (0.09, 0.12) | <0.001 |
| **Nicaragua** | 49.85(48.78-50.92) | 52.04(51.38-52.69) | 0.14 (0.1, 0.2) | <0.001 |
| **Panama** | 51.43(50.18-52.68) | 52.34(51.47-53.20) | 0.05 (0.04, 0.06) | <0.001 |
| **Venezuela (Bolivarian Republic of)** | 50.25(49.80-50.69) | 51.07(50.70-51.44) | 0.05 (-0.04, 0.15) | 0.127 |
| **Central Sub-Saharan Africa** | 37.59(37.35-37.83) | 44.16(43.99-44.33) | 0.51 (0.5, 0.53) | <0.001 |
| **Angola** | 35.09(34.57-35.61) | 44.49(44.15-44.83) | 0.77 (0.73, 0.82) | <0.001 |
| **Central African Republic** | 35.25(34.28-36.22) | 39.51(38.78-40.25) | 0.38 (0.34, 0.42) | <0.001 |
| **Congo** | 40.57(39.41-41.73) | 45.06(44.26-45.85) | 0.32 (0.21, 0.44) | <0.001 |
| **Democratic Republic of the Congo** | 38.21(37.92-38.51) | 44.29(44.08-44.49) | 0.48 (0.45, 0.51) | <0.001 |
| **Equatorial Guinea** | 37.75(35.00-40.49) | 44.09(42.55-45.63) | 0.49 (0.47, 0.52) | <0.001 |
| **Gabon** | 42.93(40.94-44.92) | 46.12(44.73-47.52) | 0.22 (0.21, 0.24) | <0.001 |
| **East Asia** | 43.70(43.65-43.76) | 49.39(49.34-49.44) | 0.39 (0.38, 0.4) | <0.001 |
| **China** | 43.53(43.47-43.59) | 49.37(49.32-49.42) | 0.4 (0.39, 0.41) | <0.001 |
| **Democratic People's Republic of Korea** | 47.46(47.03-47.89) | 48.42(48.05-48.78) | 0.01 (-0.12, 0.12) | 0.963 |
| **Taiwan (Province of China)** | 49.91(49.48-50.34) | 52.09(51.74-52.43) | 0.14 (0.13, 0.15) | <0.001 |
| **Eastern Europe** | 49.73(49.60-49.86) | 50.47(50.35-50.60) | 0.04 (0.02, 0.05) | <0.001 |
| **Belarus** | 48.21(47.60-48.81) | 48.69(48.09-49.29) | 0.04 (0.02, 0.06) | <0.001 |
| **Estonia** | 47.90(46.29-49.51) | 49.63(48.04-51.23) | 0.13 (0.1, 0.15) | <0.001 |
| **Latvia** | 47.78(46.53-49.04) | 49.04(47.69-50.40) | 0.08 (0.07, 0.1) | <0.001 |
| **Lithuania** | 47.18(46.14-48.22) | 48.16(47.01-49.31) | 0.06 (0.05, 0.07) | <0.001 |
| **Republic of Moldova** | 47.27(46.36-48.19) | 48.60(47.66-49.55) | 0.09 (0.07, 0.1) | <0.001 |
| **Russian Federation** | 50.13(49.97-50.29) | 50.88(50.73-51.04) | 0.04 (0.02, 0.06) | 0.001 |
| **Ukraine** | 49.40(49.13-49.67) | 49.73(49.44-50.03) | 0.02 (0.01, 0.03) | 0.002 |
| **Eastern Sub-Saharan Africa** | 29.29(29.16-29.43) | 35.63(35.53-35.73) | 0.62 (0.51, 0.75) | <0.001 |
| **Burundi** | 29.28(28.53-30.04) | 35.69(35.09-36.30) | 0.65 (0.58, 0.72) | <0.001 |
| **Comoros** | 32.91(29.75-36.06) | 37.21(34.71-39.70) | 0.39 (0.37, 0.41) | <0.001 |
| **Djibouti** | 33.87(30.47-37.28) | 36.44(34.59-38.29) | 0.23 (0.21, 0.24) | <0.001 |
| **Eritrea** | 28.13(27.30-28.96) | 35.98(35.17-36.79) | 0.83 (0.76, 0.9) | <0.001 |
| **Ethiopia** | 25.89(25.66-26.13) | 34.76(34.54-34.98) | 0.94 (0.91, 0.97) | <0.001 |
| **Kenya** | 35.59(35.12-36.06) | 38.08(37.79-38.38) | 0.21 (0.2, 0.22) | <0.001 |
| **Madagascar** | 31.90(31.30-32.50) | 36.10(35.70-36.51) | 0.4 (0.39, 0.4) | <0.001 |
| **Malawi** | 28.06(27.51-28.61) | 35.38(34.92-35.84) | 0.75 (0.73, 0.77) | <0.001 |
| **Mozambique** | 28.84(28.34-29.35) | 34.37(34.02-34.72) | 0.57 (0.56, 0.59) | <0.001 |
| **Rwanda** | 29.85(29.17-30.53) | 37.28(36.69-37.87) | -0.13 (-1.49, 1.2) | 0.616 |
| **Somalia** | 28.34(27.71-28.97) | 32.59(32.17-33.01) | 0.52 (0.29, 0.75) | <0.001 |
| **United Republic of Tanzania** | 30.91(30.52-31.30) | 36.95(36.66-37.23) | 0.59 (0.57, 0.61) | <0.001 |
| **Uganda** | 28.73(28.35-29.11) | 35.65(35.32-35.98) | 0.72 (0.7, 0.73) | <0.001 |
| **Zambia** | 31.38(30.74-32.01) | 36.96(36.52-37.40) | 0.54 (0.52, 0.56) | <0.001 |
| **South Sudan** | 29.02(28.23-29.80) | 32.08(31.43-32.73) | 0.32 (0.28, 0.36) | <0.001 |
| **High-income Asia Pacific** | 44.27(44.13-44.41) | 45.76(45.63-45.89) | 0.1 (0.1, 0.11) | <0.001 |
| **Brunei Darussalam** | 42.15(38.03-46.26) | 43.12(40.59-45.64) | 0.07 (0.07, 0.08) | <0.001 |
| **Japan** | 43.56(43.40-43.72) | 45.30(45.14-45.46) | 0.13 (0.12, 0.13) | <0.001 |
| **Republic of Korea** | 46.08(45.81-46.36) | 47.13(46.90-47.36) | 0.07 (0.06, 0.08) | <0.001 |
| **Singapore** | 38.15(37.02-39.28) | 42.66(41.95-43.38) | 0.37 (0.35, 0.38) | <0.001 |
| **High-income North America** | 57.89(57.80-57.97) | 57.81(57.72-57.89) | 0 (-0.01, 0) | 0.396 |
| **Canada** | 55.95(55.66-56.23) | 56.50(56.26-56.75) | 0.03 (0.03, 0.03) | <0.001 |
| **United States of America** | 58.09(58.00-58.19) | 57.94(57.86-58.03) | -0.01 (-0.01, 0) | 0.068 |
| **Greenland** | 51.51(44.71-58.30) | 55.01(48.25-61.78) | 0.21 (0.21, 0.22) | <0.001 |
| **North Africa and Middle East** | 49.92(49.82-50.02) | 54.11(54.04-54.18) | 0.26 (0.25, 0.27) | <0.001 |
| **Algeria** | 51.80(51.41-52.19) | 54.58(54.34-54.83) | 0.16 (0.16, 0.17) | <0.001 |
| **Bahrain** | 51.96(49.51-54.42) | 52.90(51.64-54.15) | 0.06 (0.05, 0.07) | <0.001 |
| **Egypt** | 49.43(49.20-49.66) | 54.93(54.77-55.08) | 0.34 (0.33, 0.34) | <0.001 |
| **Iran (Islamic Republic of)** | 52.45(52.20-52.71) | 57.51(57.34-57.69) | 0.27 (0.25, 0.3) | <0.001 |
| **Iraq** | 50.68(50.23-51.12) | 53.60(53.34-53.85) | 0.19 (0.16, 0.22) | <0.001 |
| **Jordan** | 52.88(51.86-53.90) | 54.53(54.07-54.99) | 0.1 (0.09, 0.1) | <0.001 |
| **Kuwait** | 53.06(51.53-54.59) | 56.05(55.34-56.77) | 0.2 (0.17, 0.24) | <0.001 |
| **Lebanon** | 52.79(51.71-53.87) | 55.18(54.51-55.85) | 0.13 (0.11, 0.15) | <0.001 |
| **Libya** | 52.43(51.45-53.40) | 53.54(52.93-54.15) | 0.04 (0, 0.08) | 0.041 |
| **Morocco** | 50.38(50.02-50.73) | 54.55(54.30-54.80) | 0.25 (0.25, 0.26) | <0.001 |
| **Palestine** | 52.56(51.15-53.96) | 54.86(54.10-55.62) | 0.12 (0.07, 0.16) | <0.001 |
| **Oman** | 50.43(49.07-51.80) | 52.76(51.99-53.52) | 0.15 (0.14, 0.16) | <0.001 |
| **Qatar** | 50.66(47.82-53.49) | 52.56(51.47-53.66) | 0.12 (0.12, 0.13) | <0.001 |
| **Saudi Arabia** | 50.10(49.63-50.57) | 53.12(52.87-53.38) | 0.18 (0.17, 0.19) | <0.001 |
| **Syrian Arab Republic** | 52.07(51.52-52.62) | 55.65(55.20-56.11) | 0.22 (0.15, 0.27) | <0.001 |
| **Tunisia** | 52.66(52.04-53.29) | 55.06(54.61-55.52) | 0.15 (0.14, 0.16) | <0.001 |
| **Turkey** | 50.22(49.99-50.45) | 54.67(54.50-54.84) | 0.27 (0.25, 0.28) | <0.001 |
| **United Arab Emirates** | 50.78(49.41-52.15) | 53.01(52.40-53.62) | 0.14 (0.13, 0.16) | <0.001 |
| **Yemen** | 45.82(45.33-46.31) | 51.48(51.16-51.79) | 0.38 (0.37, 0.39) | <0.001 |
| **Afghanistan** | 43.31(42.75-43.88) | 48.70(48.38-49.02) | 0.41 (0.37, 0.47) | <0.001 |
| **Sudan** | 44.66(44.28-45.04) | 52.58(52.32-52.85) | 0.51 (0.5, 0.52) | <0.001 |
| **Oceania** | 48.55(47.83-49.27) | 49.97(49.49-50.45) | 0.1 (0.09, 0.12) | <0.001 |
| **Fiji** | 51.62(49.57-53.67) | 51.93(50.13-53.74) | 0.02 (0.01, 0.03) | <0.001 |
| **Kiribati** | 47.14(40.62-53.67) | 50.69(45.56-55.83) | 0.23 (0.18, 0.28) | <0.001 |
| **Marshall Islands** | 50.14(40.93-59.35) | 51.30(43.98-58.61) | 0.08 (0.07, 0.08) | <0.001 |
| **Micronesia (Federated States of)** | 49.88(43.97-55.79) | 52.00(46.49-57.51) | 0.13 (0.12, 0.15) | <0.001 |
| **Papua New Guinea** | 47.51(46.60-48.43) | 49.50(48.94-50.05) | 0.14 (0.13, 0.17) | <0.001 |
| **Samoa** | 51.61(46.72-56.50) | 52.96(48.94-56.98) | 0.07 (0.01, 0.13) | 0.011 |
| **Solomon Islands** | 49.03(45.77-52.28) | 51.54(49.36-53.73) | 0.16 (0.16, 0.17) | <0.001 |
| **Tonga** | 52.72(46.55-58.90) | 53.89(48.32-59.46) | 0.07 (0.04, 0.09) | <0.001 |
| **Vanuatu** | 50.10(45.27-54.92) | 51.62(48.40-54.83) | 0.09 (0.08, 0.11) | <0.001 |
| **American Samoa** | 52.66(44.12-61.19) | 53.17(45.15-61.19) | 0.03 (0.01, 0.04) | 0.002 |
| **Cook Islands** | 51.87(37.92-65.82) | 54.91(42.07-67.75) | 0.16 (0.09, 0.23) | <0.001 |
| **Guam** | 53.31(48.32-58.30) | 53.95(49.59-58.30) | 0.02 (-0.02, 0.07) | 0.37 |
| **Nauru** | 49.39(31.58-67.21) | 50.44(33.60-67.29) | 0.07 (0.06, 0.07) | <0.001 |
| **Niue** | 52.26(9.81-94.71) | 50.44(-0.91-101.79) | -0.14 (-0.2, -0.11) | <0.001 |
| **Northern Mariana Islands** | 53.00(44.99-61.00) | 53.48(45.67-61.28) | 0.04 (0.04, 0.05) | <0.001 |
| **Palau** | 50.92(36.24-65.60) | 51.52(37.07-65.96) | 0.04 (0.03, 0.04) | <0.001 |
| **Tokelau** | 52.06(-3.45-107.56) | 50.10(-7.02-107.23) | -0.18 (-0.26, -0.14) | <0.001 |
| **Tuvalu** | 47.96(29.14-66.79) | 51.93(35.48-68.37) | 0.23 (0.17, 0.28) | <0.001 |
| **South Asia** | 47.85(47.80-47.91) | 53.30(53.26-53.34) | 0.35 (0.35, 0.36) | <0.001 |
| **Bangladesh** | 45.71(45.54-45.88) | 53.92(53.79-54.05) | 0.56 (0.52, 0.62) | <0.001 |
| **Bhutan** | 45.95(43.69-48.21) | 53.77(51.86-55.69) | 0.51 (0.5, 0.52) | <0.001 |
| **India** | 48.23(48.17-48.29) | 53.65(53.61-53.69) | 0.35 (0.34, 0.35) | <0.001 |
| **Nepal** | 46.43(46.05-46.81) | 54.63(54.34-54.92) | 0.52 (0.5, 0.54) | <0.001 |
| **Pakistan** | 47.89(47.71-48.06) | 51.30(51.19-51.42) | 0.23 (0.21, 0.25) | <0.001 |
| **Southeast Asia** | 53.16(53.08-53.24) | 56.74(56.68-56.80) | 0.2 (0.18, 0.22) | <0.001 |
| **Cambodia** | 48.16(47.63-48.69) | 55.02(54.62-55.41) | 0.43 (0.42, 0.43) | <0.001 |
| **Indonesia** | 52.83(52.70-52.95) | 57.00(56.91-57.09) | 0.25 (0.2, 0.32) | <0.001 |
| **Lao People's Democratic Republic** | 44.30(43.52-45.08) | 54.31(53.70-54.91) | 0.66 (0.65, 0.66) | <0.001 |
| **Malaysia** | 53.81(53.37-54.25) | 54.99(54.71-55.28) | 0.09 (0.07, 0.1) | <0.001 |
| **Maldives** | 52.56(48.58-56.55) | 56.58(54.34-58.82) | 0.24 (0.23, 0.26) | <0.001 |
| **Myanmar** | 46.50(46.24-46.75) | 54.53(54.31-54.75) | 0.57 (0.5, 0.64) | <0.001 |
| **Philippines** | 54.77(54.53-55.00) | 56.69(56.54-56.84) | 0.13 (0.12, 0.15) | <0.001 |
| **Sri Lanka** | 54.99(54.54-55.44) | 57.86(57.52-58.19) | 0.18 (0.14, 0.23) | <0.001 |
| **Thailand** | 57.83(57.60-58.05) | 58.87(58.66-59.08) | 0.06 (0.05, 0.07) | <0.001 |
| **Timor-Leste** | 46.91(44.98-48.84) | 54.67(53.18-56.16) | 0.53 (0.42, 0.64) | <0.001 |
| **Viet Nam** | 54.71(54.49-54.93) | 57.01(56.86-57.16) | 0.13 (0.13, 0.14) | <0.001 |
| **Mauritius** | 55.76(54.26-57.27) | 56.77(55.37-58.16) | 0.05 (0.04, 0.06) | <0.001 |
| **Seychelles** | 55.79(49.38-62.19) | 56.44(51.85-61.04) | 0.04 (0.03, 0.04) | <0.001 |
| **Southern Latin America** | 45.38(45.11-45.66) | 47.23(47.01-47.45) | 0.13 (0.12, 0.13) | <0.001 |
| **Argentina** | 45.03(44.70-45.36) | 47.06(46.79-47.33) | 0.15 (0.14, 0.15) | <0.001 |
| **Chile** | 46.06(45.54-46.59) | 47.70(47.29-48.11) | 0.11 (0.11, 0.12) | <0.001 |
| **Uruguay** | 46.18(45.12-47.24) | 47.16(46.12-48.20) | 0.09 (0.07, 0.1) | <0.001 |
| **Southern Sub-Saharan Africa** | 44.62(44.36-44.88) | 44.90(44.70-45.09) | 0.01 (0, 0.03) | 0.09 |
| **Botswana** | 44.10(42.37-45.83) | 44.45(43.34-45.56) | 0.02 (-0.01, 0.04) | 0.191 |
| **Lesotho** | 43.67(42.06-45.28) | 39.59(38.44-40.74) | -0.31 (-0.36, -0.27) | <0.001 |
| **Namibia** | 43.99(42.30-45.68) | 44.92(43.77-46.06) | 0.06 (0.04, 0.09) | <0.001 |
| **South Africa** | 44.93(44.62-45.24) | 45.79(45.55-46.02) | 0.05 (0.03, 0.07) | <0.001 |
| **Eswatini** | 43.60(41.35-45.85) | 41.91(40.37-43.44) | -0.13 (-0.17, -0.08) | <0.001 |
| **Zimbabwe** | 43.55(42.93-44.16) | 42.82(42.38-43.26) | -0.05 (-0.07, -0.02) | <0.001 |
| **Tropical Latin America** | 57.35(57.23-57.47) | 60.54(60.45-60.64) | 0.17 (0.16, 0.18) | <0.001 |
| **Brazil** | 57.32(57.20-57.44) | 60.61(60.51-60.71) | 0.17 (0.16, 0.19) | <0.001 |
| **Paraguay** | 58.04(57.21-58.87) | 58.62(58.07-59.17) | 0.03 (0.03, 0.04) | <0.001 |
| **Western Europe** | 59.46(59.39-59.53) | 60.58(60.53-60.64) | 0.06 (0.06, 0.06) | <0.001 |
| **Andorra** | 58.66(52.93-64.40) | 59.81(55.73-63.89) | 0.06 (0.06, 0.07) | <0.001 |
| **Austria** | 57.44(56.92-57.95) | 58.45(58.03-58.87) | 0.06 (0.05, 0.06) | <0.001 |
| **Belgium** | 61.01(60.59-61.43) | 62.78(62.43-63.12) | 0.09 (0.09, 0.09) | <0.001 |
| **Cyprus** | 58.34(56.81-59.87) | 59.93(58.92-60.95) | 0.09 (0.08, 0.09) | <0.001 |
| **Denmark** | 55.55(54.91-56.19) | 57.19(56.66-57.72) | 0.09 (0.09, 0.1) | <0.001 |
| **Finland** | 58.68(58.04-59.32) | 59.72(59.15-60.30) | 0.06 (0.06, 0.06) | <0.001 |
| **France** | 58.81(58.62-59.00) | 59.64(59.48-59.80) | 0.04 (0.04, 0.05) | <0.001 |
| **Germany** | 59.65(59.50-59.80) | 60.91(60.78-61.04) | 0.07 (0.06, 0.07) | <0.001 |
| **Greece** | 59.75(59.34-60.17) | 60.35(59.96-60.75) | 0.04 (0.03, 0.04) | <0.001 |
| **Iceland** | 59.02(56.17-61.87) | 59.82(57.60-62.03) | 0.04 (0.03, 0.06) | <0.001 |
| **Ireland** | 58.76(58.03-59.49) | 60.03(59.48-60.58) | 0.07 (0.07, 0.07) | <0.001 |
| **Israel** | 58.96(58.32-59.60) | 59.98(59.55-60.41) | 0.06 (0.05, 0.07) | <0.001 |
| **Italy** | 61.86(61.69-62.03) | 63.14(63.00-63.28) | 0.07 (0.05, 0.08) | <0.001 |
| **Luxembourg** | 57.76(55.41-60.11) | 59.02(57.56-60.48) | 0.07 (0.07, 0.07) | <0.001 |
| **Malta** | 58.92(56.83-61.01) | 59.58(57.66-61.51) | 0.04 (0.04, 0.04) | <0.001 |
| **Netherlands** | 57.37(57.01-57.72) | 59.36(59.06-59.65) | 0.11 (0.1, 0.11) | <0.001 |
| **Norway** | 58.48(57.78-59.18) | 61.47(60.96-61.98) | 0.16 (0.15, 0.17) | <0.001 |
| **Portugal** | 58.26(57.78-58.74) | 59.96(59.58-60.35) | 0.1 (0.09, 0.1) | <0.001 |
| **Spain** | 59.36(59.13-59.59) | 60.52(60.35-60.69) | 0.06 (0.06, 0.06) | <0.001 |
| **Sweden** | 60.29(59.83-60.75) | 61.19(60.81-61.57) | 0.05 (0.05, 0.05) | <0.001 |
| **Switzerland** | 56.63(56.05-57.20) | 57.93(57.51-58.34) | 0.07 (0.07, 0.08) | <0.001 |
| **United Kingdom** | 59.15(58.97-59.33) | 60.24(60.08-60.40) | 0.06 (0.06, 0.06) | <0.001 |
| **Monaco** | 59.08(50.99-67.17) | 59.52(52.04-67.00) | 0.02 (0.02, 0.03) | <0.001 |
| **San Marino** | 59.33(50.67-67.98) | 60.39(53.74-67.04) | 0.06 (0.05, 0.06) | <0.001 |
| **Western Sub-Saharan Africa** | 41.59(41.47-41.71) | 48.40(48.32-48.48) | 0.49 (0.48, 0.5) | <0.001 |
| **Benin** | 42.49(41.68-43.29) | 48.26(47.75-48.76) | 0.42 (0.41, 0.42) | <0.001 |
| **Burkina Faso** | 39.65(39.12-40.18) | 46.83(46.45-47.21) | 0.53 (0.5, 0.55) | <0.001 |
| **Cameroon** | 44.43(43.87-44.99) | 48.14(47.82-48.45) | 0.26 (0.25, 0.26) | <0.001 |
| **Cabo Verde** | 50.52(46.89-54.16) | 53.05(50.80-55.31) | 0.15 (0.14, 0.17) | <0.001 |
| **Chad** | 40.38(39.68-41.07) | 45.45(45.00-45.90) | 0.37 (0.36, 0.39) | <0.001 |
| **Côte d'Ivoire** | 42.83(42.34-43.31) | 48.20(47.86-48.54) | 0.38 (0.37, 0.39) | <0.001 |
| **Gambia** | 44.73(42.90-46.56) | 49.69(48.52-50.85) | 0.34 (0.33, 0.34) | <0.001 |
| **Ghana** | 45.25(44.78-45.71) | 50.18(49.88-50.49) | 0.33 (0.32, 0.34) | <0.001 |
| **Guinea** | 39.21(38.55-39.87) | 47.34(46.84-47.83) | 0.6 (0.59, 0.62) | <0.001 |
| **Guinea-Bissau** | 39.42(37.84-41.01) | 47.68(46.47-48.88) | 0.6 (0.56, 0.63) | <0.001 |
| **Liberia** | 36.51(35.53-37.49) | 48.43(47.67-49.20) | 1 (0.95, 1.08) | <0.001 |
| **Mali** | 38.01(37.47-38.55) | 46.33(45.96-46.71) | 0.62 (0.6, 0.65) | <0.001 |
| **Mauritania** | 46.35(45.06-47.65) | 51.73(50.82-52.64) | 0.35 (0.34, 0.36) | <0.001 |
| **Niger** | 35.76(35.23-36.30) | 47.41(47.01-47.81) | 0.94 (0.92, 0.98) | <0.001 |
| **Nigeria** | 41.69(41.52-41.86) | 48.96(48.84-49.08) | 0.52 (0.51, 0.53) | <0.001 |
| **Sao Tome and Principe** | 47.03(41.32-52.73) | 52.53(48.74-56.32) | 0.36 (0.35, 0.37) | <0.001 |
| **Senegal** | 44.53(43.86-45.19) | 50.53(50.07-50.99) | 0.4 (0.39, 0.41) | <0.001 |
| **Sierra Leone** | 38.16(37.40-38.92) | 46.66(46.07-47.26) | 0.69 (0.65, 0.73) | <0.001 |
| **Togo** | 44.27(43.31-45.22) | 49.26(48.64-49.87) | 0.34 (0.33, 0.35) | <0.001 |

Table S7. Regional AAPC in migraine lifetime risk with 95% confidence intervals, 1990-2021, males.

| **Region** | **Lifetime Risk 1990** | **Lifetime Risk 2021** | **AAPC(95%CI)** | **P value** |
| --- | --- | --- | --- | --- |
| **Global** | 38.04(38.00-38.08) | 41.99(41.96-42.02) | 0.32 (0.31, 0.33) | <0.001 |
| **High SDI** | 40.55(40.45-40.64) | 42.44(42.35-42.53) | 0.15 (0.14, 0.15) | <0.001 |
| **High-middle SDI** | 37.74(37.65-37.83) | 41.12(41.04-41.20) | 0.27 (0.26, 0.28) | <0.001 |
| **Middle SDI** | 39.20(39.13-39.27) | 43.50(43.44-43.56) | 0.33 (0.32, 0.34) | <0.001 |
| **Low-middle SDI** | 40.10(40.02-40.17) | 44.11(44.05-44.17) | 0.31 (0.3, 0.32) | <0.001 |
| **Low SDI** | 32.27(32.15-32.38) | 37.77(37.69-37.86) | 0.49 (0.42, 0.57) | <0.001 |
| **Andean Latin America** | 27.62(27.09-28.15) | 30.79(30.41-31.16) | 0.35 (0.34, 0.37) | <0.001 |
| **Bolivia (Plurinational State of)** | 27.65(26.46-28.85) | 30.47(29.63-31.31) | 0.3 (0.28, 0.34) | <0.001 |
| **Ecuador** | 32.88(31.84-33.91) | 34.56(33.83-35.28) | 0.16 (0.14, 0.18) | <0.001 |
| **Peru** | 25.22(24.51-25.94) | 28.94(28.42-29.45) | 0.46 (0.44, 0.47) | <0.001 |
| **Australasia** | 42.05(41.40-42.69) | 43.33(42.83-43.83) | 0.1 (0.09, 0.1) | <0.001 |
| **Australia** | 41.90(41.20-42.60) | 43.13(42.58-43.68) | 0.09 (0.09, 0.09) | <0.001 |
| **New Zealand** | 42.77(41.18-44.36) | 44.28(43.10-45.47) | 0.12 (0.11, 0.12) | <0.001 |
| **Caribbean** | 37.48(37.00-37.97) | 38.70(38.28-39.12) | 0.11 (-0.05, 0.26) | 0.159 |
| **Antigua and Barbuda** | 40.48(28.30-52.67) | 41.12(31.67-50.57) | 0.06 (0.05, 0.06) | <0.001 |
| **Bahamas** | 39.34(33.92-44.76) | 39.55(35.22-43.88) | 0.03 (0, 0.05) | 0.051 |
| **Barbados** | 40.24(34.31-46.17) | 41.11(35.57-46.65) | 0.07 (0.06, 0.08) | <0.001 |
| **Belize** | 39.17(31.37-46.98) | 40.12(35.78-44.46) | 0.09 (0.08, 0.1) | <0.001 |
| **Cuba** | 40.64(39.74-41.55) | 40.91(40.04-41.77) | 0.03 (0.02, 0.05) | <0.001 |
| **Dominica** | 40.02(28.61-51.43) | 39.53(28.46-50.60) | -0.03 (-0.04, -0.01) | 0.007 |
| **Dominican Republic** | 38.02(36.85-39.18) | 39.78(38.91-40.65) | 0.15 (0.14, 0.16) | <0.001 |
| **Grenada** | 39.50(28.91-50.09) | 40.02(31.55-48.49) | 0.04 (0.02, 0.05) | <0.001 |
| **Guyana** | 36.69(33.68-39.69) | 38.11(35.17-41.05) | 0.13 (0.11, 0.14) | <0.001 |
| **Haiti** | 32.22(31.25-33.20) | 36.15(35.42-36.88) | 0.34 (-0.24, 0.98) | 0.209 |
| **Jamaica** | 40.41(38.36-42.46) | 40.64(38.96-42.31) | 0.05 (0.03, 0.07) | <0.001 |
| **Saint Lucia** | 39.51(31.23-47.79) | 40.15(33.29-47.01) | 0.06 (0.05, 0.07) | <0.001 |
| **Saint Vincent and the Grenadines** | 39.29(30.03-48.56) | 40.23(31.70-48.76) | 0.08 (0.07, 0.1) | <0.001 |
| **Suriname** | 38.04(33.49-42.58) | 39.35(35.62-43.09) | 0.12 (0.11, 0.13) | <0.001 |
| **Trinidad and Tobago** | 39.34(36.69-41.98) | 39.71(37.22-42.20) | 0.05 (0.04, 0.06) | <0.001 |
| **Bermuda** | 40.42(29.28-51.57) | 41.64(29.59-53.68) | 0.1 (0.09, 0.11) | <0.001 |
| **Puerto Rico** | 39.87(38.36-41.38) | 41.31(39.56-43.05) | 0.12 (0.1, 0.14) | <0.001 |
| **Saint Kitts and Nevis** | 39.08(24.68-53.48) | 40.11(28.28-51.94) | 0.09 (0.09, 0.1) | <0.001 |
| **United States Virgin Islands** | 39.63(30.32-48.94) | 40.18(29.02-51.33) | 0.03 (0.02, 0.04) | <0.001 |
| **Central Asia** | 37.63(37.27-38.00) | 39.64(39.35-39.94) | 0.16 (0.14, 0.18) | <0.001 |
| **Armenia** | 38.98(37.34-40.62) | 40.86(39.19-42.54) | 0.08 (-0.12, 0.34) | 0.173 |
| **Azerbaijan** | 36.90(35.79-38.01) | 39.52(38.64-40.39) | 0.2 (0.16, 0.25) | <0.001 |
| **Georgia** | 38.51(37.21-39.81) | 40.07(38.46-41.68) | 0.19 (0.16, 0.21) | <0.001 |
| **Kazakhstan** | 37.96(37.22-38.69) | 39.51(38.85-40.17) | 0.13 (0.1, 0.14) | <0.001 |
| **Kyrgyzstan** | 37.18(35.77-38.59) | 39.92(38.81-41.02) | 0.23 (0.21, 0.24) | <0.001 |
| **Mongolia** | 35.99(33.98-38.01) | 39.30(37.74-40.87) | 0.29 (0.28, 0.3) | <0.001 |
| **Tajikistan** | 36.89(35.52-38.26) | 39.01(38.09-39.93) | 0.19 (0.07, 0.32) | 0.004 |
| **Turkmenistan** | 36.92(35.33-38.51) | 38.65(37.43-39.87) | 0.14 (0.13, 0.16) | <0.001 |
| **Uzbekistan** | 38.17(37.46-38.87) | 40.09(39.58-40.60) | 0.16 (0.15, 0.17) | <0.001 |
| **Central Europe** | 38.81(38.54-39.08) | 40.39(40.11-40.66) | 0.13 (0.12, 0.14) | <0.001 |
| **Albania** | 38.73(36.97-40.49) | 40.25(38.44-42.05) | 0.16 (0.05, 0.27) | 0.001 |
| **Bosnia and Herzegovina** | 39.38(37.99-40.77) | 40.31(38.72-41.90) | 0.12 (0.01, 0.22) | 0.031 |
| **Bulgaria** | 38.47(37.39-39.55) | 38.98(37.74-40.23) | 0.04 (0.03, 0.05) | <0.001 |
| **Croatia** | 39.15(37.78-40.52) | 40.49(39.08-41.89) | 0.15 (0.1, 0.19) | <0.001 |
| **Czechia** | 39.00(38.07-39.93) | 40.70(39.79-41.61) | 0.14 (0.13, 0.14) | <0.001 |
| **Hungary** | 38.16(37.23-39.08) | 40.02(39.07-40.97) | 0.16 (0.14, 0.17) | <0.001 |
| **North Macedonia** | 38.51(36.48-40.54) | 39.69(37.80-41.58) | 0.1 (0.08, 0.11) | <0.001 |
| **Montenegro** | 39.67(35.95-43.39) | 39.85(36.19-43.51) | 0.01 (0, 0.02) | <0.001 |
| **Poland** | 39.57(39.09-40.05) | 41.23(40.75-41.71) | 0.14 (0.13, 0.15) | <0.001 |
| **Romania** | 38.10(37.46-38.75) | 39.55(38.84-40.26) | 0.1 (0.09, 0.12) | <0.001 |
| **Serbia** | 38.30(37.33-39.28) | 40.25(39.36-41.14) | 0.18 (0.08, 0.32) | <0.001 |
| **Slovakia** | 38.68(37.41-39.94) | 40.01(38.75-41.27) | 0.12 (0.1, 0.13) | <0.001 |
| **Slovenia** | 39.44(37.28-41.60) | 41.25(39.25-43.25) | 0.15 (0.14, 0.17) | <0.001 |
| **Central Latin America** | 35.71(35.47-35.96) | 37.43(37.25-37.61) | 0.15 (0.13, 0.16) | <0.001 |
| **Colombia** | 36.03(35.50-36.55) | 38.45(38.01-38.89) | 0.21 (0.2, 0.23) | <0.001 |
| **Costa Rica** | 38.30(36.40-40.20) | 38.41(37.03-39.80) | 0.03 (0.02, 0.04) | <0.001 |
| **El Salvador** | 34.84(33.60-36.07) | 37.09(35.97-38.21) | 0.21 (0.18, 0.23) | <0.001 |
| **Guatemala** | 33.40(32.44-34.36) | 36.57(35.86-37.27) | 0.31 (0.28, 0.33) | <0.001 |
| **Honduras** | 35.41(33.94-36.88) | 37.15(36.23-38.07) | 0.16 (0.12, 0.22) | <0.001 |
| **Mexico** | 35.74(35.39-36.08) | 37.35(37.10-37.59) | 0.13 (0.11, 0.14) | <0.001 |
| **Nicaragua** | 36.02(34.22-37.83) | 37.74(36.64-38.83) | 0.15 (0.12, 0.18) | <0.001 |
| **Panama** | 37.77(35.63-39.91) | 38.45(36.92-39.97) | 0.05 (0.03, 0.06) | <0.001 |
| **Venezuela (Bolivarian Republic of)** | 36.10(35.37-36.82) | 36.21(35.64-36.78) | 0.01 (-0.03, 0.05) | 0.615 |
| **Central Sub-Saharan Africa** | 31.19(30.84-31.53) | 36.88(36.64-37.13) | 0.53 (0.51, 0.56) | <0.001 |
| **Angola** | 28.94(28.21-29.67) | 36.98(36.47-37.50) | 0.8 (0.72, 0.87) | <0.001 |
| **Central African Republic** | 28.85(27.49-30.22) | 32.24(31.20-33.28) | 0.37 (0.31, 0.44) | <0.001 |
| **Congo** | 33.57(31.90-35.23) | 38.00(36.79-39.22) | 0.45 (0.31, 0.61) | <0.001 |
| **Democratic Republic of the Congo** | 31.81(31.39-32.24) | 37.08(36.78-37.38) | 0.5 (0.46, 0.54) | <0.001 |
| **Equatorial Guinea** | 30.85(26.79-34.90) | 37.49(35.37-39.62) | 0.62 (0.6, 0.64) | <0.001 |
| **Gabon** | 35.45(32.61-38.29) | 38.25(36.10-40.41) | 0.24 (0.22, 0.25) | <0.001 |
| **East Asia** | 33.89(33.80-33.98) | 39.06(38.98-39.14) | 0.45 (0.44, 0.46) | <0.001 |
| **China** | 33.76(33.67-33.85) | 39.04(38.95-39.12) | 0.46 (0.45, 0.47) | <0.001 |
| **Democratic People's Republic of Korea** | 36.75(36.04-37.46) | 38.15(37.55-38.74) | 0.06 (-0.06, 0.19) | 0.327 |
| **Taiwan (Province of China)** | 38.71(38.02-39.40) | 41.90(41.33-42.48) | 0.26 (0.25, 0.27) | <0.001 |
| **Eastern Europe** | 36.94(36.73-37.15) | 37.85(37.64-38.07) | 0.07 (0.04, 0.09) | <0.001 |
| **Belarus** | 34.89(33.90-35.89) | 35.40(34.38-36.42) | 0.05 (0.02, 0.06) | <0.001 |
| **Estonia** | 34.53(31.90-37.16) | 36.77(33.98-39.57) | 0.2 (0.16, 0.23) | <0.001 |
| **Latvia** | 34.37(32.33-36.42) | 35.89(33.59-38.19) | 0.14 (0.11, 0.16) | <0.001 |
| **Lithuania** | 32.69(30.99-34.39) | 33.83(31.84-35.83) | 0.09 (0.06, 0.11) | <0.001 |
| **Republic of Moldova** | 34.19(32.68-35.71) | 35.63(33.99-37.26) | 0.13 (0.11, 0.16) | <0.001 |
| **Russian Federation** | 37.68(37.43-37.93) | 38.58(38.33-38.83) | 0.06 (0.02, 0.09) | 0.001 |
| **Ukraine** | 35.84(35.38-36.29) | 36.28(35.79-36.76) | 0.04 (0.02, 0.06) | <0.001 |
| **Eastern Sub-Saharan Africa** | 23.09(22.91-23.27) | 28.33(28.19-28.48) | 0.65 (0.42, 0.93) | <0.001 |
| **Burundi** | 23.12(22.07-24.16) | 28.69(27.84-29.54) | 0.7 (0.59, 0.81) | <0.001 |
| **Comoros** | 26.38(21.85-30.91) | 30.14(26.49-33.79) | 0.42 (0.39, 0.45) | <0.001 |
| **Djibouti** | 27.42(22.78-32.05) | 29.82(27.23-32.42) | 0.26 (0.24, 0.28) | <0.001 |
| **Eritrea** | 20.95(20.01-21.89) | 28.74(27.62-29.85) | 1.12 (0.99, 1.27) | <0.001 |
| **Ethiopia** | 20.26(19.96-20.55) | 28.00(27.70-28.31) | 1.04 (0.99, 1.08) | <0.001 |
| **Kenya** | 28.83(28.17-29.49) | 30.48(30.06-30.91) | 0.17 (0.16, 0.19) | <0.001 |
| **Madagascar** | 25.63(24.78-26.47) | 29.02(28.44-29.60) | 0.4 (0.39, 0.41) | <0.001 |
| **Malawi** | 22.41(21.65-23.17) | 27.99(27.35-28.64) | 0.72 (0.7, 0.75) | <0.001 |
| **Mozambique** | 22.63(21.91-23.36) | 27.08(26.58-27.57) | 0.59 (0.58, 0.61) | <0.001 |
| **Rwanda** | 23.54(22.63-24.45) | 29.75(28.90-30.60) | -0.03 (-1.52, 1.59) | 0.705 |
| **Somalia** | 22.50(21.69-23.31) | 25.78(25.22-26.33) | 0.54 (0.29, 0.77) | <0.001 |
| **United Republic of Tanzania** | 23.60(23.05-24.15) | 28.43(28.01-28.84) | 0.62 (0.6, 0.65) | <0.001 |
| **Uganda** | 22.75(22.21-23.28) | 28.19(27.74-28.65) | 0.71 (0.69, 0.73) | <0.001 |
| **Zambia** | 25.84(24.95-26.74) | 30.27(29.65-30.90) | 0.52 (0.5, 0.54) | <0.001 |
| **South Sudan** | 23.26(22.21-24.32) | 25.37(24.46-26.27) | 0.27 (0.22, 0.33) | <0.001 |
| **High-income Asia Pacific** | 31.08(30.84-31.32) | 33.42(33.19-33.64) | 0.24 (0.23, 0.24) | <0.001 |
| **Brunei Darussalam** | 31.10(24.78-37.41) | 32.02(28.04-35.99) | 0.1 (0.1, 0.1) | <0.001 |
| **Japan** | 29.77(29.48-30.05) | 32.60(32.32-32.89) | 0.3 (0.29, 0.3) | <0.001 |
| **Republic of Korea** | 33.93(33.50-34.36) | 35.31(34.92-35.70) | 0.13 (0.12, 0.15) | <0.001 |
| **Singapore** | 27.56(25.78-29.34) | 31.95(30.71-33.19) | 0.48 (0.47, 0.5) | <0.001 |
| **High-income North America** | 41.73(41.56-41.90) | 41.80(41.65-41.96) | 0 (-0.01, 0.01) | 0.712 |
| **Canada** | 38.27(37.71-38.83) | 39.14(38.65-39.64) | 0.07 (0.07, 0.08) | <0.001 |
| **United States of America** | 42.09(41.91-42.27) | 42.07(41.91-42.24) | -0.01 (-0.02, 0) | 0.136 |
| **Greenland** | 36.69(25.45-47.94) | 39.08(26.54-51.62) | 0.21 (0.2, 0.21) | <0.001 |
| **North Africa and Middle East** | 40.66(40.50-40.82) | 44.17(44.06-44.28) | 0.26 (0.26, 0.27) | <0.001 |
| **Algeria** | 42.41(41.76-43.06) | 44.83(44.41-45.25) | 0.17 (0.16, 0.18) | <0.001 |
| **Bahrain** | 43.85(40.16-47.54) | 45.42(43.58-47.27) | 0.12 (0.11, 0.12) | <0.001 |
| **Egypt** | 40.62(40.24-40.99) | 44.39(44.13-44.64) | 0.28 (0.27, 0.3) | <0.001 |
| **Iran (Islamic Republic of)** | 43.00(42.59-43.42) | 46.68(46.38-46.98) | 0.23 (0.22, 0.26) | <0.001 |
| **Iraq** | 41.19(40.49-41.88) | 43.85(43.44-44.27) | 0.21 (0.17, 0.25) | <0.001 |
| **Jordan** | 43.97(42.31-45.64) | 45.38(44.64-46.12) | 0.1 (0.09, 0.11) | <0.001 |
| **Kuwait** | 45.11(42.92-47.31) | 47.09(45.90-48.28) | 0.2 (0.15, 0.27) | <0.001 |
| **Lebanon** | 42.38(40.66-44.10) | 45.21(44.07-46.35) | 0.19 (0.16, 0.22) | <0.001 |
| **Libya** | 43.48(41.95-45.01) | 43.82(42.81-44.83) | 0.02 (-0.05, 0.08) | 0.331 |
| **Morocco** | 41.06(40.49-41.64) | 44.71(44.27-45.15) | 0.27 (0.26, 0.28) | <0.001 |
| **Palestine** | 42.78(40.40-45.16) | 44.98(43.69-46.28) | 0.13 (0.05, 0.2) | 0.005 |
| **Oman** | 42.84(40.91-44.77) | 44.90(43.78-46.02) | 0.15 (0.14, 0.16) | <0.001 |
| **Qatar** | 44.11(40.15-48.08) | 46.15(44.56-47.75) | 0.15 (0.14, 0.16) | <0.001 |
| **Saudi Arabia** | 43.00(42.33-43.67) | 45.76(45.38-46.14) | 0.19 (0.18, 0.21) | <0.001 |
| **Syrian Arab Republic** | 42.62(41.73-43.51) | 44.50(43.58-45.43) | 0.23 (0.13, 0.33) | <0.001 |
| **Tunisia** | 43.11(42.06-44.16) | 44.84(44.05-45.64) | 0.13 (0.12, 0.14) | <0.001 |
| **Turkey** | 39.32(38.94-39.70) | 43.38(43.08-43.68) | 0.31 (0.29, 0.32) | <0.001 |
| **United Arab Emirates** | 44.03(42.08-45.98) | 46.28(45.27-47.29) | 0.17 (0.16, 0.18) | <0.001 |
| **Yemen** | 37.18(36.42-37.93) | 41.49(41.01-41.97) | 0.37 (0.35, 0.39) | <0.001 |
| **Afghanistan** | 34.81(33.85-35.77) | 39.08(38.63-39.53) | 0.4 (0.34, 0.46) | <0.001 |
| **Sudan** | 36.13(35.53-36.73) | 42.77(42.34-43.20) | 0.53 (0.5, 0.55) | <0.001 |
| **Oceania** | 41.22(40.14-42.30) | 42.37(41.65-43.10) | 0.1 (0.09, 0.11) | <0.001 |
| **Fiji** | 43.64(40.51-46.77) | 43.94(41.12-46.76) | 0.03 (0.02, 0.04) | <0.001 |
| **Kiribati** | 39.59(29.75-49.43) | 42.37(34.52-50.22) | 0.23 (0.19, 0.25) | <0.001 |
| **Marshall Islands** | 42.04(28.82-55.25) | 43.44(32.32-54.56) | 0.11 (0.1, 0.11) | <0.001 |
| **Micronesia (Federated States of)** | 42.03(33.32-50.74) | 43.91(35.56-52.25) | 0.14 (0.13, 0.15) | <0.001 |
| **Papua New Guinea** | 40.44(39.07-41.80) | 42.03(41.20-42.86) | 0.14 (0.12, 0.16) | <0.001 |
| **Samoa** | 43.82(36.44-51.20) | 45.22(38.94-51.50) | 0.09 (0.06, 0.12) | <0.001 |
| **Solomon Islands** | 41.34(36.57-46.11) | 43.50(40.19-46.80) | 0.17 (0.16, 0.18) | <0.001 |
| **Tonga** | 44.67(34.76-54.58) | 45.60(36.44-54.75) | 0.06 (0, 0.11) | 0.042 |
| **Vanuatu** | 42.11(34.94-49.28) | 43.26(38.39-48.14) | 0.09 (0.08, 0.1) | <0.001 |
| **American Samoa** | 44.57(31.51-57.62) | 45.22(32.37-58.07) | 0.04 (0.03, 0.06) | <0.001 |
| **Cook Islands** | 44.06(22.35-65.77) | 46.23(23.64-68.82) | 0.12 (0.07, 0.16) | <0.001 |
| **Guam** | 45.61(37.91-53.31) | 45.71(38.65-52.77) | -0.02 (-0.08, 0.07) | 0.435 |
| **Nauru** | 41.52(14.76-68.27) | 42.19(16.88-67.49) | 0.05 (0.05, 0.06) | <0.001 |
| **Niue** | 44.10(-27.93-116.12) | 43.00(-43.25-129.26) | -0.1 (-0.13, -0.07) | <0.001 |
| **Northern Mariana Islands** | 45.35(33.07-57.64) | 45.60(33.15-58.05) | 0.03 (0.01, 0.04) | 0.004 |
| **Palau** | 43.12(21.36-64.88) | 44.52(24.83-64.22) | 0.11 (0.1, 0.11) | <0.001 |
| **Tokelau** | 44.29(-62.85-151.43) | 43.11(-55.88-142.09) | -0.13 (-0.2, -0.09) | <0.001 |
| **Tuvalu** | 39.61(9.61-69.60) | 44.06(19.12-69.00) | 0.33 (0.28, 0.39) | <0.001 |
| **South Asia** | 41.54(41.46-41.62) | 45.99(45.93-46.05) | 0.33 (0.32, 0.34) | <0.001 |
| **Bangladesh** | 40.03(39.77-40.28) | 46.96(46.75-47.17) | 0.55 (0.51, 0.59) | <0.001 |
| **Bhutan** | 40.55(37.21-43.89) | 47.23(44.24-50.22) | 0.5 (0.49, 0.51) | <0.001 |
| **India** | 41.68(41.59-41.77) | 46.04(45.98-46.11) | 0.32 (0.31, 0.33) | <0.001 |
| **Nepal** | 41.01(40.43-41.59) | 47.56(47.08-48.04) | 0.47 (0.46, 0.49) | <0.001 |
| **Pakistan** | 42.54(42.28-42.80) | 45.28(45.11-45.45) | 0.2 (0.19, 0.21) | <0.001 |
| **Southeast Asia** | 44.74(44.61-44.87) | 48.21(48.11-48.31) | 0.24 (0.22, 0.27) | <0.001 |
| **Cambodia** | 39.99(39.14-40.85) | 46.48(45.84-47.12) | 0.49 (0.48, 0.5) | <0.001 |
| **Indonesia** | 44.99(44.79-45.18) | 48.64(48.49-48.79) | 0.25 (0.21, 0.31) | <0.001 |
| **Lao People's Democratic Republic** | 36.98(35.77-38.18) | 46.09(45.12-47.05) | 0.71 (0.7, 0.71) | <0.001 |
| **Malaysia** | 46.56(45.87-47.25) | 47.82(47.37-48.27) | 0.11 (0.1, 0.13) | <0.001 |
| **Maldives** | 45.28(38.96-51.60) | 49.82(46.51-53.12) | 0.31 (0.29, 0.34) | <0.001 |
| **Myanmar** | 38.60(38.22-38.98) | 45.78(45.41-46.14) | 0.62 (0.56, 0.67) | <0.001 |
| **Philippines** | 46.22(45.86-46.57) | 48.12(47.88-48.36) | 0.14 (0.12, 0.16) | <0.001 |
| **Sri Lanka** | 46.15(45.47-46.84) | 49.09(48.51-49.67) | 0.23 (0.19, 0.27) | <0.001 |
| **Thailand** | 47.18(46.80-47.56) | 49.08(48.71-49.45) | 0.13 (0.12, 0.14) | <0.001 |
| **Timor-Leste** | 40.10(37.11-43.08) | 46.60(44.20-48.99) | 0.56 (0.46, 0.7) | <0.001 |
| **Viet Nam** | 45.78(45.43-46.14) | 48.35(48.10-48.61) | 0.18 (0.18, 0.18) | <0.001 |
| **Mauritius** | 47.15(44.84-49.47) | 48.19(45.85-50.53) | 0.05 (0.04, 0.07) | <0.001 |
| **Seychelles** | 47.15(37.36-56.95) | 48.61(42.01-55.20) | 0.09 (0.08, 0.1) | <0.001 |
| **Southern Latin America** | 33.81(33.36-34.26) | 35.59(35.21-35.96) | 0.16 (0.16, 0.17) | <0.001 |
| **Argentina** | 34.36(33.82-34.91) | 36.19(35.73-36.64) | 0.17 (0.16, 0.17) | <0.001 |
| **Chile** | 32.38(31.50-33.27) | 34.17(33.45-34.89) | 0.17 (0.16, 0.18) | <0.001 |
| **Uruguay** | 34.01(32.19-35.83) | 34.80(33.00-36.60) | 0.09 (0.08, 0.11) | <0.001 |
| **Southern Sub-Saharan Africa** | 37.02(36.65-37.39) | 37.29(37.00-37.58) | 0.02 (0, 0.03) | 0.019 |
| **Botswana** | 36.37(33.85-38.88) | 37.15(35.49-38.81) | 0.06 (0.02, 0.09) | 0.013 |
| **Lesotho** | 35.21(32.75-37.68) | 32.29(30.62-33.95) | -0.28 (-0.32, -0.24) | <0.001 |
| **Namibia** | 36.46(33.98-38.94) | 37.11(35.41-38.82) | 0.07 (0.03, 0.11) | 0.004 |
| **South Africa** | 37.30(36.87-37.74) | 38.14(37.79-38.48) | 0.07 (0.04, 0.09) | <0.001 |
| **Eswatini** | 35.56(32.19-38.93) | 34.57(32.35-36.80) | -0.1 (-0.13, -0.06) | <0.001 |
| **Zimbabwe** | 36.39(35.51-37.27) | 35.27(34.62-35.93) | -0.1 (-0.13, -0.08) | <0.001 |
| **Tropical Latin America** | 44.41(44.21-44.62) | 47.82(47.65-47.99) | 0.23 (0.21, 0.25) | <0.001 |
| **Brazil** | 44.37(44.16-44.57) | 47.89(47.72-48.06) | 0.24 (0.21, 0.26) | <0.001 |
| **Paraguay** | 45.80(44.36-47.23) | 46.01(45.07-46.94) | 0.01 (0, 0.03) | 0.033 |
| **Western Europe** | 46.63(46.50-46.76) | 48.03(47.92-48.15) | 0.1 (0.09, 0.1) | <0.001 |
| **Andorra** | 46.67(36.59-56.75) | 47.51(39.64-55.38) | 0.06 (0.06, 0.06) | <0.001 |
| **Austria** | 44.60(43.64-45.55) | 45.87(45.06-46.68) | 0.09 (0.09, 0.1) | <0.001 |
| **Belgium** | 48.92(48.13-49.71) | 50.77(50.09-51.44) | 0.12 (0.12, 0.12) | <0.001 |
| **Cyprus** | 46.02(43.19-48.85) | 47.42(45.45-49.39) | 0.1 (0.09, 0.1) | <0.001 |
| **Denmark** | 42.84(41.67-44.00) | 44.55(43.55-45.55) | 0.13 (0.12, 0.13) | <0.001 |
| **Finland** | 45.91(44.72-47.09) | 47.28(46.18-48.39) | 0.1 (0.09, 0.1) | <0.001 |
| **France** | 46.86(46.51-47.20) | 47.96(47.66-48.26) | 0.07 (0.07, 0.08) | <0.001 |
| **Germany** | 46.80(46.51-47.08) | 48.57(48.32-48.81) | 0.12 (0.11, 0.12) | <0.001 |
| **Greece** | 47.82(47.03-48.61) | 48.28(47.50-49.05) | 0.03 (0.03, 0.04) | <0.001 |
| **Iceland** | 46.59(41.31-51.86) | 47.55(43.29-51.82) | 0.06 (0.06, 0.07) | <0.001 |
| **Ireland** | 46.22(44.87-47.57) | 47.50(46.43-48.56) | 0.09 (0.08, 0.09) | <0.001 |
| **Israel** | 46.48(45.28-47.67) | 47.52(46.71-48.32) | 0.08 (0.07, 0.1) | <0.001 |
| **Italy** | 48.48(48.15-48.82) | 50.10(49.82-50.39) | 0.11 (0.08, 0.13) | <0.001 |
| **Luxembourg** | 45.61(41.31-49.91) | 47.20(44.54-49.86) | 0.11 (0.11, 0.12) | <0.001 |
| **Malta** | 46.45(42.57-50.33) | 47.37(43.71-51.03) | 0.06 (0.06, 0.07) | <0.001 |
| **Netherlands** | 43.91(43.26-44.57) | 46.51(45.94-47.08) | 0.18 (0.17, 0.19) | <0.001 |
| **Norway** | 45.29(43.99-46.59) | 48.82(47.84-49.80) | 0.24 (0.23, 0.25) | <0.001 |
| **Portugal** | 45.38(44.48-46.27) | 47.24(46.50-47.99) | 0.13 (0.13, 0.14) | <0.001 |
| **Spain** | 46.84(46.40-47.27) | 48.22(47.89-48.56) | 0.1 (0.09, 0.1) | <0.001 |
| **Sweden** | 46.45(45.58-47.32) | 47.74(46.98-48.49) | 0.09 (0.08, 0.09) | <0.001 |
| **Switzerland** | 43.86(42.80-44.92) | 45.48(44.70-46.27) | 0.12 (0.12, 0.13) | <0.001 |
| **United Kingdom** | 45.61(45.27-45.95) | 46.82(46.52-47.13) | 0.08 (0.08, 0.09) | <0.001 |
| **Monaco** | 46.51(30.86-62.15) | 46.86(32.15-61.57) | 0.02 (0.02, 0.03) | <0.001 |
| **San Marino** | 46.73(30.22-63.24) | 47.80(34.31-61.30) | 0.07 (0.07, 0.07) | <0.001 |
| **Western Sub-Saharan Africa** | 35.54(35.36-35.72) | 41.22(41.10-41.35) | 0.48 (0.47, 0.48) | <0.001 |
| **Benin** | 36.04(34.79-37.29) | 41.03(40.27-41.79) | 0.42 (0.41, 0.44) | <0.001 |
| **Burkina Faso** | 33.59(32.79-34.39) | 39.65(39.07-40.24) | 0.52 (0.5, 0.55) | <0.001 |
| **Cameroon** | 37.96(37.10-38.82) | 41.09(40.63-41.56) | 0.26 (0.25, 0.26) | <0.001 |
| **Cabo Verde** | 42.79(37.04-48.54) | 45.49(41.99-48.99) | 0.19 (0.17, 0.21) | <0.001 |
| **Chad** | 34.26(33.18-35.33) | 38.72(38.05-39.40) | 0.38 (0.36, 0.4) | <0.001 |
| **Côte d'Ivoire** | 36.55(35.86-37.25) | 41.36(40.86-41.86) | 0.39 (0.38, 0.41) | <0.001 |
| **Gambia** | 37.92(35.20-40.64) | 42.40(40.65-44.15) | 0.35 (0.34, 0.37) | <0.001 |
| **Ghana** | 38.80(38.09-39.51) | 42.71(42.25-43.17) | 0.3 (0.29, 0.31) | <0.001 |
| **Guinea** | 33.69(32.65-34.73) | 40.23(39.47-40.99) | 0.57 (0.54, 0.59) | <0.001 |
| **Guinea-Bissau** | 32.52(30.19-34.86) | 40.14(38.36-41.91) | 0.64 (0.6, 0.68) | <0.001 |
| **Liberia** | 30.17(28.83-31.50) | 41.79(40.65-42.93) | 1.05 (0.98, 1.15) | <0.001 |
| **Mali** | 32.47(31.63-33.30) | 39.43(38.86-40.00) | 0.6 (0.55, 0.64) | <0.001 |
| **Mauritania** | 39.94(37.94-41.94) | 44.57(43.12-46.02) | 0.35 (0.33, 0.36) | <0.001 |
| **Niger** | 30.81(30.00-31.63) | 40.57(39.96-41.17) | 0.92 (0.89, 0.97) | <0.001 |
| **Nigeria** | 35.60(35.35-35.85) | 41.70(41.52-41.89) | 0.52 (0.51, 0.53) | <0.001 |
| **Sao Tome and Principe** | 40.32(31.43-49.22) | 45.36(39.41-51.30) | 0.38 (0.36, 0.39) | <0.001 |
| **Senegal** | 37.82(36.81-38.84) | 43.34(42.63-44.05) | 0.43 (0.42, 0.44) | <0.001 |
| **Sierra Leone** | 32.37(31.22-33.52) | 39.96(39.06-40.85) | 0.73 (0.68, 0.79) | <0.001 |
| **Togo** | 37.64(36.17-39.11) | 41.84(40.90-42.78) | 0.34 (0.32, 0.35) | <0.001 |

Table S8. Regional AAPC in migraine lifetime risk with 95% confidence intervals, 1990-2021, females.

| **Region** | **Lifetime Risk 1990** | **Lifetime Risk 2021** | **AAPC(95%CI)** | **P value** |
| --- | --- | --- | --- | --- |
| **Global** | 55.72(55.69-55.75) | 59.84(59.81-59.86) | 0.23 (0.23, 0.23) | <0.001 |
| **High SDI** | 65.11(65.06-65.17) | 66.57(66.52-66.62) | 0.07 (0.07, 0.07) | <0.001 |
| **High-middle SDI** | 58.26(58.20-58.33) | 61.63(61.58-61.68) | 0.18 (0.18, 0.18) | <0.001 |
| **Middle SDI** | 57.40(57.35-57.46) | 62.04(62.00-62.08) | 0.25 (0.24, 0.25) | <0.001 |
| **Low-middle SDI** | 53.89(53.82-53.95) | 59.51(59.46-59.55) | 0.32 (0.32, 0.33) | <0.001 |
| **Low SDI** | 44.03(43.93-44.14) | 51.74(51.66-51.82) | 0.52 (0.5, 0.54) | <0.001 |
| **Andean Latin America** | 48.88(48.44-49.32) | 53.88(53.59-54.16) | 0.33 (0.32, 0.34) | <0.001 |
| **Bolivia (Plurinational State of)** | 47.91(46.92-48.89) | 53.21(52.54-53.88) | 0.34 (0.33, 0.35) | <0.001 |
| **Ecuador** | 55.25(54.45-56.06) | 57.61(57.10-58.12) | 0.13 (0.12, 0.14) | <0.001 |
| **Peru** | 46.26(45.64-46.88) | 52.08(51.68-52.47) | 0.4 (0.39, 0.42) | <0.001 |
| **Australasia** | 59.78(59.35-60.21) | 60.70(60.38-61.02) | 0.05 (0.05, 0.05) | <0.001 |
| **Australia** | 59.60(59.13-60.07) | 60.50(60.15-60.85) | 0.05 (0.05, 0.05) | <0.001 |
| **New Zealand** | 60.66(59.61-61.71) | 61.67(60.89-62.45) | 0.05 (0.05, 0.06) | <0.001 |
| **Caribbean** | 58.21(57.85-58.56) | 60.24(59.94-60.53) | 0.1 (-0.09, 0.3) | 0.256 |
| **Antigua and Barbuda** | 62.65(54.35-70.96) | 62.97(57.41-68.53) | 0.01 (0, 0.03) | 0.075 |
| **Bahamas** | 61.55(57.50-65.59) | 62.21(59.16-65.25) | 0.03 (-0.03, 0.11) | 0.166 |
| **Barbados** | 62.23(58.28-66.18) | 62.85(59.52-66.18) | 0.03 (0.02, 0.03) | <0.001 |
| **Belize** | 60.61(55.07-66.15) | 62.46(59.55-65.37) | 0.1 (0.09, 0.11) | <0.001 |
| **Cuba** | 62.54(61.94-63.14) | 63.30(62.78-63.83) | 0.04 (0.04, 0.05) | <0.001 |
| **Dominica** | 62.11(54.58-69.64) | 61.59(54.24-68.95) | -0.01 (-0.04, 0.01) | 0.321 |
| **Dominican Republic** | 59.01(58.19-59.82) | 61.99(61.41-62.57) | 0.15 (0.15, 0.16) | <0.001 |
| **Grenada** | 61.28(53.61-68.96) | 62.17(56.20-68.13) | 0.06 (0.05, 0.07) | <0.001 |
| **Guyana** | 58.57(56.17-60.96) | 60.74(58.43-63.05) | 0.12 (0.11, 0.13) | <0.001 |
| **Haiti** | 50.37(49.59-51.15) | 56.48(55.91-57.06) | 0.33 (-0.46, 1.2) | 0.337 |
| **Jamaica** | 61.76(60.41-63.11) | 62.46(61.35-63.56) | 0.04 (0.03, 0.04) | <0.001 |
| **Saint Lucia** | 61.49(55.69-67.30) | 62.51(58.19-66.84) | 0.06 (0.05, 0.06) | <0.001 |
| **Saint Vincent and the Grenadines** | 60.92(54.08-67.76) | 62.27(56.26-68.28) | 0.07 (0.07, 0.08) | <0.001 |
| **Suriname** | 59.65(56.22-63.07) | 61.46(58.79-64.13) | 0.1 (0.09, 0.1) | <0.001 |
| **Trinidad and Tobago** | 61.39(59.57-63.21) | 62.32(60.67-63.97) | 0.05 (0.04, 0.05) | <0.001 |
| **Bermuda** | 62.96(55.78-70.14) | 63.97(57.24-70.70) | 0.05 (0.05, 0.06) | <0.001 |
| **Puerto Rico** | 62.73(61.73-63.73) | 63.90(62.98-64.83) | 0.06 (0.03, 0.1) | <0.001 |
| **Saint Kitts and Nevis** | 60.35(49.12-71.58) | 62.45(55.17-69.73) | 0.11 (0.1, 0.11) | <0.001 |
| **United States Virgin Islands** | 62.21(56.33-68.09) | 63.70(57.35-70.05) | 0.07 (0.07, 0.08) | <0.001 |
| **Central Asia** | 57.93(57.66-58.20) | 60.20(60.00-60.40) | 0.12 (0.11, 0.12) | <0.001 |
| **Armenia** | 59.22(58.12-60.31) | 61.53(60.54-62.51) | 0.1 (0.06, 0.16) | <0.001 |
| **Azerbaijan** | 56.77(55.98-57.55) | 59.93(59.35-60.51) | 0.18 (0.17, 0.19) | <0.001 |
| **Georgia** | 59.35(58.50-60.19) | 61.11(60.14-62.09) | 0.1 (0.08, 0.1) | <0.001 |
| **Kazakhstan** | 59.11(58.58-59.64) | 60.65(60.21-61.10) | 0.08 (0.08, 0.09) | <0.001 |
| **Kyrgyzstan** | 57.68(56.58-58.78) | 60.65(59.87-61.42) | 0.16 (0.15, 0.16) | <0.001 |
| **Mongolia** | 55.29(53.71-56.87) | 60.42(59.33-61.51) | 0.29 (0.29, 0.3) | <0.001 |
| **Tajikistan** | 55.99(54.93-57.04) | 59.19(58.53-59.85) | 0.17 (0.14, 0.2) | <0.001 |
| **Turkmenistan** | 56.82(55.60-58.03) | 59.29(58.35-60.23) | 0.14 (0.13, 0.14) | <0.001 |
| **Uzbekistan** | 58.22(57.70-58.75) | 60.23(59.89-60.57) | 0.11 (0.1, 0.11) | <0.001 |
| **Central Europe** | 61.24(61.07-61.41) | 62.68(62.52-62.84) | 0.08 (0.07, 0.08) | <0.001 |
| **Albania** | 59.82(58.61-61.04) | 61.85(60.78-62.92) | 0.1 (0.1, 0.11) | <0.001 |
| **Bosnia and Herzegovina** | 61.29(60.43-62.14) | 62.17(61.24-63.09) | 0.05 (0.03, 0.08) | <0.001 |
| **Bulgaria** | 60.70(60.01-61.38) | 61.31(60.55-62.08) | 0.04 (0.03, 0.04) | <0.001 |
| **Croatia** | 61.51(60.68-62.34) | 62.54(61.73-63.36) | 0.06 (0.04, 0.08) | <0.001 |
| **Czechia** | 61.44(60.86-62.01) | 62.57(62.04-63.10) | 0.06 (0.06, 0.06) | <0.001 |
| **Hungary** | 60.74(60.13-61.34) | 62.09(61.54-62.65) | 0.07 (0.07, 0.08) | <0.001 |
| **North Macedonia** | 59.55(58.23-60.88) | 61.63(60.45-62.81) | 0.11 (0.1, 0.12) | <0.001 |
| **Montenegro** | 61.50(59.14-63.86) | 61.97(59.78-64.17) | 0.02 (0.02, 0.03) | <0.001 |
| **Poland** | 62.67(62.37-62.97) | 63.92(63.65-64.19) | 0.07 (0.06, 0.07) | <0.001 |
| **Romania** | 60.04(59.62-60.46) | 61.82(61.40-62.23) | 0.09 (0.08, 0.1) | <0.001 |
| **Serbia** | 60.46(59.84-61.07) | 62.08(61.53-62.63) | 0.1 (0.08, 0.11) | <0.001 |
| **Slovakia** | 61.27(60.45-62.09) | 62.13(61.39-62.86) | 0.05 (0.04, 0.06) | <0.001 |
| **Slovenia** | 61.80(60.47-63.12) | 62.95(61.82-64.08) | 0.06 (0.06, 0.07) | <0.001 |
| **Central Latin America** | 60.96(60.79-61.13) | 63.10(62.98-63.21) | 0.1 (0.1, 0.11) | <0.001 |
| **Colombia** | 62.11(61.75-62.47) | 64.09(63.84-64.35) | 0.1 (0.1, 0.11) | <0.001 |
| **Costa Rica** | 63.25(62.06-64.45) | 63.84(63.04-64.64) | 0.03 (0.03, 0.04) | <0.001 |
| **El Salvador** | 60.09(59.13-61.05) | 63.22(62.49-63.95) | 0.17 (0.16, 0.18) | <0.001 |
| **Guatemala** | 57.06(56.25-57.87) | 61.89(61.40-62.38) | 0.27 (0.26, 0.28) | <0.001 |
| **Honduras** | 59.23(58.10-60.36) | 62.10(61.50-62.70) | 0.17 (0.12, 0.24) | <0.001 |
| **Mexico** | 60.98(60.75-61.21) | 63.04(62.89-63.20) | 0.1 (0.09, 0.11) | <0.001 |
| **Nicaragua** | 60.32(59.12-61.53) | 63.15(62.44-63.86) | 0.16 (0.13, 0.2) | <0.001 |
| **Panama** | 62.46(61.07-63.85) | 63.53(62.62-64.44) | 0.05 (0.05, 0.06) | <0.001 |
| **Venezuela (Bolivarian Republic of)** | 61.42(60.93-61.91) | 62.53(62.16-62.90) | 0.06 (-0.03, 0.18) | 0.096 |
| **Central Sub-Saharan Africa** | 43.36(43.03-43.68) | 50.79(50.57-51.02) | 0.51 (0.5, 0.52) | <0.001 |
| **Angola** | 41.06(40.34-41.79) | 50.83(50.37-51.29) | 0.7 (0.68, 0.72) | <0.001 |
| **Central African Republic** | 41.13(39.78-42.47) | 46.22(45.22-47.22) | 0.37 (0.36, 0.39) | <0.001 |
| **Congo** | 46.77(45.19-48.36) | 51.31(50.26-52.35) | 0.29 (0.27, 0.32) | <0.001 |
| **Democratic Republic of the Congo** | 43.85(43.45-44.24) | 51.04(50.76-51.31) | 0.49 (0.48, 0.5) | <0.001 |
| **Equatorial Guinea** | 43.54(39.91-47.17) | 50.86(48.72-53.00) | 0.51 (0.49, 0.53) | <0.001 |
| **Gabon** | 49.97(47.22-52.72) | 52.82(51.01-54.63) | 0.17 (0.15, 0.18) | <0.001 |
| **East Asia** | 52.75(52.68-52.82) | 58.83(58.77-58.88) | 0.35 (0.34, 0.36) | <0.001 |
| **China** | 52.57(52.49-52.64) | 58.83(58.77-58.88) | 0.37 (0.36, 0.38) | <0.001 |
| **Democratic People's Republic of Korea** | 55.70(55.20-56.21) | 57.57(57.14-57.99) | 0.05 (-0.06, 0.16) | 0.369 |
| **Taiwan (Province of China)** | 59.89(59.38-60.40) | 60.87(60.48-61.27) | 0.05 (0.05, 0.06) | <0.001 |
| **Eastern Europe** | 60.24(60.10-60.37) | 60.88(60.74-61.02) | 0.03 (0.02, 0.04) | <0.001 |
| **Belarus** | 59.01(58.37-59.65) | 59.67(59.03-60.31) | 0.04 (0.03, 0.05) | <0.001 |
| **Estonia** | 58.92(57.24-60.59) | 60.42(58.72-62.13) | 0.09 (0.07, 0.1) | <0.001 |
| **Latvia** | 58.76(57.45-60.08) | 59.97(58.54-61.39) | 0.07 (0.06, 0.07) | <0.001 |
| **Lithuania** | 58.79(57.70-59.89) | 59.80(58.62-60.98) | 0.06 (0.05, 0.06) | <0.001 |
| **Republic of Moldova** | 57.71(56.72-58.70) | 59.41(58.40-60.42) | 0.09 (0.08, 0.1) | <0.001 |
| **Russian Federation** | 60.48(60.31-60.65) | 61.04(60.87-61.20) | 0.02 (0.01, 0.03) | <0.001 |
| **Ukraine** | 60.20(59.92-60.48) | 60.78(60.48-61.09) | 0.03 (0.02, 0.04) | <0.001 |
| **Eastern Sub-Saharan Africa** | 35.02(34.83-35.21) | 42.26(42.11-42.40) | 0.6 (0.55, 0.64) | <0.001 |
| **Burundi** | 34.73(33.66-35.79) | 42.36(41.51-43.22) | 0.63 (0.6, 0.67) | <0.001 |
| **Comoros** | 38.74(34.43-43.05) | 43.65(40.31-46.98) | 0.38 (0.37, 0.39) | <0.001 |
| **Djibouti** | 40.63(35.75-45.51) | 43.61(41.00-46.22) | 0.21 (0.2, 0.23) | <0.001 |
| **Eritrea** | 35.76(34.34-37.18) | 43.01(41.88-44.13) | 0.63 (0.61, 0.66) | <0.001 |
| **Ethiopia** | 31.41(31.06-31.76) | 41.20(40.89-41.50) | 0.87 (0.86, 0.89) | <0.001 |
| **Kenya** | 41.61(40.96-42.27) | 45.02(44.62-45.43) | 0.25 (0.24, 0.26) | <0.001 |
| **Madagascar** | 37.62(36.80-38.45) | 42.35(41.80-42.90) | 0.38 (0.37, 0.39) | <0.001 |
| **Malawi** | 33.14(32.38-33.91) | 41.95(41.31-42.60) | 0.76 (0.74, 0.78) | <0.001 |
| **Mozambique** | 34.10(33.40-34.79) | 40.63(40.13-41.13) | 0.57 (0.56, 0.59) | <0.001 |
| **Rwanda** | 35.44(34.48-36.40) | 43.75(42.96-44.53) | 0.15 (-0.66, 0.85) | 0.669 |
| **Somalia** | 34.33(33.41-35.26) | 39.33(38.71-39.95) | 0.51 (0.28, 0.73) | <0.001 |
| **United Republic of Tanzania** | 37.08(36.54-37.61) | 44.09(43.71-44.47) | 0.57 (0.55, 0.58) | <0.001 |
| **Uganda** | 34.24(33.70-34.78) | 42.26(41.79-42.72) | 0.69 (0.67, 0.7) | <0.001 |
| **Zambia** | 36.29(35.40-37.17) | 43.19(42.56-43.81) | 0.58 (0.56, 0.6) | <0.001 |
| **South Sudan** | 35.28(34.12-36.44) | 38.23(37.33-39.13) | 0.25 (0.23, 0.26) | <0.001 |
| **High-income Asia Pacific** | 55.32(55.17-55.48) | 56.31(56.17-56.45) | 0.06 (0.05, 0.06) | <0.001 |
| **Brunei Darussalam** | 52.83(47.73-57.92) | 53.66(50.64-56.68) | 0.05 (0.05, 0.05) | <0.001 |
| **Japan** | 54.96(54.79-55.14) | 56.00(55.83-56.18) | 0.06 (0.06, 0.07) | <0.001 |
| **Republic of Korea** | 56.54(56.21-56.87) | 57.47(57.21-57.72) | 0.06 (0.05, 0.07) | <0.001 |
| **Singapore** | 47.56(46.19-48.93) | 52.03(51.18-52.87) | 0.29 (0.28, 0.29) | <0.001 |
| **High-income North America** | 69.85(69.77-69.94) | 69.74(69.66-69.82) | -0.01 (-0.01, 0) | 0.002 |
| **Canada** | 68.88(68.60-69.16) | 69.23(69.00-69.46) | 0.01 (0.01, 0.02) | <0.001 |
| **United States of America** | 69.96(69.87-70.05) | 69.80(69.72-69.88) | -0.01 (-0.01, -0.01) | <0.001 |
| **Greenland** | 64.83(56.81-72.85) | 67.68(60.67-74.68) | 0.14 (0.14, 0.14) | <0.001 |
| **North Africa and Middle East** | 58.08(57.96-58.20) | 63.08(63.00-63.15) | 0.26 (0.26, 0.27) | <0.001 |
| **Algeria** | 59.92(59.45-60.38) | 62.86(62.58-63.14) | 0.15 (0.15, 0.16) | <0.001 |
| **Bahrain** | 61.77(58.66-64.89) | 63.52(61.92-65.13) | 0.09 (0.09, 0.1) | <0.001 |
| **Egypt** | 57.03(56.75-57.31) | 64.05(63.88-64.23) | 0.37 (0.37, 0.38) | <0.001 |
| **Iran (Islamic Republic of)** | 60.53(60.22-60.84) | 66.65(66.47-66.84) | 0.29 (0.26, 0.34) | <0.001 |
| **Iraq** | 59.41(58.88-59.95) | 62.49(62.19-62.79) | 0.17 (0.15, 0.18) | <0.001 |
| **Jordan** | 61.14(59.93-62.36) | 63.52(62.98-64.06) | 0.12 (0.12, 0.12) | <0.001 |
| **Kuwait** | 62.61(60.64-64.59) | 64.23(63.30-65.16) | 0.09 (0.08, 0.11) | <0.001 |
| **Lebanon** | 61.18(59.98-62.37) | 63.76(63.02-64.50) | 0.12 (0.12, 0.13) | <0.001 |
| **Libya** | 61.20(60.01-62.38) | 62.18(61.47-62.89) | 0.04 (0.02, 0.05) | 0.003 |
| **Morocco** | 58.08(57.66-58.51) | 62.81(62.53-63.10) | 0.25 (0.25, 0.25) | <0.001 |
| **Palestine** | 60.66(59.10-62.21) | 63.39(62.52-64.26) | 0.15 (0.14, 0.18) | <0.001 |
| **Oman** | 61.25(59.47-63.03) | 63.47(62.45-64.50) | 0.11 (0.11, 0.12) | <0.001 |
| **Qatar** | 62.45(58.36-66.54) | 64.24(62.77-65.72) | 0.09 (0.09, 0.09) | <0.001 |
| **Saudi Arabia** | 58.81(58.16-59.46) | 61.97(61.64-62.31) | 0.16 (0.16, 0.17) | <0.001 |
| **Syrian Arab Republic** | 60.28(59.61-60.96) | 63.09(62.60-63.58) | 0.16 (0.14, 0.18) | <0.001 |
| **Tunisia** | 60.75(60.02-61.48) | 63.47(62.97-63.98) | 0.14 (0.14, 0.15) | <0.001 |
| **Turkey** | 59.53(59.27-59.80) | 64.09(63.91-64.27) | 0.24 (0.23, 0.25) | <0.001 |
| **United Arab Emirates** | 61.96(59.95-63.96) | 63.32(62.52-64.11) | 0.07 (0.07, 0.08) | <0.001 |
| **Yemen** | 53.11(52.49-53.73) | 60.11(59.73-60.49) | 0.4 (0.4, 0.41) | <0.001 |
| **Afghanistan** | 48.92(48.25-49.59) | 57.50(57.08-57.91) | 0.54 (0.52, 0.58) | <0.001 |
| **Sudan** | 51.73(51.26-52.20) | 60.81(60.49-61.13) | 0.52 (0.51, 0.53) | <0.001 |
| **Oceania** | 55.49(54.54-56.43) | 57.08(56.46-57.70) | 0.1 (0.08, 0.12) | <0.001 |
| **Fiji** | 58.86(56.21-61.50) | 59.24(56.98-61.51) | 0.02 (0.02, 0.03) | <0.001 |
| **Kiribati** | 53.64(45.19-62.10) | 57.73(51.32-64.14) | 0.23 (0.23, 0.25) | <0.001 |
| **Marshall Islands** | 57.91(45.44-70.38) | 58.56(49.20-67.91) | 0.03 (0.03, 0.04) | <0.001 |
| **Micronesia (Federated States of)** | 57.27(49.44-65.10) | 59.50(52.58-66.41) | 0.12 (0.1, 0.15) | <0.001 |
| **Papua New Guinea** | 54.26(53.05-55.46) | 56.54(55.82-57.27) | 0.14 (0.12, 0.18) | <0.001 |
| **Samoa** | 59.15(52.98-65.33) | 60.07(55.06-65.09) | 0.03 (0, 0.05) | 0.034 |
| **Solomon Islands** | 56.43(52.12-60.75) | 58.88(56.11-61.65) | 0.14 (0.13, 0.15) | <0.001 |
| **Tonga** | 59.59(52.02-67.16) | 60.91(54.21-67.61) | 0.07 (0.06, 0.08) | <0.001 |
| **Vanuatu** | 57.71(51.32-64.11) | 59.20(55.15-63.25) | 0.08 (0.07, 0.09) | <0.001 |
| **American Samoa** | 60.29(49.20-71.39) | 60.42(50.79-70.06) | 0 (-0.02, 0.02) | 0.992 |
| **Cook Islands** | 59.31(41.39-77.23) | 61.97(47.52-76.43) | 0.12 (0.04, 0.21) | <0.001 |
| **Guam** | 61.00(54.97-67.04) | 61.53(56.72-66.34) | 0.03 (0.01, 0.05) | 0.008 |
| **Nauru** | 57.10(32.55-81.65) | 57.98(35.85-80.10) | 0.05 (0.05, 0.05) | <0.001 |
| **Niue** | 59.76(5.76-113.75) | 57.10(-10.71-124.90) | -0.16 (-0.21, -0.12) | <0.001 |
| **Northern Mariana Islands** | 60.54(48.91-72.18) | 61.20(51.73-70.66) | 0.03 (0.03, 0.04) | <0.001 |
| **Palau** | 58.24(38.74-77.74) | 59.34(39.65-79.03) | 0.06 (0.06, 0.06) | <0.001 |
| **Tokelau** | 58.54(-12.34-129.43) | 56.33(-20.84-133.49) | -0.18 (-0.27, -0.14) | <0.001 |
| **Tuvalu** | 55.01(31.48-78.53) | 59.70(38.67-80.72) | 0.24 (0.21, 0.28) | <0.001 |
| **South Asia** | 53.74(53.67-53.81) | 59.97(59.92-60.02) | 0.36 (0.36, 0.37) | <0.001 |
| **Bangladesh** | 51.10(50.87-51.33) | 59.79(59.63-59.95) | 0.54 (0.48, 0.62) | <0.001 |
| **Bhutan** | 51.27(48.25-54.28) | 59.94(57.54-62.34) | 0.51 (0.5, 0.52) | <0.001 |
| **India** | 54.29(54.21-54.37) | 60.65(60.60-60.71) | 0.36 (0.36, 0.37) | <0.001 |
| **Nepal** | 51.18(50.68-51.68) | 60.26(59.90-60.61) | 0.53 (0.5, 0.55) | <0.001 |
| **Pakistan** | 53.06(52.83-53.29) | 56.85(56.71-57.00) | 0.24 (0.23, 0.26) | <0.001 |
| **Southeast Asia** | 60.38(60.28-60.48) | 64.24(64.17-64.31) | 0.19 (0.17, 0.24) | <0.001 |
| **Cambodia** | 54.55(53.89-55.21) | 62.29(61.82-62.77) | 0.43 (0.42, 0.43) | <0.001 |
| **Indonesia** | 59.54(59.38-59.69) | 64.43(64.32-64.54) | 0.24 (0.19, 0.33) | <0.001 |
| **Lao People's Democratic Republic** | 50.58(49.58-51.58) | 61.55(60.80-62.29) | 0.63 (0.63, 0.64) | <0.001 |
| **Malaysia** | 60.35(59.81-60.89) | 61.86(61.52-62.21) | 0.09 (0.08, 0.09) | <0.001 |
| **Maldives** | 59.06(54.00-64.12) | 65.00(62.15-67.86) | 0.31 (0.3, 0.33) | <0.001 |
| **Myanmar** | 53.51(53.18-53.83) | 61.85(61.59-62.11) | 0.51 (0.44, 0.6) | <0.001 |
| **Philippines** | 62.35(62.06-62.63) | 64.41(64.22-64.59) | 0.11 (0.1, 0.12) | <0.001 |
| **Sri Lanka** | 62.91(62.37-63.46) | 65.03(64.66-65.40) | 0.12 (0.07, 0.17) | <0.001 |
| **Thailand** | 66.57(66.33-66.82) | 66.97(66.75-67.18) | 0.02 (0.01, 0.03) | <0.001 |
| **Timor-Leste** | 53.31(50.87-55.74) | 61.75(59.90-63.60) | 0.48 (0.46, 0.52) | <0.001 |
| **Viet Nam** | 62.06(61.80-62.32) | 64.70(64.52-64.87) | 0.13 (0.13, 0.14) | <0.001 |
| **Mauritius** | 63.31(61.42-65.20) | 64.23(62.66-65.80) | 0.04 (0.04, 0.05) | <0.001 |
| **Seychelles** | 63.50(55.36-71.63) | 64.01(57.98-70.04) | 0.03 (0.03, 0.04) | <0.001 |
| **Southern Latin America** | 54.97(54.65-55.29) | 56.87(56.62-57.12) | 0.11 (0.11, 0.11) | <0.001 |
| **Argentina** | 54.01(53.61-54.41) | 56.12(55.81-56.42) | 0.12 (0.12, 0.13) | <0.001 |
| **Chile** | 56.98(56.40-57.57) | 58.74(58.30-59.17) | 0.1 (0.09, 0.1) | <0.001 |
| **Uruguay** | 56.19(54.99-57.40) | 57.26(56.15-58.37) | 0.07 (0.06, 0.08) | <0.001 |
| **Southern Sub-Saharan Africa** | 51.21(50.84-51.57) | 51.77(51.51-52.04) | 0.03 (0.01, 0.05) | 0.016 |
| **Botswana** | 50.54(48.19-52.89) | 51.06(49.58-52.54) | 0.02 (0, 0.05) | 0.077 |
| **Lesotho** | 50.02(47.97-52.08) | 46.48(44.90-48.05) | -0.26 (-0.31, -0.21) | <0.001 |
| **Namibia** | 50.62(48.35-52.90) | 51.80(50.27-53.32) | 0.07 (0.05, 0.09) | <0.001 |
| **South Africa** | 51.58(51.15-52.01) | 52.78(52.47-53.10) | 0.06 (0.04, 0.08) | <0.001 |
| **Eswatini** | 50.30(47.31-53.30) | 48.66(46.56-50.76) | -0.12 (-0.16, -0.08) | <0.001 |
| **Zimbabwe** | 49.75(48.90-50.59) | 49.30(48.71-49.89) | -0.02 (-0.04, 0) | 0.06 |
| **Tropical Latin America** | 67.41(67.28-67.54) | 70.34(70.24-70.44) | 0.13 (0.12, 0.14) | <0.001 |
| **Brazil** | 67.39(67.26-67.52) | 70.39(70.29-70.49) | 0.14 (0.13, 0.14) | <0.001 |
| **Paraguay** | 67.69(66.77-68.62) | 68.78(68.18-69.37) | 0.05 (0.05, 0.06) | <0.001 |
| **Western Europe** | 69.55(69.48-69.62) | 70.43(70.37-70.49) | 0.04 (0.04, 0.04) | <0.001 |
| **Andorra** | 69.06(62.61-75.51) | 69.81(65.62-74.01) | 0.03 (0.03, 0.04) | <0.001 |
| **Austria** | 67.68(67.16-68.21) | 68.59(68.17-69.01) | 0.04 (0.04, 0.05) | <0.001 |
| **Belgium** | 70.63(70.19-71.07) | 72.12(71.77-72.47) | 0.07 (0.07, 0.07) | <0.001 |
| **Cyprus** | 68.33(66.72-69.93) | 69.69(68.66-70.71) | 0.06 (0.06, 0.06) | <0.001 |
| **Denmark** | 65.87(65.20-66.55) | 67.33(66.79-67.88) | 0.07 (0.07, 0.07) | <0.001 |
| **Finland** | 68.90(68.25-69.54) | 69.69(69.12-70.27) | 0.04 (0.04, 0.04) | <0.001 |
| **France** | 68.41(68.21-68.61) | 68.84(68.68-69.00) | 0.02 (0.02, 0.02) | <0.001 |
| **Germany** | 69.84(69.69-70.00) | 70.97(70.84-71.10) | 0.05 (0.05, 0.05) | <0.001 |
| **Greece** | 69.20(68.79-69.62) | 69.80(69.41-70.19) | 0.03 (0.03, 0.03) | <0.001 |
| **Iceland** | 68.95(65.96-71.95) | 69.68(67.49-71.87) | 0.04 (0.02, 0.05) | <0.001 |
| **Ireland** | 68.73(67.95-69.50) | 69.66(69.10-70.22) | 0.04 (0.04, 0.04) | <0.001 |
| **Israel** | 68.61(67.93-69.29) | 69.75(69.31-70.20) | 0.05 (0.05, 0.06) | <0.001 |
| **Italy** | 72.01(71.84-72.18) | 73.13(72.99-73.27) | 0.05 (0.03, 0.06) | <0.001 |
| **Luxembourg** | 67.66(65.19-70.13) | 68.65(67.07-70.23) | 0.05 (0.04, 0.05) | <0.001 |
| **Malta** | 68.76(66.56-70.97) | 69.49(67.57-71.42) | 0.03 (0.03, 0.03) | <0.001 |
| **Netherlands** | 67.99(67.61-68.37) | 69.38(69.07-69.68) | 0.07 (0.06, 0.07) | <0.001 |
| **Norway** | 68.98(68.25-69.71) | 71.48(70.96-72.00) | 0.11 (0.11, 0.12) | <0.001 |
| **Portugal** | 68.27(67.79-68.74) | 69.66(69.26-70.05) | 0.07 (0.06, 0.07) | <0.001 |
| **Spain** | 69.29(69.05-69.52) | 70.18(70.00-70.36) | 0.04 (0.04, 0.04) | <0.001 |
| **Sweden** | 70.99(70.53-71.45) | 71.73(71.35-72.10) | 0.03 (0.03, 0.03) | <0.001 |
| **Switzerland** | 67.02(66.43-67.61) | 67.97(67.53-68.41) | 0.04 (0.04, 0.05) | <0.001 |
| **United Kingdom** | 69.54(69.36-69.72) | 70.37(70.21-70.52) | 0.04 (0.04, 0.04) | <0.001 |
| **Monaco** | 68.92(60.64-77.21) | 69.23(61.73-76.74) | 0.01 (0.01, 0.01) | <0.001 |
| **San Marino** | 69.09(60.00-78.18) | 69.92(63.19-76.65) | 0.04 (0.04, 0.04) | <0.001 |
| **Western Sub-Saharan Africa** | 47.11(46.96-47.27) | 54.48(54.37-54.58) | 0.47 (0.46, 0.48) | <0.001 |
| **Benin** | 47.86(46.82-48.90) | 54.54(53.87-55.20) | 0.43 (0.42, 0.43) | <0.001 |
| **Burkina Faso** | 44.64(43.95-45.33) | 52.87(52.37-53.37) | 0.53 (0.52, 0.55) | <0.001 |
| **Cameroon** | 50.03(49.30-50.76) | 54.46(54.04-54.88) | 0.27 (0.26, 0.28) | <0.001 |
| **Cabo Verde** | 56.33(51.97-60.70) | 60.08(57.31-62.85) | 0.21 (0.19, 0.23) | <0.001 |
| **Chad** | 45.49(44.59-46.38) | 51.39(50.80-51.98) | 0.4 (0.39, 0.41) | <0.001 |
| **Côte d'Ivoire** | 48.99(48.31-49.68) | 54.91(54.45-55.37) | 0.36 (0.35, 0.38) | <0.001 |
| **Gambia** | 51.19(48.66-53.72) | 56.03(54.47-57.58) | 0.29 (0.28, 0.3) | <0.001 |
| **Ghana** | 50.90(50.29-51.51) | 56.50(56.11-56.89) | 0.33 (0.32, 0.34) | <0.001 |
| **Guinea** | 43.79(42.95-44.64) | 53.23(52.59-53.88) | 0.63 (0.61, 0.64) | <0.001 |
| **Guinea-Bissau** | 45.61(43.48-47.73) | 54.18(52.57-55.78) | 0.55 (0.52, 0.57) | <0.001 |
| **Liberia** | 42.67(41.31-44.03) | 54.53(53.51-55.56) | 0.93 (0.86, 1.01) | <0.001 |
| **Mali** | 42.83(42.13-43.53) | 52.46(51.96-52.96) | 0.65 (0.63, 0.66) | <0.001 |
| **Mauritania** | 51.90(50.23-53.58) | 57.73(56.59-58.86) | 0.34 (0.34, 0.35) | <0.001 |
| **Niger** | 40.03(39.33-40.73) | 53.32(52.80-53.84) | 0.95 (0.93, 0.98) | <0.001 |
| **Nigeria** | 47.49(47.26-47.72) | 54.84(54.69-54.99) | 0.47 (0.46, 0.48) | <0.001 |
| **Sao Tome and Principe** | 52.76(45.53-59.98) | 58.93(54.13-63.72) | 0.36 (0.35, 0.37) | <0.001 |
| **Senegal** | 50.25(49.39-51.12) | 56.92(56.32-57.51) | 0.4 (0.39, 0.41) | <0.001 |
| **Sierra Leone** | 43.30(42.29-44.30) | 52.64(51.84-53.43) | 0.67 (0.63, 0.7) | <0.001 |
| **Togo** | 49.83(48.60-51.06) | 55.63(54.84-56.43) | 0.36 (0.35, 0.36) | <0.001 |

Table S9. Projected global migraine lifetime risk (%) with 95% confidence intervals, 2020-2050.

| Year | Predicted lifetime risk (95% CI) |
| --- | --- |
| 2020 | 51.86 (51.73-51.99) |
| 2021 | 52.03 (51.81-52.26) |
| 2022 | 52.21 (51.88-52.54) |
| 2023 | 52.39 (51.95-52.83) |
| 2024 | 52.57 (52-53.13) |
| 2025 | 52.74 (52.05-53.44) |
| 2026 | 52.92 (52.09-53.75) |
| 2027 | 53.1 (52.12-54.07) |
| 2028 | 53.28 (52.15-54.4) |
| 2029 | 53.45 (52.17-54.74) |
| 2030 | 53.63 (52.18-55.08) |
| 2031 | 53.81 (52.18-55.43) |
| 2032 | 53.99 (52.18-55.79) |
| 2033 | 54.16 (52.17-56.15) |
| 2034 | 54.34 (52.16-56.52) |
| 2035 | 54.52 (52.14-56.89) |
| 2036 | 54.7 (52.12-57.27) |
| 2037 | 54.87 (52.09-57.66) |
| 2038 | 55.05 (52.05-58.05) |
| 2039 | 55.23 (52.01-58.44) |
| 2040 | 55.4 (51.97-58.84) |
| 2041 | 55.58 (51.92-59.25) |
| 2042 | 55.76 (51.86-59.66) |
| 2043 | 55.94 (51.8-60.07) |
| 2044 | 56.11 (51.74-60.49) |
| 2045 | 56.29 (51.67-60.92) |
| 2046 | 56.47 (51.59-61.34) |
| 2047 | 56.65 (51.52-61.78) |
| 2048 | 56.82 (51.43-62.21) |
| 2049 | 57 (51.35-62.66) |
| 2050 | 57.18 (51.26-63.1) |

Table S10. Concentration index of migraine lifetime risk (%) with 95% confidence intervals, 1990-2021, both sexes.

| **Year** | Concentration index (95% CI) |
| --- | --- |
| **1990** | 0.35 (0.28, 0.41) |
| **1991** | 0.35 (0.29, 0.42) |
| **1992** | 0.36 (0.29, 0.43) |
| **1993** | 0.36 (0.29, 0.43) |
| **1994** | 0.24 (0.19, 0.29) |
| **1995** | 0.36 (0.29, 0.43) |
| **1996** | 0.35 (0.28, 0.42) |
| **1997** | 0.34 (0.28, 0.41) |
| **1998** | 0.34 (0.27, 0.41) |
| **1999** | 0.33 (0.27, 0.4) |
| **2000** | 0.33 (0.26, 0.4) |
| **2001** | 0.33 (0.26, 0.39) |
| **2002** | 0.33 (0.26, 0.4) |
| **2003** | 0.33 (0.26, 0.39) |
| **2004** | 0.33 (0.26, 0.39) |
| **2005** | 0.32 (0.26, 0.39) |
| **2006** | 0.32 (0.25, 0.39) |
| **2007** | 0.32 (0.25, 0.39) |
| **2008** | 0.31 (0.24, 0.38) |
| **2009** | 0.31 (0.24, 0.37) |
| **2010** | 0.28 (0.22, 0.34) |
| **2011** | 0.24 (0.18, 0.3) |
| **2012** | 0.28 (0.21, 0.34) |
| **2013** | 0.29 (0.22, 0.35) |
| **2014** | 0.29 (0.22, 0.35) |
| **2015** | 0.28 (0.22, 0.35) |
| **2016** | 0.28 (0.21, 0.34) |
| **2017** | 0.27 (0.21, 0.33) |
| **2018** | 0.27 (0.2, 0.33) |
| **2019** | 0.26 (0.2, 0.33) |
| **2020** | 0.27 (0.2, 0.34) |
| **2021** | 0.27 (0.21, 0.34) |

Table S11. Concentration index of migraine lifetime risk (%) with 95% confidence intervals, 1990-2021, males.

| **Year** | Concentration index (95% CI) |
| --- | --- |
| **1990** | 0.27 (0.2, 0.34) |
| **1991** | 0.27 (0.2, 0.35) |
| **1992** | 0.28 (0.2, 0.35) |
| **1993** | 0.28 (0.2, 0.36) |
| **1994** | 0.18 (0.13, 0.23) |
| **1995** | 0.27 (0.19, 0.34) |
| **1996** | 0.27 (0.19, 0.34) |
| **1997** | 0.26 (0.19, 0.34) |
| **1998** | 0.26 (0.19, 0.33) |
| **1999** | 0.26 (0.18, 0.33) |
| **2000** | 0.25 (0.18, 0.32) |
| **2001** | 0.25 (0.17, 0.32) |
| **2002** | 0.25 (0.17, 0.32) |
| **2003** | 0.24 (0.17, 0.32) |
| **2004** | 0.24 (0.17, 0.32) |
| **2005** | 0.24 (0.17, 0.32) |
| **2006** | 0.24 (0.16, 0.32) |
| **2007** | 0.23 (0.16, 0.31) |
| **2008** | 0.23 (0.15, 0.3) |
| **2009** | 0.22 (0.15, 0.3) |
| **2010** | 0.21 (0.14, 0.29) |
| **2011** | 0.17 (0.11, 0.24) |
| **2012** | 0.2 (0.13, 0.27) |
| **2013** | 0.21 (0.13, 0.28) |
| **2014** | 0.2 (0.13, 0.28) |
| **2015** | 0.2 (0.13, 0.27) |
| **2016** | 0.2 (0.12, 0.27) |
| **2017** | 0.19 (0.12, 0.26) |
| **2018** | 0.19 (0.11, 0.26) |
| **2019** | 0.18 (0.11, 0.26) |
| **2020** | 0.19 (0.11, 0.26) |
| **2021** | 0.19 (0.12, 0.27) |

Table S12. Concentration index of migraine lifetime risk (%) with 95% confidence intervals, 1990-2021, females.

| **Year** | **Concentration index (95% CI)** |
| --- | --- |
| **1990** | 0.4 (0.33, 0.46) |
| **1991** | 0.4 (0.34, 0.47) |
| **1992** | 0.4 (0.34, 0.47) |
| **1993** | 0.4 (0.34, 0.47) |
| **1994** | 0.3 (0.24, 0.35) |
| **1995** | 0.4 (0.33, 0.47) |
| **1996** | 0.4 (0.33, 0.46) |
| **1997** | 0.39 (0.32, 0.46) |
| **1998** | 0.39 (0.32, 0.45) |
| **1999** | 0.38 (0.31, 0.44) |
| **2000** | 0.38 (0.31, 0.44) |
| **2001** | 0.38 (0.31, 0.44) |
| **2002** | 0.37 (0.31, 0.44) |
| **2003** | 0.37 (0.31, 0.44) |
| **2004** | 0.37 (0.31, 0.44) |
| **2005** | 0.37 (0.31, 0.44) |
| **2006** | 0.37 (0.31, 0.44) |
| **2007** | 0.37 (0.31, 0.44) |
| **2008** | 0.37 (0.31, 0.44) |
| **2009** | 0.37 (0.3, 0.43) |
| **2010** | 0.31 (0.25, 0.37) |
| **2011** | 0.29 (0.23, 0.34) |
| **2012** | 0.33 (0.27, 0.39) |
| **2013** | 0.35 (0.28, 0.41) |
| **2014** | 0.34 (0.28, 0.41) |
| **2015** | 0.34 (0.28, 0.4) |
| **2016** | 0.33 (0.27, 0.39) |
| **2017** | 0.32 (0.26, 0.39) |
| **2018** | 0.32 (0.26, 0.38) |
| **2019** | 0.32 (0.25, 0.38) |
| **2020** | 0.32 (0.26, 0.39) |
| **2021** | 0.32 (0.26, 0.39) |

Figure S1. Lifetime and age-conditional probability of developing migraine in the 21 areas of the world for both sexes, 2021

Figure S2. Lifetime and age-conditional probability of developing migraine in the 21 areas of the world for males, 2021

Figure S3. Lifetime and age-conditional probability of developing migraine in the 21 areas of the world for females, 2021

Figure S4 Female-to-male ratios of migraine lifetime risk by temporal and age patterns across SDI levels.

(A) Temporal trends in female-to-male lifetime risk ratios from 1990 to 2020. (B) Age-specific female-to-male lifetime risk ratios across different SDI levels.
